# General α‐Amino 1,3,4‐Oxadiazole Synthesis via Late‐Stage Reductive Functionalization of Tertiary Amides and Lactams**

**DOI:** 10.1002/anie.202107536

**Published:** 2021-08-03

**Authors:** Daniel Matheau‐Raven, Darren J. Dixon

**Affiliations:** ^1^ Chemistry Research Laboratory Department of Chemistry University of Oxford 12 Mansfield Road Oxford UK

**Keywords:** carboxylic acids, C−C coupling, heterodiazoles, iridium, tertiary amides

## Abstract

An iridium‐catalyzed reductive three‐component coupling reaction for the synthesis of medicinally relevant α‐amino 1,3,4‐oxadiazoles from abundant tertiary amides or lactams, carboxylic acids, and (N‐isocyanimino) triphenylphosphorane, is described. Proceeding under mild conditions using (<1 mol %) Vaska's complex (IrCl(CO)(PPh_3_)_2_) and tetramethyldisiloxane to access the key reactive iminium ion intermediates, a broad range of α‐amino 1,3,4‐oxadiazole architectures were accessed from carboxylic acid feedstock coupling partners. Extension to α‐amino heterodiazole synthesis was readily achieved by exchanging the carboxylic acid coupling partner for *C*‐, *S*‐, or *N*‐centered Brønsted acids, and provided rapid and modular access to these desirable, yet difficult‐to‐access, heterocycles. The high chemoselectivity of the catalytic reductive activation step allowed late‐stage functionalization of 10 drug molecules, including the synthesis of heterodiazole‐fused drug–drug conjugates.

## Graphical abstract

An iridium‐catalyzed reductive three‐component coupling reaction for the synthesis of medicinally relevant and structurally complex α‐amino 1,3,4‐oxadiazoles, from tertiary amides, carboxylic acids, and (N‐isocyanimino) triphenylphosphorane, is described. Extension to heterodiazole synthesis was readily achieved as was application to the late‐stage functionalization of 10 APIs.

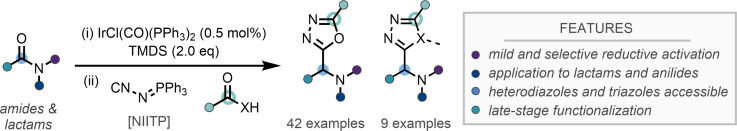

The 1,3,4‐oxadiazoles, and related heterodiazoles, are privileged heterocyclic structures in medicinal chemistry (Figure 1). As heteroaromatic bioisosteres for esters and amides, they conserve hydrogen bonding networks within receptor sites, while providing hydrolytic stability and favourable metabolic and pharmacokinetic properties.^[1]^ Examples in pharmaceutical compounds include HIV anti‐retroviral Raltegravir, and clinical candidates targetting cystic fibrosis (PTI‐428) and Huntington's disease (GSK‐356278). Continuing investigations into their biological activities as antimicrobial, anticancer, anticonvulsants, and anti‐inflammatory agents (amongst others) highlight their significant and growing impact within drug discovery programs.^[2]^ Beyond simple bioisosteres, α‐amino 1,3,4‐oxadiazoles are also universal peptidomimetics capable of mimicking any local pair of amino acids, in any secondary structure,^[3]^ a feature which has been exploited through their use as a conformation stabilising unit within peptide macrocycles.[^3b^, ^3c^]

**Figure 1:**
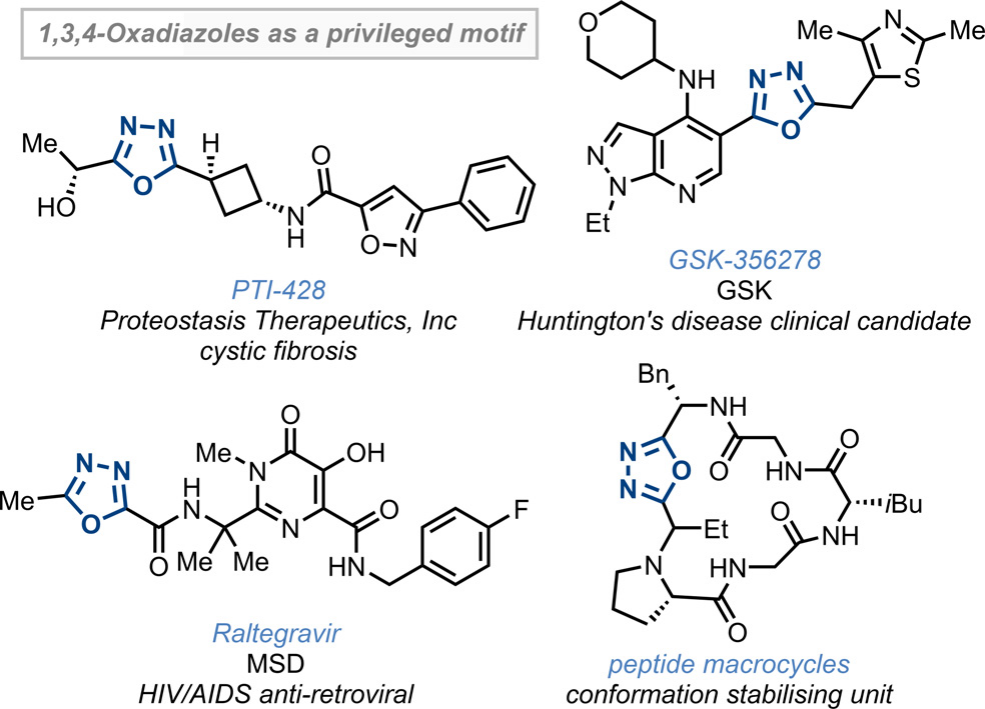
1,3,4‐Oxadiazoles as privileged structural motifs.

Traditional syntheses of α‐amino 1,3,4‐oxadiazoles are largely based on indirect condensation approaches where the oxadiazole unit is constructed through dehydration of a 1,2‐diacylhydrazine.[^2k^, ^4^] The need for multistep synthesis, and harsh dehydration conditions typically dictates that the oxadiazole be introduced at an early stage in the synthetic sequence, and thus a direct and late‐stage introduction of this motif would be both desirable and enabling.^[5]^

Tertiary amides, and lactams, abundant in pharmaceutical compounds, are commonly considered inert functional groups. However, in recent years, reports from Nagashima,^[6]^ Huang,^[7]^ Chida and Sato,^[8]^ and our group,^[9]^ among others,^[10]^ have shown that tertiary amides can act efficiently as robust and ubiquitous iminium ion, and enamine, precursors.^[11]^ The dormant amide can be reductively activated with exquisite chemoselectivity by Vaska's complex (IrCl(CO)(PPh_3_)_2_), at low catalyst loadings (typically < 1 mol%), and 1,1,3,3‐tetramethyldisiloxane (TMDS), affording an *O*‐silylated hemiaminal which is smoothly transformed into a reactive iminium ion upon treatment with Brønsted or Lewis acids.

Advancing our group's program on reductive amide functionalization, the late‐stage synthesis of α‐amino 1,3,4‐oxadiazoles presented an attractive and unsolved challenge. Previous work on related Ugi‐type reactions highlighted that reductively‐generated iminium ions can engage with isocyanide nucleophiles.[^7b^, ^9g^] As such, and inspired by work from Ramazani,[^5a^, ^5b^, ^5c^] we hypothesized that treatment of a reductively‐generated *O*‐silylated hemiaminal with the functionalized isocyanide (*N*‐isocyanimino) triphenylphosphorane (NIITP),^[12]^ in conjunction with a carboxylic acid (or appropriate *C*‐, *S*‐, or *N*‐centered Brønsted acid), would efficiently afford the α‐amino 1,3,4‐oxadiazoles (or heterodiazole) products in a late‐stage three‐component coupling reaction, following the mechanism proposed in Scheme 1. Nitrogen heterocycles, and their saturated analogues are essential elements of numerous pharmaceuticals;^[13]^ and through the reductive functionalization of lactams our strategy would enable the synthesis of valuable α‐functionalized heterocycles in a divergent manner. Furthermore, this strategy would allow the late‐stage functionalization (LSF) of amide or lactams, and carboxylic acid containing active pharmaceutical ingredients (APIs), providing α‐amino 1,3,4‐oxadiazoles with complex structural and chemical features. Herein we wish to report our findings.

**Scheme 1:**
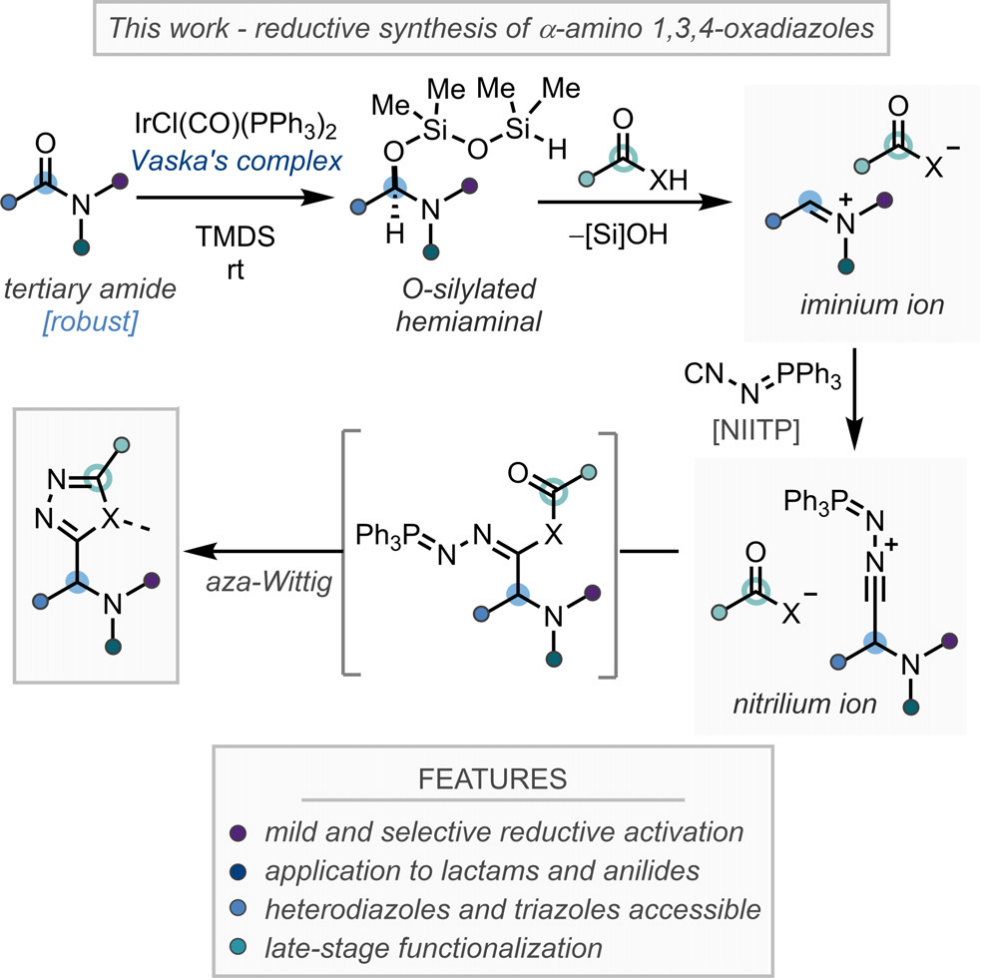
General reductive approach to α‐amino 1,3,4‐oxadiazoles, and heterodiazoles.

As late‐stage access to saturated nitrogen heterocycles possessing an α‐1,3,4‐oxadiazole moiety was unprecedented, *N*‐benzyl lactam (**1**) was chosen as a model substrate. Pleasingly, treatment of a THF solution of **1** with 0.5 mol % Vaska's complex, and 2 equiv TMDS, followed by addition of 2 equiv of NIITP and benzoic acid, afforded, on the first attempt, the desired α‐amino 1,3,4‐oxadiazole **2** in 78 % isolated yield (Scheme 2, entry 1). The formation of **3** as a side‐product, arising from the excess of NIITP and benzoic acid in the reaction mixture, complicated purification of **2**.^[14]^ Lowering the equivalents of NIITP and benzoic acid as well as reducing the reaction time for the second stage from 16 h to 1 h, delivered **2** in 87 % yield with no formation of **3** observed by NMR analysis of the crude reaction product (Scheme 2, entry 2). A solvent screen for the amide reduction subsequently revealed THF to be optimal (Scheme 2, entries 3 and 4).

**Scheme 2:**
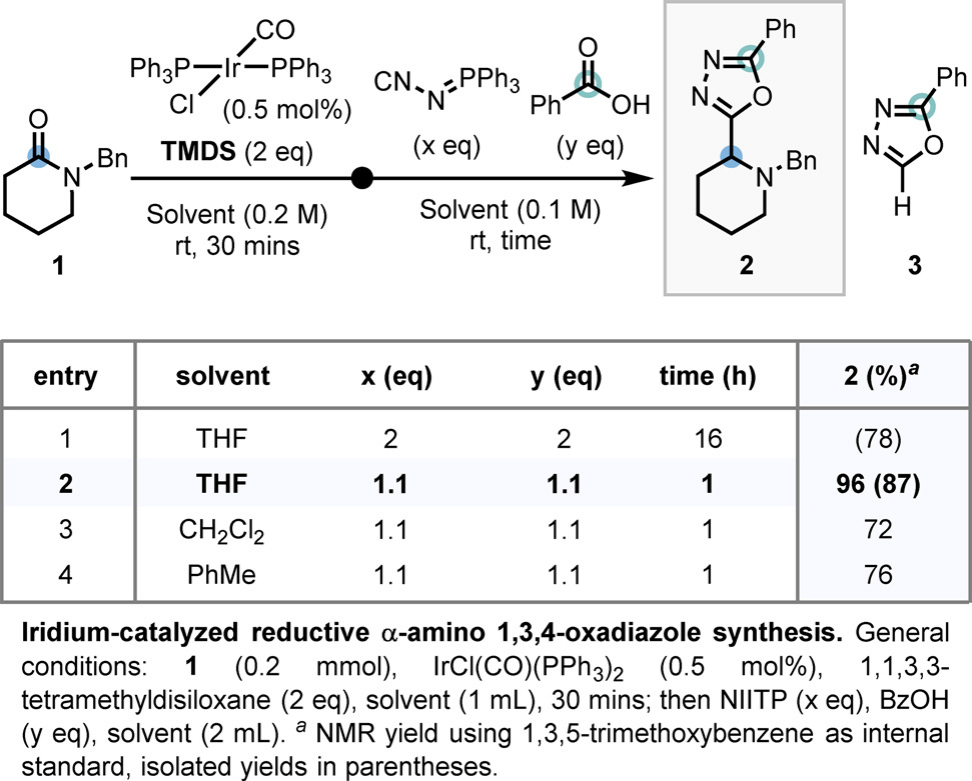
Reaction discovery and optimization.

With optimal conditions in hand, we investigated the scope of the reaction with respect to the carboxylic acid component (Scheme 3). We found that acetic acid (**4**), and 1‐phenyl‐1‐cyclopropanecarboxylic acid (**5**) gave excellent yields of the desired products. *N*‐Boc glycine (**6**) could be successfully employed affording a product with two orthogonally protected amines. A 1,3,4‐oxadiazole functionalized with a terminal alkyne (**7**) was readily synthesized by use of propynoic acid. A trifluoromethyl 1,3,4‐oxadiazole (**8**) was synthesized using trifluoroacetic acid, however an excess of amide was required for acceptable yield. Heteroaryl carboxylic acids could be employed, showcasing the tolerance of the reaction to the presence of Lewis basic functionalities, producing **9**–**11**. Carboxylic acids bearing electrophilic substituents including; aldehydes (**12**), sulfonyl fluorides (**13**), trifluoromethyl alkenes (**14**), allyl bromides (**15**), and alkyl bromides (**16**), acted as efficient coupling partners providing products with additional sites for further nucleophilic functionalization. Pyruvic acid (**17**), and an oxalic acid half ester (**18**) reacted successfully affording acyl α‐amino 1,3,4‐oxadiazoles in 48 % and 57 % yield, respectively. The late‐stage functionalization of two carboxylic acid containing APIs (**19**, **20**) was realized, demonstrating the potential application of this methodology in drug discovery programmes.

**Scheme 3:**
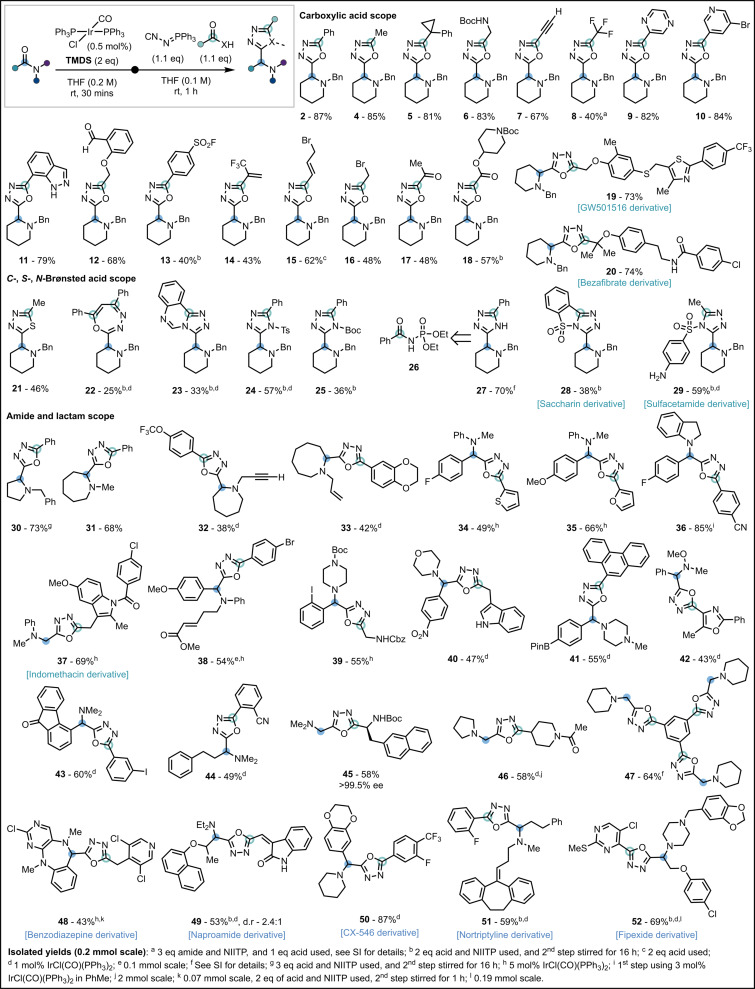
Reaction scope.

Encouraged by these results we hypothesized that a range of *C*‐, *S*‐, and *N*‐centered Brønsted acidic reaction partners could be suitable replacements for a carboxylic acid and produce distinct α‐amino heterodiazole scaffolds. Pleasingly, use of thioacetic acid afforded the α‐amino 1,3,4‐thiadiazole (**21**) thus granting access to a single‐atom modification of the parent oxadiazole.^[15]^ A 1,3‐dicarbonyl compound (1,3‐diphenyl‐1,3‐propanedione) proved suitably acidic allowing facile access to a unique α‐amino 1,3,4‐oxadiazepine scaffold (**22**).^[16]^ Our investigation of *N*‐H Brønsted acids started with use of 4‐hydroxyquinazoline and gave the α‐amino [1,2,4]triazolo[4,3‐c]quinazoline (**23**) in 33 % yield.^[17]^ Building upon this result, *N*‐acyl sulfonamides (**24**) and *N*‐acyl carbamates (**25**) were reacted to afford *N*‐functionalized α‐amino 1,2,4‐triazoles.^[18]^ Fortuitously, the product deriving from *N*‐acyl phosphonate ester (**26**) proved susceptible to mild hydrolysis, allowing for isolation of the *N*‐H α‐amino 1,2,4‐triazole (**27**) in a one‐pot procedure. These examples demonstrate the first use of protected amides as *N*‐H Brønsted acids for the assembly of desirable α‐amino triazoles using NIITP. *N*‐Acyl sulfonamides are a valued motif in the pharmaceutical industry, and successful functionalization of saccharin (**28**) and sulfacetamide (**29**) demonstrates, to our knowledge, the first use of this functional group for LSF.^[19]^ Notably, compounds such as benzamide, glutarimide, and *N*‐hydroxy benzamide gave complex reaction mixtures and failed to yield the desired heterodiazole, highlighting that acidification of the acyl *N*‐H by an appropriate electron‐withdrawing group is essential.

Next studied was the scope with respect to the amide component. Lactams with ring sizes from 5–8 (**2**, **30**–**33**) gave good yields of the desired products and showed excellent chemoselectivity for tertiary amide activation in the presence of terminal alkynes (**32**) and alkenes (**33**). The reactivity of simple anilides was assessed using derivatives of *N*‐methylaniline, and indoline, and successfully delivered *N*‐arylated α‐amino 1,3,4‐oxadiazoles (**34**–**36**). Complex, and functionalized, α‐amino 1,3,4‐oxadiazole structures containing anilines were synthesized by coupling of *N*‐methyl‐*N*‐phenylformamide with the anti‐inflammatory drug indomethacin (**37**), and chemoselective reductive functionalization of an α,β‐unsaturated ester‐containing anilide (**38**). The chemoselectivity of the method was further explored with amides bearing reactive, and reducible, functional groups such as aryl iodides and carbamates (**39**), nitro groups (**40**), boronic esters (**41**), *N*−*O* bonds (**42**),^[8a]^ and ketones (**43**) providing the desired α‐amino 1,3,4‐oxadiazoles without any observable reduction of these potentially sensitive functionalities. An aliphatic tertiary amide was successfully engaged (**44**), without complications arising from possible enamine reaction intermediates. Commercially available formamides were functionalized into a range of valorized products including; an enantiopure α‐amino 1,3,4‐oxadiazole (**45**), a tertiary amide (**46**) and a symmetric triamine (**47**) from coupling with benzene‐1,3,5‐tricarboxylic acid. The method was also applied to the LSF of five tertiary amide containing pharmaceutical molecules: a benzodiazepine scaffold (**48**), naproamide (**49**), CX‐546 (**50**), a derivative of nortiptyline (**51**), and fipexide (**52**) demonstrating excellent selectivity for tertiary amides, and efficient reactivity, in the presence of intricate chemical functionality.

The practicality of our chemistry was demonstrated by subjecting drug candidate CX‐546 and API probenecid to the standard reaction conditions on gram scale, affording the oxadiazole‐fused drug‐drug conjugate (**53**) in 80 % yield (Scheme 4). Investigation of structure–activity relationships (SAR) and synthesis of analogues is a critical process in drug discovery. As such, the capability of our method for the rapid production of heterodiazole analogues of **53** was examined; pleasingly, drug‐drug conjugates featuring heterodiazoles (**54**, **55**) were readily constructed using our standard conditions in combination with *N*‐tosyl probenecid (**56**), and thio‐probenecid (**57**), which were each readily available in high yield from the parent carboxylic acid.

**Scheme 4:**
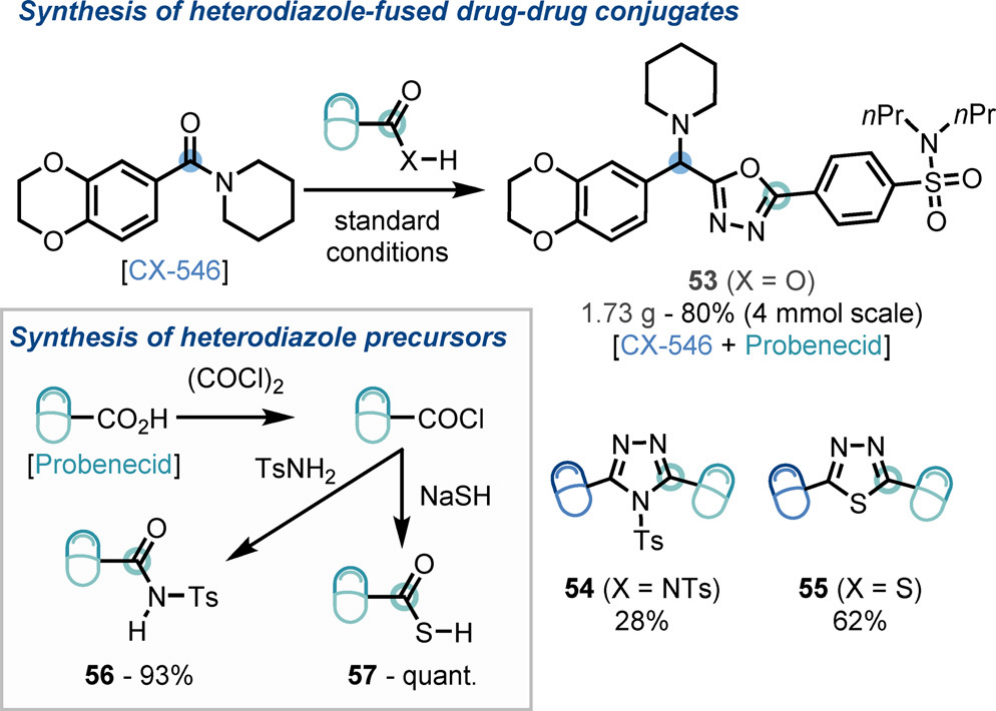
Scale‐up and synthesis of heterodiazole‐fused drug‐drug conjugates.

A new broad scope iridium‐catalyzed reductive three‐component coupling reaction for the synthesis of medicinally relevant α‐amino 1,3,4‐oxadiazoles from tertiary amides or lactams, carboxylic acids, and (N‐isocyanimino) triphenylphosphorane, has been developed. The reaction exhibits excellent chemoselectivity, and functional group tolerance for structurally diverse carboxylic acid and amide (or lactam) coupling partners, demonstrated by the LSF of 10 drug molecules. Furthermore, a subtle change of reaction conditions allowed ready extension to α‐amino heterodiazole synthesis, and rapid production of heterodiazole‐fused drug–drug conjugates.

## Conflict of interest

The authors declare no conflict of interest.

## Supporting information

As a service to our authors and readers, this journal provides supporting information supplied by the authors. Such materials are peer reviewed and may be re‐organized for online delivery, but are not copy‐edited or typeset. Technical support issues arising from supporting information (other than missing files) should be addressed to the authors.

Supporting Information
