# Supplementary material for: General α‐Amino 1,3,4‐Oxadiazole Synthesis via Late‐Stage Reductive Functionalization of Tertiary Amides and Lactams
Source: Angew Chem Int Ed Engl. 2021 Aug 3;60(36):19725–9. doi: 10.1002/anie.202107536 (PMC8457168; doi:10.1002/anie.202107536)
Supplement: Supplementary file 1 — Supporting Information [file ANIE-60-19725-s001.pdf]

Angewandte Chemie
Eine Zeitschrift der Gesellschaft Deutscher Chemiker

Supporting Information

General α-Amino 1,3,4-Oxadiazole Synthesis via Late-Stage Reductive Functionalization of Tertiary Amides and Lactams**

*Daniel Matheau-Raven and Darren J. Dixon**

anie_202107536_sm_miscellaneous_information.pdf

1. General information

General Techniques: Reactions were carried out under a nitrogen atmosphere unless stated otherwise. Glassware was oven-dried and cooled under vacuum then purged with nitrogen before use. Room temperature refers to 22 ± 2 °C. Inert atmosphere techniques, such as Schlenk technique, were used for the handling of air/moisture sensitive reagents. Reactions carried out at 0 °C were cooled using an ice bath. Reactions carried out at high temperatures were heated using an oil bath or, preferably, using DrySyn® heating blocks. Reaction temperatures refer to external temperatures, for example of a heating block or oil bath, not of internal reaction temperatures unless stated otherwise.

Nomenclature and Numbering: Compounds are named following IUPAC nomenclature as generated by ACD LABS or ChemDraw.

Solvents and Reagents: Ether refers to diethyl ether. Where solvent dryness was important, CH_2Cl_2, Et_2O, methanol, THF, and toluene were either obtained from dry solvent bottles with septa (Aldrich), an MBRAUN-SPS solvent purification system in which solvent is passed through an activated alumina column under nitrogen, or by standing over 3 Å molecular sieves under an atmosphere of nitrogen. Reagents were used as obtained without further purification unless stated otherwise. Chromatography: Thin layer chromatography (TLC) was carried out using Merck aluminium backed DC60 F254 plates (particle size 0.2 mm). TLC sheets were visualised by UV light, then developed by staining with potassium permanganate, anisaldehyde, cerium ammonium molybdate, vanillin, or iodine on silica. Purification by flash column chromatography[1] was carried out using Merck silicagel 60 F254 (particle size 43–60 μm).

Characterisation: Proton (^1H) and carbon (^{13}C) spectra were recorded on Bruker AVG400 (400/101 MHz), Bruker AVH400 (400/101 MHz), Bruker AVF400 (400/101 MHz), Bruker AVB500 (500/126 MHz), Bruker AVC500 (500/126 MHz), and Bruker DPX200 (200 MHz) NMR spectrometers. Spectra are referenced to the residual solvent peak. Chemical shifts (δ) are given in parts per million (ppm, ± 0.01) and coupling constants (*J*) are given in Hertz (Hz, ± 0.1 as measured on Mestrenova, without rounding). The following convention is used to report chemical shifts: δ (multiplicity, coupling constant(s), number of protons), with chemical shifts reported in descending order. When a large roof effect was observed, it was included after the multiplicity. Peak multiplicities are described as singlet (s), doublet (d), triplet (t), quartet (q), pentet (p), heptet (h), nonuplet (n), a combination e.g. doublet of doublets (dd), or as a multiplet (m) over a peak range. Additionally, peaks may be described as broad (br), or apparent (app).

Infrared spectra were recorded using a Bruker Tensor 27 FT-IR spectrometer. Selected diagnostic absorption maxima (νmax) are reported in wavenumbers (cm^{-1}). Low-resolution mass spectra were acquired using a Micromass LCT Premier spectrometer (ESI). High resolution mass spectra were recorded by Chemistry Research Laboratory staff using a Bruker Daltronics MicroTOF spectrometer (ESI). Mass to charge ratios (m/z) are reported in Daltons. Melting points were recorded using a Leica Galen III hot-stage microscope apparatus and are reported uncorrected in degrees Celcius (°C). Optical rotations were recorded

using a Perkin Elmer 241 optical activity polarimeter at 25 °C. The enantiomeric excesses were determined by HPLC analysis on an Agilent 1200 Series instrument employing a chiral stationary phase column specified in the individual experiment and by comparing the samples with the appropriate racemic mixtures. Optical rotations were recorded using a Perkin Elmer 341 polarimeter; $[\alpha]_D^T$ values are reported in $10^{-1}deg{\cdot}cm^2g^{-1}$; concentrations (c) are quoted in g/100 mL; D refers to the D-line of sodium (589 nm); temperatures (T) are given in degrees Celsius (°C). (+) and (–) compound number prefixes indicate the sign of the optical rotation.

Starting materials: Carboxylic acids were obtained from commercial chemical suppliers and used as received or synthesised according to literature procedures. Unless otherwise stated, amides were synthesised according to our previous literature reports, or obtained from commercial chemical suppliers, and where novel full characterisation is given.[1-4] (*N*-isocyanimino) triphenylphosphorane (NIITP) was synthesised according to Bio's method.[5]

2. General procedures

General Procedure A: Reductive synthesis of α-amino heterodiazoles

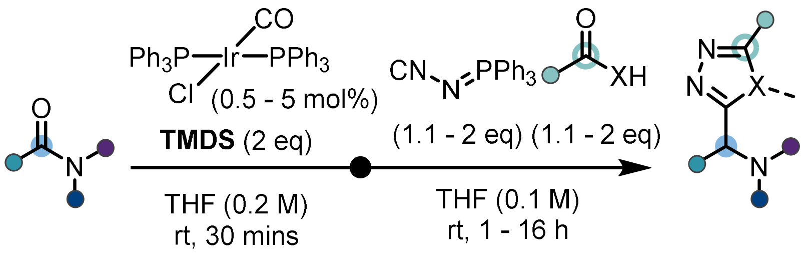

To an oven dried screw cap vial equipped with magnetic stirrer amide (0.2 mmol, 1 eq), and $IrCl(CO)(PPh_3)_2$ (Vaska's complex, 0.5 – 5 mol%) were added. The vial was evacuated under vacuum and backfilled with nitrogen (x3). Anhydrous THF (1 mL) was added followed in quick succession by TMDS (71 μL, 0.4 mmol, 2 eq), and the vial capped and stirred at rt. After 30 mins, the vial was opened and a suspension of (*N*-isocyanimino) triphenylphosphorane (NIITP, 1.1 – 2 eq) in THF (1 mL) added, followed by the appropriate *O*-, *C*-, *S*-, or *N*-Brønsted acid (1.1 – 2 eq) as a single portion. The vial was recapped and stirred at rt for 1 - 16 h. After this time CH_2Cl_2 (10 mL) and the reaction washed with saturated aqueous $NaHCO_3$ (20 mL), then the aqueous extracted with CH_2Cl_2 (3 x 10 mL). The combined organics were dried over $MgSO_4$ and then concentrated *in vacuo* to afford the crude product. The crude was then purified by flash column chromatography (FCC), and subsequent preparative thin layer chromatography (PTLC) when required, to afford the pure α-amino heterodiazole products.

3. Synthesis and characterization of starting materials

2-((1-(tert-butoxycarbonyl)piperidin-4-yl)oxy)-2-oxoacetic acid - **S1**

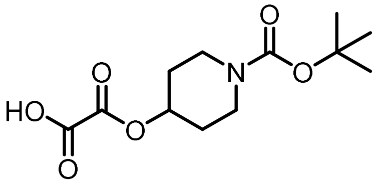

S1

Synthesised according to a literature procedure.[6]

^{1}H NMR (400 MHz, $CDCl_3$) δ 11.05 (s, 1H), 5.09 (tt, *J* = 7.8, 3.7, 1H), 3.73 (ddd, *J* = 13.6, 7.0, 4.0, 2H), 3.30 (ddd, *J* = 13.7, 8.3, 3.7, 2H), 1.93 (ddt, *J* = 14.2, 7.4, 3.8, 2H), 1.82 – 1.70 (m, 2H), 1.45 (s, 9H).

^{13}C NMR (101 MHz, $CDCl_3$) δ 158.3, 157.8, 155.4, 81.1, 73.1, 40.8, 30.1, 28.4.

N-tosylbenzamide – S2

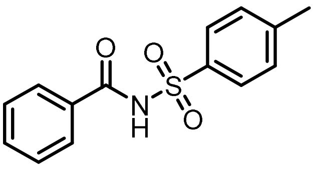

S2

Synthesised according to a literature procedure.[7]

^{1}H NMR (400 MHz, $CDCl_3$) δ 9.55 (s, 1H), 8.10 – 8.04 (m, 2H), 7.89 – 7.83 (m, 2H), 7.61 – 7.52 (m, 1H), 7.46 – 7.40 (m, 2H), 7.39 – 7.34 (m, 2H), 2.45 (s, 3H).

^{13}C NMR (101 MHz, $CDCl_3$) δ 164.5, 145.3, 135.5, 133.5, 131.2, 129.7, 128.9, 128.7, 127.9, 21.7.

tert-butyl benzoylcarbamate – S3

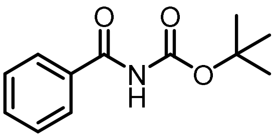

S3

Synthesised according to a literature procedure.[8]

^{1}H NMR (400 MHz, $CDCl_3$) δ 8.02 (s, 1H), 7.85 – 7.75 (m, 2H), 7.58 – 7.51 (m, 1H), 7.50 – 7.41 (m, 2H), 1.53 (s, 9H).

^{13}C NMR (101 MHz, $CDCl_3$) δ 165.3, 149.7, 133.5, 132.7, 128.8, 127.5, 82.8, 28.0.

diethyl benzoylphosphoramidate – 26

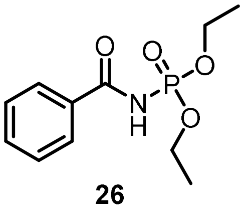

26

Synthesised according to a literature procedure.[9]

^{1}H NMR (400 MHz, $CDCl_3$) δ 9.41 (s, 1H), 8.07 – 7.99 (m, 2H), 7.53 – 7.44 (m, 1H), 7.42 – 7.33 (m, 2H), 4.29 – 4.08 (m, 4H), 1.29 (td, *J* = 7.1, 1.0, 6H).

^{31}P NMR (162 MHz, $CDCl_3$) δ -1.6.

^{13}C NMR (101 MHz, $CDCl_3$) δ 168.0 (d, *J* = 2.8), 132.7, 132.6 (d, *J* = 10.6), 128.5, 128.4, 64.2 (d, *J* = 5.9), 16.1 (d, *J* = 6.8).

1-allylazocan-2-one – S4

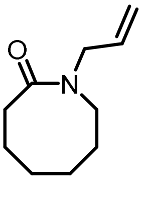

S4

Synthesised according to a literature procedure.[10]

^{1}H NMR (400 MHz, $CDCl_3$) δ 5.66 (ddt, *J* = 17.2, 10.1, 6.1, 1H), 5.07 – 4.97 (m, 2H), 3.85 (dt, *J* = 6.2, 1.5, 2H), 3.36 – 3.30 (m, 2H), 2.43 – 2.35 (m, 2H), 1.67 (ddd, *J* = 9.5, 7.4, 4.4, 2H), 1.51 (m, 2H), 1.45 – 1.39 (m, 2H), 1.39 – 1.30 (m, 2H).

^{13}C NMR (101 MHz, $CDCl_3$) δ 174.5, 133.7, 117.0, 47.3, 46.1, 33.7, 28.9, 28.7, 26.1, 24.3.

tert-butyl 4-(2-iodobenzoyl)piperazine-1-carboxylate– S5

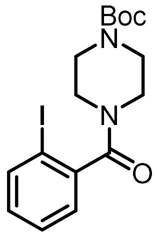

S5

White crystals – m.p.: 94 – 96 °C

^{1}H NMR (400 MHz, CDCl$_3$) δ 7.79 (dd, *J* = 8.0, 1.1, 1H), 7.36 (td, *J* = 7.5, 1.1, 1H), 7.15 (dd, *J* = 7.6, 1.6, 1H), 7.05 (td, *J* = 7.7, 1.7, 1H), 3.86 – 3.75 (m, 1H), 3.74 – 3.63 (m, 1H), 3.59 – 3.44 (m, 3H), 3.38 – 3.27 (m, 1H), 3.26 – 3.16 (m, 1H), 3.15 – 3.05 (m, 1H), 1.42 (s, 9H).

^{13}C NMR (101 MHz, CDCl$_3$) δ 169.4, 154.4, 141.9, 139.3, 130.4, 128.5, 127.0, 92.4, 80.3, 46.7, 43.5, 41.5, 28.4.

FT-IR(thin film): ν_{max}(cm^{-1}) = 2972, 2929, 2869, 1676, 1638, 1585, 1473, 1457, 1422, 1364, 1285, 1267, 1246, 1160, 1130, 1069, 1001, 949, 863, 761, 728, 655, 631.

(ESI): m/z calculated for $C_{16}H_{22}O_3N_2I$ requires 417.0670 for [M+H]$^+$, found 417.0669.

N,N-dimethyl-9-oxo-9H-fluorene-4-carboxamide – S6

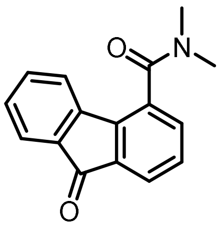

S6

Yellow solid – m.p.: 154 – 156 °C

^{1}H NMR (400 MHz, CDCl$_3$) δ 7.71 – 7.65 (m, 2H), 7.46 (td, *J* = 7.5, 1.3, 1H), 7.40 – 7.33 (m, 3H), 7.31 (td, *J* = 7.4, 1.1, 1H), 3.24 (s, 3H), 2.92 (s, 3H).

^{13}C NMR (101 MHz, CDCl$_3$) δ 192.9, 169.3, 142.9, 140.1, 135.2, 134.7, 134.1, 132.3, 131.1, 129.5, 129.4, 124.6, 124.4, 122.2, 38.5, 34.9.

FT-IR(thin film): $\nu_{max}(cm^{-1})$ = 2928, 1712, 1625, 1604, 1579, 1505, 1474, 1448, 1419, 1396, 1301, 1268, 1246, 1164, 1108, 970, 909, 828, 776, 733, 697, 654.

(ESI): m/z calculated for $C_{16}H_{14}O_2N$ requires 252.1019 for $[M+H]^+$, found 252.109.

4-(N,N-dipropylsulfamoyl)-N-tosylbenzamide - 56

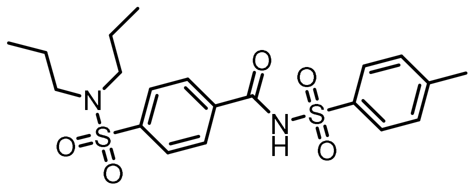

56

In an oven-dried round-bottom flask probenecid (571 mg, 2 mmol, 1 eq) was suspended in CH_2Cl_2 (4 mL), then oxalyl chloride (340 uL, 4 mmol, 2 eq) and DMF (3 drops) were added. After stirring at rt for 1 h the reaction was concentrated *in vacuo*, and the residue uptaken into a mixture of toluene:ethyl acetate (2.5 mL:1 mL). To reaction was added triethylamine (560 uL, 4 mmol, 2 eq), DMAP (12.2 mg, 0.1 mmol, 5 mol%), and *p*-Toluenesulfonamide (377 mg, 2.2 mmol, 1.1 eq), then the reaction was heated to 55 °C for 2 h. The reaction was quenched by addition of 2M hydrochloric acid (50 mL), extracted with EtOAc (3 x 25 mL), and the combined organics dried over $MgSO_4$ and concentrated *in vacuo* to afford the crude product. The crude was purified by flash column chromatography (EtOAc), and the product fractions concentrated *in vacuo* then washed with 2M hydrochloric acid (50 mL), extracted with CH_2Cl_2 (3 x 25 mL), and the combined organics dried over $MgSO_4$, and concentrated *in vacuo* to afford the *title compound* (814.3 mg, 1.86 mmol, 93%) as a white powder.

m.p.: 138 – 140 °C

^{1}H NMR (400 MHz, $CDCl_3$) δ 9.58 (s, 1H), 8.03 (d, *J* = 8.4, 2H), 7.93 (d, *J* = 8.5, 2H), 7.80 (d, *J* = 8.5, 2H), 7.39 – 7.34 (m, 2H), 3.09 – 3.02 (m, 4H), 2.44 (s, 3H), 1.57 – 1.45 (m, 4H), 0.84 (t, *J* = 7.4, 6H).

^{13}C NMR (101 MHz, $CDCl_3$) δ 163.3, 145.6, 144.6, 135.2, 134.6, 129.7, 128.7, 127.4, 49.9, 21.9, 21.7, 11.1.

FT-IR(thin film): $\nu_{max}(cm^{-1})$ = 3238, 2965, 2922, 1700, 1636, 1436, 1344, 1324, 1298, 1184, 1151, 1116, 1073, 991, 889, 852, 829, 754, 662.

(ESI): m/z calculated for $C_{20}H_{27}O_5N_2S_2$ requires 439.1356 for $[M+H]^+$, found 439.1360.

4-(N,N-dipropylsulfamoyl)benzothioic S-acid - 57

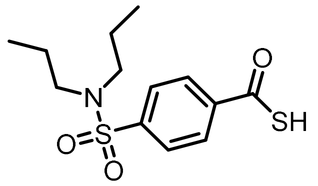

57

In an oven-dried round-bottom flask probenecid (571 mg, 2 mmol, 1 eq) was suspended in CH_2Cl_2 (4 mL), then oxalyl chloride (340 uL, 4 mmol, 2 eq) and DMF (3 drops) were added. After stirring at rt for 1 h the reaction was concentrated *in vacuo*, then uptaken into THF (5 mL). In a separate rbf a suspension of sodium hydrosulfide hydrate (673 mg, 12 mmol, 6 eq) in THF (5 mL) was cooled to 0 °C, then the above THF solution of acid chloride added dropwise over 5 mins. The reaction was stirred at 0 °C for 1 h, then warmed to rt and stirred for a further 2 h. The reaction was quenched by addition of 2M hydrochloric acid (50 mL), extracted with EtOAc (3 x 25 mL), and the combined organics dried over $MgSO_4$ and concentrated *in vacuo* to afford the *title compound* (660 mg, >2.00 mmol, quant.) as a yellow oil which was used without further purification

^{1}H NMR (400 MHz, $CDCl_3$) δ 8.00 (d, *J* = 8.5, 2H), 7.89 (d, *J* = 8.5, 2H), 4.83 (s, 1H), 3.14 – 3.05 (m, 4H), 1.61 – 1.48 (m, 4H), 0.86 (t, *J* = 7.4, 6H).

^{13}C NMR (101 MHz, $CDCl_3$) δ 189.1, 145.1, 139.2, 128.4, 127.4, 50.0, 22.0, 11.1.

FT-IR(thin film): ν_{max}(cm^{-1}) = 3656, 2979, 2880, 1695, 1466, 1396, 1341, 1291, 1265, 1198, 1178, 1156, 1088, 993, 888, 844, 797, 775, 732, 702, 609.

(ESI): m/z calculated for $C_{13}H_{20}O_3N_1S_2$ requires 302.0879 for [M+H]$^+$, found 302.0881.

4. Synthesis and characterization of products

2-(1-benzylpiperidin-2-yl)-5-phenyl-1,3,4-oxadiazole - 2

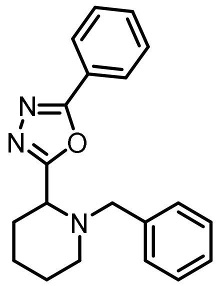

2

Following **general procedure A** (using 0.5 mol% Vaska's complex, and stirring for 1 h after addition of 1.1 eq of acid and NIITP): after FCC (30% Et_2O/Pentane), **2** (55.8 mg, 0.174 mmol, 87%) was afforded as a colourless oil.

^{1}H NMR (400 MHz, $CDCl_3$) δ 8.05 – 8.00 (m, 2H), 7.53 – 7.43 (m, 3H), 7.30 – 7.20 (m, 4H), 7.14 (d, *J* = 7.1, 1H), 3.97 (dd, *J* = 7.3, 4.7, 1H), 3.63 (d, *J* = 13.6, 1H), 3.42 (d, *J* = 13.7, 1H), 2.95 (dt, *J* = 11.7, 5.1, 1H), 2.33 – 2.23 (m, 1H), 2.03 – 1.90 (m, 2H), 1.86 – 1.73 (m, 1H), 1.69 – 1.59 (m, 2H), 1.52 – 1.42 (m, 1H).

^{13}C NMR (101 MHz, $CDCl_3$) δ 167.3, 164.8, 138.1, 131.6, 129.0, 128.9, 128.2, 127.0, 127.0, 124.0, 60.4, 57.5, 51.1, 30.6, 25.4, 22.4.

FT-IR(thin film): ν_{max}(cm^{-1}) = 2980, 2889, 1533, 1473, 1450, 1251, 1069, 1028, 1007, 957, 732, 690.

(ESI): m/z calculated for $C_{20}H_{22}ON_3$ requires 320.1757 for $[M+H]^+$, found 320.1754.

2-(1-benzylpiperidin-2-yl)-5-methyl-1,3,4-oxadiazole - 4

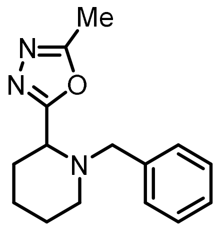

4

Following **general procedure A** (using 0.5 mol% Vaska's complex, and stirring for 1 h after addition of 1.1 eq of acid and NIITP): after FCC (60% Et_2O/Pentane), **4** (44.0 mg, 0.170 mmol, 85%) was afforded as a tan semi-solid.

m.p.: 46 – 48 °C

^{1}H NMR (400 MHz, $CDCl_3$) δ 7.18 – 7.02 (m, 5H), 3.69 (t, *J* = 6.2, 1H), 3.43 (d, *J* = 13.7, 1H), 3.23 (d, *J* = 13.7, 1H), 2.79 (dt, *J* = 11.6, 4.6, 1H), 2.32 (s, 3H), 2.15 – 2.04 (m, 1H), 1.79 – 1.71 (m, 2H), 1.69 – 1.56 (m, 1H), 1.54 – 1.43 (m, 2H), 1.33 – 1.20 (m, 1H).

^{13}C NMR (101 MHz, $CDCl_3$) δ 167.5, 163.8, 137.9, 129.0, 128.2, 127.0, 60.4, 57.7, 51.5, 30.6, 25.3, 22.6, 11.0.

FT-IR(thin film): $\nu_{max}(cm^{-1})$ = 2980, 2888, 1714, 1591, 1472, 1383, 1251, 1154, 1071, 955, 735, 698.

(ESI): m/z calculated for $C_{15}H_{20}ON_3$ requires 258.1601 for $[M+H]^+$, found 258.1601.

2-(1-benzylpiperidin-2-yl)-5-(1-phenylcyclopropyl)-1,3,4-oxadiazole - **5**

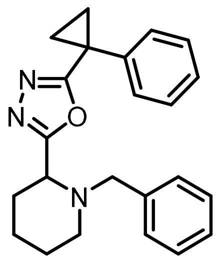

5

Following **general procedure A** (using 0.5 mol% Vaska's complex, and stirring for 1 h after addition of 1.1 eq of acid and NIITP): after FCC (30% Et_2O/Pentane), **5** (57.9 mg, 0.162 mmol, 81%) was afforded as a yellow oil.

^{1}H NMR (400 MHz, $CDCl_3$) δ 7.29 – 7.23 (m, 2H), 7.22 – 7.14 (m, 3H), 7.14 – 7.05 (m, 3H), 7.03 (dd, *J* = 7.8, 1.8, 2H), 3.68 (dd, *J* = 7.1, 4.9, 1H), 3.37 (d, *J* = 13.6, 1H), 3.15 (d, *J* = 13.6, 1H), 2.69 (dt, *J* = 11.6, 4.9, 1H), 2.09 (dt, *J* = 11.9, 6.4, 1H), 1.75 – 1.66 (m, 2H), 1.66 – 1.56 (m, 1H), 1.55 – 1.47 (m, 2H), 1.44 (m, 2H), 1.33 – 1.23 (m, 3H).

^{13}C NMR (101 MHz, $CDCl_3$) δ 169.7, 167.2, 138.8, 138.3, 129.5, 128.9, 128.6, 128.2, 127.7, 127.0, 60.2, 57.3, 51.1, 30.4, 25.3, 22.4, 22.3, 16.1, 16.1.

FT-IR(thin film): $\nu_{max}(cm^{-1})$ = 2980, 2888, 1601, 1557, 1472, 1382, 1151, 1069, 1027, 965, 836, 737, 697, 636.

(ESI): m/z calculated for $C_{23}H_{26}ON_3$ requires 360.2070 for $[M+H]^+$, found 360.2067.

tert-butyl ((5-(1-benzylpiperidin-2-yl)-1,3,4-oxadiazol-2-yl)methyl)carbamate - 6

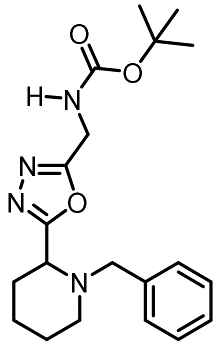

6

Following **general procedure A** (using 0.5 mol% Vaska's complex, and stirring for 1 h after addition of 1.1 eq of acid and NIITP): after FCC (60% Et_2O/Pentane), **6** (61.5 mg, 0.166 mmol, 83%) was afforded as a tan powder.

m.p.: 80 – 82 °C

^{1}H NMR (400 MHz, $CDCl_3$) δ 7.18 – 7.03 (m, 5H), 5.01 (br, s, 1H), 4.39 – 4.26 (m, 2H), 3.74 (t, *J* = 6.1, 1H), 3.42 (d, *J* = 13.6, 1H), 3.27 (d, *J* = 13.7, 1H), 2.79 (dt, *J* = 11.6, 4.8, 1H), 2.16 (dt, *J* = 12.0, 6.3, 1H), 1.75 (td, *J* = 6.6, 4.7, 2H), 1.62 (dt, *J* = 13.1, 5.2, 1H), 1.54 – 1.45 (m, 2H), 1.31 (s, 10H).

^{13}C NMR (101 MHz, $CDCl_3$) δ 167.9, 163.9, 155.4, 138.0, 128.9, 128.1, 127.0, 80.5, 60.4, 57.5, 51.4, 35.9, 30.4, 28.3, 25.3, 22.3.

FT-IR(thin film): ν_{max}(cm^{-1}) = 2980, 1715, 1584, 1507, 1453, 1391, 1366, 1250, 1163, 970, 937, 735, 698.

(ESI): m/z calculated for $C_{20}H_{29}O_3N_4$ requires 373.2234 for $[M+H]^+$, found 373.2227.

2-(1-benzylpiperidin-2-yl)-5-ethynyl-1,3,4-oxadiazole - **7**

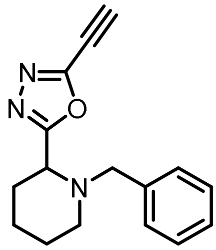

7

Following **general procedure A** (using 0.5 mol% Vaska's complex, and stirring for 1 h after addition of 1.1 eq of acid and NIITP): after FCC (20% Et_2O/Pentane), **7** (36.0 mg, 0.134 mmol, 67%) was afforded as a colourless oil.

^{1}H NMR (400 MHz, $CDCl_3$) δ 7.32 – 7.20 (m, 5H), 3.97 (t, *J* = 5.9, 1H), 3.59 (d, *J* = 13.6, 1H), 3.53 (s, 1H), 3.43 (d, *J* = 13.6, 1H), 2.92 (dt, *J* = 11.7, 5.1, 1H), 2.32 (dt, *J* = 11.8, 5.8, 1H), 1.99 – 1.85 (m, 2H), 1.75 (dq, *J* = 16.5, 5.5, 1H), 1.71 – 1.55 (m, 2H), 1.56 – 1.39 (m, 1H).

^{13}C NMR (101 MHz, $CDCl_3$) δ 168.0, 149.9, 137.8, 128.9, 128.3, 128.2, 127.2, 85.7, 67.6, 60.3, 57.3, 50.7, 30.4, 25.2, 22.0.

FT-IR(thin film): ν_{max}(cm^{-1}) = 2939, 2855, 2134, 1595, 1518, 1494, 1452, 1371, 1345, 1319, 1261, 1209, 1178, 1156, 1128, 1105, 1068, 1049, 1027, 988, 910, 881, 836, 802, 777, 735, 697.

(ESI): m/z calculated for $C_{16}H_{18}ON_3$ requires 268.1444 for $[M+H]^+$, found 268.1445.

2-(1-benzylpiperidin-2-yl)-5-(trifluoromethyl)-1,3,4-oxadiazole - **8**

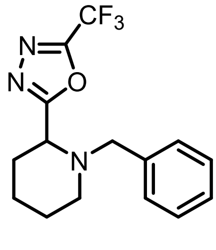

8

To an oven dried screw cap vial equipped with magnetic stirrer 1-benzylpiperidin-2-one (56.8 mg, 0.3 mmol, 3 eq), and Vaska's complex (1.2 mg, 0.0015 mmol, 0.5 mol% with respect to the amide) were added. The vial was evacuated under vacuum and backfilled with nitrogen (x3). Then anhydrous THF (1 mL) was added followed in quick succession by TMDS (108 µL, 0.6 mmol, 6 eq), and the vial capped and stirred at rt. After 30 mins, the vial was opened and (*N*-isocyanimino) triphenylphosphorane (90.9 mg, 0.3 mmol, 3 eq) added,

the vial was then sealed with a subaseal and a solution of trifluoroacetic acid (7.7 µL, 0.1 mmol, 1 eq) in THF (1 mL) over 30 mins. The vial stirred at rt for a further 1 h. After this time CH_2Cl_2 (10 mL) was added and the reaction washed with saturated aqueous $NaHCO_3$ (20 mL), then the aqueous was extracted with CH_2Cl_2 (3 x 10 mL). The combined organics were dried over $MgSO_4$and then concentrated *in vacuo* to afford the crude product: after FCC (10% Et_2O/Pentane), **8** (12.5 mg, 0.040 mmol, 40%) was afforded as a colourless oil.

^{1}H NMR (400 MHz, $CDCl_3$) δ 7.35 – 7.03 (m, 5H), 3.97 (dd, *J* = 6.7, 4.9, 1H), 3.52 (d, *J* = 13.7, 1H), 3.41 (d, *J* = 13.6, 1H), 2.87 (dt, *J* = 11.9, 5.3, 1H), 2.33 (dt, *J* = 11.7, 5.8, 1H), 1.94 – 1.83 (m, 2H), 1.76 – 1.64 (m, 1H), 1.64 – 1.56 (m, 2H), 1.50 – 1.40 (m, 1H).

^{19}F NMR (377 MHz, $CDCl_3$) δ -65.10

^{13}C NMR (101 MHz, $CDCl_3$) δ 169.5, 137.6, 128.7, 128.3, 127.3, 60.5, 57.1, 50.8, 30.2, 25.2, 21.8.

FT-IR(thin film): ν_{max}(cm^{-1}) = 2980, 1586, 1453, 1406, 1322, 1259, 1207, 1164, 1128, 1048, 909, 754, 736,. 698.

(ESI): m/z calculated for $C_{15}H_{17}ON_3F_3$ requires 312.1318 for $[M+H]^+$, found 312.1316.

2-(1-benzylpiperidin-2-yl)-5-(pyrazin-2-yl)-1,3,4-oxadiazole - **9**

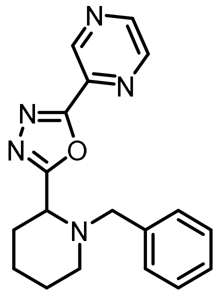

9

Following **general procedure A** (using 0.5 mol% Vaska's complex, and stirring for 1 h after addition of 1.1 eq of acid and NIITP): after FCC (65% Et_2O/Pentane), **9** (53.0 mg, 0.164 mmol, 82%) was afforded as a yellow oil.

^{1}H NMR (400 MHz, $CDCl_3$) δ 9.25 (d, *J* = 1.4, 1H), 8.59 – 8.55 (m, 2H), 7.15 – 7.10 (m, 2H), 7.10 – 7.04 (m, 2H), 7.01 – 6.95 (m, 1H), 3.91 (dd, *J* = 7.6, 4.2, 1H), 3.50 (d, *J* = 13.7, 1H), 3.34 (d, *J* = 13.7, 1H), 2.90 – 2.80 (m, 1H), 2.25 – 2.16 (m, 1H), 1.95 – 1.78 (m, 2H), 1.71 – 1.59 (m, 1H), 1.59 – 1.44 (m, 2H), 1.40 – 1.29 (m, 1H).

^{13}C NMR (101 MHz, $CDCl_3$) δ 168.8, 162.2, 146.4, 144.6, 144.2, 139.8, 138.1, 128.8, 128.1, 127.0, 60.5, 57.5, 51.1, 30.5, 25.3, 22.2.

FT-IR(thin film): $\nu_{max}(cm^{-1})$ = 2981, 2889, 1568, 1454, 1420, 1382, 1251, 1161, 1106, 1016, 965, 856, 734, 698.

(ESI): m/z calculated for $C_{18}H_{20}ON_5$ requires 322.1662 for $[M+H]^+$, found 322.1663.

2-(1-benzylpiperidin-2-yl)-5-(5-bromopyridin-3-yl)-1,3,4-oxadiazole - **10**

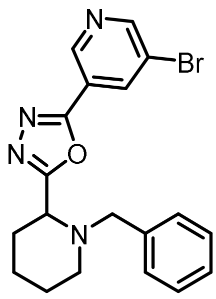

10

Following **general procedure A** (using 0.5 mol% Vaska's complex, and stirring for 1 h after addition of 1.1 eq of acid and NIITP): after FCC (30% Et_2O/Pentane), **10** (67.1 mg, 0.168 mmol, 84%) was afforded as a brown powder.

m.p.: 96 – 98 °C

^1H NMR (400 MHz, $CDCl_3$) δ 9.06 (d, *J* = 1.8, 1H), 8.74 (d, *J* = 2.2, 1H), 8.34 (t, *J* = 2.1, 1H), 7.21 – 7.13 (m, 4H), 7.10 – 7.04 (m, 1H), 3.93 (t, *J* = 6.1, 1H), 3.55 (d, *J* = 13.7, 1H), 3.41 (d, *J* = 13.8, 1H), 2.93 (dt, *J* = 11.7, 4.9, 1H), 2.30 (ddd, *J* = 12.0, 7.3, 5.3, 1H), 1.94 – 1.86 (m, 2H), 1.79 – 1.70 (m, 1H), 1.67 – 1.58 (m, 2H), 1.49 – 1.37 (m, 1H).

^{13}C NMR (101 MHz, $CDCl_3$) δ 168.2, 161.5, 153.3, 145.8, 137.9, 136.4, 128.8, 128.2, 127.0, 121.7, 120.9, 60.6, 57.7, 51.7, 30.6, 25.3, 22.4.

FT-IR(thin film): $\nu_{max}(cm^{-1})$ = 2980, 2889, 1600, 1537, 1472, 1451, 1382, 1302, 1263, 1153, 1099, 1078, 969, 890, 784, 733, 694.

(ESI): m/z calculated for $C_{19}H_{20}ON_4{}^{79}Br$ requires 399.0815 for $[M+H]^+$, found 399.0816.

2-(1-benzylpiperidin-2-yl)-5-(1H-indazol-7-yl)-1,3,4-oxadiazole - **11**

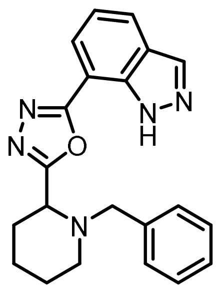

11

Following **general procedure A** (using 0.5 mol% Vaska's complex, and stirring for 1 h after addition of 1.1 eq of acid and NIITP): after FCC (60% Et_2O/Pentane), **11** (56.9 mg, 0.158 mmol, 79%) was afforded as a yellow oil.

^{1}H NMR (400 MHz, $CDCl_3$) δ 11.48 (br, s, 1H), 8.02 (s, 1H), 7.81 – 7.76 (m, 2H), 7.17 – 7.11 (m, 3H), 7.10 – 7.05 (m, 2H), 7.01 – 6.95 (m, 1H), 3.89 (dd, *J* = 7.4, 4.6, 1H), 3.52 (d, *J* = 13.6, 1H), 3.33 (d, *J* = 13.7, 1H), 2.89 – 2.80 (m, 1H), 2.26 – 2.17 (m, 1H), 1.93 – 1.80 (m, 2H), 1.74 – 1.62 (m, 1H), 1.58 – 1.47 (m, 2H), 1.43 – 1.30 (m, 1H).

^{13}C NMR (101 MHz, $CDCl_3$) δ 166.8, 163.3, 137.9, 136.7, 135.3, 128.9, 128.2, 128.2, 127.1, 127.0, 125.1, 125.0, 124.1, 120.7, 106.4, 60.5, 57.4, 51.1, 30.6, 25.3, 22.3.

FT-IR(thin film): ν_{max}(cm^{-1}) = 3201, 2980, 1609, 1550, 1495, 1452, 1319, 1198, 1068, 946, 839, 734, 698.

(ESI): m/z calculated for $C_{21}H_{22}ON_5$ requires 360.1819 for $[M+H]^+$, found 360.1817.

2-((5-(1-benzylpiperidin-2-yl)-1,3,4-oxadiazol-2-yl)methoxy)benzaldehyde - **12**

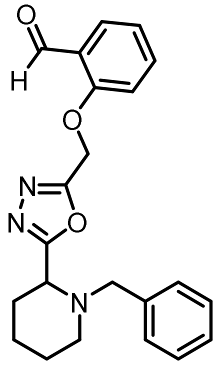

12

Following **general procedure A** (using 0.5 mol% Vaska's complex, and stirring for 1 h after addition of 1.1 eq of acid and NIITP): after FCC (55% Et_2O/Pentane), **12** (51.1 mg, 0.136 mmol, 68%) was afforded as a white crystalline solid.

m.p.: 110 – 112 °C

^{1}H NMR (400 MHz, $CDCl_3$) δ 10.40 (d, *J* = 0.8, 1H), 7.78 (dd, *J* = 7.6, 1.8, 1H), 7.47 (ddd, *J* = 8.4, 7.3, 1.8, 1H), 7.21 – 7.09 (m, 5H), 7.07 – 7.00 (m, 2H), 5.36 – 5.18 (m, 2H), 3.87 (dd, *J* = 6.7, 5.3, 1H), 3.46 (d, *J* = 13.7, 1H), 3.31 (d, *J* = 13.6, 1H), 2.83 (dt, *J* = 11.6, 4.9, 1H), 2.23 (dt, *J* = 11.9, 6.3, 1H), 1.89 – 1.79 (m, 2H), 1.74 – 1.63 (m, 1H), 1.60 – 1.52 (m, 2H), 1.44 – 1.32 (m, 1H).

^{13}C NMR (101 MHz, $CDCl_3$) δ 188.9, 168.8, 161.8, 159.6, 137.9, 135.9, 128.8, 128.8, 128.2, 127.0, 125.5, 122.4, 112.9, 60.3, 57.4, 51.1, 30.4, 25.2, 22.2.

FT-IR(thin film): ν_{max}(cm^{-1}) = 2980, 2886, 1688, 1599, 1483, 1456, 1392, 1286, 1238, 1162, 1104, 1019, 855, 833, 759, 741, 696.

(ESI): m/z calculated for $C_{22}H_{24}O_3N_3$ requires 378.1812 for $[M+H]^+$, found 378.1810.

4-(5-(1-benzylpiperidin-2-yl)-1,3,4-oxadiazol-2-yl)benzenesulfonyl fluoride - 13

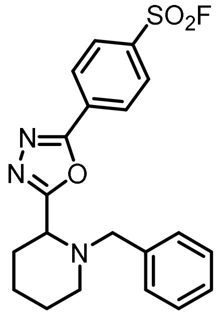

13

Following **general procedure A** (using 0.5 mol% Vaska's complex, and stirring for 16 h after addition of 2 eq of acid and NIITP): after FCC (40% Et_2O/Pentane), **13** (32.0 mg, 0.080 mmol, 40%) was afforded as an amber oil.

^{1}H NMR (400 MHz, $CDCl_3$) δ 8.24 – 8.18 (m, 2H), 8.10 – 8.05 (m, 2H), 7.23 – 7.13 (m, 4H), 7.10 – 7.04 (m, 1H), 3.95 (t, *J* = 6.1, 1H), 3.56 (d, *J* = 13.7, 1H), 3.41 (d, *J* = 13.7, 1H), 2.94 (dt, *J* = 11.7, 4.8, 1H), 2.30 (dt, *J* = 12.1, 6.2, 1H), 1.96 – 1.87 (m, 2H), 1.76 (dt, *J* = 13.2, 5.2, 1H), 1.67 – 1.58 (m, 2H), 1.44 (dt, *J* = 13.4, 6.7, 1H).

^{19}F NMR (377 MHz, $CDCl_3$) δ 66.1.

^{13}C NMR (101 MHz, $CDCl_3$) δ 168.6, 162.9, 137.9, 135.4 (d, *J* = 25.7), 130.4, 129.2, 128.8, 128.2, 127.9, 127.1, 60.6, 57.7, 51.6, 30.7, 25.3, 22.4.

FT-IR(thin film): ν_{max}(cm^{-1}) = 2980, 1607, 1550, 1485, 1453, 1415, 1293, 1213, 1171, 1081, 1009, 964, 846, 787, 737, 698, 616.

(ESI): m/z calculated for $C_{20}H_{21}O_3N_3FS$ requires 402.1281 for $[M+H]^+$, found 402.1283.

2-(1-benzylpiperidin-2-yl)-5-(3,3,3-trifluoroprop-1-en-2-yl)-1,3,4-oxadiazole - **14**

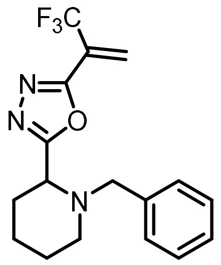

14

Following **general procedure A** (using 0.5 mol% Vaska's complex, and stirring for 1 h after addition of 1.1 eq of acid and NIITP): after FCC (15% Et_2O/Pentane), **14** (29.3 mg, 0.086 mmol, 43%) was afforded as a yellow oil.

^{1}H NMR (400 MHz, $CDCl_3$) δ 7.20 – 7.16 (m, 4H), 7.16 – 7.10 (m, 1H), 6.51 – 6.48 (m, 1H), 6.30 – 6.27 (m, 1H), 3.92 (dd, *J* = 6.9, 5.0, 1H), 3.52 (d, *J* = 13.7, 1H), 3.38 (d, *J* = 13.7, 1H), 2.88 (dt, *J* = 11.7, 5.0, 1H), 2.29 (dt, *J* = 11.9, 6.1, 1H), 1.91 – 1.83 (m, 2H), 1.77 – 1.66 (m, 1H), 1.62 – 1.53 (m, 2H), 1.49 – 1.37 (m, 1H).

^{19}F NMR (377 MHz, $CDCl_3$) δ -66.09.

^{13}C NMR (101 MHz, $CDCl_3$) δ 167.9, 159.2, 138.0, 128.8, 128.2, 127.1, 126.9 (q, *J* = 4.9), 121.0 (q, *J* = 273.2), 60.5, 57.3, 51.2, 30.5, 25.3, 22.1.

FT-IR(thin film): ν_{max}(cm^{-1}) = 2980, 2888, 1538, 1454, 1382, 1304, 1253, 1147, 1084, 965, 737, 698.

(ESI): m/z calculated for $C_{17}H_{219}ON_3F_3$ requires 338.1475 for $[M+H]^+$, found 338.1472.

(E)-2-(1-benzylpiperidin-2-yl)-5-(3-bromoprop-1-en-1-yl)-1,3,4-oxadiazole - **15**

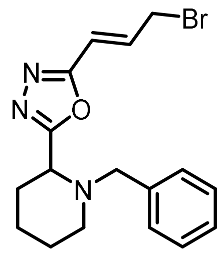

15

Following **general procedure A** (using 0.5 mol% Vaska's complex, and stirring for 1 h after addition of 2 eq of acid and 1.1 eq of NIITP): after FCC (40% Et_2O/Pentane), **15** (45.2 mg, 0.124 mmol, 62%) was afforded as a tan powder.

m.p.: 136 – 138 °C

^{1}H NMR (400 MHz, $CDCl_3$) δ 7.15 – 7.08 (m, 4H), 7.08 – 7.02 (m, 1H), 6.70 (dt, *J* = 15.8, 7.5, 1H), 6.45 (dt, *J* = 15.8, 1.2, 1H), 3.95 (dd, *J* = 7.5, 1.2, 2H), 3.76 (t, *J* = 6.1, 1H), 3.44 (d, *J* = 13.6, 1H), 3.26 (d, *J* = 13.7, 1H), 2.80 (dt, *J* = 11.7, 4.8, 1H), 2.15 (dt, *J* = 12.0, 6.2, 1H), 1.81 – 1.73 (m, 2H), 1.68 – 1.58 (m, 1H), 1.54 – 1.44 (m, 2H), 1.36 – 1.24 (m, 1H).

^{13}C NMR (101 MHz, $CDCl_3$) δ 167.2, 162.7, 137.9, 136.1, 128.9, 128.2, 127.1, 116.3, 60.4, 57.5, 51.3, 30.6, 29.9, 25.3, 22.4.

FT-IR(thin film): ν_{max}(cm^{-1}) = 2980, 2888, 1529, 1472, 1459, 1382, 1251, 1153, 1072, 955, 814, 732, 701.

(ESI): m/z calculated for $C_{17}H_{21}ON_3{}^{79}Br$ requires 362.0863 for $[M+H]^+$, found 362.0859.

2-(1-benzylpiperidin-2-yl)-5-(bromomethyl)-1,3,4-oxadiazole - 16

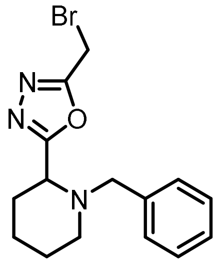

16

Following **general procedure A** (using 0.5 mol% Vaska's complex, and stirring for 1 h after addition of 1.1 eq of acid and NIITP): after FCC (30% Et_2O/Pentane), **16** (32.4 mg, 0.096 mmol, 48%) was afforded as a light yellow semi-solid.

m.p.: 118 – 120 °C

^{1}H NMR (400 MHz, $CDCl_3$) δ 7.16 – 7.10 (m, 4H), 7.10 – 7.04 (m, 1H), 4.38 – 4.25 (m, 2H), 3.78 (t, *J* = 6.0, 1H), 3.44 (d, *J* = 13.6, 1H), 3.26 (d, *J* = 13.6, 1H), 2.78 (dt, *J* = 11.9, 4.9, 1H), 2.17 (dt, *J* = 11.9, 6.1, 1H), 1.82 – 1.74 (m, 2H), 1.69 – 1.58 (m, 1H), 1.55 – 1.44 (m, 2H), 1.38 – 1.28 (m, 1H).

^{13}C NMR (101 MHz, $CDCl_3$) δ 168.7, 162.6, 137.9, 129.0, 128.2, 127.1, 60.4, 57.4, 51.0, 30.4, 25.2, 22.2, 16.5.

FT-IR(thin film): ν_{max}(cm^{-1}) = 2980, 1699, 1542, 1492, 1453, 1264, 1161, 1034, 731, 700.

(ESI): m/z calculated for $C_{15}H_{19}ON_3{}^{79}Br$ requires 336.0706 for $[M+H]^+$, found 336.0704.

1-(5-(1-benzylpiperidin-2-yl)-1,3,4-oxadiazol-2-yl)ethan-1-one - **17**

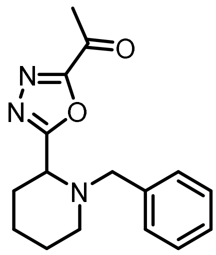

17

Following **general procedure A** (using 0.5 mol% Vaska's complex, and stirring for 1 h after addition of 1.1 eq of acid and NIITP): after FCC (20% Et_2O/Pentane), **17** (27.1 mg, 0.096 mmol, 48%) was afforded as a yellow oil.

^{1}H NMR (400 MHz, $CDCl_3$) δ 7.18 – 7.13 (m, 4H), 7.13 – 7.06 (m, 1H), 3.91 (dd, *J* = 6.9, 4.8, 1H), 3.48 (d, *J* = 13.7, 1H), 3.37 (d, *J* = 13.7, 1H), 2.85 (ddd, *J* = 12.0, 5.9, 4.4, 1H), 2.62 (s, 3H), 2.27 (ddd, *J* = 11.7, 6.9, 4.9, 1H), 1.91 – 1.80 (m, 2H), 1.71 – 1.60 (m, 1H), 1.59 – 1.50 (m, 2H), 1.44 – 1.34 (m, 1H).

^{13}C NMR (101 MHz, $CDCl_3$) δ 184.3, 169.7, 161.3, 138.0, 128.8, 128.2, 127.0, 60.5, 57.4, 50.9, 30.3, 27.2, 25.2, 22.0.

FT-IR(thin film): ν_{max}(cm^{-1}) = 2981, 2889, 1715, 1493, 1382, 1252, 1152, 1071, 955, 835, 803, 733, 698.

(ESI): m/z calculated for $C_{16}H_{20}O_2N_3$ requires 286.1550 for $[M+H]^+$, found 286.1551.

1-(tert-butoxycarbonyl)piperidin-4-yl 5-(1-benzylpiperidin-2-yl)-1,3,4-oxadiazole-2-carboxylate - 18

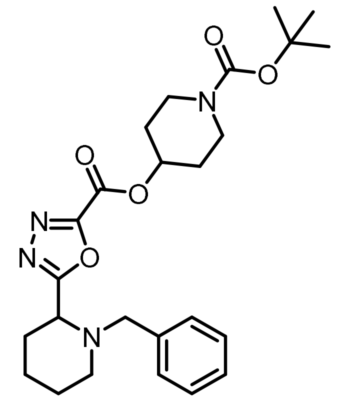

18

Following **general procedure A** (using 0.5 mol% Vaska's complex, and stirring for 16 h after addition of 2 eq of acid and NIITP): after FCC (50% Et_2O/Pentane), **18** (51.6 mg, 0.114 mmol, 57%) was afforded as a yellow oil.

^{1}H NMR (400 MHz, $CDCl_3$) δ 7.15 – 7.08 (m, 4H), 7.05 (dt, *J* = 8.7, 4.2, 1H), 5.11 (tt, *J* = 8.0, 3.9, 1H), 3.87 (dd, *J* = 6.7, 5.0, 1H), 3.72 – 3.59 (m, 2H), 3.43 (d, *J* = 13.7, 1H), 3.30 (d, *J* = 13.7, 1H), 3.20 – 3.08 (m, 2H), 2.84 – 2.76 (m, 1H), 2.20 (tt, *J* = 9.8, 4.4, 1H), 1.91 – 1.75 (m, 4H), 1.73 – 1.55 (m, 3H), 1.55 – 1.45 (m, 2H), 1.32 (s, 10H).

^{13}C NMR (101 MHz, $CDCl_3$) δ 169.7, 156.8, 154.6, 153.7, 137.9, 128.8, 128.2, 127.1, 79.9, 73.6, 60.5, 57.4, 51.0, 40.8, 30.4, 28.4, 25.2, 22.0.

FT-IR(thin film): ν_{max}(cm^{-1}) = 2935, 1745, 1691, 1539, 1453, 1422, 1365, 1320, 1274, 1238, 1165, 1135, 941, 770, 736, 699.

(ESI): m/z calculated for $C_{25}H_{35}O_5N_4$ requires 471.2602 for $[M+H]^+$, found 471.2599.

2-(1-benzylpiperidin-2-yl)-5-((2-methyl-4-(((4-methyl-2-(4-(trifluoromethyl)phenyl)thiazol-5-yl)methyl)thio)phenoxy)methyl)-1,3,4-oxadiazole - **19**

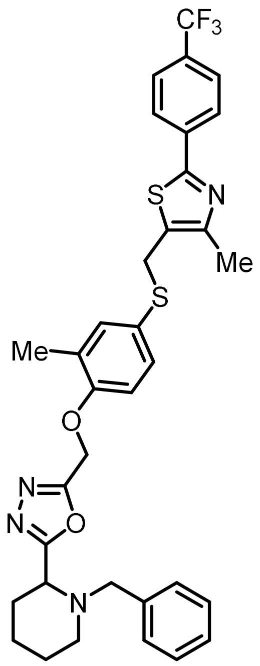

19

Following **general procedure A** (using 0.5 mol% Vaska's complex, and stirring for 1 h after addition of 1.1 eq of acid and NIITP): after FCC (45% Et_2O/Pentane), **19** (94.4 mg, 0.146 mmol, 73%) was afforded as a white powder.

m.p.: 116 – 118 °C

^1H NMR (400 MHz, $CDCl_3$) δ 7.96 – 7.89 (m, 2H), 7.60 (d, *J* = 8.2, 2H), 7.24 – 7.08 (m, 7H), 6.80 (d, *J* = 8.4, 1H), 5.21 – 5.10 (m, 2H), 4.05 (s, 2H), 3.90 (t, *J* = 6.0, 1H), 3.49 (d, *J* = 13.6, 1H), 3.33 (d, *J* = 13.6, 1H), 2.85 (dt, *J* = 11.7, 4.9, 1H), 2.29 – 2.20 (m, 1H), 2.17 (s, 3H), 2.13 (s, 3H), 1.90 – 1.84 (m, 2H), 1.76 – 1.67 (m, 1H), 1.63 – 1.54 (m, 2H), 1.47 – 1.36 (m, 1H).

^{19}F NMR (377 MHz, $CDCl_3$) δ -62.70.

^{13}C NMR (101 MHz, $CDCl_3$) δ 168.6, 163.1, 162.4, 155.9, 151.4, 138.0, 136.8, 135.9, 131.9, 131.3 (q, *J* = 32.4), 130.6, 128.8, 128.4, 128.2, 127.0, 126.4, 126.2, 125.9 (q, *J* = 3.9), 124.0 (q, *J* = 272.5), 112.13, 60.3, 60.2, 57.3, 51.0, 32.3, 30.4, 25.3, 22.2, 16.0, 14.9.

FT-IR(thin film): ν_{max}(cm^{-1}) = 2940, 1616, 1591, 1489, 1452, 1323, 1244, 1165, 1124, 1109, 1066, 1001, 844, 734, 696.

(ESI): m/z calculated for $C_{34}H_{34}O_2N_4F_3S_2$ requires 651.2070 for $[M+H]^+$, found 651.2058.

N-(4-((2-(5-(1-benzylpiperidin-2-yl)-1,3,4-oxadiazol-2-yl)propan-2-yl)oxy)phenethyl)-4-chlorobenzamide - **20**

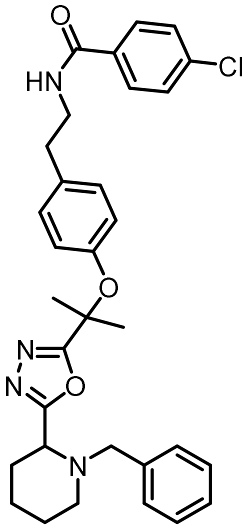

20

Following **general procedure A** (using 0.5 mol% Vaska's complex, and stirring for 1 h after addition of 1.1 eq of acid and NIITP): after FCC (70% Et_2O/Pentane), **20** (87.5 mg, 0.148 mmol, 74%) was afforded as a yellow oil.

^{1}H NMR (400 MHz, $CDCl_3$) δ 7.45 – 7.38 (m, 2H), 7.22 – 7.17 (m, 2H), 7.10 – 6.99 (m, 5H), 6.79 – 6.72 (m, 2H), 6.53 – 6.46 (m, 2H), 6.05 (t, *J* = 5.9, 1H), 3.74 (t, *J* = 6.0, 1H), 3.46 – 3.27 (m, 3H), 3.11 (d, *J* = 13.6, 1H), 2.70 (dt, *J* = 11.6, 4.8, 1H), 2.57 (t, *J* = 6.9, 2H), 2.09 – 2.01 (m, 1H), 1.77 – 1.70 (m, 2H), 1.68 (s, 3H), 1.66 (s, 3H), 1.63 – 1.55 (m, 1H), 1.50 – 1.40 (m, 2H), 1.33 – 1.23 (m, 1H).

^{13}C NMR (101 MHz, $CDCl_3$) δ 168.5, 168.2, 166.4, 153.4, 138.0, 137.6, 134.1, 133.0, 129.6, 128.8, 128.3, 128.2, 127.1, 121.3, 75.2, 60.1, 57.4, 50.8, 41.1, 34.7, 30.5, 26.3, 26.0, 25.2, 22.3.

FT-IR(thin film): ν_{max}(cm^{-1}) = 2938, 2857, 1639, 1596, 1540, 1506, 1486, 1314, 1224, 1154, 1127, 1067, 984, 881, 846, 757, 698.

(ESI): m/z calculated for $C_{32}H_{36}O_3N_4{}^{35}Cl$ requires 559.2470 for $[M+H]^+$, found 559.2464.

2-(1-benzylpiperidin-2-yl)-5-methyl-1,3,4-thiadiazole - 21

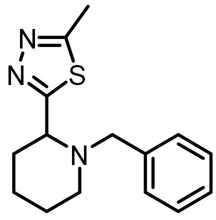

21

Following **general procedure A** (using 0.5 mol% Vaska's complex, and stirring for 1 h after addition of 1.1 eq of acid and NIITP): after FCC (40% Et_2O/Pentane), **21** (25.2 mg, 0.092 mmol, 46%) was afforded as a brown semi-solid.

m.p.: 80 – 82 °C

^{1}H NMR (400 MHz, $CDCl_3$) δ 7.14 (d, *J* = 4.4, 4H), 7.07 (dt, *J* = 5.7, 4.1, 1H), 3.74 (dd, *J* = 10.6, 3.4, 1H), 3.59 (d, *J* = 13.8, 1H), 2.98 (d, *J* = 13.8, 1H), 2.79 (ddt, *J* = 11.7, 3.6, 1.8, 1H), 2.59 (s, 3H), 1.95 – 1.85 (m, 2H), 1.69 – 1.61 (m, 1H), 1.57 – 1.29 (m, 4H).

^{13}C NMR (101 MHz, $CDCl_3$) δ 176.1, 165.4, 138.0, 128.1, 127.8, 126.6, 62.5, 59.6, 52.0, 35.1, 24.8, 23.4, 15.4.

FT-IR(thin film): ν_{max}(cm^{-1}) = 2941, 2804, 1494, 1475, 1373, 1244, 1093, 974, 836, 738, 693.

(ESI): m/z calculated for $C_{15}H_{20}N_3S$ requires 274.1372 for $[M+H]^+$, found 274.1373.

2-(1-benzylpiperidin-2-yl)-5,7-diphenyl-1,3,4-oxadiazepine - 22

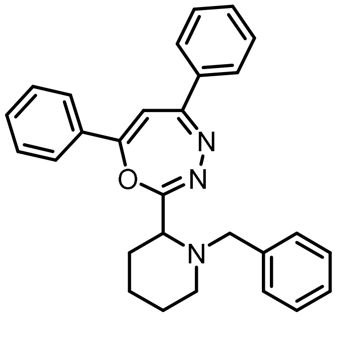

22

Following **general procedure A** (using 1 mol% Vaska's complex, and stirring for 16 h after addition of 2 eq of acid and NIITP): after FCC (10% Et_2O/Pentane —> 20% Et_2O/Pentane), **22** (23.0 mg, 0.05 mmol, 25%) was afforded as a yellow oil.

^{1}H NMR (400 MHz, $CDCl_3$) δ 7.96 – 7.88 (m, 2H), 7.83 – 7.77 (m, 2H), 7.48 – 7.35 (m, 6H), 7.16 – 7.09 (m, 3H), 7.09 – 7.02 (m, 2H), 6.58 (s, 1H), 4.07 (d, *J* = 13.3, 1H), 3.34 – 3.25 (m, 2H), 2.97 (dt, *J* = 11.5, 4.4, 1H), 2.03 – 1.81 (m, 3H), 1.70 (dt, *J* = 13.4, 4.7, 1H), 1.54 – 1.43 (m, 2H), 1.38 – 1.25 (m, 1H).

^{13}C NMR (101 MHz, $CDCl_3$) δ 162.8, 161.3, 154.8, 138.5, 137.5, 132.6, 130.8, 130.4, 129.0, 128.7, 128.5, 128.0, 127.4, 126.7, 126.6, 104.5, 64.6, 60.4, 50.5, 29.2, 25.3, 22.8.

FT-IR(thin film): $\nu_{max}(cm^{-1})$ = 3059, 2934, 2855, 1629, 1576, 1524, 1492, 1447, 1342, 1273, 1170, 1105, 1084, 1066, 1028, 837, 766, 737, 605.

(ESI): m/z calculated for $C_{28}H_{28}ON_3$ requires 422.2227 for $[M+H]^+$, found 422.2222.

3-(1-benzylpiperidin-2-yl)-[1,2,4]triazolo[4,3-c]quinazoline - 23

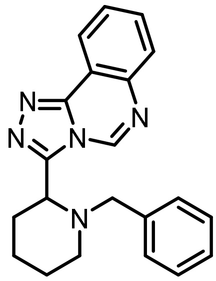

23

Following **general procedure A** (using 1 mol% Vaska's complex, and stirring for 16 h after addition of 2 eq of acid and NIITP): after FCC (100% Et_2O —> 100% EtOAc), **23** (22.8 mg, 0.0 mmol, 33%) was afforded as a yellow crystalline solid.

m.p.; 160 – 162 °C

^{1}H NMR (400 MHz, $CDCl_3$) δ 9.62 (s, 1H), 8.57 (dd, *J* = 7.9, 1.5, 1H), 7.96 (dd, *J* = 8.1, 1.2, 1H), 7.81 – 7.74 (m, 1H), 7.69 (td, *J* = 7.6, 1.3, 1H), 7.19 – 7.13 (m, 2H), 7.12 – 7.08 (m, 2H), 7.08 – 7.02 (m, 1H), 4.21 (dd, *J* = 11.2, 3.5, 1H), 3.54 (d, *J* = 13.7, 1H), 3.28 (d, *J* = 13.7, 1H), 3.17 – 3.09 (m, 1H), 2.22 – 2.12 (m, 1H), 2.08 – 2.00 (m, 1H), 1.98 – 1.87 (m, 2H), 1.82 – 1.73 (m, 1H), 1.73 – 1.63 (m, 1H), 1.56 – 1.46 (m, 1H).

^{13}C NMR (101 MHz, $CDCl_3$) δ 149.8, 148.4, 140.7, 137.1, 135.9, 131.7, 129.4, 128.7, 128.6, 128.3, 127.0, 123.4, 117.2, 61.0, 60.9, 53.5, 31.4, 25.4, 23.9.

FT-IR(thin film): $\nu_{max}(cm^{-1})$ = 2936, 2855, 2801, 1620, 1609, 1527, 1494, 1473, 1453, 1371, 1314, 1302, 1102, 907, 878, 769, 734, 698.

(ESI): m/z calculated for $C_{21}H_{22}N_5$ requires 344.1870 for $[M+H]^+$, found 344.1870.

1-benzyl-2-(5-phenyl-4-tosyl-4H-1,2,4-triazol-3-yl)piperidine - 24

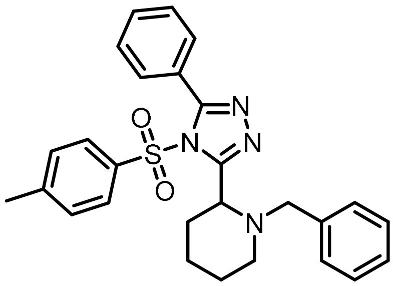

24

Following **general procedure A** (using 1 mol% Vaska's complex, and stirring for 16 h after addition of 2 eq of acid and NIITP): after FCC (40% EtOAc/Pentane), **24** (53.4 mg, 0.114 mmol, 57%) was afforded as an amber oil.

^{1}H NMR (400 MHz, $CDCl_3$) δ 7.36 – 7.29 (m, 1H), 7.26 – 7.06 (m, 11H), 7.02 – 6.95 (m, 2H), 4.45 (dd, *J* = 6.9, 4.6, 1H), 3.59 (d, *J* = 13.3, 1H), 3.19 (d, *J* = 13.2, 1H), 3.10 (ddd, *J* = 11.1, 6.7, 3.6, 1H), 2.24 (s, 4H), 1.97 – 1.84 (m, 2H), 1.76 – 1.63 (m, 1H), 1.61 – 1.45 (m, 2H), 1.45 – 1.32 (m, 1H).

^{13}C NMR (101 MHz, $CDCl_3$) δ 156.6, 153.4, 146.6, 139.0, 134.5, 130.8, 130.4, 129.9, 129.0, 128.2, 127.8, 127.7, 127.0, 126.9, 59.3, 57.9, 50.4, 31.5, 24.7, 22.3, 21.7.

FT-IR(thin film): ν_{max}(cm^{-1}) = 2980, 1595, 1444, 1385, 1265, 1192, 1178, 1121, 1091, 1030, 1010, 909, 813, 731, 698, 664.

(ESI): m/z calculated for $C_{27}H_{29}O_2N_4S$ requires 473.2006 for $[M+H]^+$, found 473.2003.

tert-butyl 3-(1-benzylpiperidin-2-yl)-5-phenyl-4H-1,2,4-triazole-4-carboxylate – 25

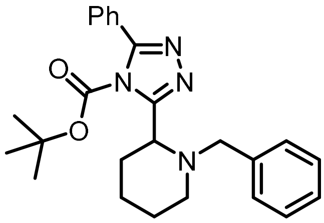

25

Following **general procedure A** (using 0.5 mol% Vaska's complex, and stirring for 16 h after addition of 2 eq of acid and NIITP): after FCC (25% Et_2O/Pentane), **25** (30.3 mg, 0.072 mmol, 36%) was afforded as a colourless oil.

^{1}H NMR (400 MHz, $CDCl_3$) δ 8.33 – 8.24 (m, 2H), 7.50 – 7.42 (m, 3H), 7.30 – 7.25 (m, 2H), 7.25 – 7.18 (m, 3H), 4.39 (dd, *J* = 7.5, 4.1, 1H), 3.80 (d, *J* = 13.1, 1H), 3.46 (d, *J* = 13.1, 1H), 3.40 (ddd, *J* = 10.7, 6.5,

3.5, 1H), 2.42 (ddd, *J* = 11.5, 8.1, 3.6, 1H), 2.03 – 1.92 (m, 1H), 1.91 – 1.80 (m, 2H), 1.80 – 1.70 (m, 2H), 1.70 – 1.64 (m, 1H), 1.61 (s, 9H).

^{13}C NMR (101 MHz, $CDCl_3$) δ 162.2, 161.3, 147.0, 138.2, 130.03, 129.96, 129.3, 128.5, 128.2, 127.3, 127.0, 86.7, 59.8, 57.4, 51.1, 31.2, 27.8, 25.5, 22.2.

FT-IR(thin film): ν_{max}(cm^{-1}) = 2934, 1777, 1754, 1531, 1448, 1371, 1323, 1258, 1152, 1106, 1044, 988, 967, 910, 843, 770, 736, 699.

(ESI): m/z calculated for $C_{25}H_{31}O_2N_4$ requires 419.2442 for $[M+H]^+$, found 419.2442.

1-benzyl-2-(5-phenyl-4H-1,2,4-triazol-3-yl)piperidine - **27**

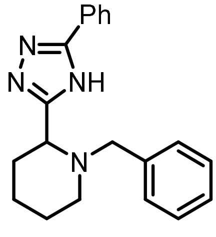

27

Following **general procedure A** (0.1 mmol scale, using 0.5 mol% Vaska's complex, and stirring for 16 h after addition of 2 eq of **25** and NIITP; after reaction completion LiOH (25.2 mg, 0.6 mmol, 6 eq) was added to the reaction mixture and stirred for 1 h before following the work up described in **general procedure A**): after FCC (40% EtOAc/Pentane), **27** (22.3 mg, 0.070 mmol, 70%) was afforded as a beige powder.

m.p.: 196 – 198 °C

^{1}H NMR (400 MHz, DMSO) δ 14.02 (s, 1H), 8.03 (d, *J* = 7.5, 2H), 7.53 – 7.34 (m, 3H), 7.33 – 7.25 (m, 4H), 7.24 – 7.15 (m, 1H), 3.70 – 3.55 (m, 1H), 3.47 (d, *J* = 13.4, 1H), 3.10 (d, *J* = 13.4, 1H), 2.85 (d, *J* = 10.8, 1H), 2.04 (t, *J* = 10.6, 1H), 1.92 – 1.70 (m, 3H), 1.65 – 1.44 (m, 2H), 1.44 – 1.28 (m, 1H).

^{13}C NMR (101 MHz, DMSO) δ 160.9, 159.6, 138.8, 132.0, 129.2, 129.1, 128.5, 127.3, 126.2, 59.7, 59.5, 51.8, 32.4, 25.4, 23.7.

FT-IR(thin film): ν_{max}(cm^{-1}) = 2980, 2928, 1472, 1443, 1373, 1316, 1274, 1254, 1130, 1104, 1073, 1063, 1028, 972, 942, 923, 907, 805, 773, 735, 712, 695, 625.

(ESI): m/z calculated for $C_{20}H_{23}N_4$ requires 319.1917 for $[M+H]^+$, found 319.1917.

3-(1-benzylpiperidin-2-yl)benzo[4,5]isothiazolo[3,2-c][1,2,4]triazole 5,5-dioxide - **28**

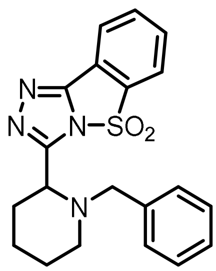

28

Following **general procedure A** (using 0.5 mol% Vaska's complex, and stirring for 16 h after addition of 2 eq of acid and NIITP): after FCC (45% EtOAc/Pentane), **28** (28.9 mg, 0.076 mmol, 38%) was afforded as a white crystalline solid.

m.p.: 70 – 72 °C

^{1}H NMR (400 MHz, $CDCl_3$) δ 8.10 (dt, *J* = 7.7, 0.9, 1H), 7.89 (dt, *J* = 7.9, 0.9, 1H), 7.81 (td, *J* = 7.7, 1.1, 1H), 7.71 (td, *J* = 7.7, 1.1, 1H), 7.40 – 7.35 (m, 2H), 7.33 – 7.27 (m, 2H), 7.26 – 7.20 (m, 1H), 3.92 (dd, *J* = 10.3, 3.6, 1H), 3.83 (d, *J* = 13.5, 1H), 3.40 (d, *J* = 13.5, 1H), 3.01 (dtd, *J* = 11.8, 4.0, 1.2, 1H), 2.16 – 2.03 (m, 2H), 1.95 (dq, *J* = 12.8, 4.0, 1H), 1.89 – 1.79 (m, 1H), 1.66 (tt, *J* = 7.5, 3.4, 2H), 1.45 – 1.30 (m, 1H).

^{13}C NMR (101 MHz, $CDCl_3$) δ 156.6, 151.9, 141.9, 136.5, 134.9, 132.0, 129.7, 128.1, 127.1, 123.3, 123.2, 122.2, 60.5, 58.8, 51.3, 31.1, 24.6, 23.4.

FT-IR(thin film): ν_{max}(cm^{-1}) = 2969, 2931, 2876, 1634, 1436, 1342, 1324, 1298, 1243, 1185, 1165, 1117, 1073, 991, 889, 854, 829, 754, 714, 666.

(ESI): m/z calculated for $C_{20}H_{21}O_2N_4S$ requires 381.1380 for $[M+H]^+$, found 381.1376.

4-((3-(1-benzylpiperidin-2-yl)-5-methyl-4H-1,2,4-triazol-4-yl)sulfonyl)aniline - 29

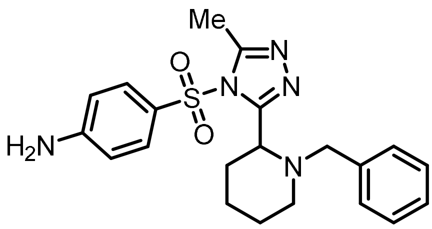

29

Following **general procedure A** (using 1 mol% Vaska's complex, and stirring for 16 h after addition of 2 eq of acid and NIITP): after FCC (75% EtOAc/Pentane) and PLTC (50% EtOAc/$CHCl_3$), **29** (48.9 mg, 0.118 mmol, 59%) was afforded as a white powder.

m.p.: 104 – 106 °C

^{1}H NMR (400 MHz, $CDCl_3$) δ 7.60 (d, *J* = 8.8, 2H), 7.26 – 7.14 (m, 5H), 6.66 (d, *J* = 8.9, 2H), 4.68 (s, 2H), 4.21 (dd, *J* = 8.3, 3.2, 1H), 3.46 (d, *J* = 13.1, 1H), 3.13 – 3.04 (m, 1H), 2.92 (d, *J* = 13.1, 1H), 2.65 (s, 3H), 2.14 (ddd, *J* = 12.0, 8.9, 3.8, 1H), 1.90 – 1.69 (m, 3H), 1.66 – 1.50 (m, 2H), 1.44 – 1.32 (m, 1H).

^{13}C NMR (101 MHz, $CDCl_3$) δ 156.0, 153.1, 150.6, 138.9, 130.2, 129.0, 128.0, 126.8, 124.2, 114.0, 59.6, 58.4, 51.2, 32.1, 24.9, 22.8, 13.9.

FT-IR(thin film): ν_{max}(cm^{-1}) = 3657, 2980, 2888, 1596, 1472, 1461, 1380, 1305, 1251, 1195, 1154, 1133, 1080, 954, 833, 738, 700, 682.

(ESI): m/z calculated for $C_{21}H_{26}O_2N_5S$ requires 412.1802 for $[M+H]^+$, found 412.1810.

2-(1-benzylpyrrolidin-2-yl)-5-phenyl-1,3,4-oxadiazole - 30

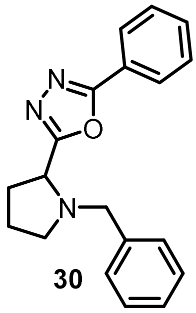

30

Following **general procedure A** (using 0.5 mol% Vaska's complex, and stirring for 16 h after addition of 3 eq of acid and NIITP): after FCC (60% EtOAc/Pentane), **30** (44.5 mg, 0.146 mmol, 73%) was afforded as an amber oil.

^{1}H NMR (400 MHz, $CDCl_3$) δ 8.00 – 7.93 (m, 2H), 7.49 – 7.38 (m, 3H), 7.24 – 7.13 (m, 4H), 7.13 – 7.06 (m, 1H), 4.00 (t, *J* = 7.6, 1H), 3.82 (d, *J* = 13.1, 1H), 3.54 (d, *J* = 13.1, 1H), 3.05 (ddd, *J* = 9.2, 7.9, 3.3, 1H), 2.53 – 2.41 (m, 1H), 2.30 – 2.08 (m, 2H), 2.07 – 1.92 (m, 1H), 1.91 – 1.78 (m, 1H).

^{13}C NMR (101 MHz, $CDCl_3$) δ 167.3, 165.1, 137.9, 131.6, 129.0, 128.2, 127.2, 127.0, 124.0, 58.8, 58.0, 53.3, 30.1, 23.0.

FT-IR(thin film): ν_{max}(cm^{-1}) = 2980, 2803, 1655, 1609, 1551, 1485, 1449, 1372, 1258, 1176, 1070, 1026, 1007, 777, 740, 690.

(ESI): m/z calculated for $C_{19}H_{20}ON_3$ requires 306.1601 for $[M+H]^+$, found 306.1602.

2-(1-methylazepan-2-yl)-5-phenyl-1,3,4-oxadiazole - **31**

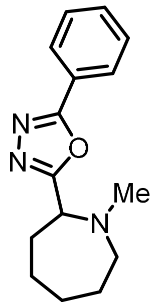

31

Following **general procedure A** (using 0.5 mol% Vaska's complex, and stirring for 1 h after addition of 1.1 eq of acid and NIITP): after FCC (40% Et_2O/Pentane), **31** (35.1 mg, 0.136 mmol, 68%) was afforded as a yellow oil.

^1H NMR (400 MHz, $CDCl_3$) δ 8.09 – 8.00 (m, 2H), 7.54 – 7.43 (m, 3H), 4.07 (dd, *J* = 8.1, 6.3, 1H), 3.14 – 3.02 (m, 1H), 2.74 (dt, *J* = 14.6, 4.9, 1H), 2.43 (s, 3H), 2.16 – 2.02 (m, 2H), 1.92 – 1.81 (m, 1H), 1.75 – 1.63 (m, 4H), 1.59 – 1.46 (m, 1H).

^{13}C NMR (101 MHz, $CDCl_3$) δ 168.3, 164.8, 131.5, 128.9, 126.9, 124.1, 60.6, 53.4, 43.1, 31.9, 28.7, 28.1, 25.5.

FT-IR(thin film): $\nu_{max}(cm^{-1})$ = 2980, 2930, 1553, 1472, 1449, 1381, 1252, 1152, 1085, 1070, 959, 800, 706, 690.

(ESI): m/z calculated for $C_{15}H_{20}ON_3$ requires 258.1601 for $[M+H]^+$, found 258.1603.

2-(1-(prop-2-yn-1-yl)azepan-2-yl)-5-(4-(trifluoromethoxy)phenyl)-1,3,4-oxadiazole - **32**

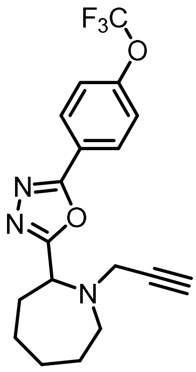

32

Following **general procedure A** (using 1 mol% Vaska's complex, and stirring for 1 h after addition of 1.1 eq of acid and NIITP): after FCC (25% Et_2O/Pentane), **32** (27.9 mg, 0.076 mmol, 38%) was afforded as an orange crystalline solid.

m.p: 94 – 96 °C

^{1}H NMR (400 MHz, $CDCl_3$) δ 8.14 – 8.08 (m, 2H), 7.37 – 7.31 (m, 2H), 4.39 (dd, *J* = 8.4, 5.8, 1H), 3.59 – 3.44 (m, 2H), 3.09 (ddd, *J* = 14.7, 7.2, 3.2, 1H), 2.92 (dt, *J* = 14.5, 3.8, 1H), 2.23 – 2.13 (m, 1H), 2.11 – 2.00 (m, 2H), 1.92 – 1.81 (m, 1H), 1.79 – 1.64 (m, 4H), 1.62 – 1.47 (m, 1H).

^{19}F NMR (377 MHz, CDCl3) δ -57.71.

^{13}C NMR (101 MHz, $CDCl_3$) δ 168.4, 163.7, 151.5, 128.7, 122.7, 121.2, 120.7 (q, *J* = 259.5), 80.1, 72.1, 57.7, 50.3, 45.0, 32.5, 29.5, 29.3, 24.8.

FT-IR(thin film): ν_{max}(cm^{-1}) = 2932, 1545, 1498, 1454, 1340, 1254, 1209, 1159, 1111, 1010, 733, 701.

(ESI): m/z calculated for $C_{18}H_{19}O_2N_3F_3$ requires 366.1424 for $[M+H]^+$, found 366.1422.

2-(1-allylazocan-2-yl)-5-(2,3-dihydrobenzo[b][1,4]dioxin-6-yl)-1,3,4-oxadiazole - **33**

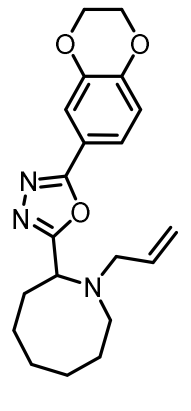

33

Following **general procedure A** (using 1 mol% Vaska's complex, and stirring for 1 h after addition of 1.1 eq of acid and NIITP): after FCC (50% Et_2O/Pentane), **33** (29.6 mg, 0.084 mmol, 42%) was afforded as a yellow oil.

^{1}H NMR (400 MHz, $CDCl_3$) δ 7.56 – 7.51 (m, 2H), 6.98 – 6.93 (m, 1H), 5.84 (ddt, *J* = 16.9, 10.0, 6.6, 1H), 5.17 (dq, *J* = 17.2, 1.6, 1H), 5.09 (dq, *J* = 10.0, 1.3, 1H), 4.31 (tt, *J* = 4.9, 2.6, 4H), 4.13 (dd, *J* = 12.0, 5.0, 1H), 3.29 (ddt, *J* = 13.8, 6.9, 1.3, 1H), 3.16 (ddt, *J* = 13.8, 6.3, 1.4, 1H), 3.02 (td, *J* = 10.2, 5.0, 1H), 2.59 – 2.39 (m, 1H), 2.16 (m, 1H), 2.05 – 1.87 (m, 2H), 1.85 – 1.65 (m, 2H), 1.64 –1.50 (m, 3H) 1.48 – 1.38 (m, 2H).

^{13}C NMR (101 MHz, $CDCl_3$) δ 166.9, 164.1, 146.5, 143.8, 136.7, 120.5, 118.0, 117.5, 117.3, 116.1, 64.6, 64.3, 58.4, 57.1, 47.1, 30.5, 28.8, 27.9, 26.6, 25.8.

FT-IR(thin film): ν_{max}(cm^{-1}) = 2980, 2928, 1617, 1593, 1564, 1500, 1418, 1311, 1283, 1252, 1151, 1125, 1065, 966, 894, 870, 818, 733.

(ESI): m/z calculated for $C_{20}H_{26}O_3N_3$ requires 356.1969 for $[M+H]^+$, found 356.1969.

N-((4-fluorophenyl)(5-(thiophen-2-yl)-1,3,4-oxadiazol-2-yl)methyl)-N-methylaniline - 34

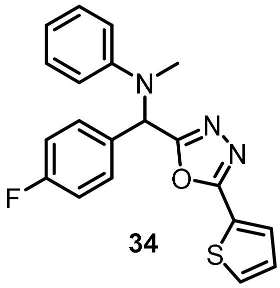

34

Following **general procedure A** (using 5 mol% Vaska's complex, and stirring for 1 h after addition of 1.1 eq of acid and NIITP): after FCC (30% Et_2O/Pentane), **34** (35.5 mg, 0.098 mmol, 49%) was afforded as a brown oil.

^{1}H NMR (400 MHz, $CDCl_3$) δ 7.63 (dd, *J* = 3.8, 1.2, 1H), 7.47 (dd, *J* = 5.0, 1.2, 1H), 7.27 – 7.17 (m, 4H), 7.07 (dd, *J* = 5.0, 3.8, 1H), 7.03 – 6.96 (m, 2H), 6.90 (dt, *J* = 7.8, 1.0, 2H), 6.78 (tt, *J* = 7.2, 1.0, 1H), 6.39 (s, 1H), 2.83 (s, 3H).

^{19}F NMR (377 MHz, $CDCl_3$) δ -113.4.

^{13}C NMR (101 MHz, $CDCl_3$) δ 164.3, 162.6 (d, *J* = 247.9), 161.4, 149.2, 131.6 (d, *J* = 3.2), 130.5, 130.1, 129.6 (d, *J* = 8.3), 129.4, 128.2, 124.9, 119.2, 115.8 (d, *J* = 21.6), 114.6, 59.1, 34.6.

FT-IR(thin film): ν_{max}(cm^{-1}) = 2980, 2923, 1596, 1555, 1505, 1424, 1265, 1225, 1159, 1105, 1034, 992, 844, 749, 723, 692.

(ESI): m/z calculated for $C_{20}H_{17}ON_3FS$ requires 366.1071 for $[M+H]^+$, found 366.1071.

N-((5-(furan-2-yl)-1,3,4-oxadiazol-2-yl)(4-methoxyphenyl)methyl)-N-methylaniline - 35

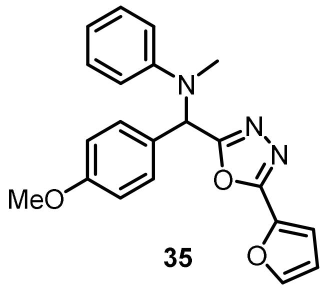

35

Following **general procedure A** (using 5 mol% Vaska's complex, and stirring for 1 h after addition of 1.1 eq of acid and NIITP): after FCC (35% Et_2O/Pentane), **35** (48.0 mg, 0.132 mmol, 66%) was afforded as a dark crystalline solid.

m.p.: 118 – 120 °C

^1H NMR (400 MHz, $CDCl_3$) δ 7.54 (dd, *J* = 1.8, 0.8, 1H), 7.22 – 7.16 (m, 2H), 7.16 – 7.11 (m, 2H), 7.06 (dd, *J* = 3.5, 0.8, 1H), 6.91 – 6.86 (m, 2H), 6.85 – 6.80 (m, 2H), 6.75 (tt, *J* = 7.3, 1.0, 1H), 6.49 (dd, *J* = 3.5, 1.8, 1H), 6.39 (s, 1H), 3.73 (s, 3H), 2.82 (s, 3H).

^{13}C NMR (101 MHz, $CDCl_3$) δ 164.8, 159.6, 157.9, 149.3, 145.8, 139.3, 129.4, 129.2, 127.7, 118.9, 114.4, 114.3, 114.2, 112.2, 59.0, 55.3, 34.4.

FT-IR(thin film): $\nu_{max}(cm^{-1})$ = 2924, 2853, 1636, 1597, 1533, 1455, 1304, 1251, 1176, 1098, 1029, 901, 735, 692.

(ESI): m/z calculated for $C_{21}H_{20}O_3N_3$ requires 362.1499 for $[M+H]^+$, found 362.1500.

4-(5-((4-fluorophenyl)(indolin-1-yl)methyl)-1,3,4-oxadiazol-2-yl)benzonitrile - 36

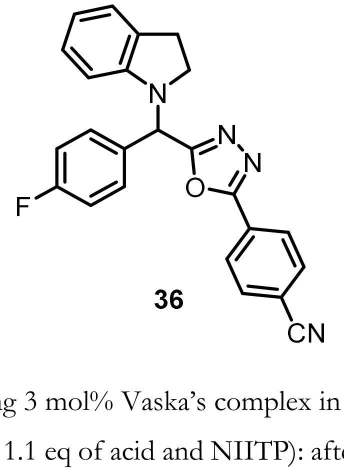

36

Following **general procedure A** (using 3 mol% Vaska's complex in PhMe for the 1st step, and stirring for 1 h after addition of THF (1 mL), and 1.1 eq of acid and NIITP): after FCC (35% Et_2O/Pentane), **36** (67.3 mg, 0.170 mmol, 85%) was afforded as a brown crystalline solid.

m.p.: 132 – 134 °C

^{1}H NMR (400 MHz, $CDCl_3$) δ 8.12 – 8.05 (m, 2H), 7.78 – 7.71 (m, 2H), 7.47 – 7.40 (m, 2H), 7.11 – 7.04 (m, 3H), 7.00 (td, *J* = 7.7, 1.3, 1H), 6.70 (td, *J* = 7.4, 1.0, 1H), 6.51 (d, *J* = 7.9, 1H), 6.15 (s, 1H), 3.59 (q, *J* = 8.9, 1H), 3.34 (td, *J* = 8.7, 6.4, 1H), 3.03 – 2.93 (m, 2H).

^{19}F NMR (377 MHz, $CDCl_3$) δ -112.75.

^{13}C NMR (101 MHz, $CDCl_3$) δ 165.4, 163.8, 162.8 (d, *J* = 248.4), 149.7, 132.9, 130.7 (d, *J* = 3.4), 130.3, 130.0 (d, *J* = 8.3), 127.50, 127.47, 127.3, 125.0, 119.3, 117.8, 116.0 (d, *J* = 21.8), 115.4, 108.0, 56.7, 50.2, 28.2.

FT-IR(thin film): ν_{max}(cm^{-1}) = 2980, 2360, 2230, 1605, 1548, 1508, 1487, 1384, 1226, 1159, 1083, 1014, 962, 845, 802, 739, 699.

(ESI): m/z calculated for $C_{24}H_{18}ON_4F$ requires 397.1459 for $[M+H]^+$, found 397.1459.

(4-chlorophenyl)(5-methoxy-2-methyl-3-((5-((methyl(phenyl)amino)methyl)-1,3,4-oxadiazol-2-yl)methyl)-1H-indol-1-yl)methanone - 37

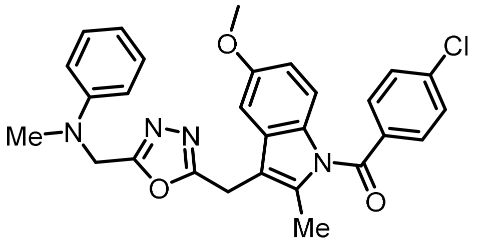

37

Following **general procedure A** (using 5 mol% Vaska's complex, and stirring for 1 h after addition of 1.1 eq of acid and NIITP): after FCC (40% EtOAc/Pentane), **37** (54.3 mg, 0.138 mmol, 69%) was afforded as a brown oil.

^{1}H NMR (400 MHz, $CDCl_3$) δ 7.52 – 7.46 (m, 2H), 7.35 – 7.28 (m, 2H), 7.10 – 7.02 (m, 2H), 6.80 (d, *J* = 2.5, 1H), 6.70 (d, *J* = 9.0, 1H), 6.68 – 6.59 (m, 3H), 6.53 (dd, *J* = 9.0, 2.5, 1H), 4.46 (s, 2H), 4.04 (s, 2H), 3.63 (s, 3H), 2.87 (s, 3H), 2.24 (s, 3H).

^{13}C NMR (101 MHz, $CDCl_3$) δ 168.3, 165.1, 164.4, 156.2, 148.4, 139.5, 136.0, 133.7, 131.2, 130.8, 130.0, 129.3, 129.2, 128.6, 125.5, 118.3, 115.0, 113.2, 112.1, 111.8, 100.9, 55.7, 47.6, 38.9, 20.9, 13.2.

FT-IR(thin film): ν_{max}(cm^{-1}) = 3657, 2980, 2889, 1681, 1599, 1505, 1477, 1455, 1356, 1314, 1176, 1119, 1087, 1067, 1034, 899, 823, 751, 733, 691.

(ESI): m/z calculated for $C_{28}H_{26}O_3N_4{}^{35}Cl$ requires 501.1688 for $[M+H]^+$, found 501.1688.

ethyl (E)-5-(((5-(4-bromophenyl)-1,3,4-oxadiazol-2-yl)(4-methoxyphenyl)methyl)(phenyl)amino)pent-2-enoate - **38**

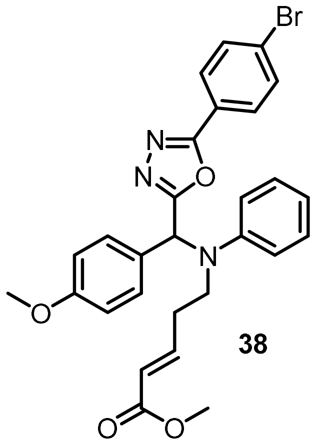

38

Following **general procedure A** (0.1 mmol scale, using 0.5 mol% Vaska's complex, and stirring for 1 h after addition of 1.1 eq of acid and NIITP): after FCC (35% Et_2O/Pentane), **38** (29.5 mg, 0.108 mmol, 54%) was afforded as a dark oil.

^1H NMR (500 MHz, $CDCl_3$) δ 7.92 – 7.86 (m, 2H), 7.69 – 7.62 (m, 2H), 7.29 (tq, *J* = 7.2, 2.5, 1.7, 4H), 7.03 – 6.98 (m, 2H), 6.95 – 6.88 (m, 3H), 6.78 (dt, *J* = 15.6, 7.1, 1H), 6.31 (s, 1H), 5.71 (dt, *J* = 15.7, 1.5, 1H), 3.84 (s, 3H), 3.71 (s, 3H), 3.48 (m, 2H), 2.35 (m, 1H), 2.28 – 2.17 (m, 1H).

^{13}C NMR (126 MHz, $CDCl_3$) δ 166.7, 165.8, 164.3, 159.8, 147.5, 146.0, 132.4, 129.7, 129.5, 128.4, 127.6, 126.6, 122.6, 122.3, 120.6, 117.5, 114.3, 60.6, 55.3, 51.4, 46.5, 30.7.

FT-IR(thin film): $\nu_{max}(cm^{-1})$ = 2980, 2889, 1720, 1656, 1601, 1511, 1480, 1436, 1382, 1250, 1175, 1082, 1032, 1008, 966, 833, 749, 732, 694.

(ESI): m/z calculated for $C_{28}H_{27}O_4N_3{}^{79}Br$ requires 548.1179 for $[M+H]^+$, found 548.1178.

tert-butyl 4-((5-((((benzyloxy)carbonyl)amino)methyl)-1,3,4-oxadiazol-2-yl)(2-iodophenyl)methyl)piperazine-1-carboxylate - 39

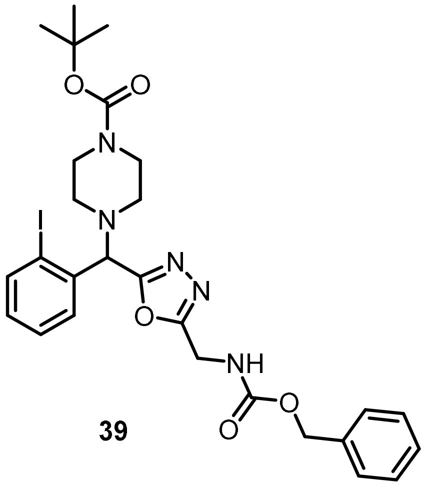

39

Following **general procedure A** (using 5 mol% Vaska's complex, and stirring for 1 h after addition of 1.1 eq of acid and NIITP): after FCC (35% EtOAc/Pentane), **39** (69.7 mg, 0.110 mmol, 55%) was afforded as an amber coloured solid.

m.p.: 60 – 62 °C

^{1}H NMR (400 MHz, $CDCl_3$) δ 7.73 (dd, *J* = 8.0, 1.3, 1H), 7.52 (d, *J* = 7.8, 1H), 7.23 – 7.13 (m, 6H), 6.88 (td, *J* = 7.6, 1.7, 1H), 5.42 (s, 1H), 5.13 (s, 1H), 4.99 (s, 2H), 4.45 (d, *J* = 6.0, 2H), 3.34 – 3.20 (m, 4H), 2.46 – 2.35 (m, 2H), 2.31 – 2.21 (m, 2H), 1.29 (s, 9H).

^{13}C NMR (101 MHz, $CDCl_3$) δ 165.1, 164.2, 156.1, 154.6, 140.3, 137.6, 136.0, 130.3, 130.0, 128.7, 128.6, 128.6, 128.6, 128.3, 128.2, 101.0, 79.8, 69.0, 67.4, 50.3, 43.1, 36.4, 28.4.

FT-IR(thin film): ν_{max}(cm^{-1}) = 3657, 2980, 2889, 1723, 1689, 1584, 1532, 1456, 1422, 1392, 1366, 1246, 1072, 998, 967, 688, 751, 734, 698.

(ESI): m/z calculated for $C_{27}H_{33}O_5N_5I$ requires 634.1521 for $[M+H]^+$, found 634.1515.

4-((5-((1H-indol-3-yl)methyl)-1,3,4-oxadiazol-2-yl)(4-nitrophenyl)methyl)morpholine - 40

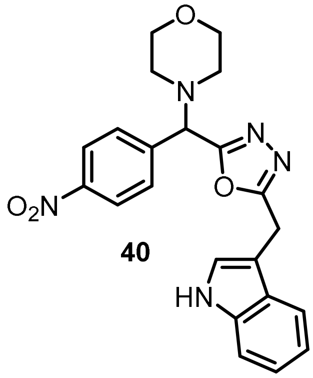

40

Following **general procedure A** (using 1 mol% Vaska's complex, and stirring for 1 h after addition of 1.1 eq of acid and NIITP): after FCC (35% Acetone/Pentane), **40** (39.8 mg, 0.094 mmol, 47%) was afforded as a dark oil.

^{1}H NMR (400 MHz, $CDCl_3$) δ 8.38 (br, 1H), 8.16 – 8.07 (m, 2H), 7.64 – 7.57 (m, 2H), 7.50 (d, *J* = 8.0, 1H), 7.34 (dt, *J* = 8.2, 1.0, 1H), 7.19 (ddd, *J* = 8.2, 7.0, 1.2, 1H), 7.12 (d, *J* = 2.5, 1H), 7.07 (ddd, *J* = 8.0, 7.0, 1.0, 1H), 4.92 (s, 1H), 4.35 (d, *J* = 0.9, 2H), 3.66 (dd, *J* = 5.3, 4.0, 4H), 2.46 (dt, *J* = 11.6, 4.7, 2H), 2.32 (dt, *J* = 11.2, 4.6, 2H).

^{13}C NMR (101 MHz, $CDCl_3$) δ 167.1, 164.0, 148.0, 142.6, 136.3, 129.6, 126.5, 123.9, 123.1, 122.5, 119.9, 118.4, 111.5, 107.9, 66.6, 65.6, 51.3, 22.3.

FT-IR(thin film): ν_{max}(cm^{-1}) = 2980, 2889, 1579, 1521, 1382, 1348, 1264, 1252, 1154, 1114, 955, 880, 835, 810, 732, 700.

(ESI): m/z calculated for $C_{22}H_{22}O_4N_5$ requires 420.1666 for $[M+H]^+$, found 420.1660.

2-((4-methylpiperazin-1-yl)(4-(4,4,5,5-tetramethyl-1,3,2-dioxaborolan-2-yl)phenyl)methyl)-5-(phenanthren-9-yl)-1,3,4-oxadiazole - **41**

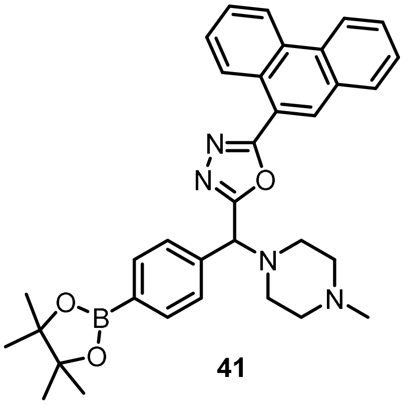

41

Following **general procedure A** (using 1 mol% Vaska's complex, and stirring for 1 h after addition of 1.1 eq of acid and NIITP): after FCC (7% MeOH/CH_2Cl_2), **41** (61.7 mg, 0.110 mmol, 55%) was afforded as a dark oil.

^{1}H NMR (400 MHz, $CDCl_3$) δ 9.22 – 9.15 (m, 1H), 8.74 – 8.67 (m, 1H), 8.65 (d, *J* = 8.3, 1H), 8.37 (s, 1H), 7.95 (dd, *J* = 8.0, 1.4, 1H), 7.91 – 7.85 (m, 2H), 7.75 – 7.67 (m, 3H), 7.67 – 7.58 (m, 3H), 5.06 (s, 1H), 2.76 (br, s, 2H), 2.63 (br, s, 6H), 2.36 (s, 3H), 1.32 (s, 12H).

^{13}C NMR (101 MHz, $CDCl_3$) δ 165.3, 164.9, 138.9, 135.4, 131.7, 130.9, 130.6, 130.2, 129.8, 128.9, 128.1, 127.9, 127.8, 127.4, 127.2, 127.0, 122.9, 122.7, 119.3, 84.0, 66.5, 54.7, 50.6, 45.4, 24.9.

FT-IR(thin film): ν_{max}(cm^{-1}) = 2980, 2888, 1611, 1539, 1472, 1380, 1359, 1293, 1264, 1142, 1088, 962, 857, 814, 768, 733, 565.

(ESI): m/z calculated for $C_{34}H_{38}O_3N_4{}^{10}B$ requires 561.3036 for $[M+H]^+$, found 561.3027.

N,O-dimethyl-N-((5-(5-methyl-2-phenyloxazol-4-yl)-1,3,4-oxadiazol-2-yl)(phenyl)methyl)hydroxylamine - 42

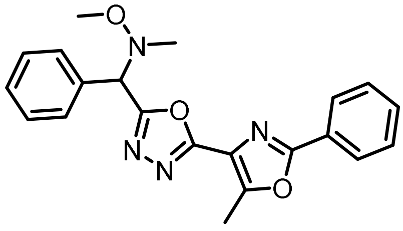

42

Following **general procedure A** (using 1 mol% Vaska's complex, and stirring for 1 h after addition of 1.1 eq of acid and NIITP): after FCC (40% Et_2O/Pentane), **42** (32.0 mg, 0.086 mmol, 43%) was afforded as a brown oil.

^{1}H NMR (400 MHz, $CDCl_3$) δ 7.99 – 7.83 (m, 2H), 7.50 – 7.38 (m, 2H), 7.38 – 7.24 (m, 3H), 7.24 – 7.12 (m, 3H), 4.98 (s, 1H), 3.33 (s, 3H), 2.61 (s, 3H), 2.42 (s, 3H).

^{13}C NMR (101 MHz, $CDCl_3$) δ 165.1, 160.9, 159.7, 151.8, 135.1, 130.9, 129.0, 129.0, 128.8, 126.6, 126.5, 123.6, 69.2, 60.3, 42.7, 11.9.

FT-IR(thin film): ν_{max}(cm^{-1}) = 3062, 2980, 2890, 1644, 1564, 1546, 1489, 1450, 1333, 1197, 1088, 1072, 1057, 1042, 958, 812, 776, 708, 694.

(ESI): m/z calculated for $C_{21}H_{21}O_3N_4$ requires 377.1608 for $[M+H]^+$, found 377.1608.

4-((dimethylamino)(5-(3-iodophenyl)-1,3,4-oxadiazol-2-yl)methyl)-9H-fluoren-9-one - 43

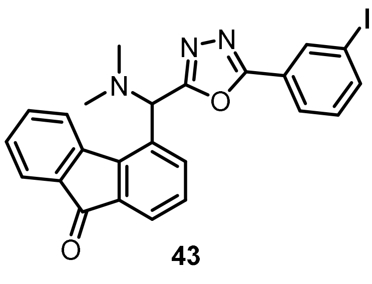

43

Following **general procedure A** (using 1 mol% Vaska's complex, and stirring for 1 h after addition of 1.1 eq of acid and NIITP): after FCC (40% Et_2O/Pentane), **43** (61.1 mg, 0.120 mmol, 60%) was afforded as a yellow crystalline solid.

m.p.: 70 – 72 °C

^{1}H NMR (400 MHz, $CDCl_3$) δ 8.32 (t, *J* = 1.7, 1H), 8.09 (d, *J* = 7.7, 1H), 7.99 (dt, *J* = 7.8, 1.3, 1H), 7.85 (ddd, *J* = 7.9, 1.8, 1.0, 1H), 7.82 (dd, *J* = 8.0, 1.2, 1H), 7.75 – 7.71 (m, 1H), 7.69 (dd, *J* = 7.3, 1.2, 1H), 7.60 (td, *J* = 7.6, 1.3, 1H), 7.39 – 7.33 (m, 2H), 7.22 (t, *J* = 7.9, 1H), 5.48 (s, 1H), 2.44 (s, 6H).

^{13}C NMR (101 MHz, $CDCl_3$) δ 193.3, 164.8, 163.9, 143.7, 143.0, 140.8, 135.6, 135.5, 135.1, 135.0, 134.6, 132.7, 130.7, 129.3, 129.2, 126.1, 125.4, 124.4, 124.3, 94.3, 63.2, 43.1.

FT-IR(thin film): $\nu_{max}(cm^{-1})$ =2980, 1711, 1605, 1575, 1542, 1463, 1425, 1239, 1169, 1094, 1039, 994, 961, 888, 811, 792, 766, 734, 721, 678.

(ESI): m/z calculated for $C_{24}H_{19}O_2N_3I$ requires 508.0516 for $[M+H]^+$, found 508.0516.

2-(5-(1-(dimethylamino)-3-phenylpropyl)-1,3,4-oxadiazol-2-yl)benzonitrile - **44**

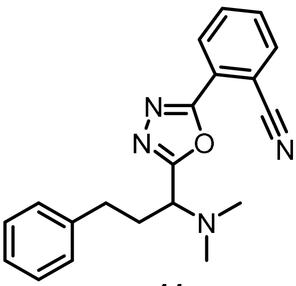

44

Following **general procedure A** (using 1 mol% Vaska's complex, and stirring for 16 h after addition of 2 eq of acid and NIITP): after FCC (45% EtOAc/Pentane), **44** (32.8 mg, 0.098 mmol, 49%) was afforded as an amber oil.

^{1}H NMR (400 MHz, $CDCl_3$) δ 8.21 (ddd, *J* = 8.0, 1.4, 0.5, 1H), 7.79 (ddd, *J* = 7.7, 1.4, 0.5, 1H), 7.69 (td, *J* = 7.8, 1.4, 1H), 7.58 (td, *J* = 7.7, 1.3, 1H), 7.19 (tdd, *J* = 7.2, 2.2, 1.0, 2H), 7.16 – 7.11 (m, 2H), 7.11 – 7.06 (m, 1H), 3.93 (t, *J* = 7.6, 1H), 2.74 – 2.61 (m, 2H), 2.30 (s, 6H), 2.29 – 2.22 (m, 2H).

^{13}C NMR (101 MHz, $CDCl_3$) δ 166.2, 162.5, 140.9, 134.8, 133.1, 131.7, 129.5, 128.6, 128.5, 126.1, 126.1, 117.0, 110.5, 59.5, 41.4, 32.2, 32.1.

FT-IR(thin film): $\nu_{max}(cm^{-1})$ = 2941, 2866, 2832, 2786, 2361, 2229, 1601, 1541, 1493, 1468, 1453, 1437, 1157, 1029, 965, 775, 747, 699.

(ESI): m/z calculated for $C_{20}H_{21}ON_4$ requires 333.1710 for $[M+H]^+$, found 333.1709.

tert-butyl (R)-(1-(5-((dimethylamino)methyl)-1,3,4-oxadiazol-2-yl)-2-(naphthalen-2-yl)ethyl)carbamate - 45

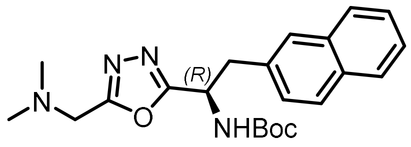

45

Following **general procedure A** (using 0.5 mol% Vaska's complex, and stirring for 1 h after addition of 1.1 eq of acid and NIITP): after FCC (40% Acetone/Pentane) and PTLC (85% EtOAc/Hexane), **45** (46.3 mg, 0.116 mmol, 58%) was afforded as a colourless oil.

ee: >99.5% [HPLC CHIRALPAK® AD-H, Hexane/IPA = 85/15, 1 mL/min, λ = 220 nm, $t_{(S)}$ = 16.5 min, $t_{(R)}$ = 19.0 min].

^{1}H NMR (400 MHz, $CDCl_3$) δ 7.83 – 7.70 (m, 3H), 7.56 (s, 1H), 7.49 – 7.41 (m, 2H), 7.22 (dd, *J* = 8.4, 1.8, 1H), 5.45 – 5.31 (m, 1H), 5.25 – 5.10 (m, 1H), 3.69 (s, 2H), 3.41 (d, *J* = 6.5, 2H), 2.23 (s, 6H), 1.38 (s, 9H).

^{13}C NMR (101 MHz, $CDCl_3$) δ 167.0, 164.0, 133.4, 132.8, 132.6, 128.5, 128.2, 127.6, 127.6, 127.2, 126.2, 125.9, 80.5, 52.7, 48.5, 44.9, 40.2, 28.2.

FT-IR(thin film): ν_{max}(cm^{-1}) = 3348, 2977, 2771, 1693, 1509, 1459, 1365, 1326, 1279, 1265, 1243, 1162, 1047, 1024, 869, 847, 821, 772, 750, 645.

(ESI): m/z calculated for $C_{22}H_{29}O_3N_4$ requires 397.2234 for $[M+H]^+$, found 397.2236.

$[\alpha]_D^{25}$= 35.47 (c= 0.21, $CHCl_3$).

Enantiopurity was of **(R)-45** was confirmed by synthesis of **(S)-45** starting from a Boc-3-(2-naphthyl)-L-alanine and combining with **(R)-45** to create a scalemic mixture for separation by chiral HPLC. Relevant HPLC traces are below.

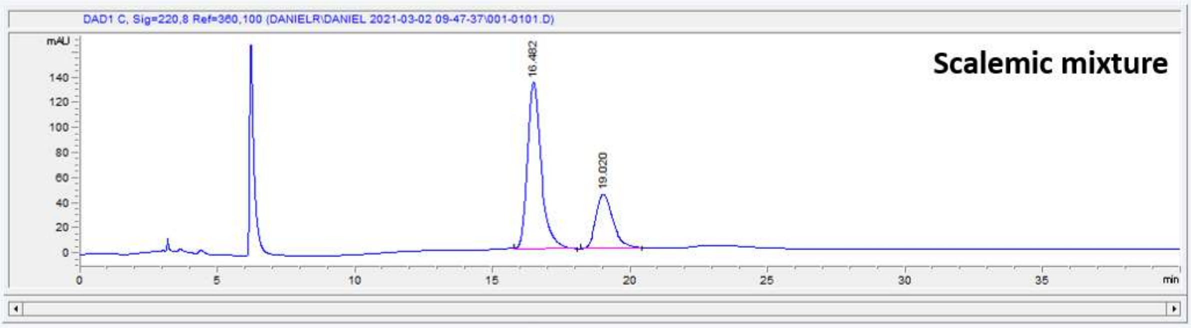

File Information	
LC-File	001-0101.D
File Path	C:\CHEM32\1\DATA\DANIELR\DANIEL 2021-03-02 09-47-37\
Date	02-Mar-21, 09:59:45
Sample	DMR-721-Rac
Sample Info	DMR-721-Rac, 15% IPA, 1 mL/min, 40 mins, AD-H
Barcode	
Operator	DMR
Method	85-15_1ML_40MIN_VALVE1.M

#	Time	Area	Height	Width	Area%	Symmetry
1	16.482	4574.7	133	0.5194	71.404	0.712
2	19.02	1832.1	43.1	0.6521	28.596	0.733

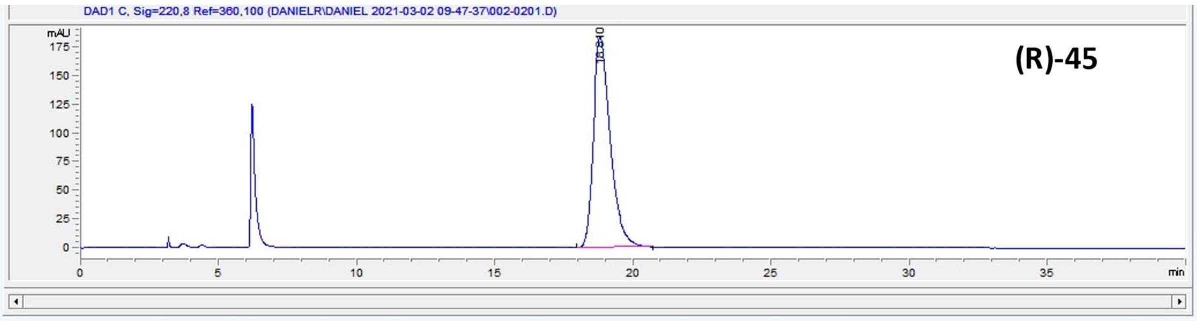

File Information	
LC-File	002-0201.D
File Path	C:\CHEM32\1\DATA\DANIELR\DANIEL 2021-03-02 09-47-37\
Date	02-Mar-21, 10:41:52
Sample	DMR-721-D
Sample Info	DMR-721-D, 15% IPA, 1 mL/min, 40 mins, AD-H
Barcode	
Operator	DMR

#	Time	Area	Height	Width	Area%	Symmetry
1	18.81	7859.3	183.5	0.6518	100.000	0.658

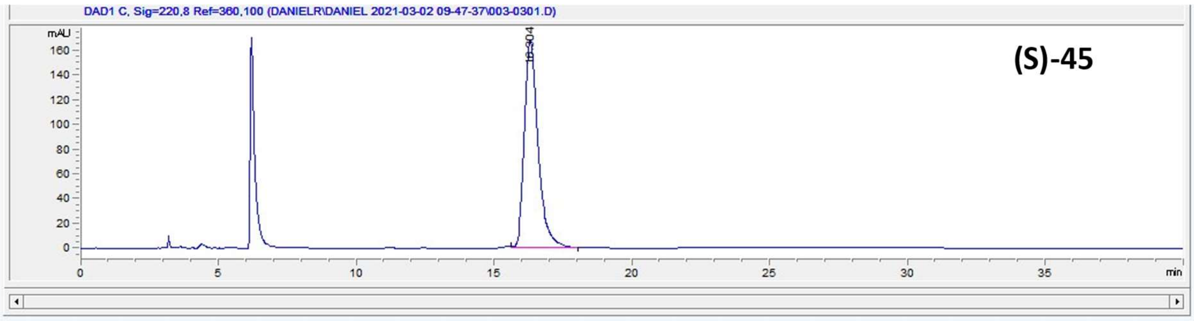

File Information	
LC-File	003-0301.D
File Path	C:\CHEM32\1\DATA\DANIELR\DANIEL 2021-03-02 09-47-37\
Date	02-Mar-21, 11:23:51
Sample	DMR-721-L
Sample Info	DMR-721-L, 15% IPA, 1 mL/min, 40 mins, AD-H
Barcode	
Operator	DMR

#	Time	Area	Height	Width	Area%	Symmetry
1	16.304	5702.2	167.2	0.5139	100.000	0.702

1-(4-(5-(pyrrolidin-1-ylmethyl)-1,3,4-oxadiazol-2-yl)piperidin-1-yl)ethan-1-one - **46**

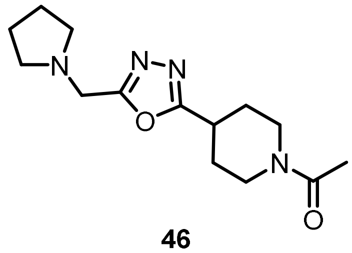

46

Following **general procedure A** (2 mmol scale, using 1 mol% Vaska's complex, and stirring for 1 h after addition of 1.1 eq of acid and NIITP): after FCC (EtOAc —> 10% MeOH/CH_2Cl_2), **46** (292 mg, 1.16 mmol, 58%) was afforded as a dark oil.

^{1}H NMR (400 MHz, $CDCl_3$) δ 4.45 (dtd, *J* = 13.6, 4.3, 1.6, 1H), 3.91 (s, 2H), 3.86 (dtd, *J* = 14.0, 4.3, 1.5, 1H), 3.24 (ddd, *J* = 14.0, 11.1, 3.0, 1H), 3.15 (tt, *J* = 10.6, 4.0, 1H), 2.91 (ddd, *J* = 13.9, 11.1, 3.1, 1H), 2.72 – 2.63 (m, 4H), 2.16 – 2.03 (m, 5H), 1.95 – 1.73 (m, 6H).

^{13}C NMR (101 MHz, $CDCl_3$) δ 168.9, 168.7, 164.1, 54.0, 49.1, 45.5, 40.6, 33.2, 29.3, 28.8, 23.7, 21.4.

FT-IR(thin film): ν_{max}(cm^{-1}) = 2970, 2801, 1635, 1586, 1561, 1435, 1372, 1295, 1272, 1229, 1181, 1143, 1117, 1044, 978, 875, 722, 697.

(ESI): m/z calculated for $C_{14}H_{23}O_2N_4$ requires 279.1816 for $[M+H]^+$, found 279.1816.

1,3,5-tris(5-(piperidin-1-ylmethyl)-1,3,4-oxadiazol-2-yl)benzene - **47**

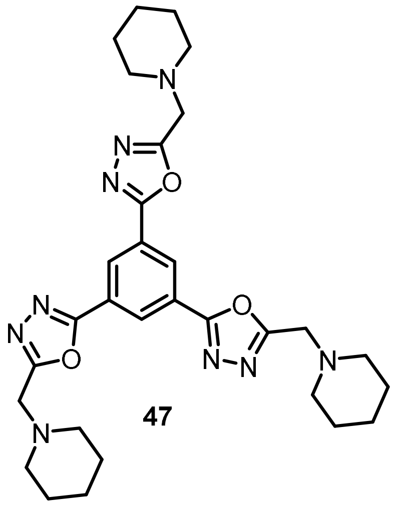

47

To an oven dried screw cap vial equipped with magnetic stirrer *N*-Formylpiperidine (67.9 mg, 0.6 mmol, 6 eq), and Vaska's complex (4.7 mg, 0.006 mmol, 1 mol% with respect to the amide) were added. The vial was evacuated under vacuum and backfilled with nitrogen (x3). Then anhydrous THF (3 mL) was added followed in quick succession by TMDS (216 μL, 1.2 mmol, 12 eq), and the vial capped and stirred at rt.

After 30 mins, the vial was opened and a suspension of (*N*-isocyanimino) triphenylphosphorane (182 mg, 0.6 mmol, 6 eq) in THF (3 mL) added, followed by 1,3,5-benzenetricarboxylic acid (21.0 mg, 0.1 mmol, 1 eq) as a single portion. The vial was recapped and stirred at rt for 80 mins. After this time CH_2Cl_2 (10 mL) was added and the reaction washed with saturated aqueous $NaHCO_3$ (20 mL), then the aqueous was extracted with CH_2Cl_2 (3 x 10 mL). The combined organics were dried over $MgSO_4$and then concentrated *in vacuo* to afford the crude product. After FCC (EtOAc —> 10% MeOH/CH_2Cl_2), **47** (36.6 mg, 0.064 mmol, 64%) was afforded as a tan powder.

m.p.: 154 – 156 °C

^{1}H NMR (400 MHz, $CDCl_3$) δ 8.91 (s, 3H), 3.90 (s, 6H), 2.59 (t, *J* = 5.3, 12H), 1.64 (p, *J* = 5.5, 12H), 1.44 (m, 6H).

^{13}C NMR (101 MHz, $CDCl_3$) δ 164.5, 163.5, 127.6, 126.1, 54.3, 52.7, 25.7, 23.7.

FT-IR(thin film): ν_{max}(cm^{-1}) = 2980, 2935, 2889, 1713, 1605, 1574, 1544, 1463, 1438, 1381, 1300, 1251, 1154, 1110, 1072, 961, 803, 733, 680.

(ESI): m/z calculated for $C_{30}H_{40}O_3N_9$ requires 574.3249 for $[M+H]^+$, found 574.3248.

2-(2-chloro-5,11-dimethyl-6,11-dihydro-5H-benzo[e]pyrimido[5,4-b][1,4]diazepin-6-yl)-5-((3,5-dichloropyridin-4-yl)methyl)-1,3,4-oxadiazole - 48

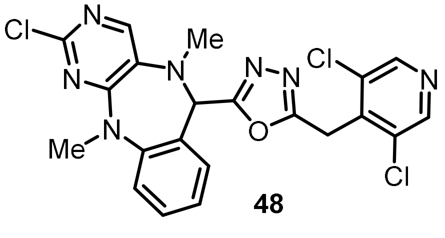

48

Following **general procedure A** (0.07 mmol scale, using 5 mol% Vaska's complex, and stirring for 1 h after addition of 2 eq of acid and NIITP): after PTLC (90% EtOAc/Hexane), **48** (14.7 mg, 0.030 mmol, 43%) was afforded as an beige crystalline solid.

m.p.: 98 – 100 °C

^{1}H NMR (400 MHz, $CDCl_3$) δ 8.53 (s, 2H), 7.67 (s, 1H), 7.43 (ddd, *J* = 8.2, 7.4, 1.6, 1H), 7.24 (d, *J* = 1.6, 1H), 7.20 – 7.11 (m, 1H), 5.29 (s, 1H), 4.44 (s, 2H), 3.24 (s, 3H), 2.98 (s, 3H).

^{13}C NMR (101 MHz, $CDCl_3$) δ 165.0, 162.2, 154.4, 151.1, 147.9, 146.4, 145.2, 138.3, 133.0, 131.2, 130.4, 129.4, 128.9, 124.5, 123.1, 64.0, 43.3, 40.0, 27.0.

FT-IR(thin film): ν_{max}(cm^{-1}) = 2980, 2917, 1556, 1532, 1497, 1476, 1448, 1403, 1344, 1241, 1204, 1174, 1141, 1119, 1095, 1038, 949, 887, 798, 767, 729, 706, 668.

(ESI): m/z calculated for $C_{21}H_{17}{}^{35}Cl_3ON_7$ requires 488.0555 for $[M+H]^+$, found 488.0554.

(Z)-3-((5-(1-(diethylamino)-2-(naphthalen-1-yloxy)propyl)-1,3,4-oxadiazol-2-yl)methylene)indolin-2-one - **49**

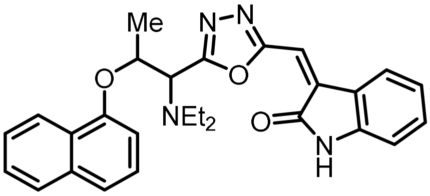

49

Following **general procedure A** (using 1 mol% Vaska's complex, and stirring for 16 h after addition of 2 eq of acid and NIITP): after FCC (60% Et_2O/Pentane), **49** (50.1 mg, 0.106 mmol, 53%) as a 2.4:1 mixture of diastereomers was afforded as an orange crystalline solid.

m.p.: 74 – 76 °C

The integral ratios are given as observed - ^1H NMR (400 MHz, $CDCl_3$) δ 9.04 – 8.98 (m, 0.4H), 8.92 – 8.85 (m, 1H), 8.72 (s, 0.4H), 8.63 (s, 1H), 8.29 – 8.22 (m, 0.4H), 7.93 (dd, *J* = 8.3, 1.4, 1H), 7.84 – 7.77 (m, 0.4H), 7.74 – 7.67 (m, 1H), 7.53 – 7.27 (m, 9H), 7.08 (td, *J* = 7.7, 1.1, 0.5H), 7.05 – 6.95 (m, 3H), 6.94 – 6.89 (m, 0.5H), 6.89 – 6.83 (m, 1H), 5.37 – 5.27 (m, 0.5H), 5.27 – 5.17 (m, 1H), 4.60 (d, *J* = 7.5, 0.4H), 4.54 (d, *J* = 9.5, 1H), 2.99 (dq, *J* = 13.2, 7.2, 0.9H), 2.89 (dq, *J* = 13.0, 7.3, 2.2H), 2.68 (dq, *J* = 13.8, 6.9, 0.9H), 2.45 (dq, *J* = 13.5, 6.8, 2.2H), 1.68 (d, *J* = 6.0, 3.2H), 1.53 (d, *J* = 6.1, 1.3H), 1.19 (t, *J* = 7.1, 6.4H), 1.09 (t, *J* = 7.1, 2.7H).

^{13}C NMR (101 MHz, $CDCl_3$) δ 169.2, 166.1, 165.9, 163.1, 162.9, 153.4, 153.1, 143.0, 142.7, 134.8, 134.6, 134.0, 133.6, 132.5, 132.3, 128.5, 128.4, 127.5, 127.4, 126.4, 126.4, 126.3, 126.3, 125.8, 125.7, 125.3, 125.2, 123.0, 122.9, 122.3, 121.6, 120.9, 120.7, 120.7, 112.2, 112.1, 110.2, 110.0, 106.8, 106.1, 74.2, 73.3, 61.6, 45.4, 45.1, 18.1, 17.5, 14.1, 13.9.

FT-IR(thin film): $\nu_{max}(cm^{-1})$ = 2657, 2980, 2888, 1712, 1611, 1462, 1382, 1251, 1154, 1072, 953, 815, 794, 772, 734, 677, 607.

(ESI): m/z calculated for $C_{28}H_{29}O_3N_4$ requires 469.2234 for $[M+H]^+$, found 469.2234.

2-((2,3-dihydrobenzo[b][1,4]dioxin-6-yl)(piperidin-1-yl)methyl)-5-(3-fluoro-4-(trifluoromethyl)phenyl)-1,3,4-oxadiazole - **50**

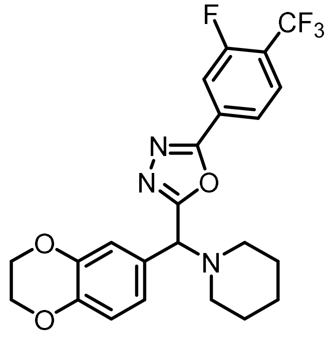

50

Following **general procedure A** (using 1 mol% Vaska's complex, and stirring for 1 h after addition of 1.1 eq of acid and NIITP): after FCC (25% Et_2O/Pentane), **50** (80.6 mg, 0.174 mmol, 87%) was afforded as an orange oil.

^1H NMR (400 MHz, $CDCl_3$) δ 7.98 – 7.92 (m, 1H), 7.90 – 7.85 (m, 1H), 7.74 (t, J = 7.6, 1H), 7.06 (d, J = 2.2, 1H), 6.96 (dd, J = 8.4, 2.2, 1H), 6.84 (d, J = 8.3, 1H), 4.82 (s, 1H), 4.24 (s, 4H), 2.58 – 2.47 (m, 2H), 2.39 – 2.28 (m, 2H), 1.65 – 1.54 (m, 4H), 1.48 – 1.39 (m, 2H).

^{19}F NMR (377 MHz, $CDCl_3$) δ -61.70 (d, J = 12.9, 3F), -112.17 (q, J = 13.1, 1F).

^{13}C NMR (101 MHz, $CDCl_3$) δ 167.0, 163.0 (d, J = 3.1), 159.9 (dq, J = 259.8, 2.1), 143.73, 143.66, 129.4 (d, J = 8.7), 129.2, 128.2 – 128.0 (m), 122.6 (d, J = 4.0), 121.5, 117.4, 117.3, 115.4 (d, J = 23.6), 66.4, 64.34, 64.32, 52.4, 25.9, 24.2.

FT-IR(thin film): $\nu_{max}(cm^{-1})$ = 2980, 2888, 1633, 1591, 1563, 1505, 1472, 1416, 132, 1284, 1255, 1130, 1068, 1047, 955, 887, 734.

(ESI): m/z calculated for $C_{23}H_{22}O_3N_3F_4$ requires 464.1592 for $[M+H]^+$, found 464.1590.

N-(3-(10,11-dihydro-5H-dibenzo[a,d][7]annulen-5-ylidene)propyl)-1-(5-(2-fluorophenyl)-1,3,4-oxadiazol-2-yl)-N-methyl-3-phenylpropan-1-amine - **51**

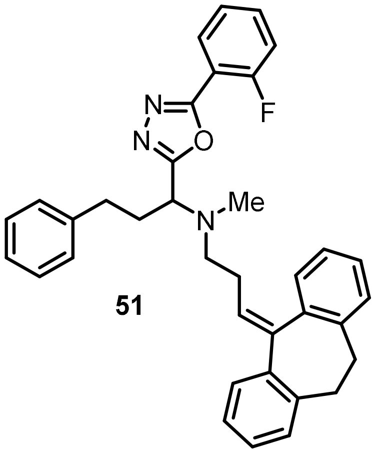

51

Following **general procedure A** (using 1 mol% Vaska's complex, and stirring for 16 h after addition of 2 eq of acid and NIITP): after FCC (12% EtOAc/Pentane), **51** (64.1 mg, 0.118 mmol, 59%) was afforded as a yellow oil.

^{1}H NMR (400 MHz, $CDCl_3$) δ 7.87 (td, *J* = 7.5, 1.8, 1H), 7.40 – 7.32 (m, 1H), 7.15 – 7.05 (m, 5H), 7.04 – 6.93 (m, 9H), 6.90 – 6.85 (m, 1H), 5.74 (t, *J* = 7.3, 1H), 3.82 (t, *J* = 7.6, 1H), 3.26 (br, 1H), 3.13 (br, 1H), 2.88 – 2.71 (br, 1H), 2.69 – 2.45 (m, 4H,), 2.39 – 2.35 (m, 1H), 2.21 – 2.10 (m, 4H), 2.09 (s, 3H).

^{19}F NMR (377 MHz, $CDCl_3$) δ -110.2

^{13}C NMR (101 MHz, $CDCl_3$) δ 166.1, 161.67 (d, *J* = 4.8), 159.98 (d, *J* = 258.2), 143.7, 141.4, 141.1, 140.1, 139.4, 137.1, 133.47 (d, *J* = 8.6), 130.0, 129.8, 129.4, 128.6, 128.5, 128.3, 128.1, 127.4, 127.1, 126.0, 125.8, 124.66 (d, *J* = 3.7), 116.98 (d, *J* = 20.9), 112.53 (d, *J* = 11.6), 58.9, 54.2, 53.6, 37.9, 37.2, 33.8, 32.3, 32.1, 32.0, 28.3.

FT-IR(thin film): ν_{max}(cm^{-1}) = 2980, 1621, 1589, 1543, 1494, 1472, 1462, 1362, 1265, 1225, 1157, 1111, 1065, 964, 822, 766, 755, 735, 699.

(ESI): m/z calculated for $C_{36}H_{35}ON_3F$ requires 544.2759 for $[M+H]^+$, found 544.2750.

2-(1-(4-(benzo[d][1,3]dioxol-5-ylmethyl)piperazin-1-yl)-2-(4-chlorophenoxy)ethyl)-5-(5-chloro-2-(methylthio)pyrimidin-4-yl)-1,3,4-oxadiazole - 52

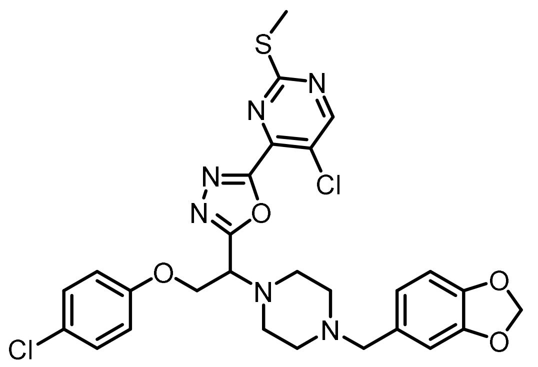

52

Following **general procedure A** (0.19 mmol scale, using 1 mol% Vaska's complex, and stirring for 16 h after addition of 2 eq of acid and NIITP): after FCC (45% EtOAc/Pentane), **52** (77.8 mg, 0.132 mmol, 69%) was afforded as a golden crystalline solid.

m.p.: 54 – 56 °C

^{1}H NMR (400 MHz, $CDCl_3$) δ 8.71 (s, 1H), 7.25 – 7.19 (m, 2H), 6.86 – 6.80 (m, 2H), 6.79 (d, *J* = 1.4, 1H), 6.71 (dd, *J* = 4.0, 1.0, 2H), 5.92 (s, 2H), 4.59 (dd, *J* = 8.5, 7.8, 1H), 4.53 (dd, *J* = 7.8, 5.1, 1H), 4.45 (dd, *J* = 8.5, 5.1, 1H), 3.39 (s, 2H), 2.81 – 2.72 (m, 2H), 2.72 – 2.64 (m, 2H), 2.59 (s, 3H), 2.47 (s, 4H).

^{13}C NMR (101 MHz, $CDCl_3$) δ 171.4, 165.0, 159.1, 156.7, 147.7, 146.8, 146.7, 131.7, 129.4, 126.5, 124.5, 122.2, 116.3, 109.4, 107.9, 100.9, 67.0, 62.6, 59.8, 53.0, 50.0, 14.6.

FT-IR(thin film): ν_{max}(cm^{-1}) = 2980, 2882, 1700, 1541, 1489, 1439, 1399, 1367, 1337, 1300, 1242, 1212, 1165, 1037, 1006, 933, 889, 885, 826, 803, 776, 729, 668.

(ESI): m/z calculated for $C_{27}H_{27}O_4N_6{}^{35}Cl_2S$ requires 601.1186 for $[M+H]^+$, found 601.1180.

4-(5-((2,3-dihydrobenzo[b][1,4]dioxin-6-yl)(piperidin-1-yl)methyl)-1,3,4-oxadiazol-2-yl)-N,N-dipropylbenzenesulfonamide - **53**

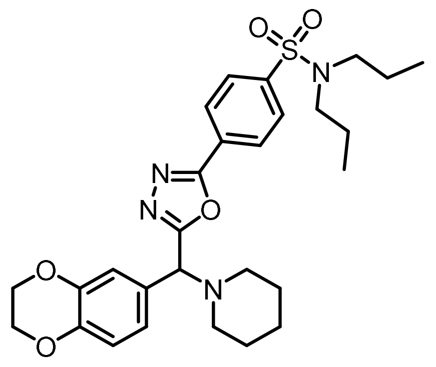

53

Following **general procedure A** (4 mmol scale, using 1 mol% Vaska's complex, and stirring for 1 h after addition of 1.1 eq of acid and NIITP): after FCC (50% Et_2O/Pentane), **53** (1.731 g, 3.20 mmol, 80%) was afforded as a white crystalline solid.

m.p.: 50 – 52 °C

^{1}H NMR (400 MHz, $CDCl_3$) δ 8.17 (d, *J* = 8.5, 2H), 7.92 (d, *J* = 8.5, 2H), 7.06 (d, *J* = 2.1, 1H), 6.97 (dd, *J* = 8.4, 2.1, 1H), 6.83 (d, *J* = 8.3, 1H), 4.81 (s, 1H), 4.24 (s, 4H), 3.13 – 3.06 (m, 4H), 2.51 (dt, *J* = 11.1, 5.3, 2H), 2.33 (dt, *J* = 10.3, 4.8, 2H), 1.64 – 1.48 (m, 8H), 1.44 (td, *J* = 6.7, 2.8, 2H), 0.87 (t, *J* = 7.4, 6H).

^{13}C NMR (101 MHz, $CDCl_3$) δ 166.7, 163.9, 143.7, 143.6, 143.1, 129.4, 127.6, 127.5, 127.3, 121.5, 117.4, 117.3, 66.4, 64.3, 52.4, 49.9, 25.9, 24.3, 21.9, 11.2.

FT-IR(thin film): ν_{max}(cm^{-1}) = 2933, 1699, 1505, 1455, 1381, 1339, 1283, 1257, 1212, 1155, 1091, 1066, 992, 980, 886, 838, 729, 699.

(ESI): m/z calculated for $C_{28}H_{37}O_5N_4S$ requires 541.2479 for $[M+H]^+$, found 541.2474

4-(5-((2,3-dihydrobenzo[b][1,4]dioxin-6-yl)(piperidin-1-yl)methyl)-4-tosyl-4H-1,2,4-triazol-3-yl)-N,N-dipropylbenzenesulfonamide - 54

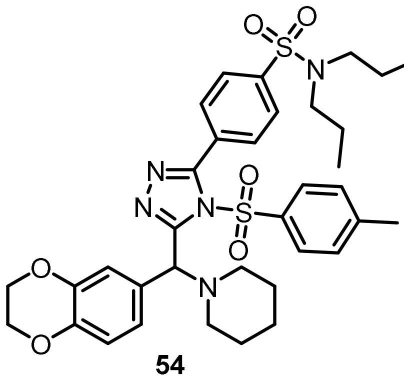

54

Following **general procedure A** (using 1 mol% Vaska's complex, and stirring for 16 h after addition of 2 eq of **55** and NIITP): after FCC (50% EtOAc/Pentane) and PLTC (50% EtOAc/Hexane), **54** (38.4 mg, 0.056 mmol, 28%) was afforded as a tan powder.

m.p.: 114 – 116 °C

^{1}H NMR (400 MHz, $CDCl_3$) δ 7.77 (d, *J* = 8.4, 2H), 7.42 (d, *J* = 8.4, 2H), 7.10 – 7.02 (m, 5H), 6.97 (dd, *J* = 8.4, 2.1, 1H), 6.83 (d, *J* = 8.3, 1H), 5.61 (s, 1H), 4.26 (s, 4H), 3.12 – 3.06 (m, 4H), 2.77 – 2.62 (m, 2H), 2.56 – 2.43 (m, 2H), 2.35 (s, 3H), 1.65 – 1.49 (m, 8H), 1.46 – 1.37 (m, 2H), 0.88 (t, *J* = 7.4 Hz, 6H).

^{13}C NMR (101 MHz, $CDCl_3$) δ 152.2, 147.0, 143.5, 143.2, 142.1, 134.2, 131.7, 130.8, 129.8, 127.8, 126.2, 123.5, 119.3, 116.8, 65.4, 64.4, 64.3, 51.8, 50.1, 26.2, 24.4, 22.0, 21.7, 11.2.

FT-IR(thin film): ν_{max}(cm^{-1}) = 2967, 2934, 2875, 1697, 1627, 1592, 1507, 1458, 1386, 1338, 1287, 1258, 1184, 1157, 1081, 1017, 992, 888, 813, 735, 701, 666.

(ESI): m/z calculated for $C_{35}H_{44}O_6N_5S_2$ requires 694.2728 for $[M+H]^+$, found 694.2720.

4-(5-((2,3-dihydrobenzo[b][1,4]dioxin-6-yl)(piperidin-1-yl)methyl)-1,3,4-thiadiazol-2-yl)-N,N-dipropylbenzenesulfonamide - 55

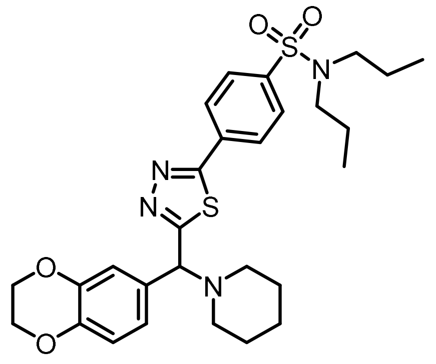

55

Following **general procedure A** (using 1 mol% Vaska's complex, and stirring for 1 h after addition of 2 eq of **56** and NIITP): after FCC (55% Et_2O/Pentane), **55** (70.8 mg, 0.124 mmol, 62%) was afforded as a orange crystalline solid.

m.p.: 60 – 62 °C

^{1}H NMR (400 MHz, $CDCl_3$) δ 8.06 (d, *J* = 8.5, 2H), 7.87 (d, *J* = 8.5, 2H), 6.98 (d, *J* = 2.1, 1H), 6.92 (dd, *J* = 8.4, 2.1, 1H), 6.82 (d, *J* = 8.3, 1H), 4.84 (s, 1H), 4.23 (s, 4H), 3.13 – 3.07 (m, 4H), 2.61 – 2.45 (m, 2H), 2.45 – 2.29 (m, 2H), 1.65 – 1.50 (m, 8H), 1.49 – 1.41 (m, 2H), 0.86 (t, *J* = 7.4, 6H).

^{13}C NMR (101 MHz, $CDCl_3$) δ 176.0, 167.8, 143.7, 143.5, 142.3, 133.9, 131.5, 128.3, 127.7, 121.5, 117.5, 117.2, 70.6, 64.3, 52.8, 49.9, 26.1, 24.3, 21.9, 11.2.

FT-IR(thin film): ν_{max}(cm^{-1}) = 2933, 1699, 1588, 1504, 1455, 1381, 1283, 1257, 1212, 1155, 1066, 992, 979, 923, 886, 776, 729, 669.

(ESI): m/z calculated for $C_{28}H_{37}O_4N_4S_2$ requires 557.2251 for $[M+H]^+$, found 557.2245.

5. NMR data

2-((1-(tert-butoxycarbonyl)piperidin-4-yl)oxy)-2-oxoacetic acid – S1

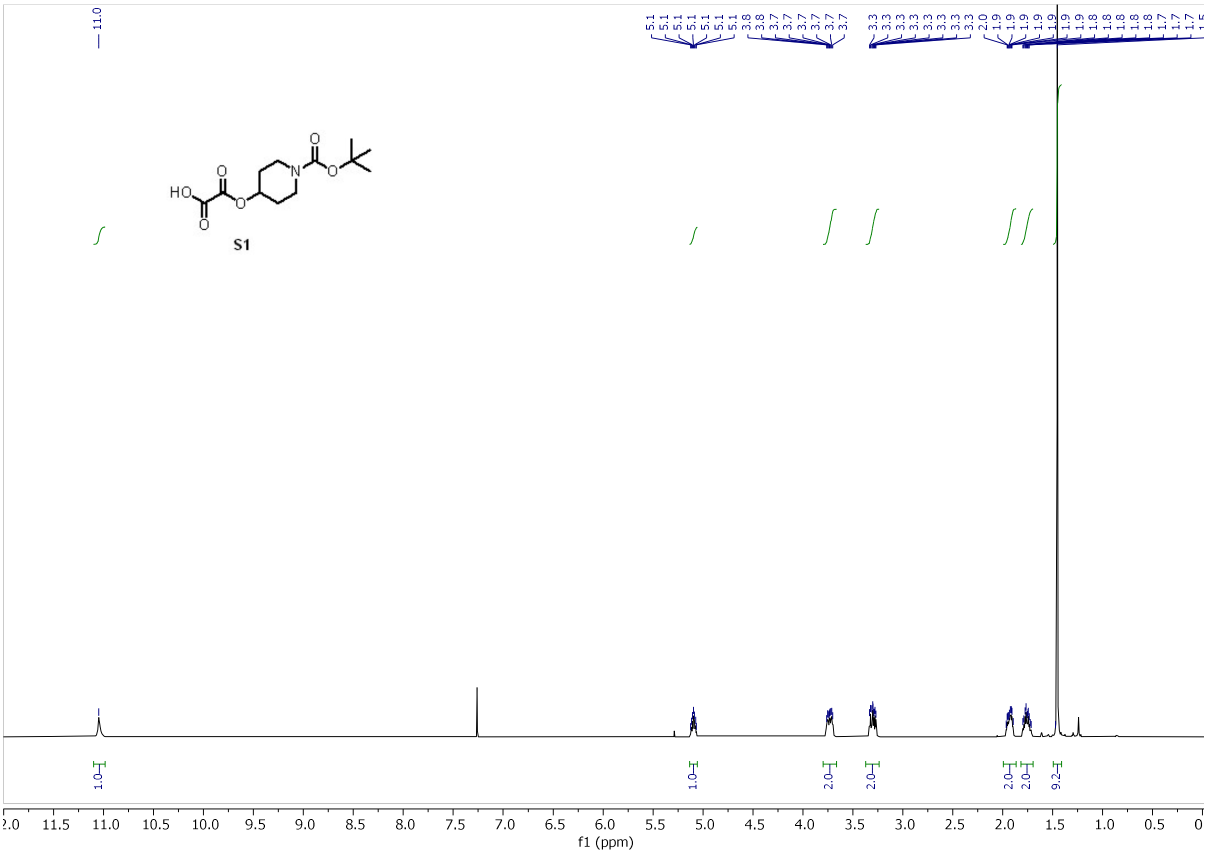

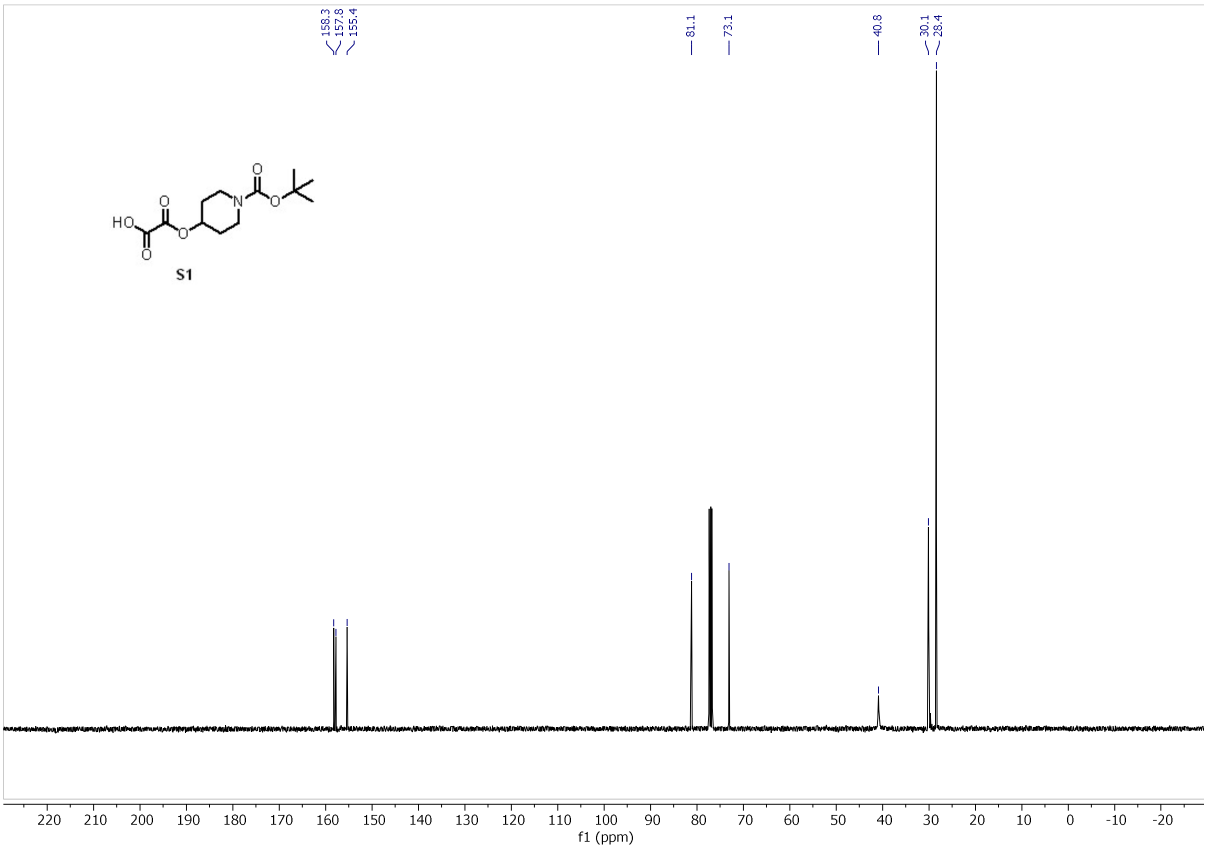

N-tosylbenzamide – S2

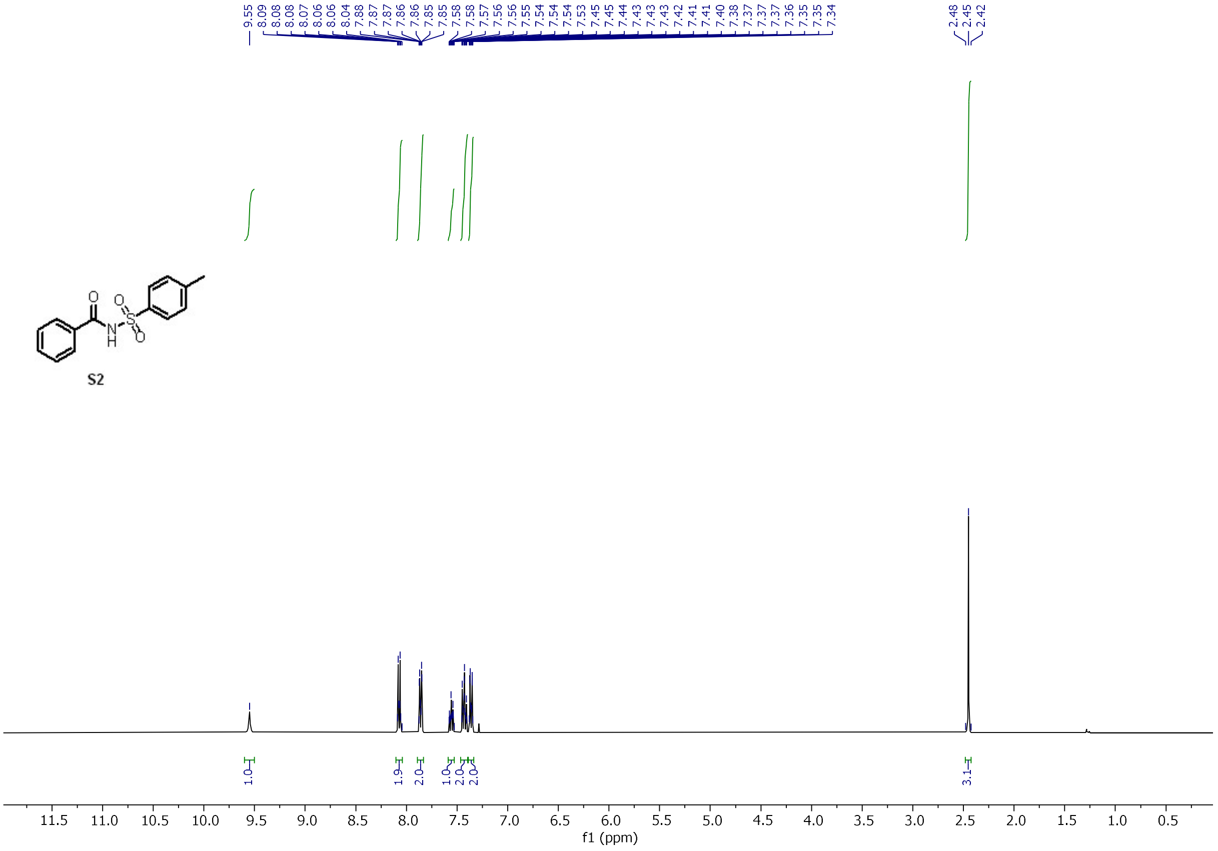

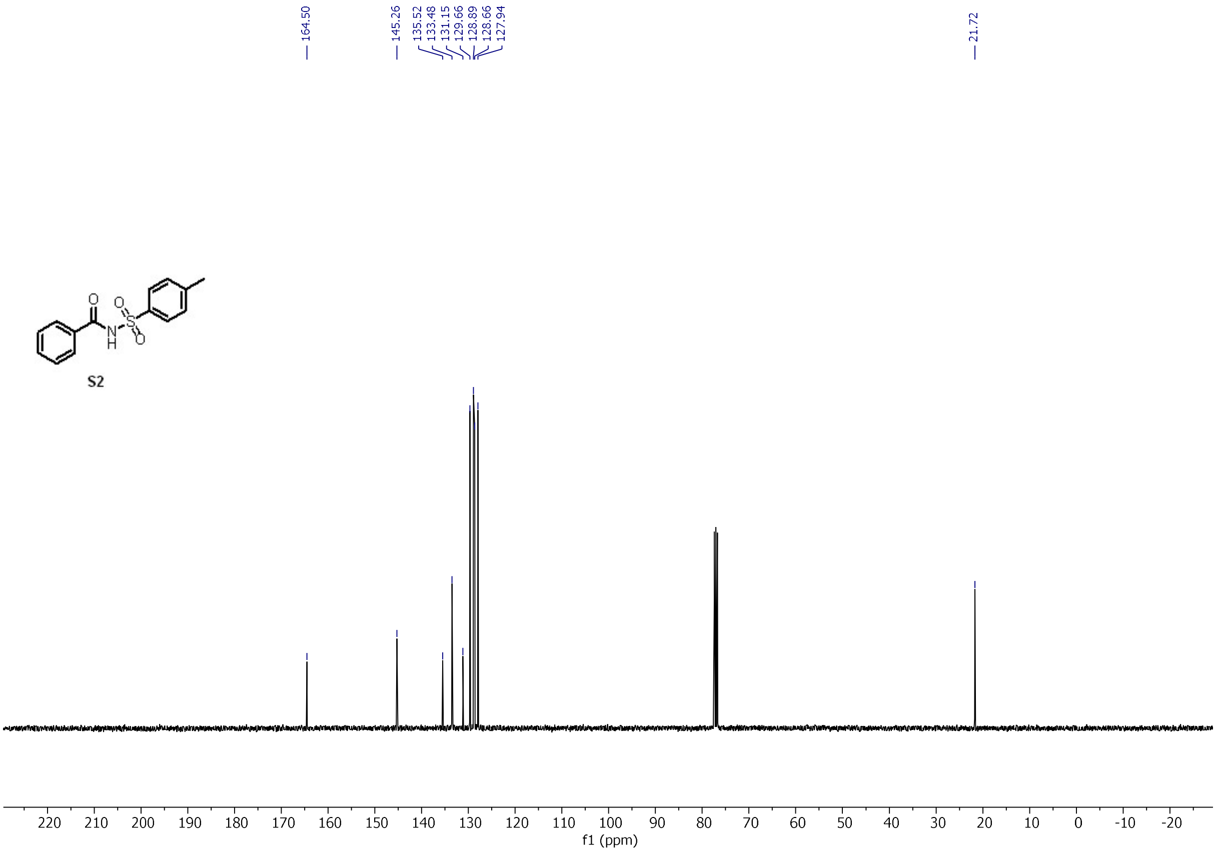

tert-butyl benzoylcarbamate – S3

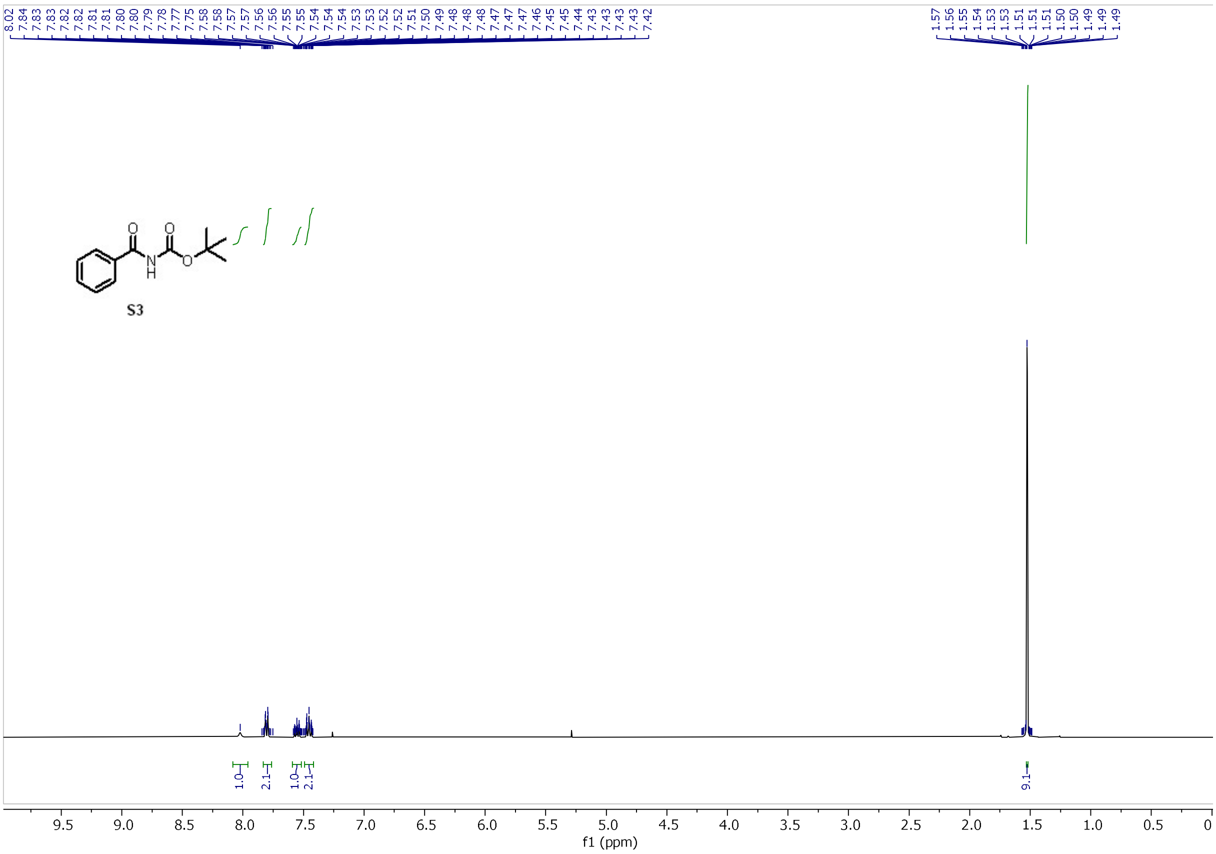

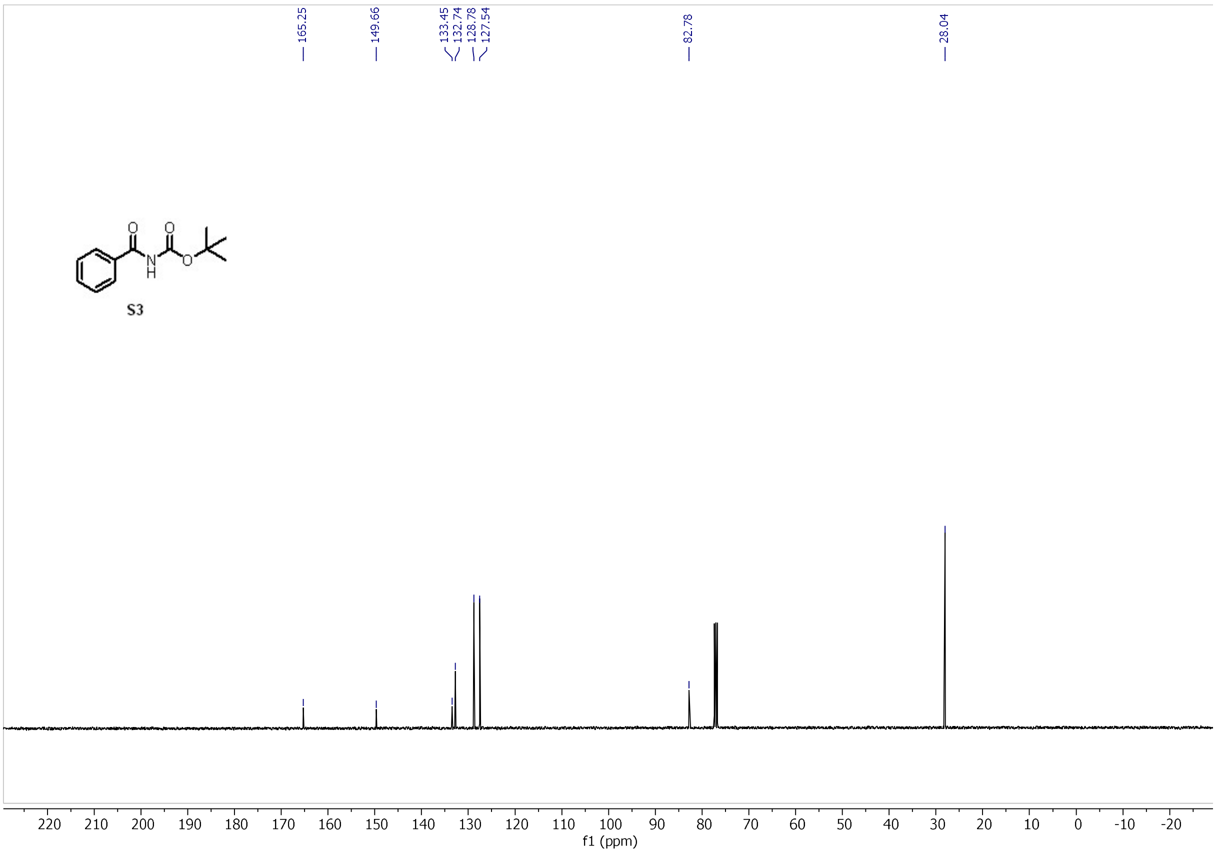

diethyl benzoylphosphoramidate – 26

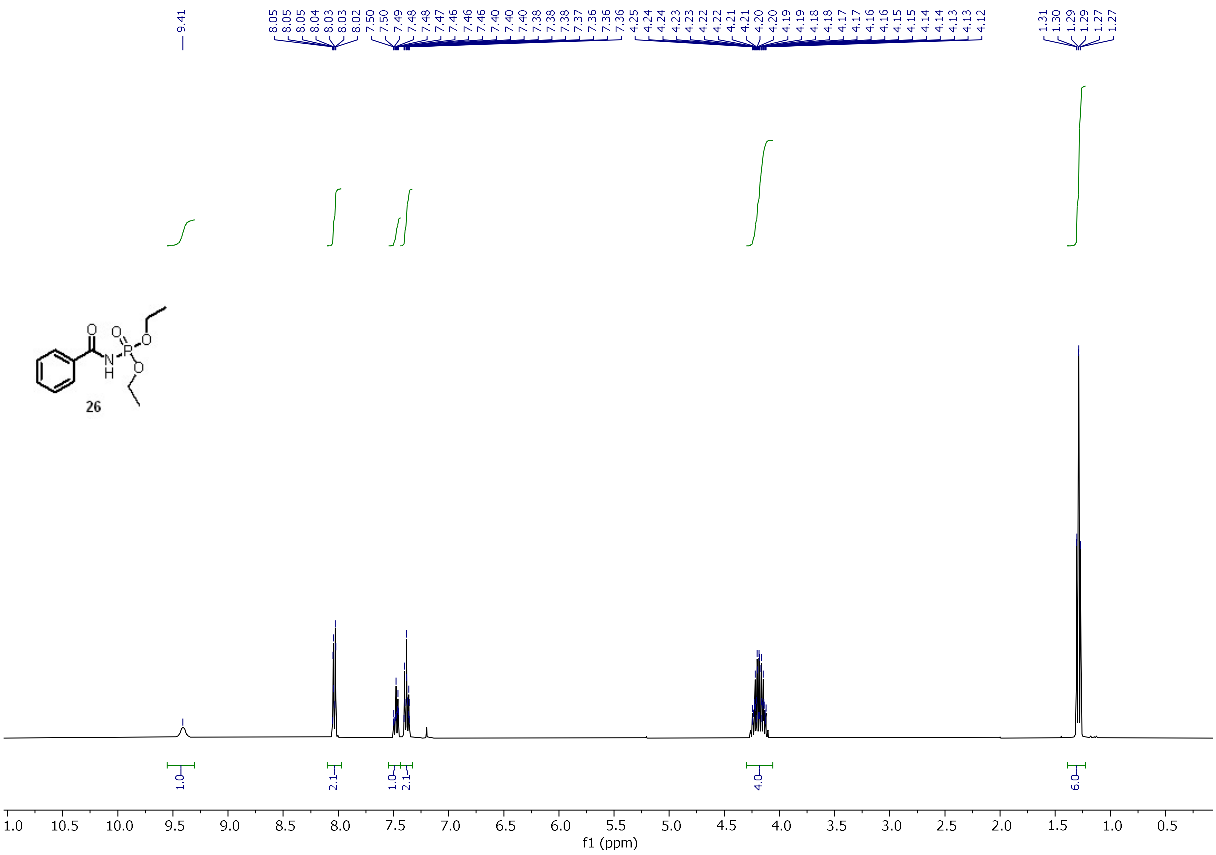

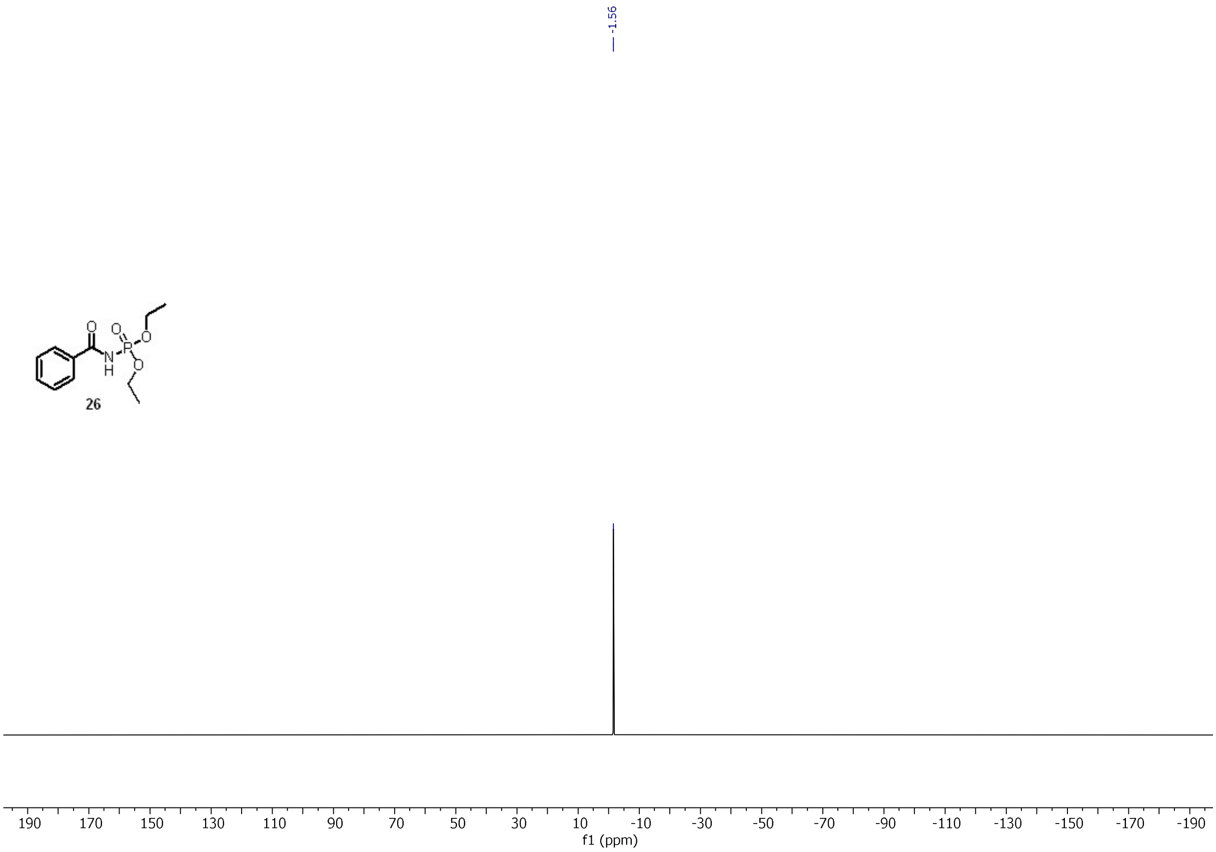

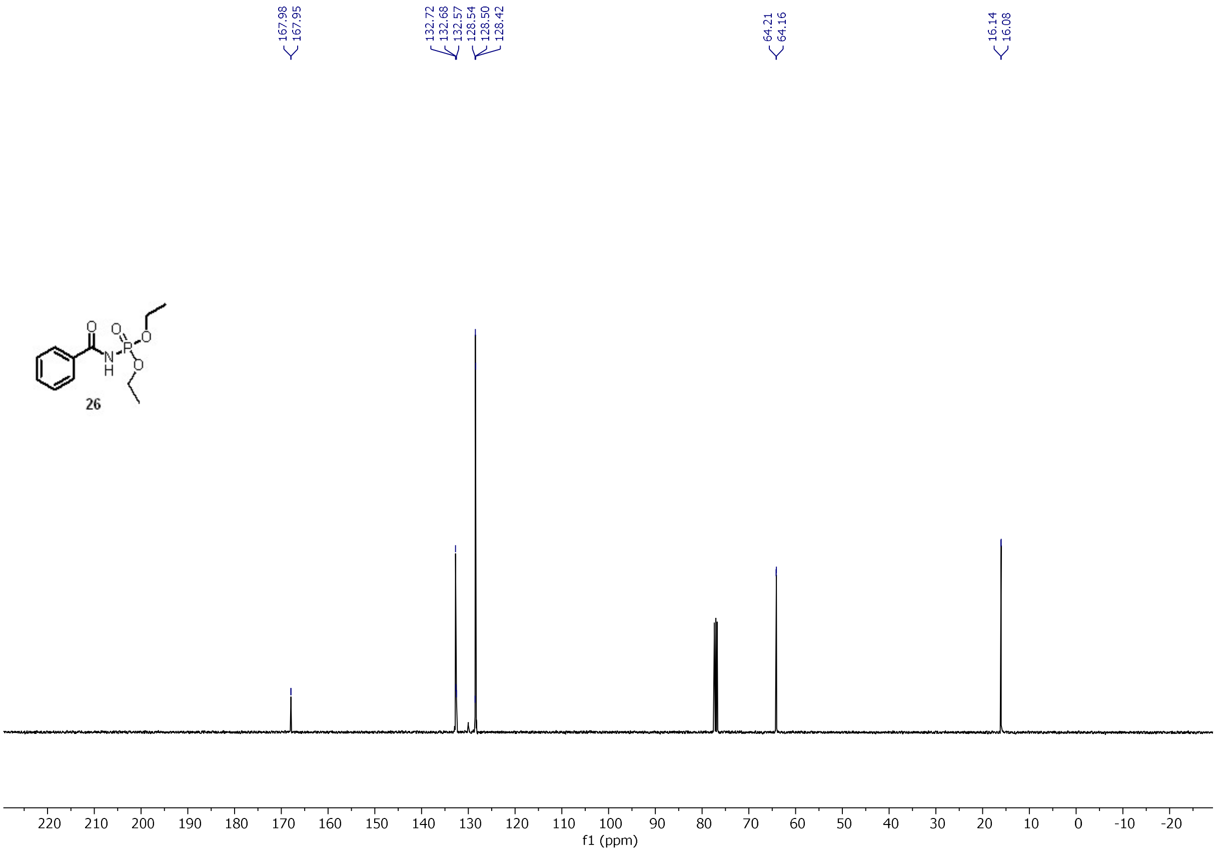

1-allylazocan-2-one – S4

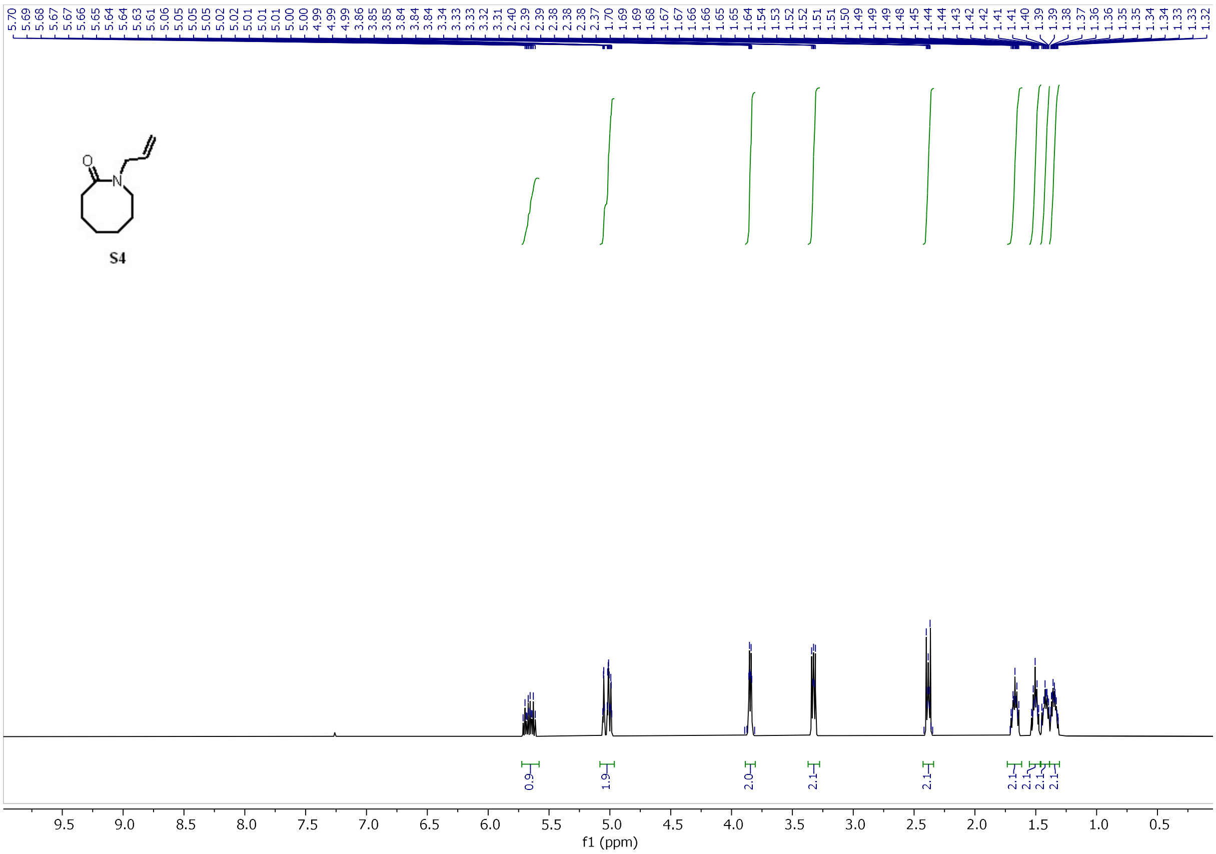

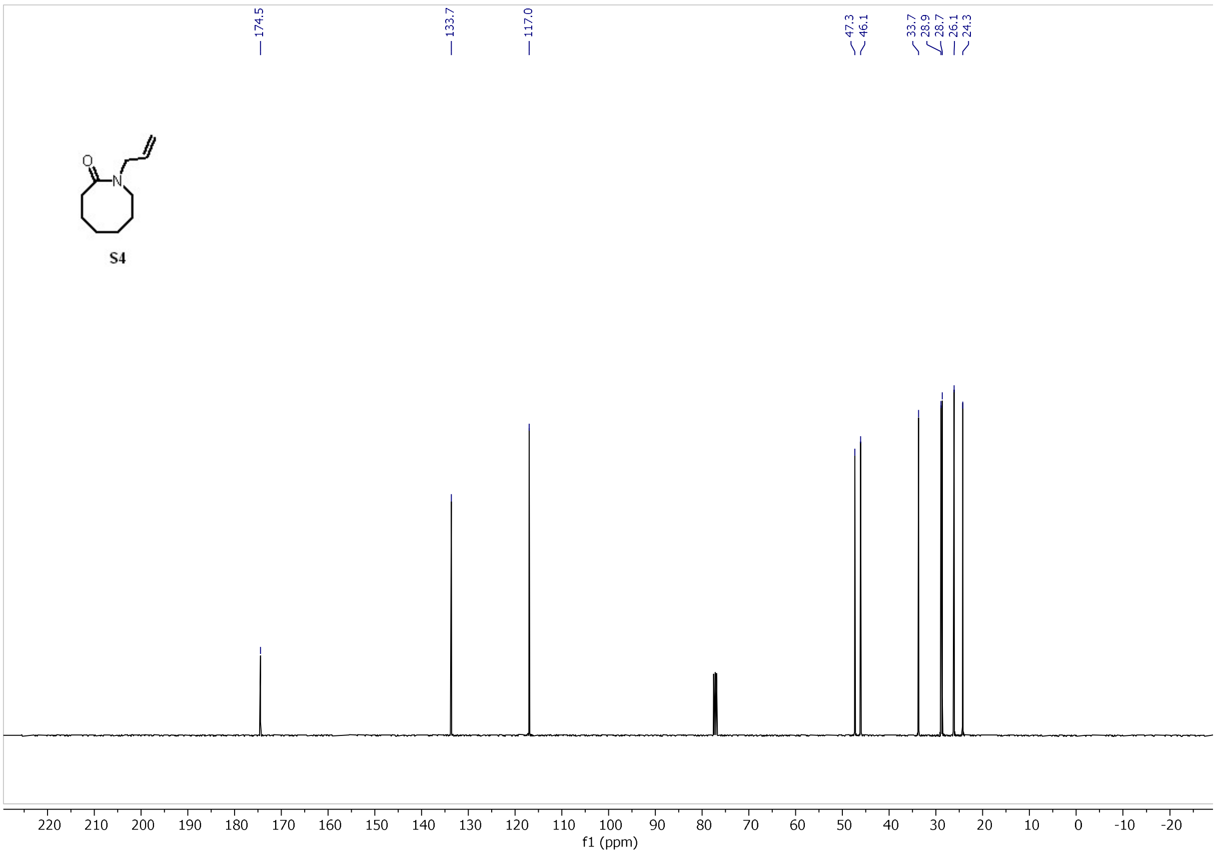

tert-butyl 4-(2-iodobenzoyl)piperazine-1-carboxylate– S5

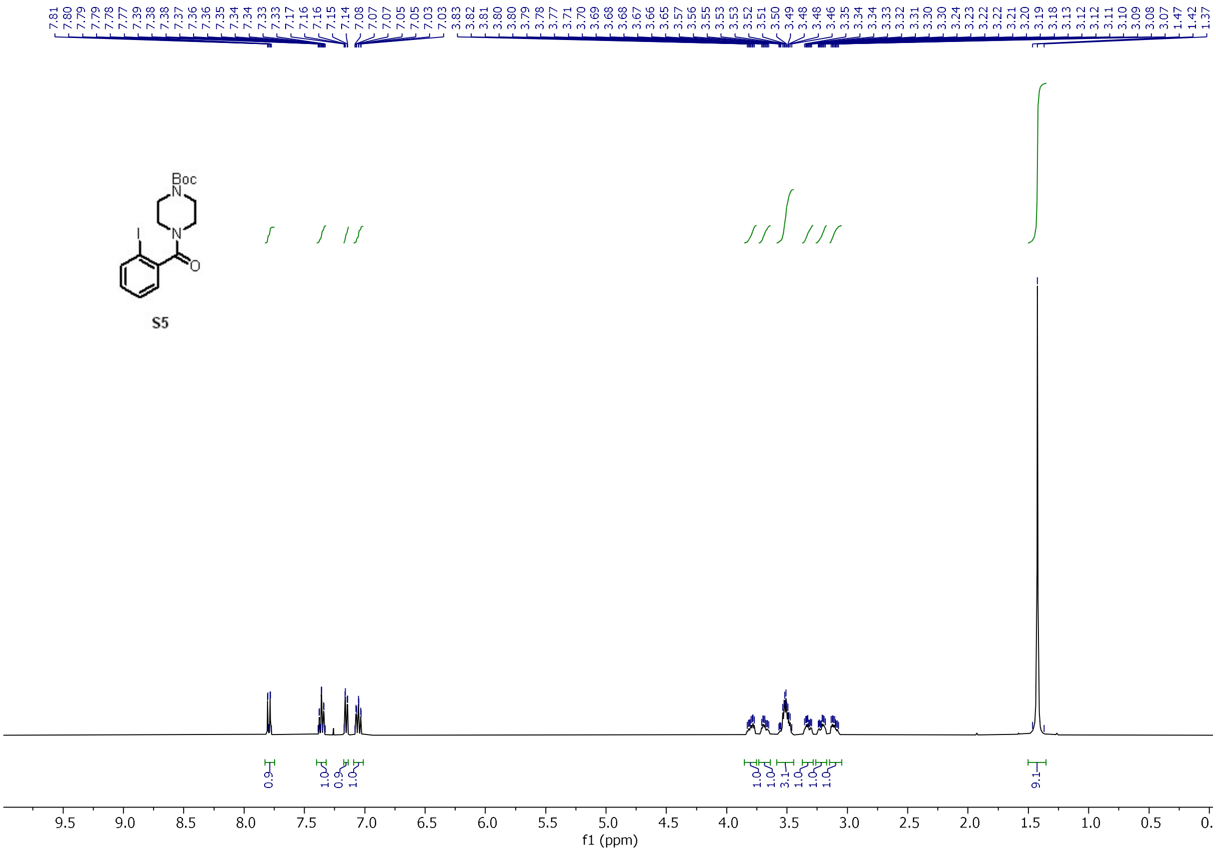

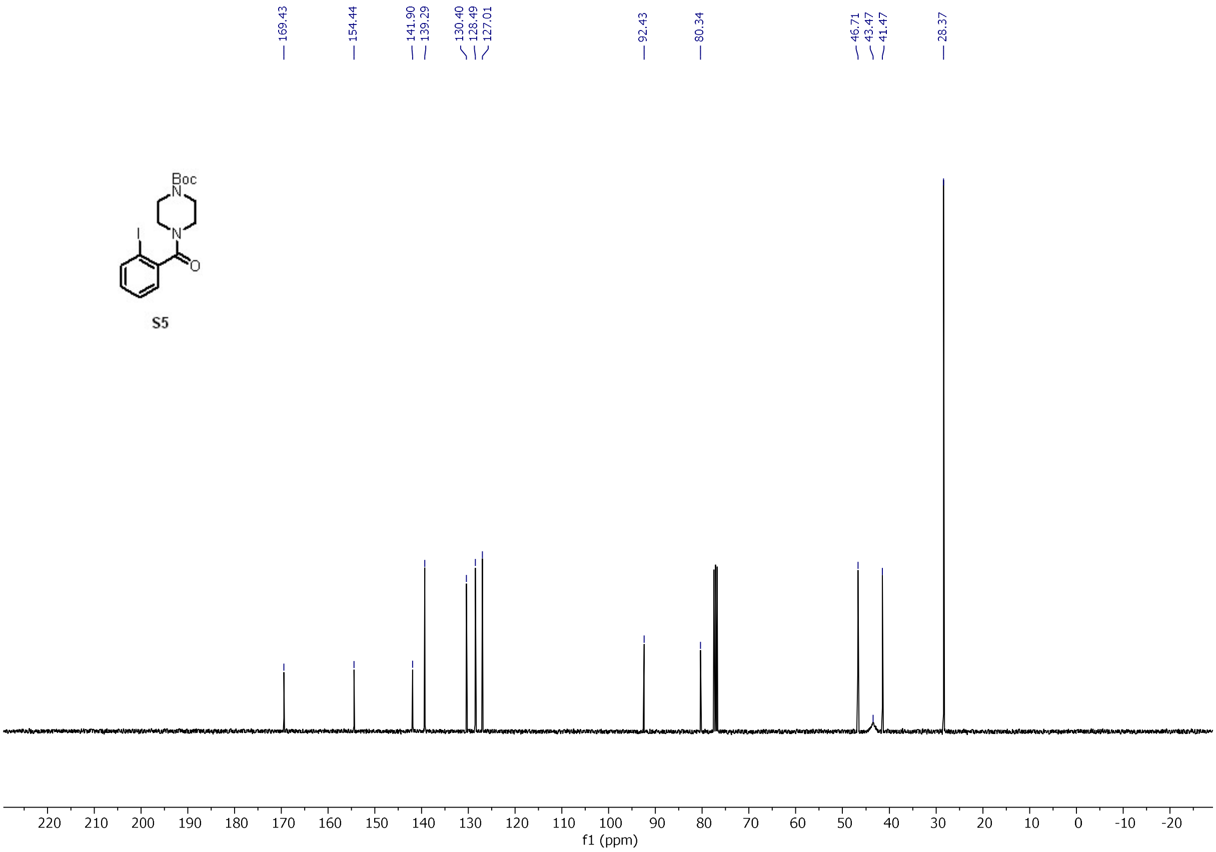

S5

N,N-dimethyl-9-oxo-9H-fluorene-4-carboxamide – S6

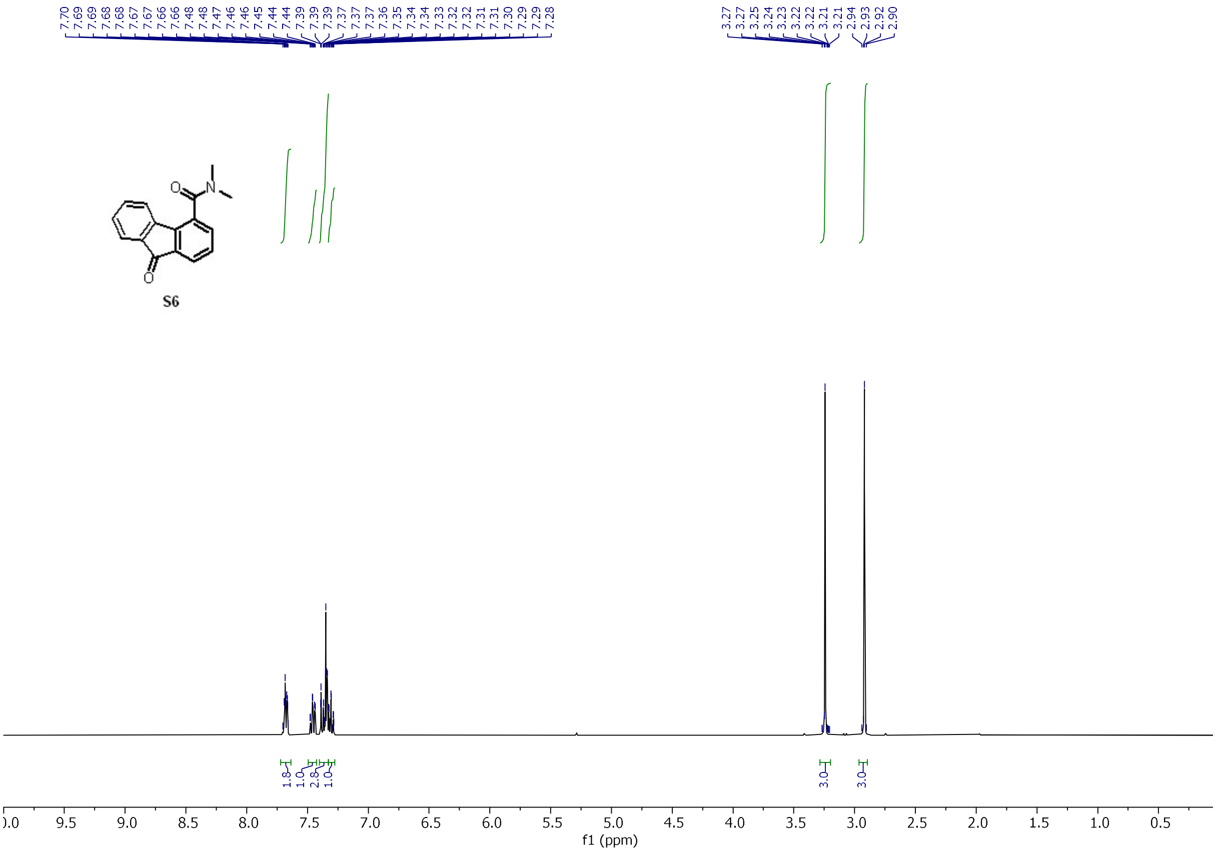

S6

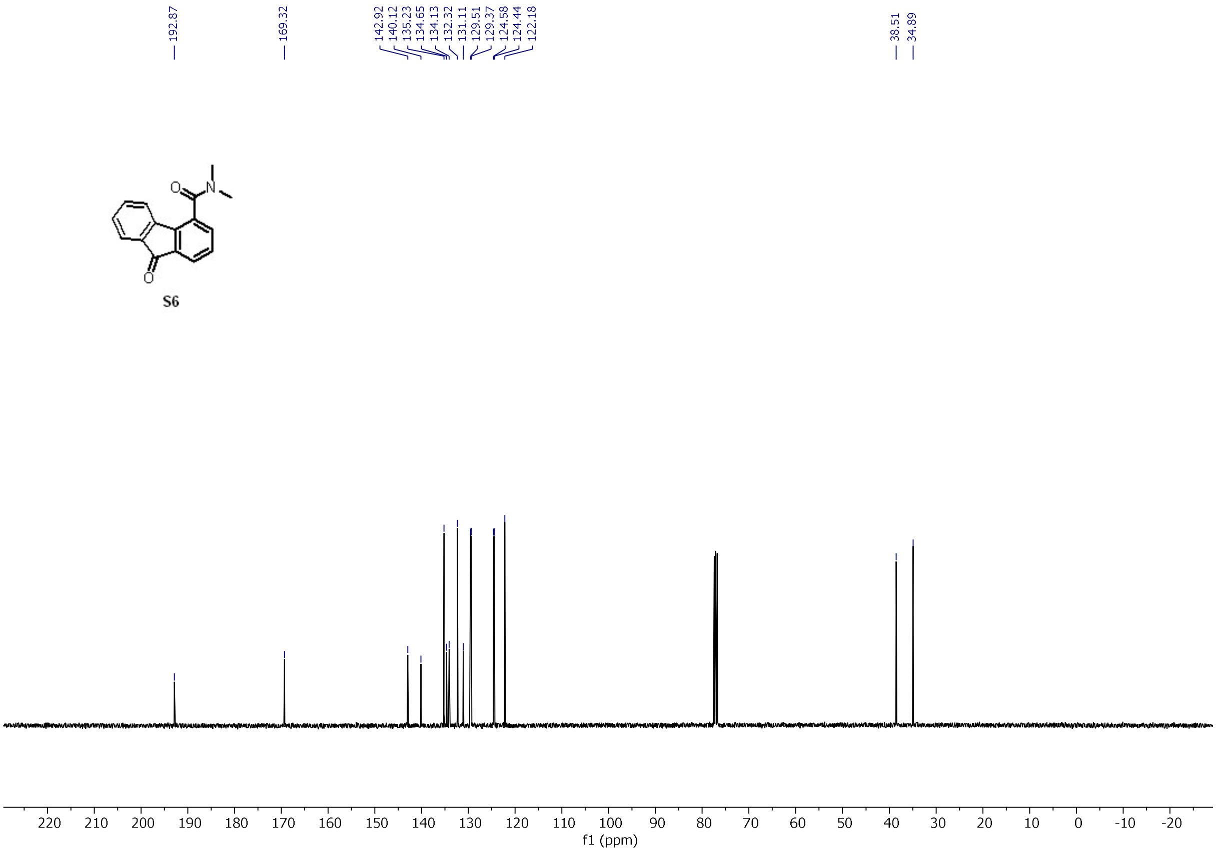

S6

4-(N,N-dipropylsulfamoyl)-N-tosylbenzamide – 56

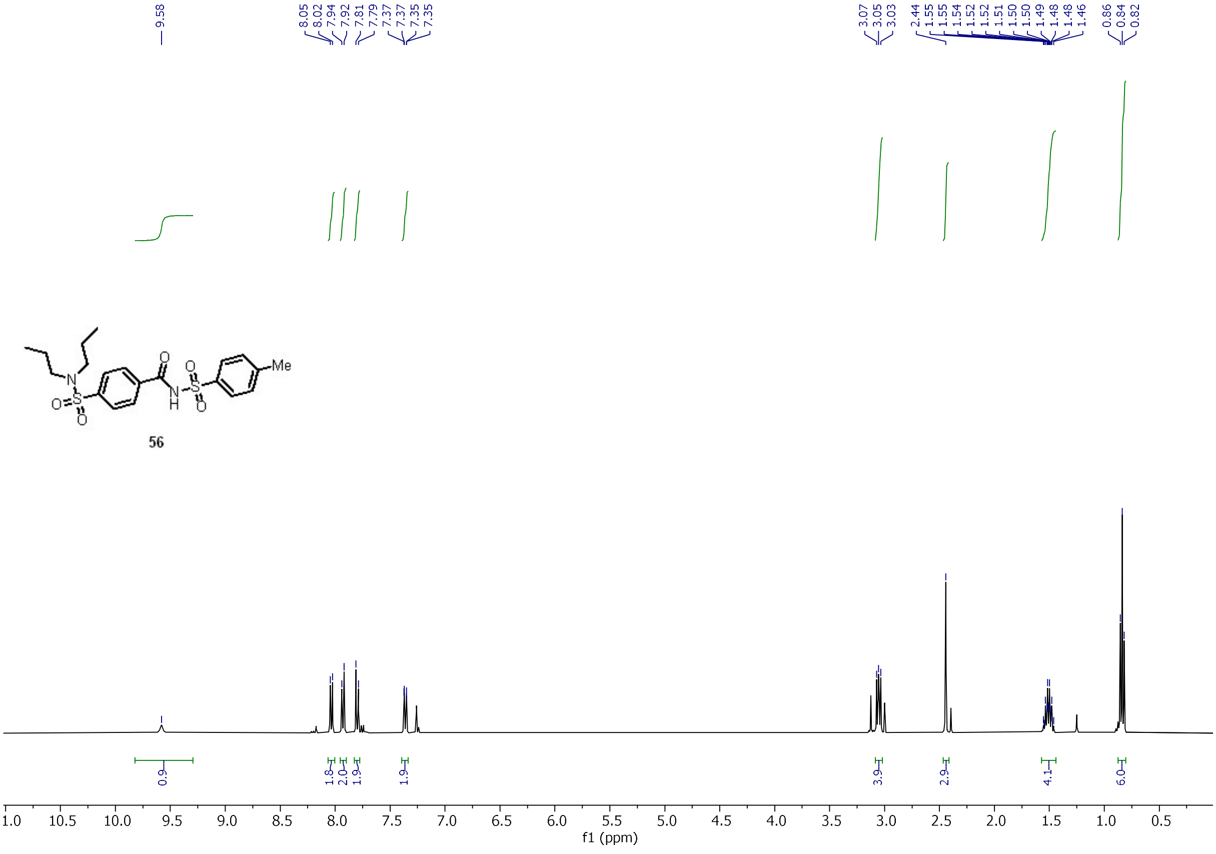

56

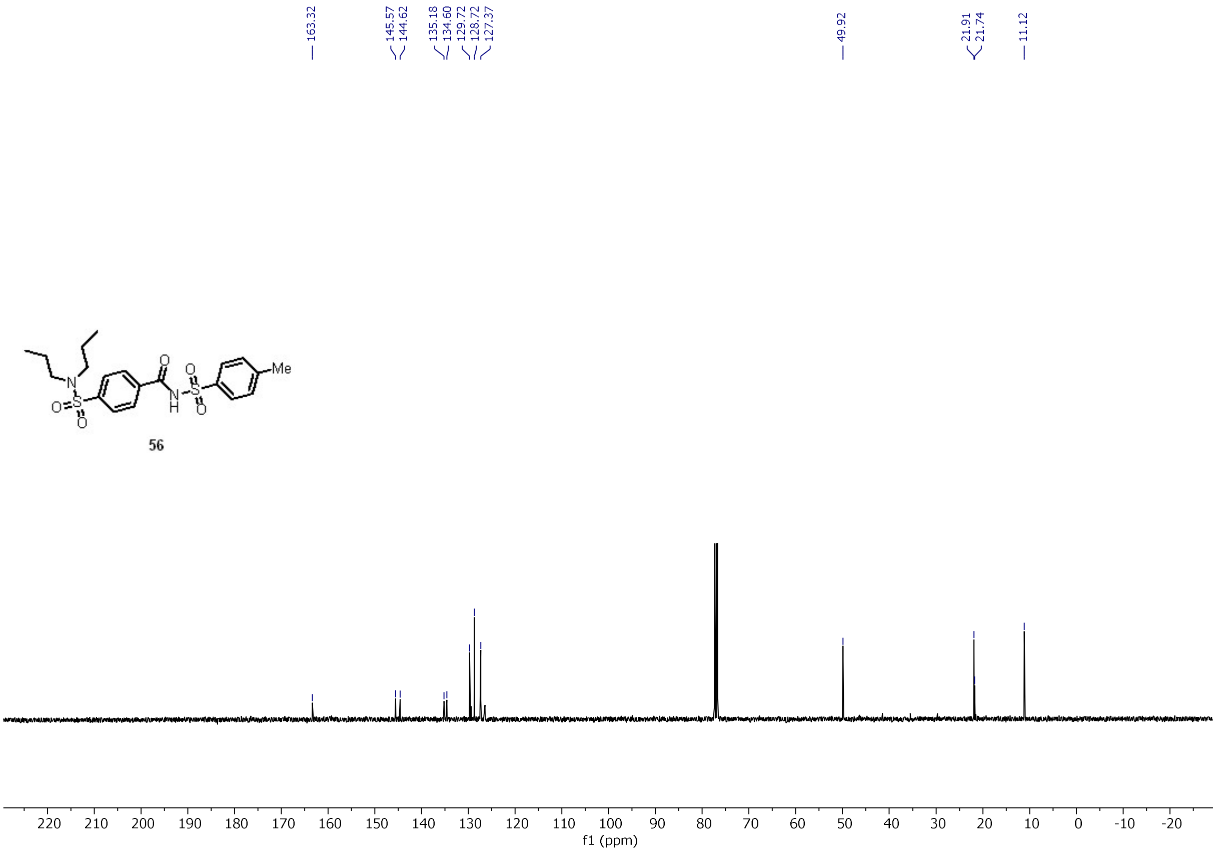

4-(N,N-dipropylsulfamoyl)benzothioic S-acid - 57

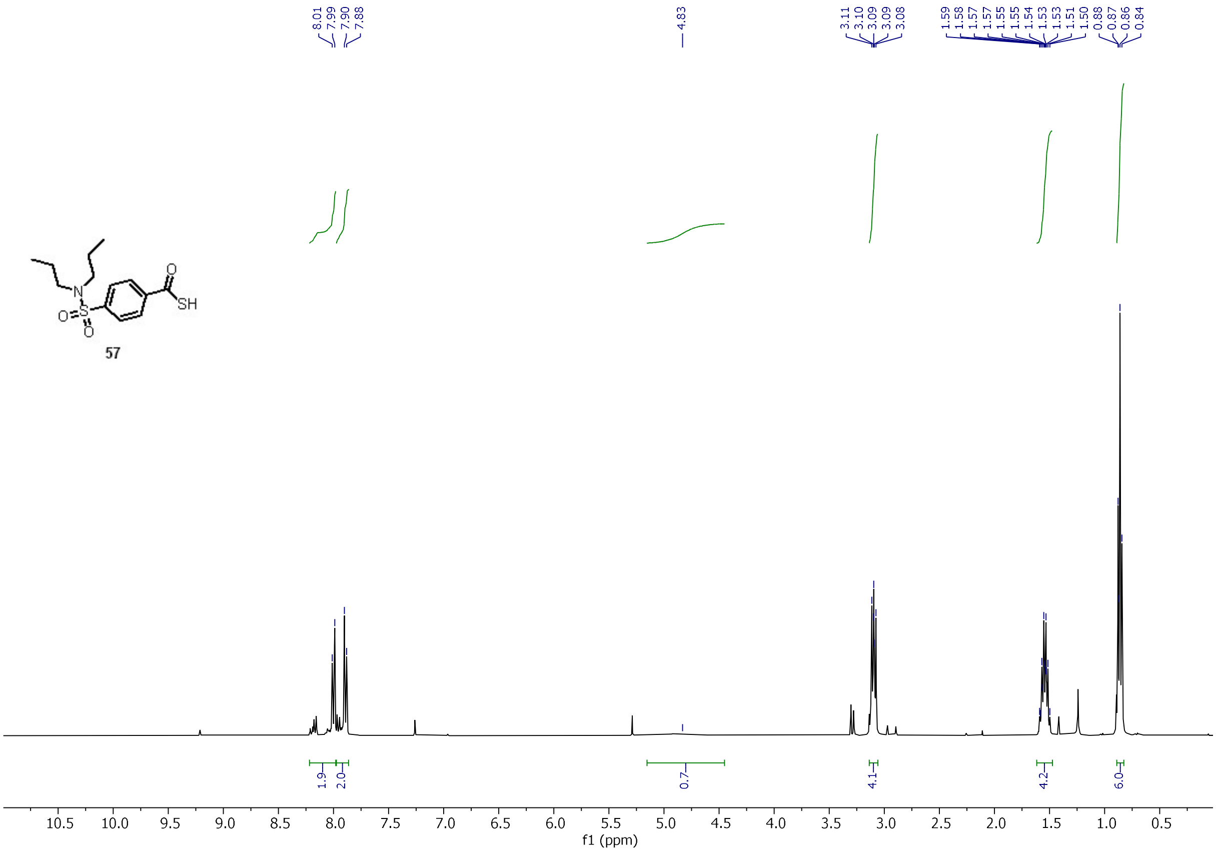

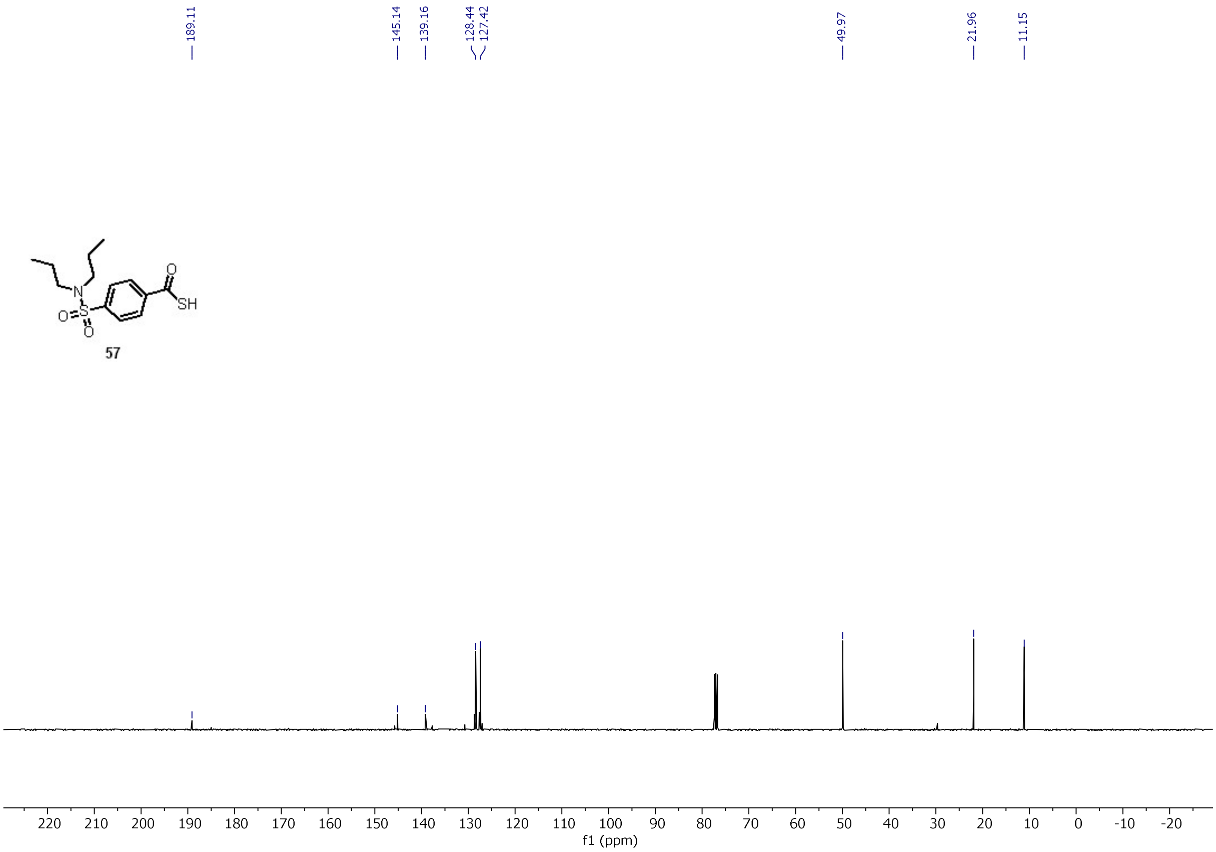

2-(1-benzylpiperidin-2-yl)-5-phenyl-1,3,4-oxadiazole – 2

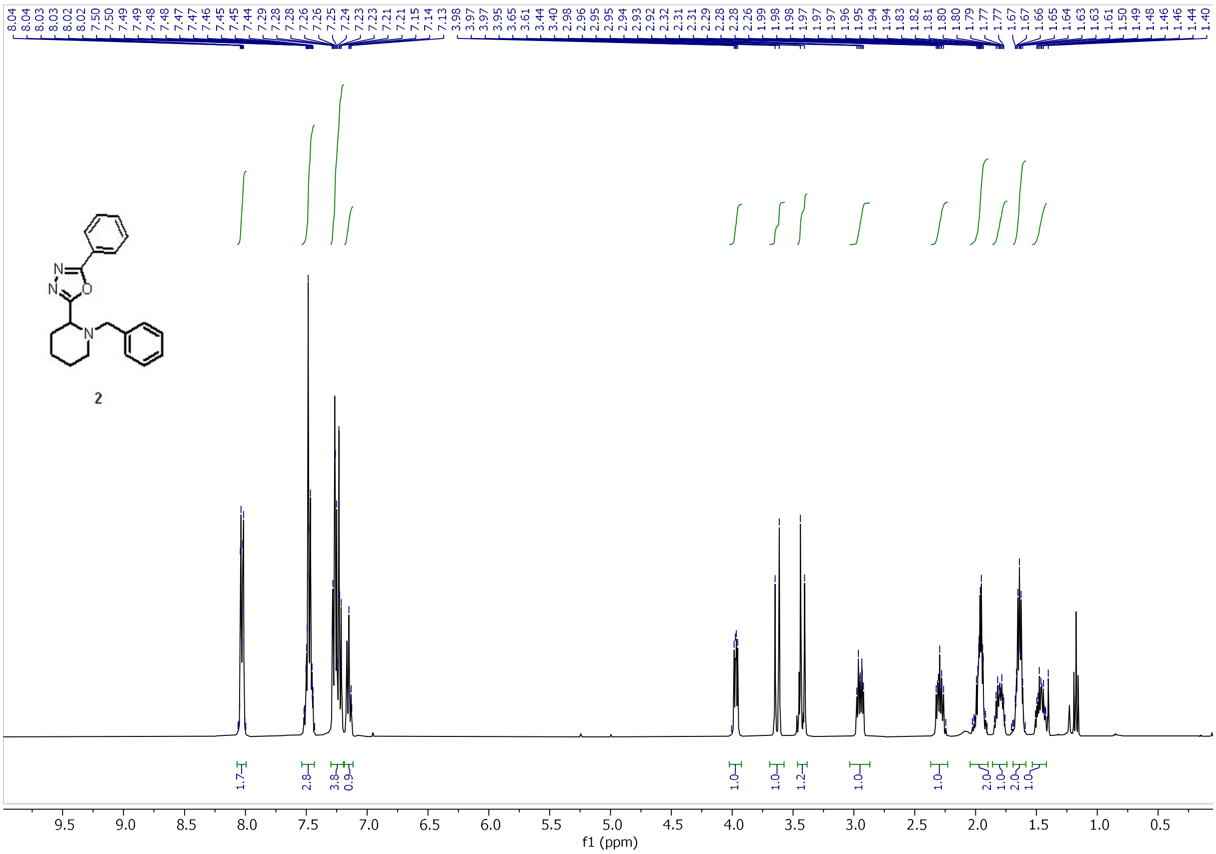

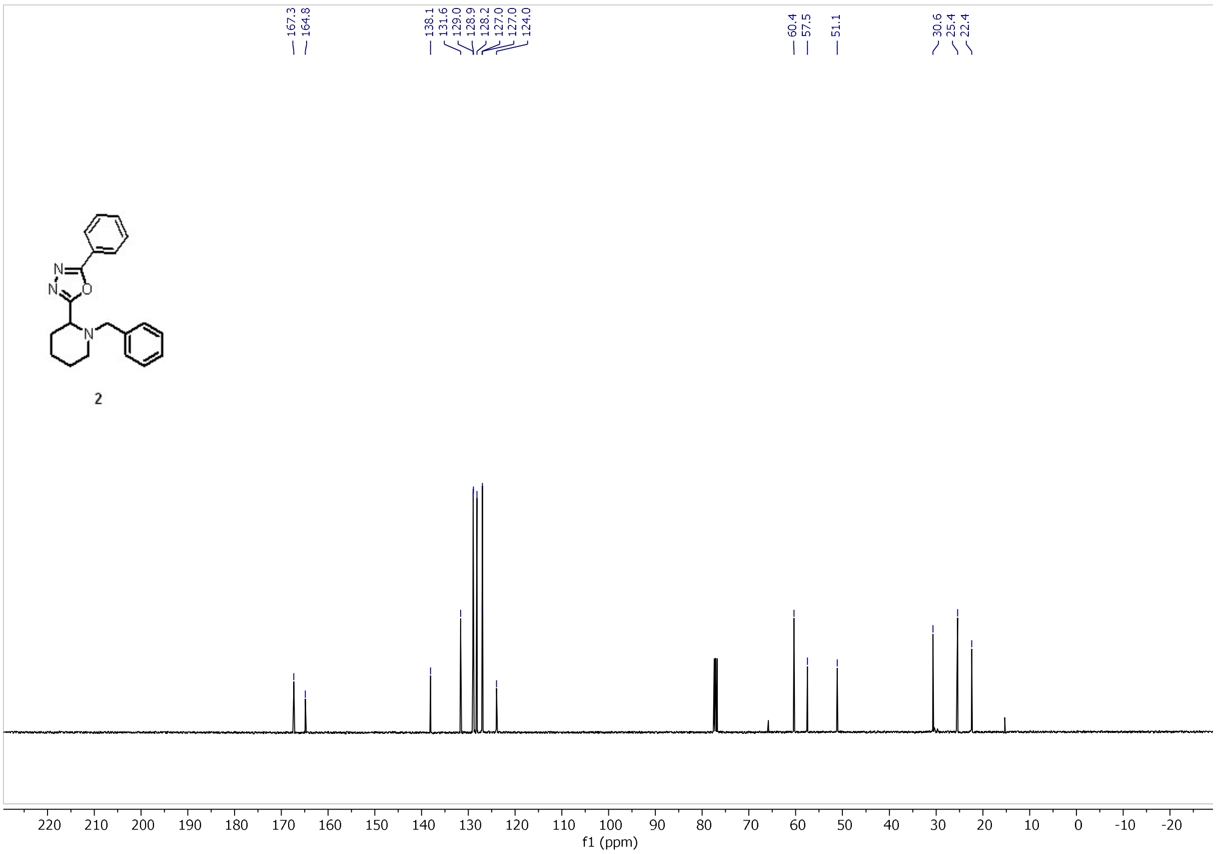

2-(1-benzylpiperidin-2-yl)-5-methyl-1,3,4-oxadiazole - 4

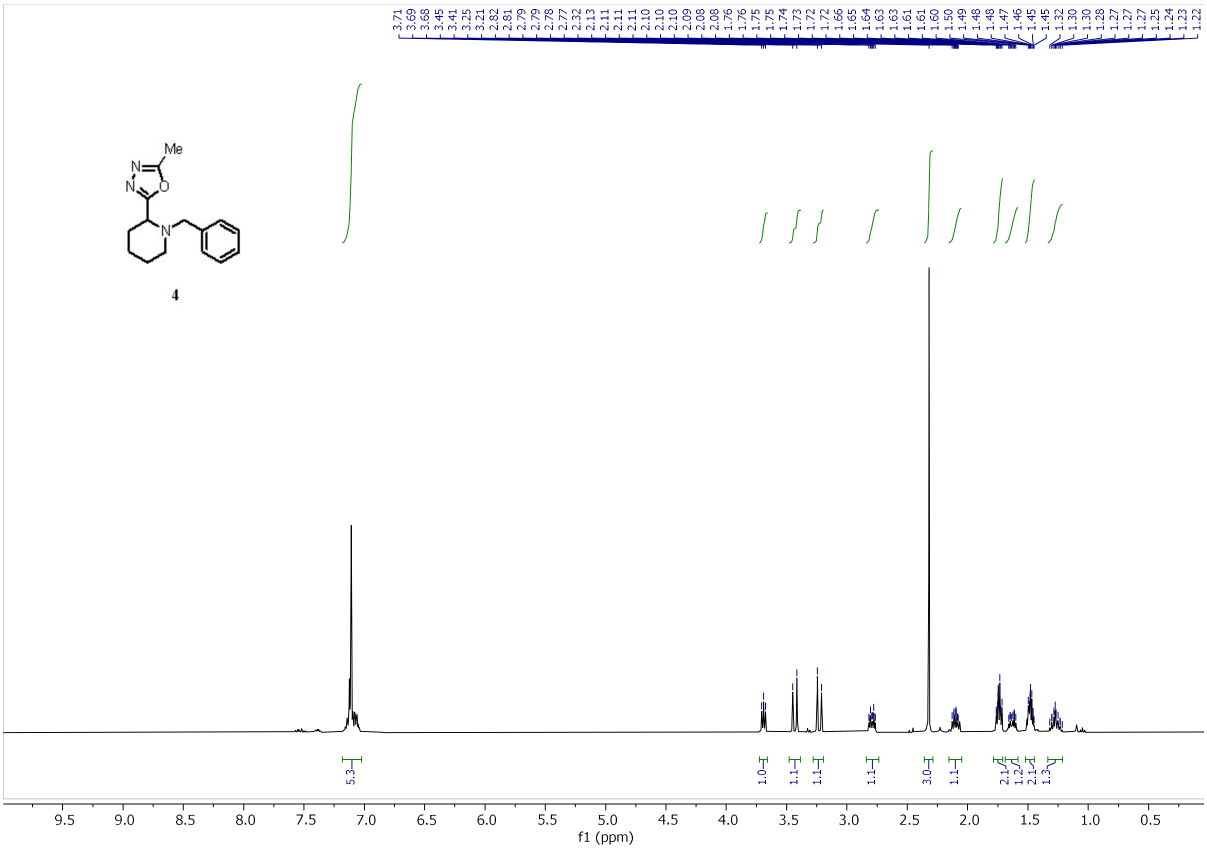

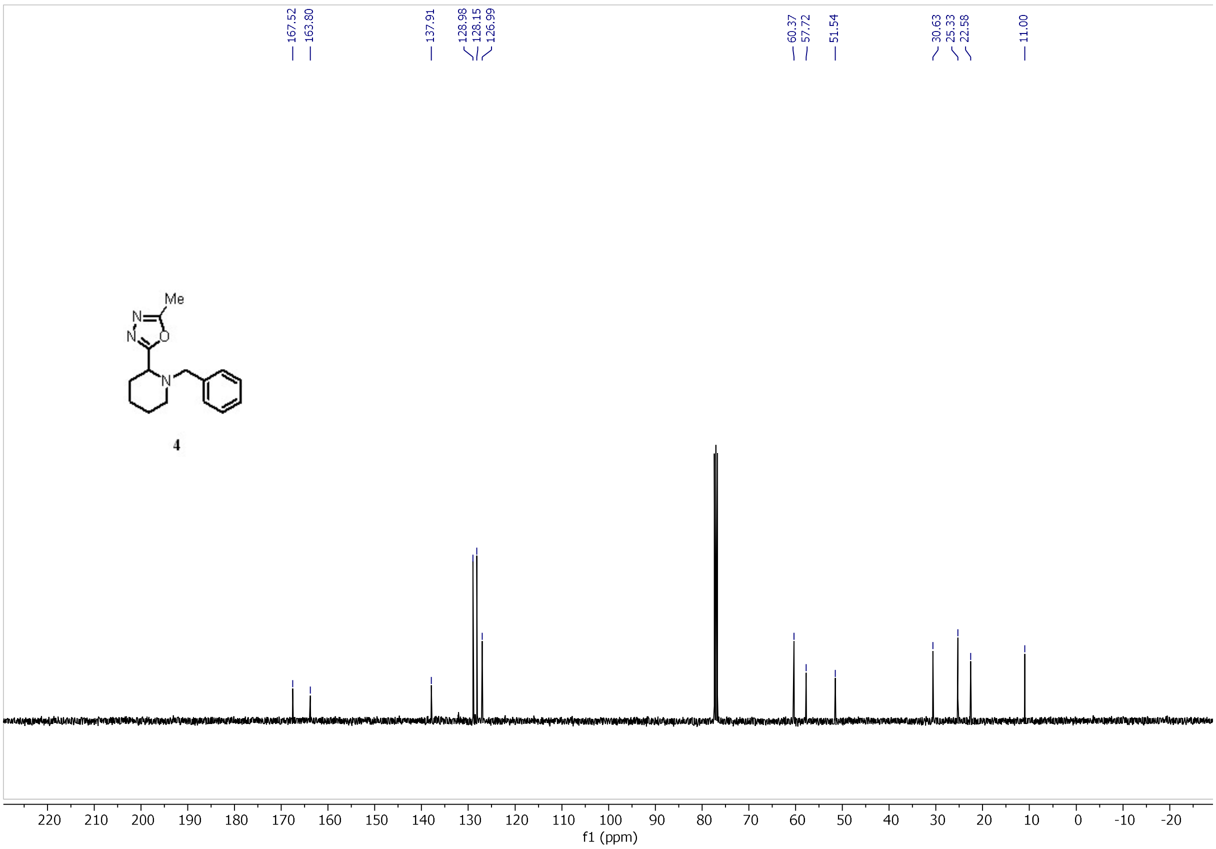

2-(1-benzylpiperidin-2-yl)-5-(1-phenylcyclopropyl)-1,3,4-oxadiazole - 5

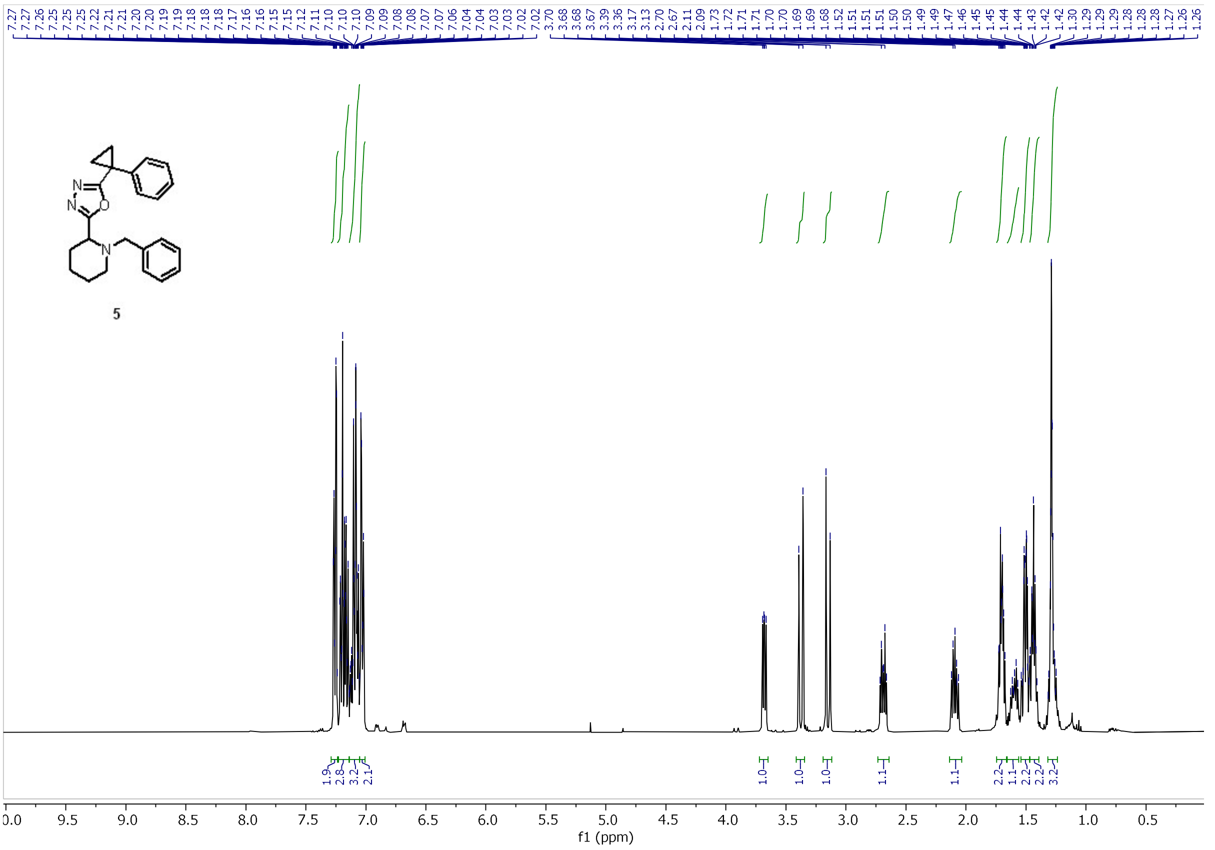

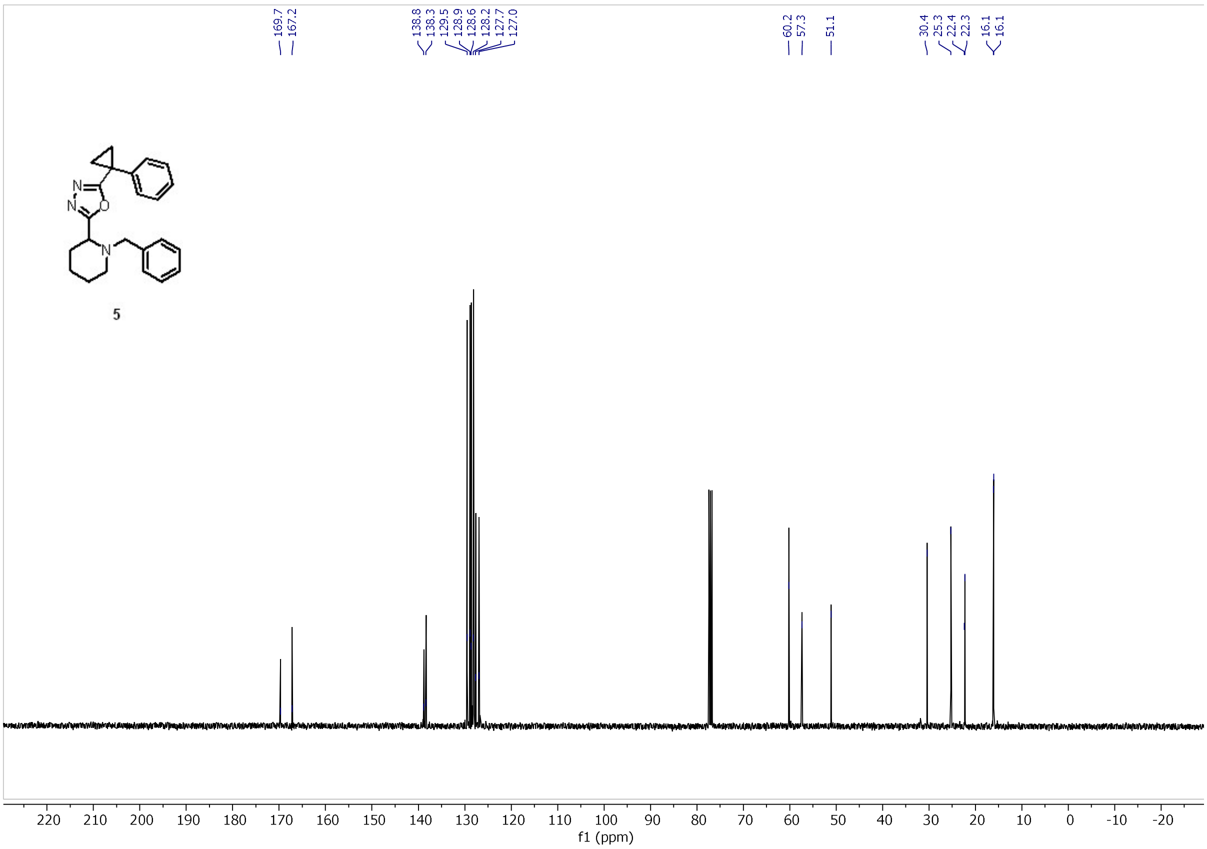

tert-butyl ((5-(1-benzylpiperidin-2-yl)-1,3,4-oxadiazol-2-yl)methyl)carbamate - 6

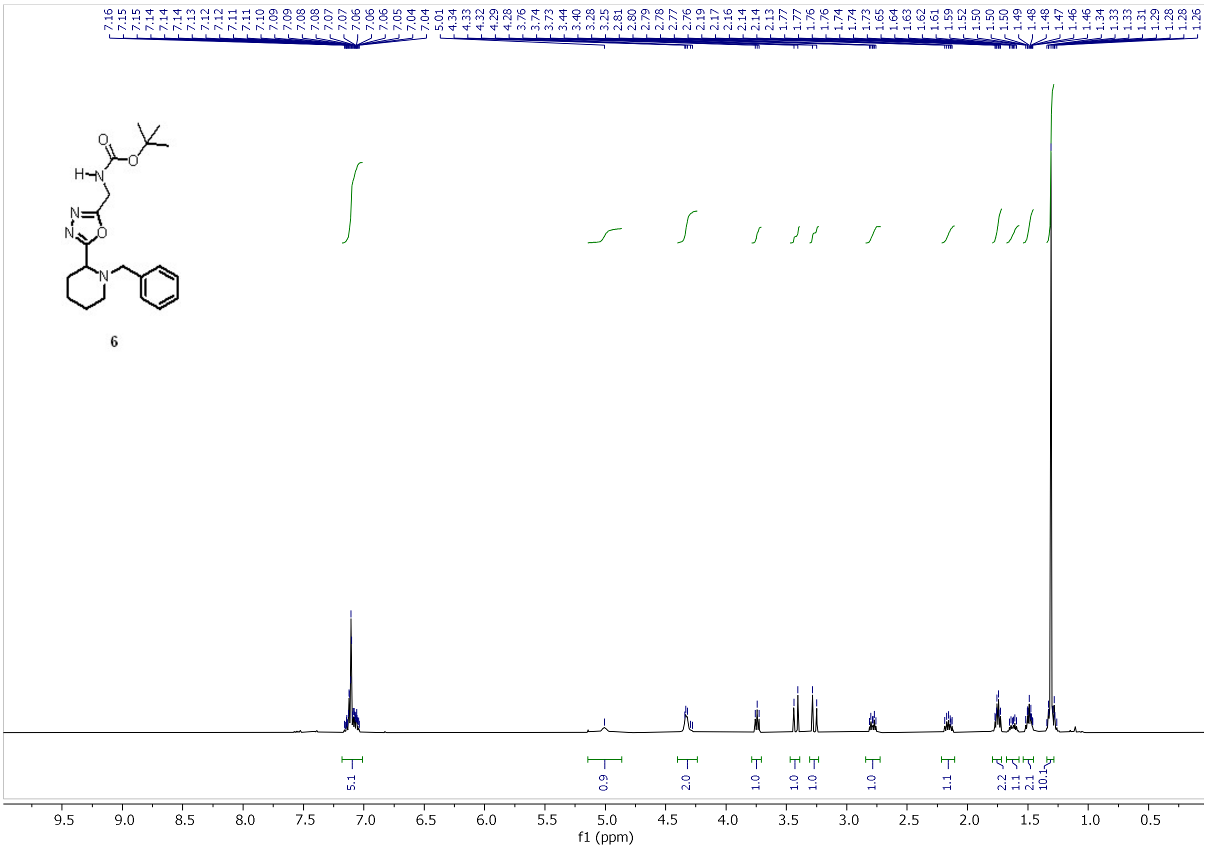

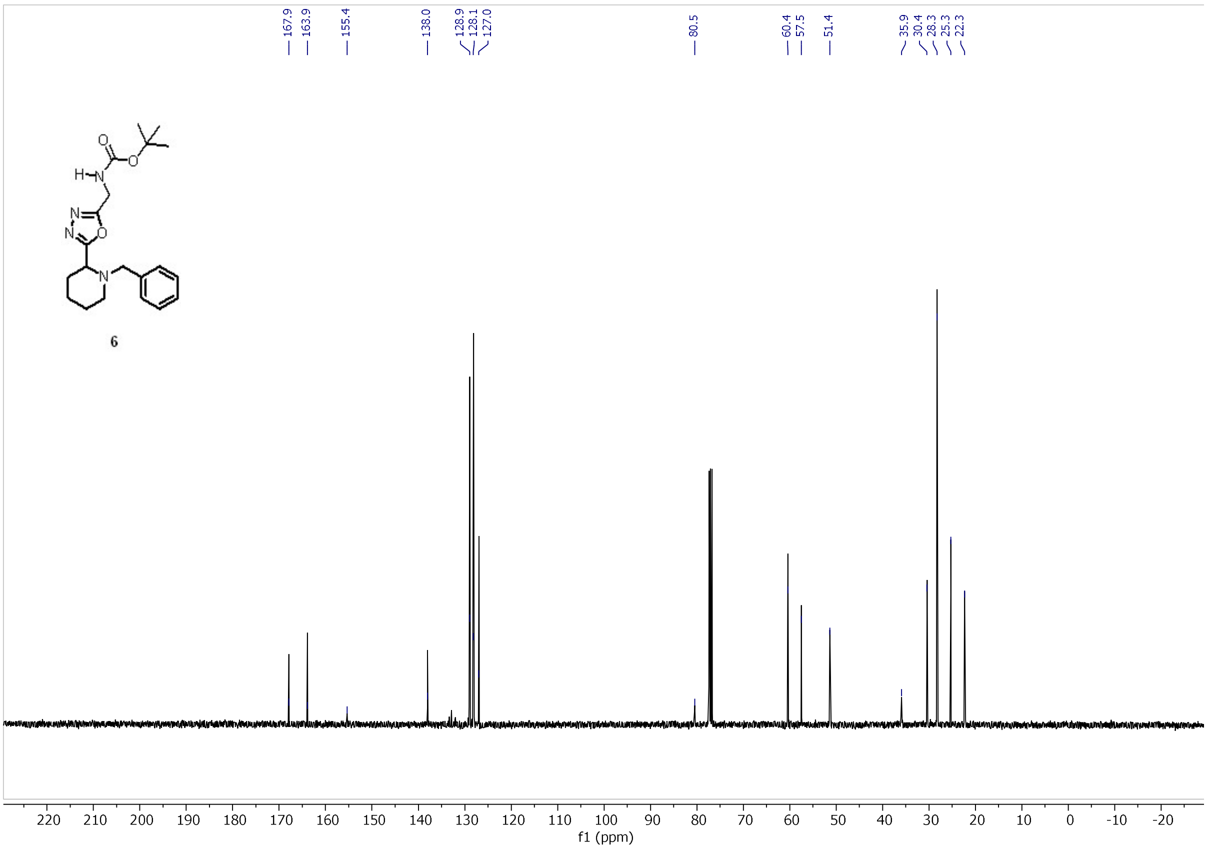

2-(1-benzylpiperidin-2-yl)-5-ethynyl-1,3,4-oxadiazole - 7

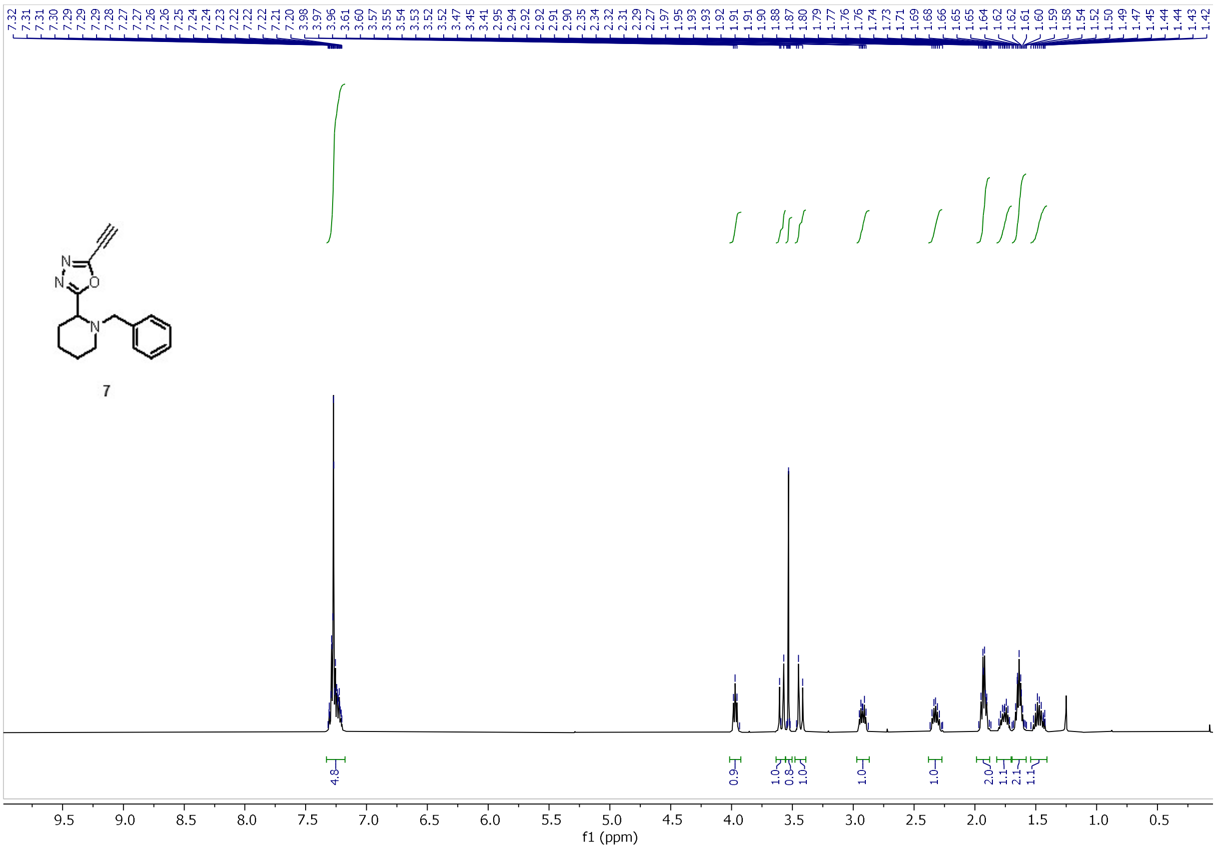

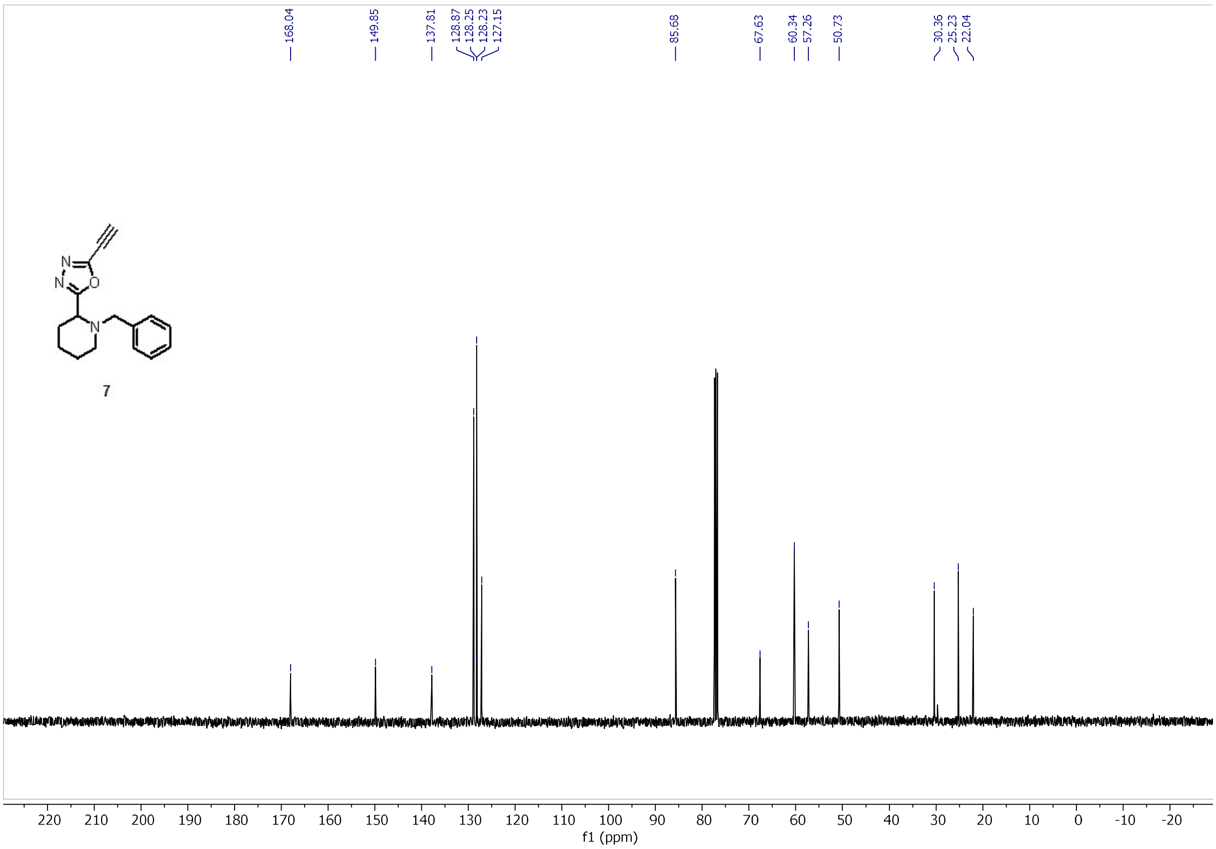

2-(1-benzylpiperidin-2-yl)-5-(trifluoromethyl)-1,3,4-oxadiazole - 8

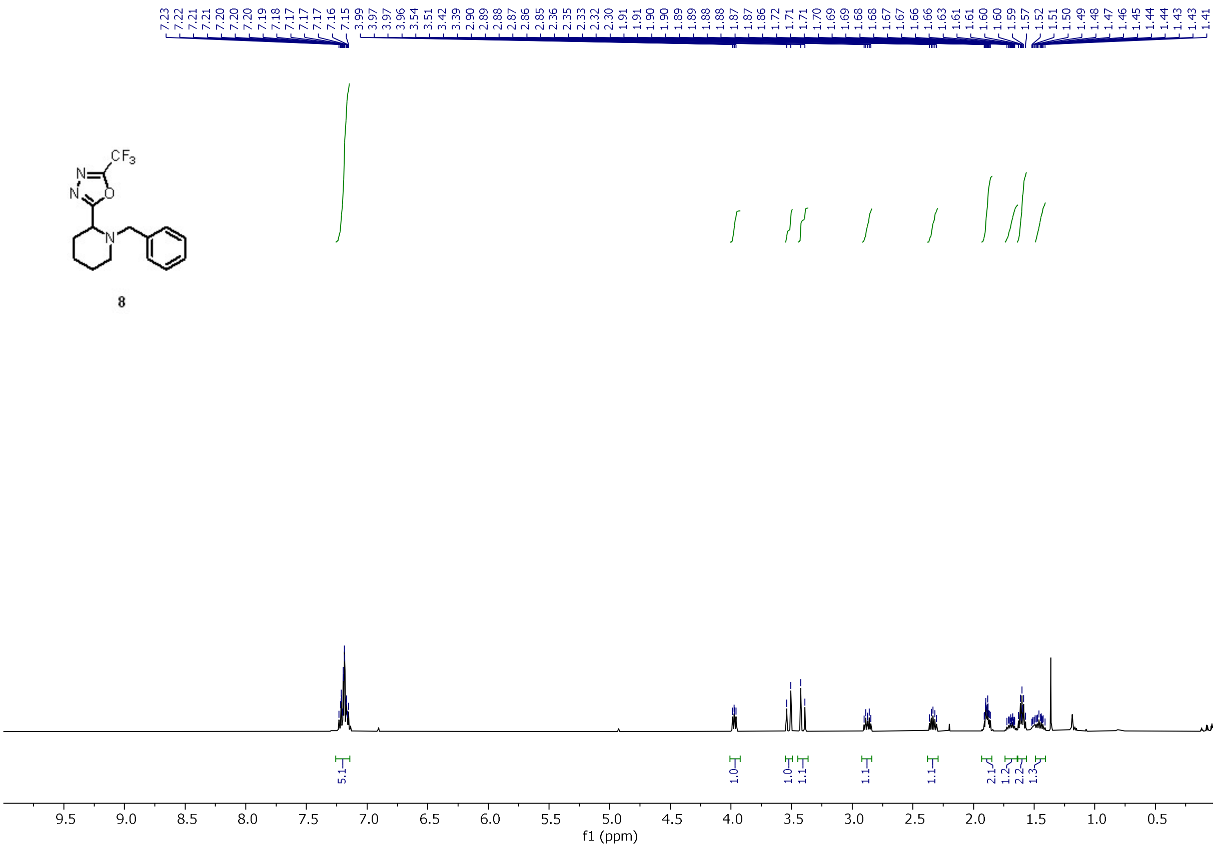

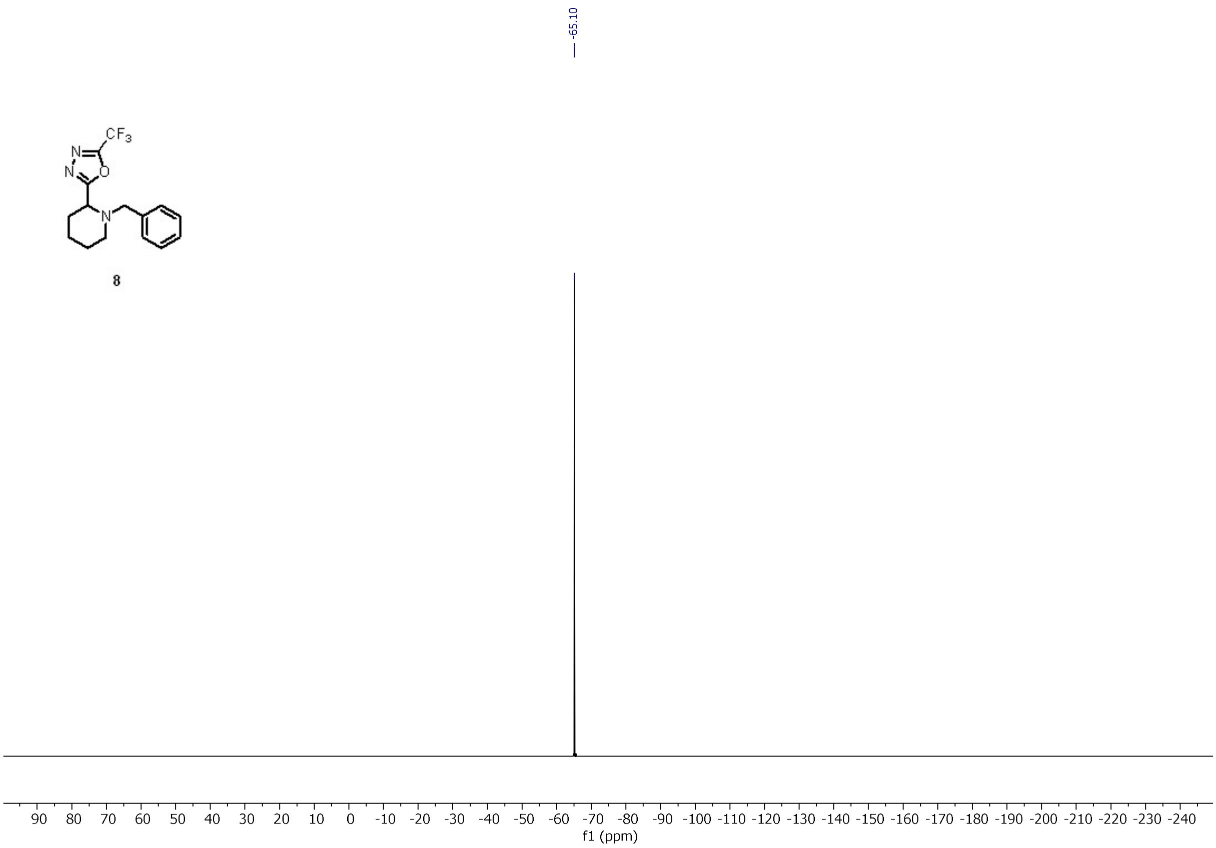
-65.10
CF3
N
N
O
N
8
90 80 70 60 50 40 30 20 10 0 -10 -20 -30 -40 -50 -60 -70 -80 -90 -100 -110 -120 -130 -140 -150 -160 -170 -180 -190 -200 -210 -220 -230 -240
f1 (ppm)

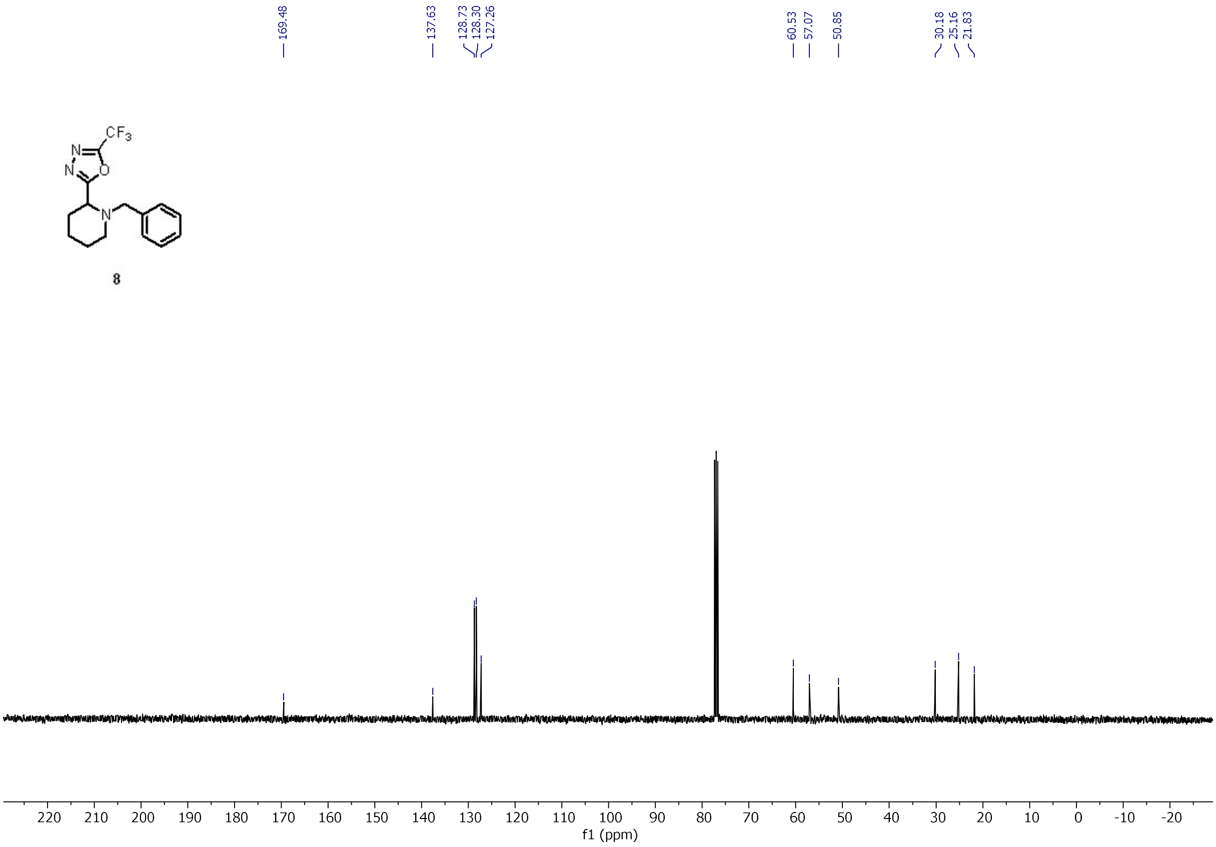
169.48
137.63
128.73
128.30
127.26
60.53
57.07
50.85
30.18
25.16
21.83
CF3
N
N
O
N
8
220 210 200 190 180 170 160 150 140 130 120 110 100 90 80 70 60 50 40 30 20 10 0 -10 -20
f1 (ppm)

2-(1-benzylpiperidin-2-yl)-5-(pyrazin-2-yl)-1,3,4-oxadiazole - 9

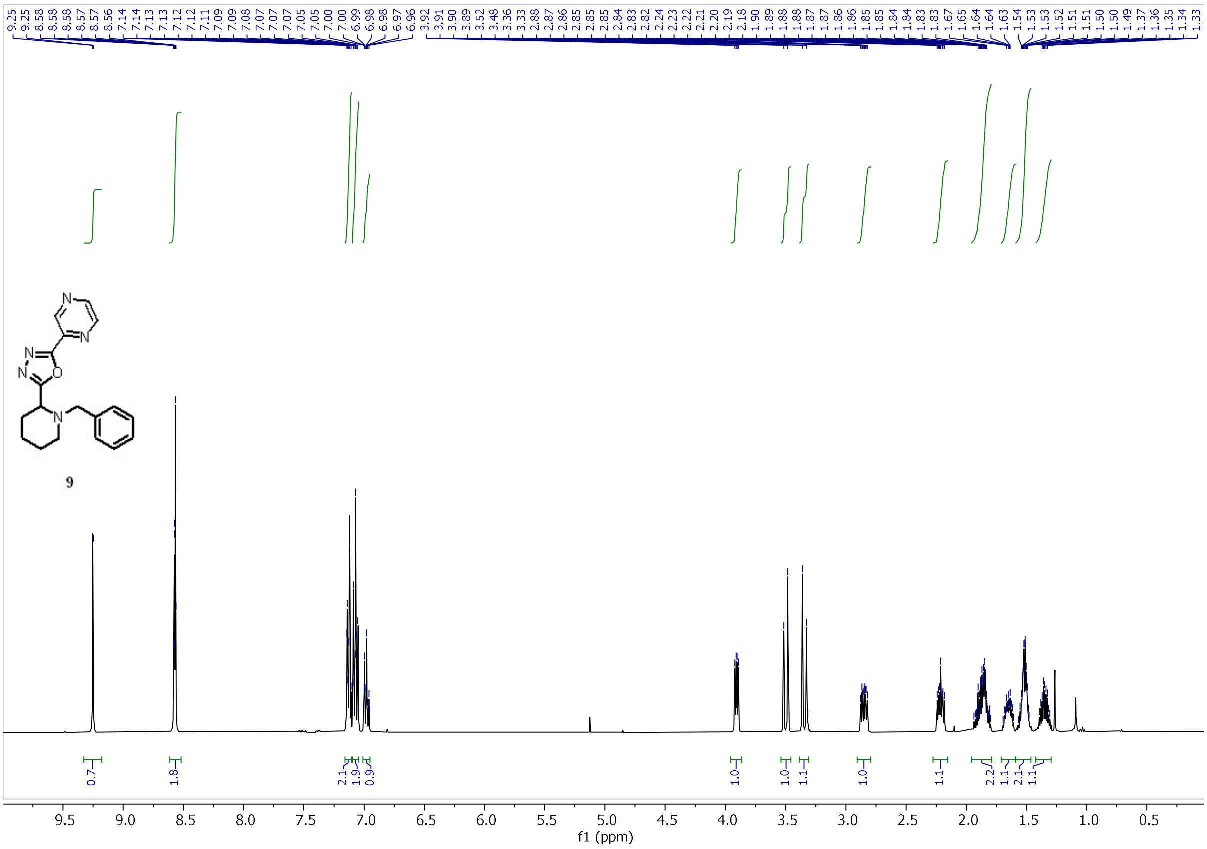

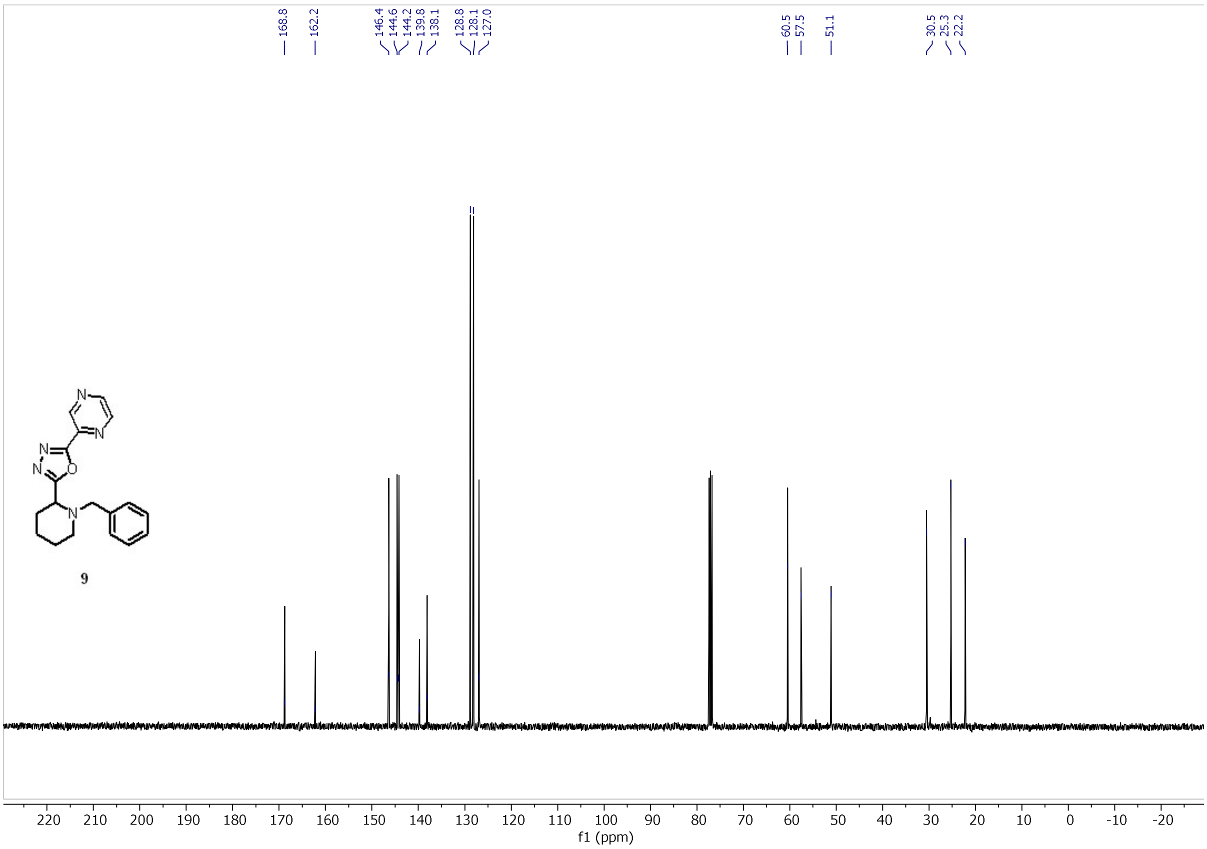

2-(1-benzylpiperidin-2-yl)-5-(5-bromopyridin-3-yl)-1,3,4-oxadiazole - 10

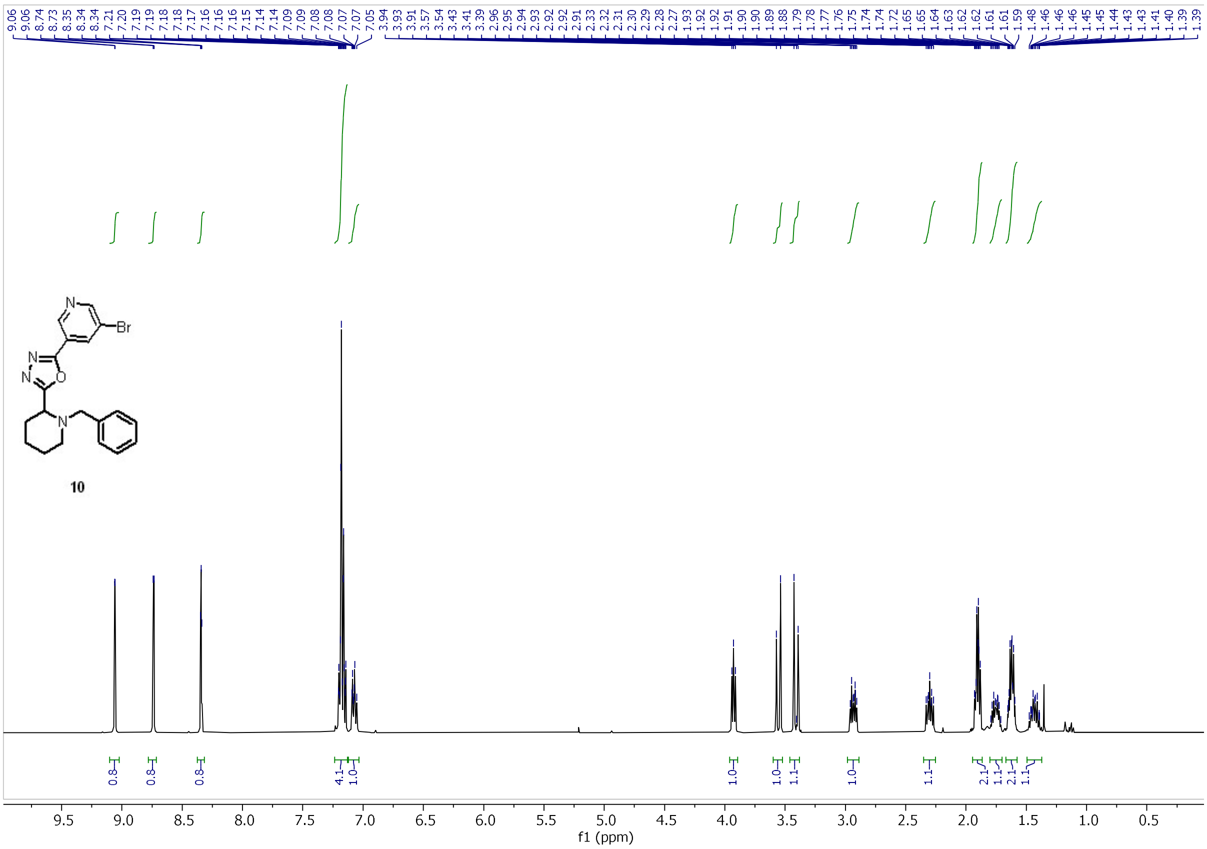

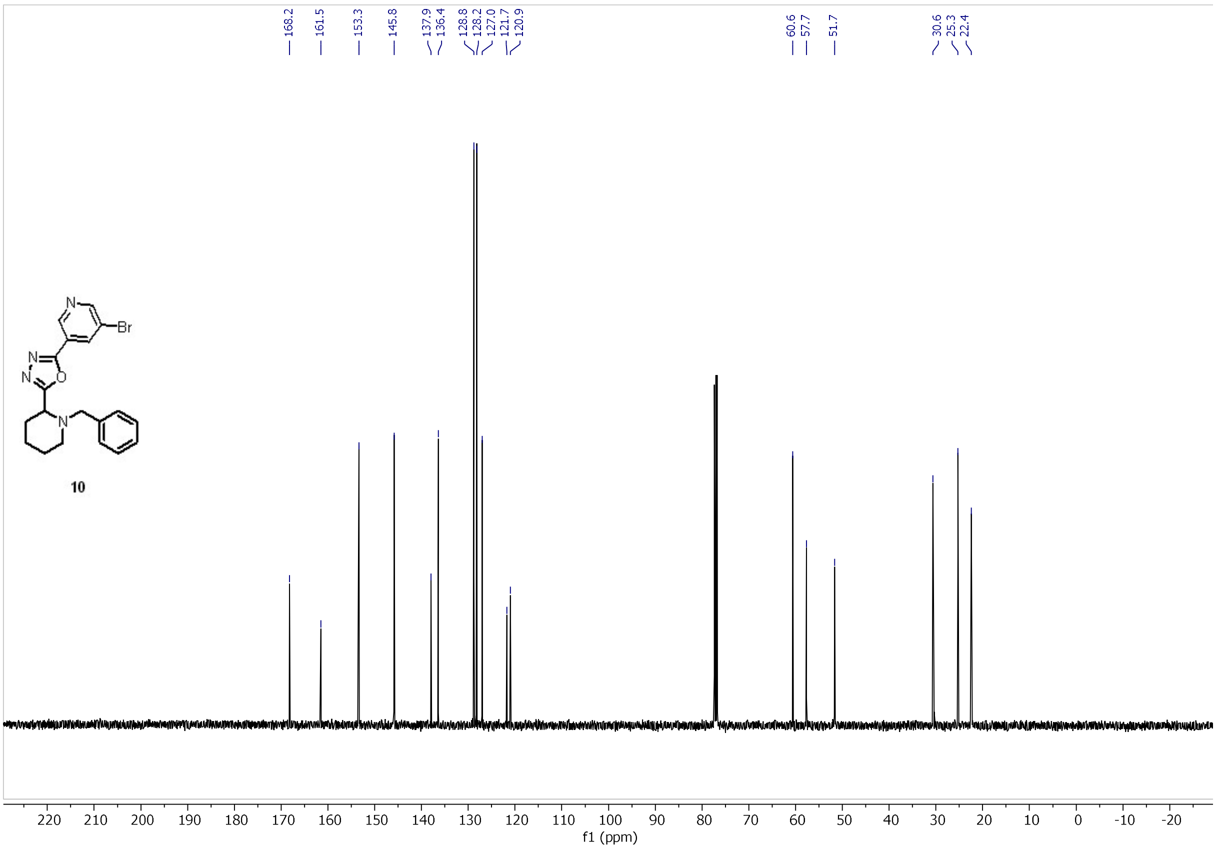

2-(1-benzylpiperidin-2-yl)-5-(1H-indazol-7-yl)-1,3,4-oxadiazole - 11

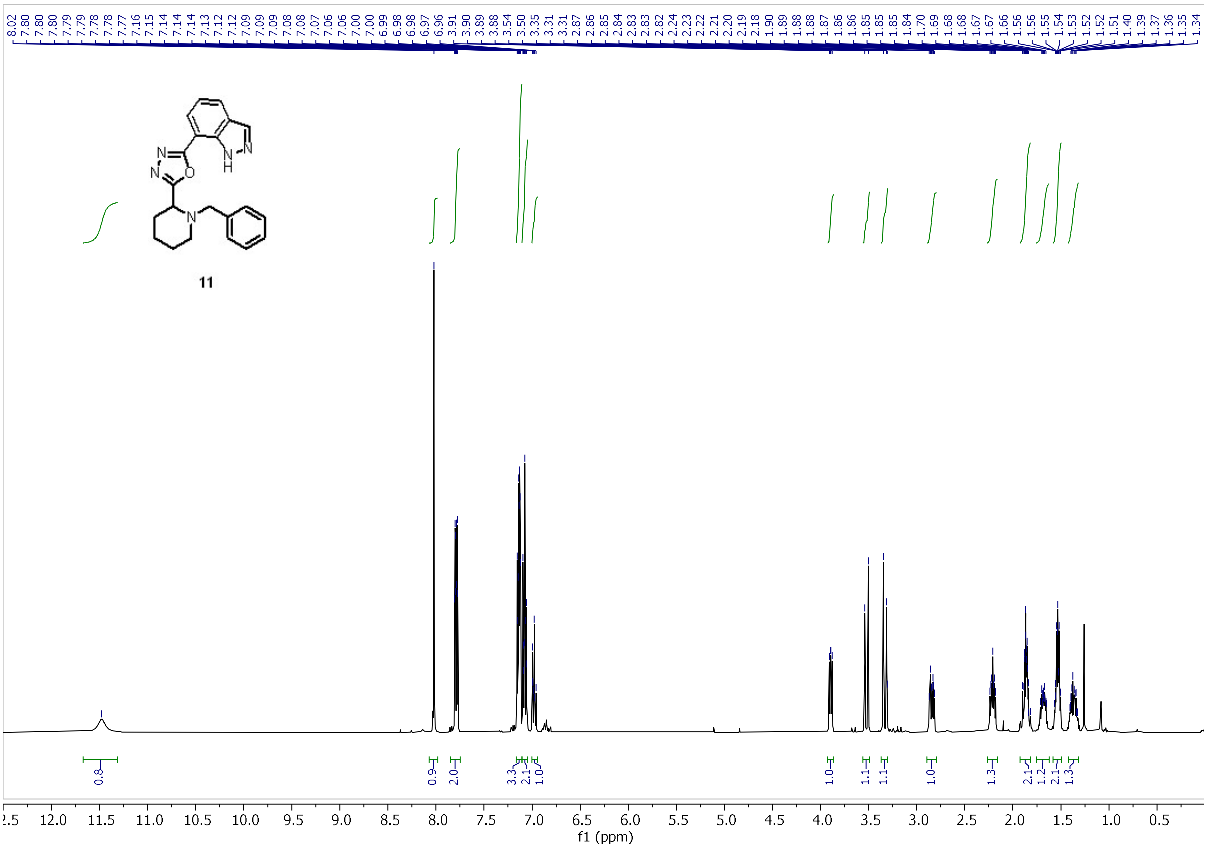

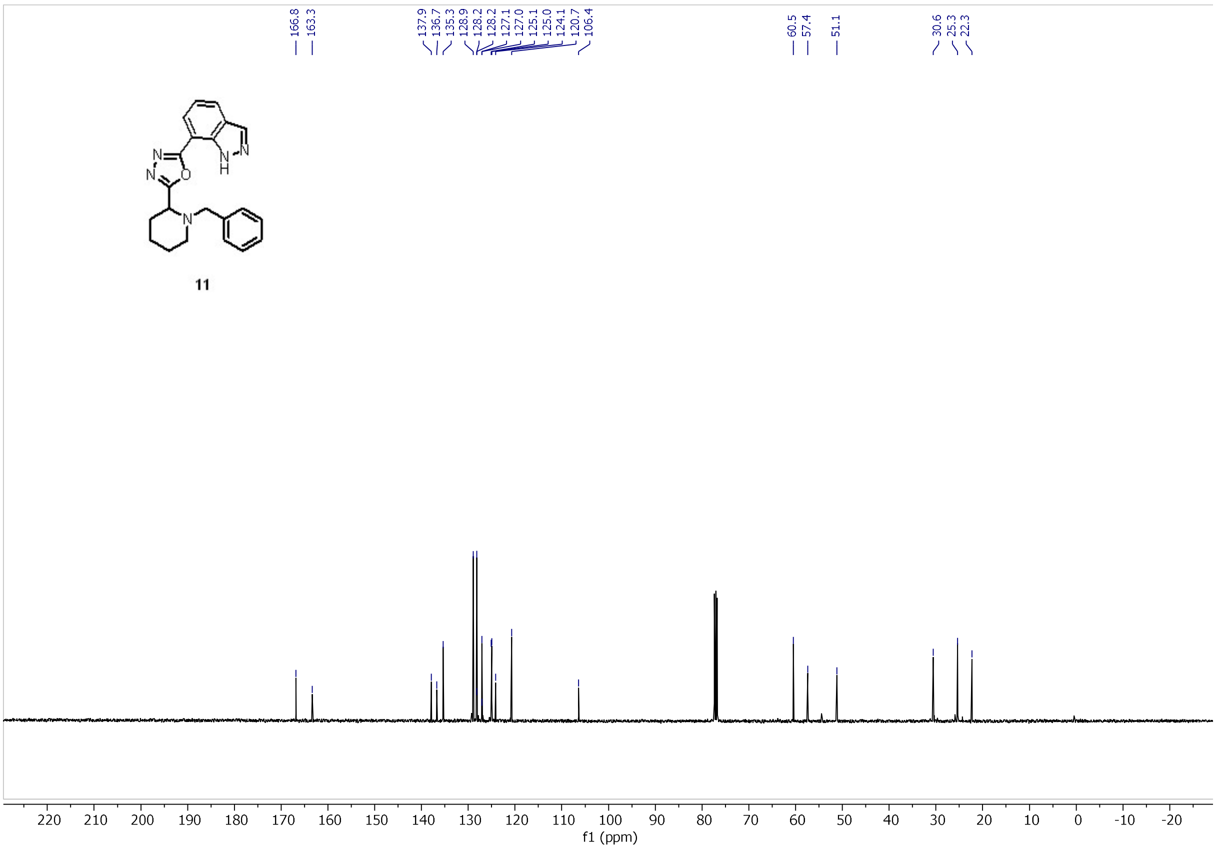

2-((5-(1-benzylpiperidin-2-yl)-1,3,4-oxadiazol-2-yl)methoxy)benzaldehyde - 12

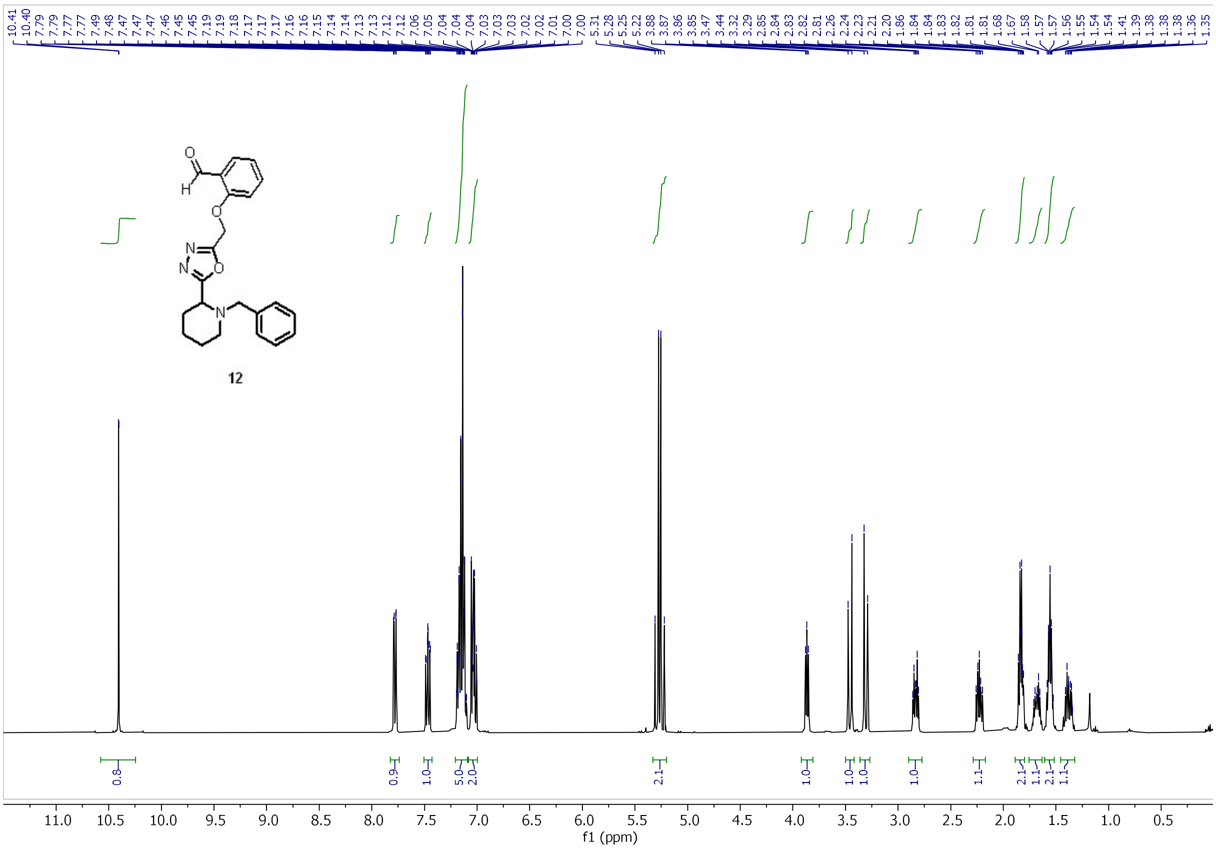

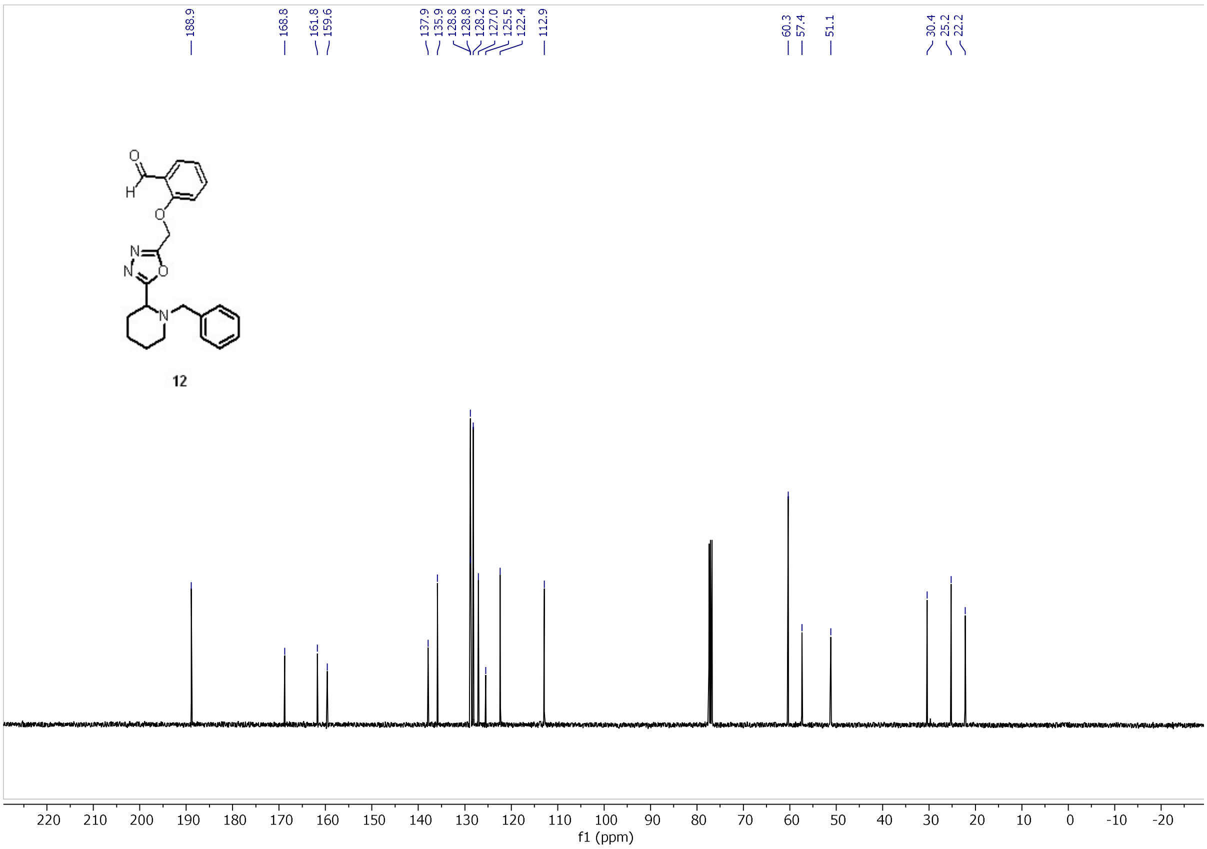

4-(5-(1-benzylpiperidin-2-yl)-1,3,4-oxadiazol-2-yl)benzenesulfonyl fluoride - 13

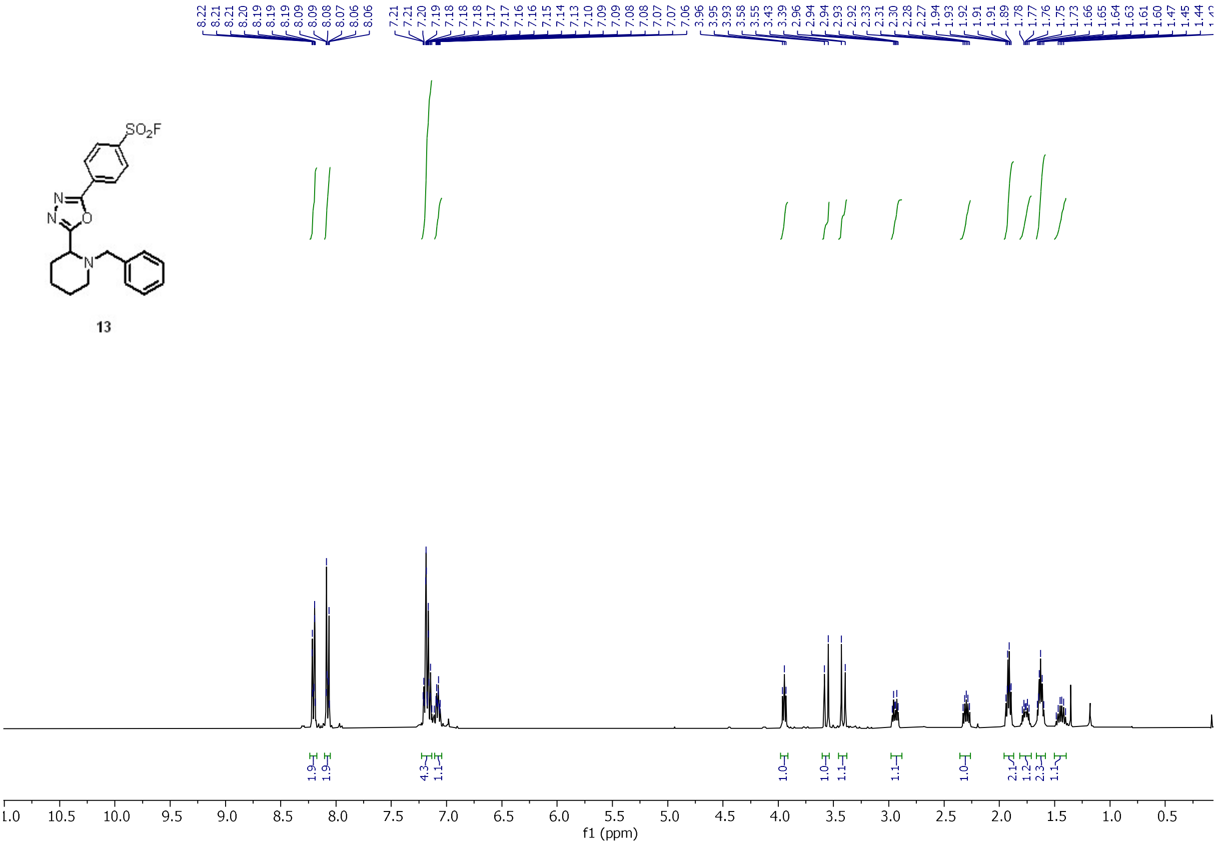

13

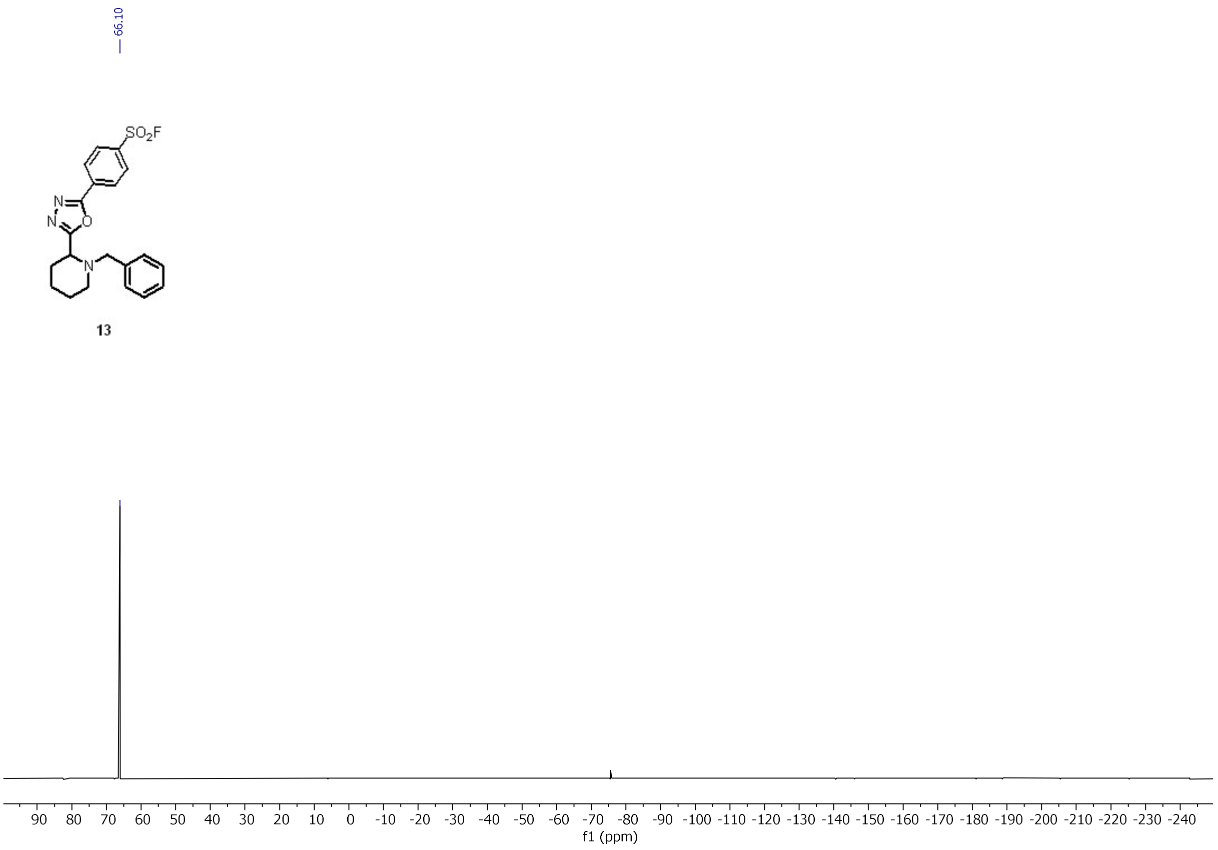

13

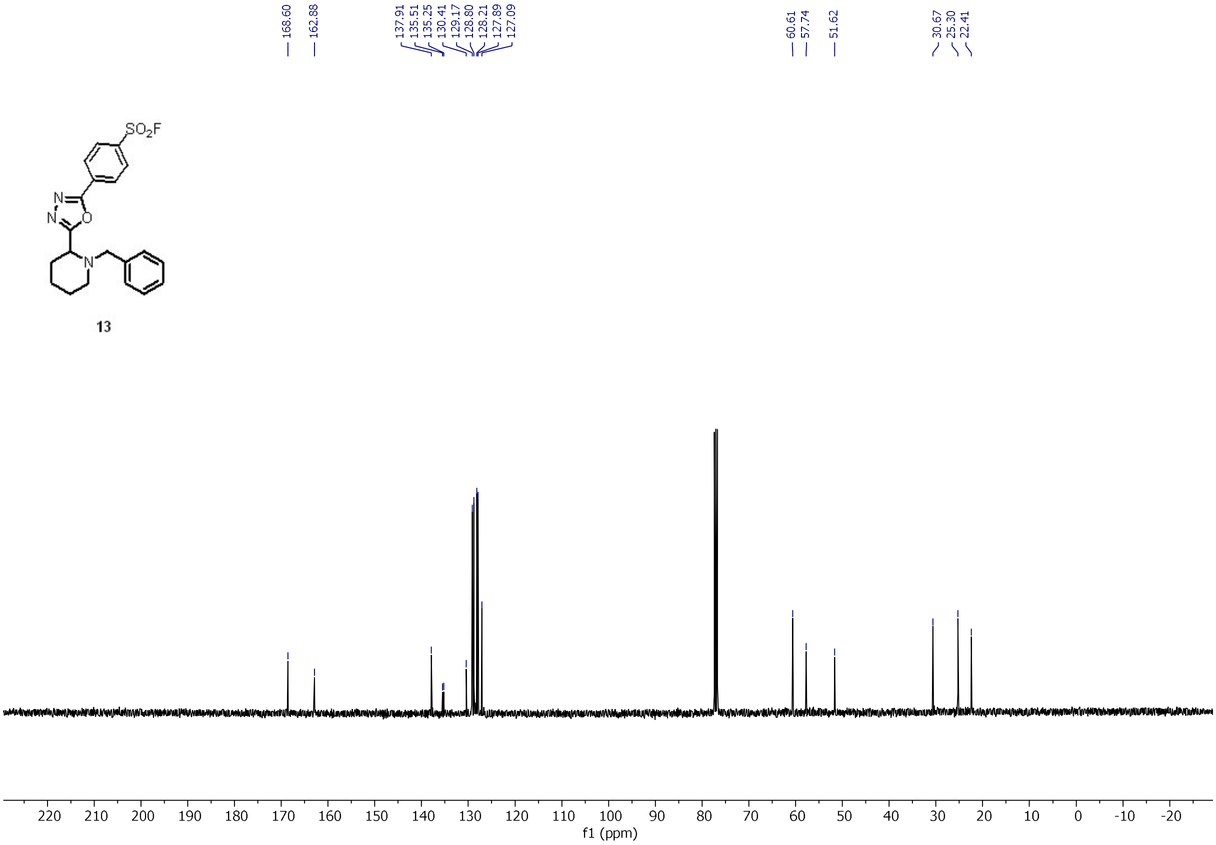

2-(1-benzylpiperidin-2-yl)-5-(3,3,3-trifluoroprop-1-en-2-yl)-1,3,4-oxadiazole - 14

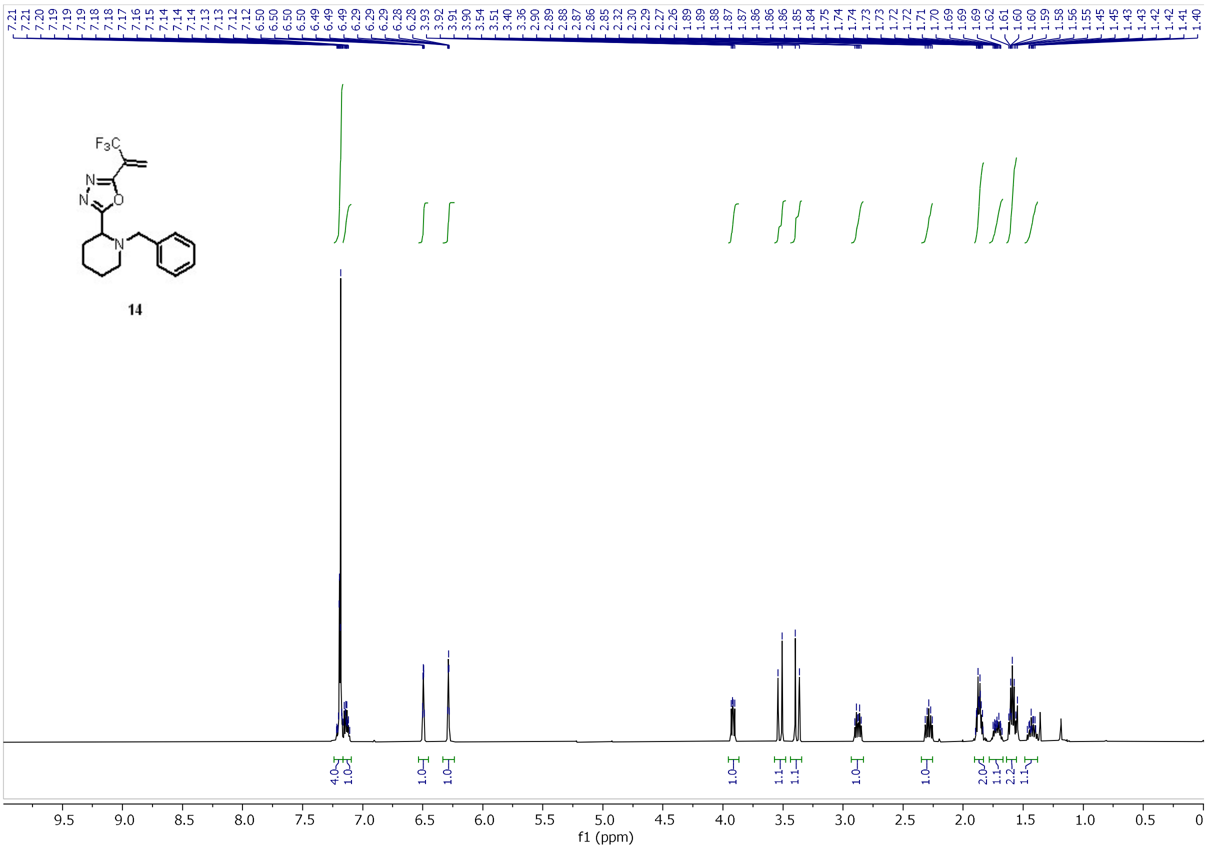

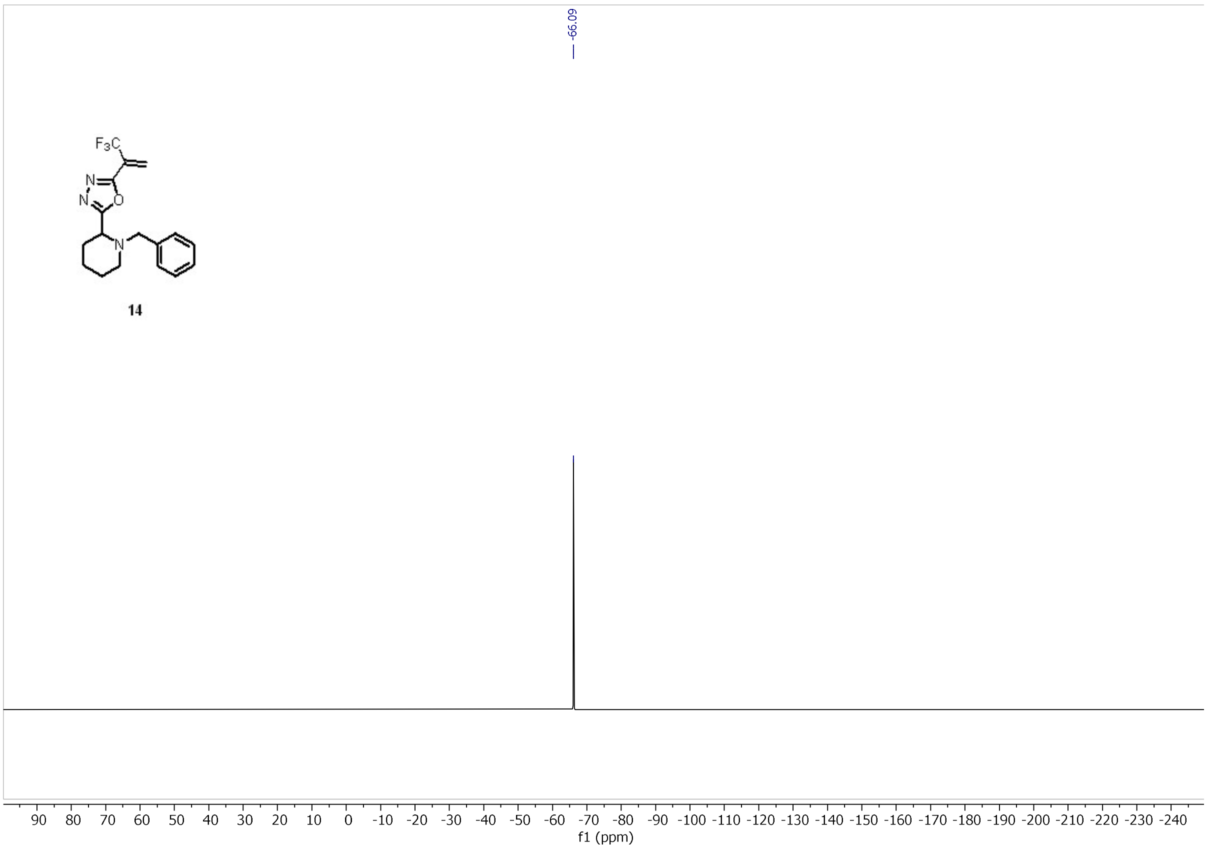
-66.09
F3C
N
N
O
N
14
90
80
70
60
50
40
30
20
10
0
-10
-20
-30
-40
-50
-60
-70
-80
-90
-100
-110
-120
-130
-140
-150
-160
-170
-180
-190
-200
-210
-220
-230
-240
f1 (ppm)

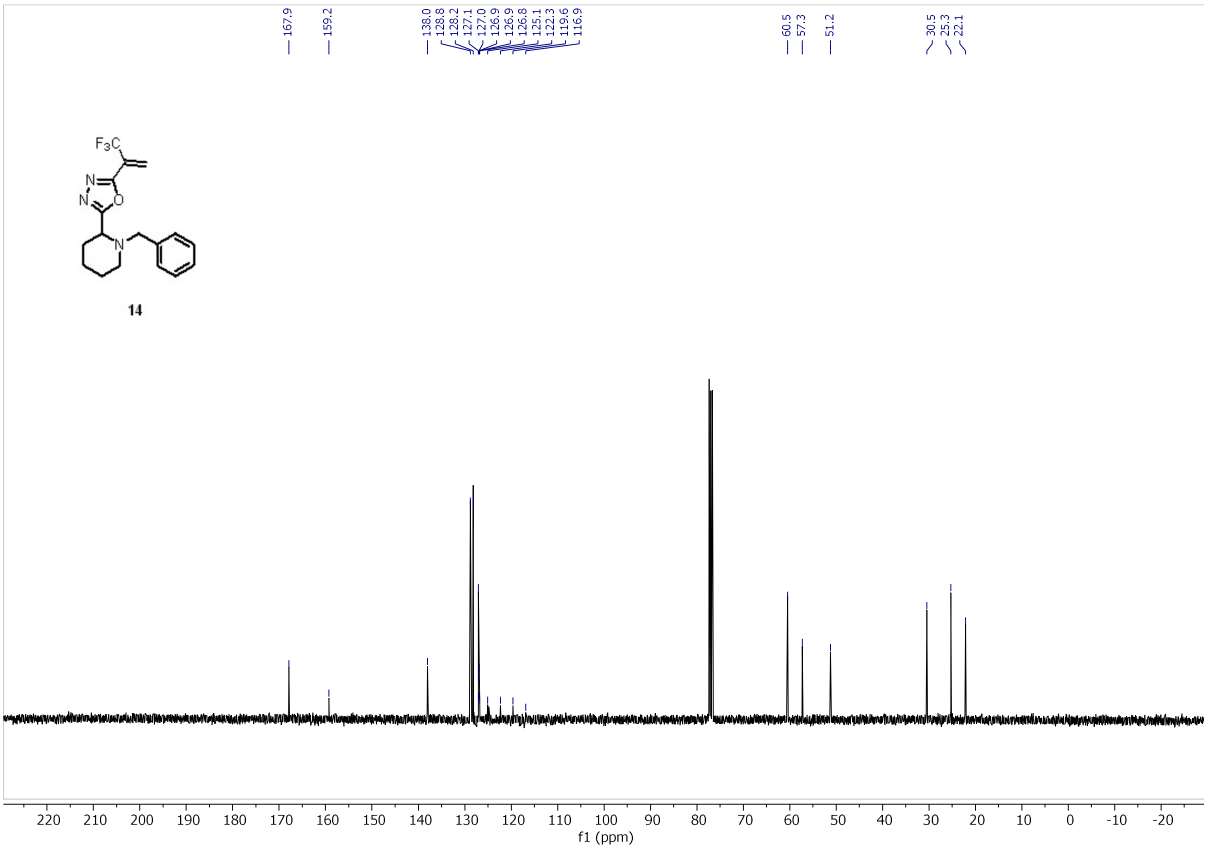
167.9
159.2
138.0
128.8
128.2
127.1
127.0
126.9
126.9
126.8
125.1
122.3
119.6
116.9
60.5
57.3
51.2
30.5
25.3
22.1
F3C
N
N
O
N
14
220
210
200
190
180
170
160
150
140
130
120
110
100
90
80
70
60
50
40
30
20
10
0
-10
-20
f1 (ppm)

(E)-2-(1-benzylpiperidin-2-yl)-5-(3-bromoprop-1-en-1-yl)-1,3,4-oxadiazole - 15

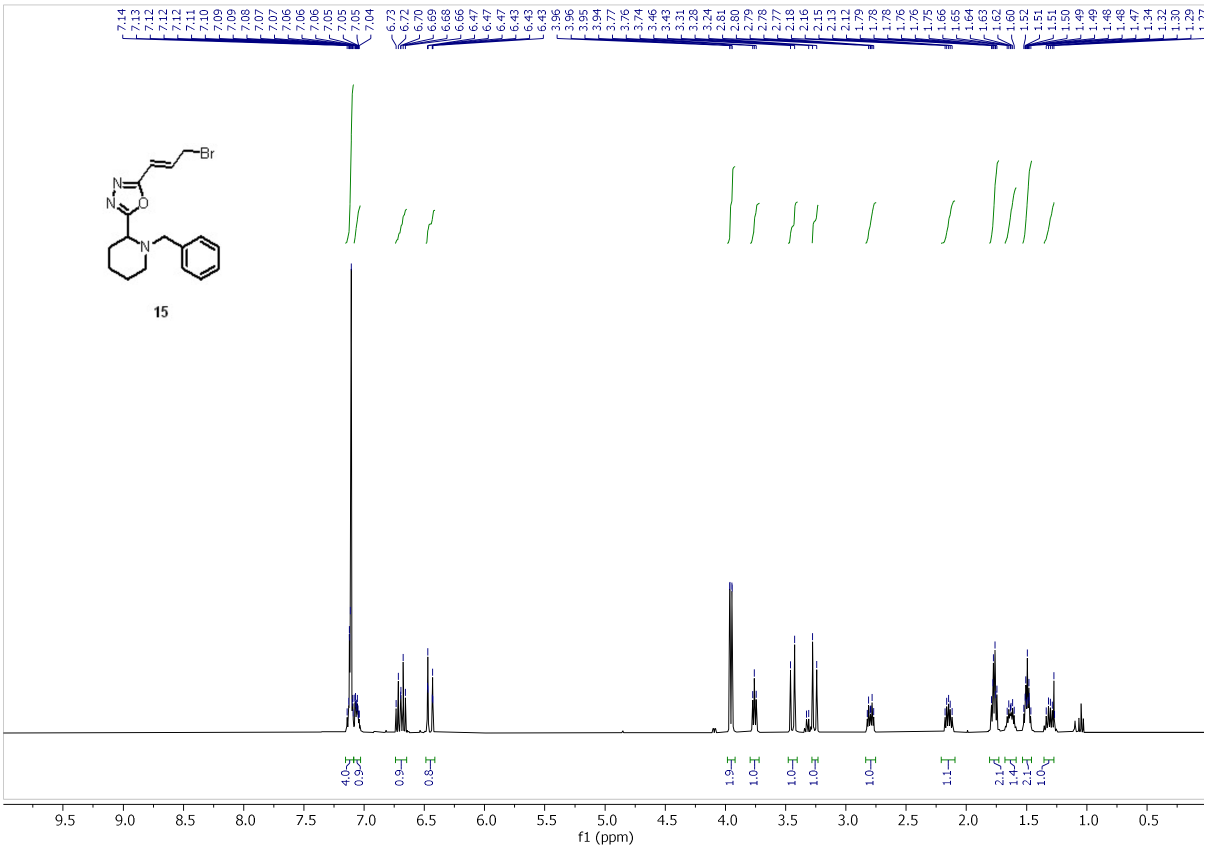

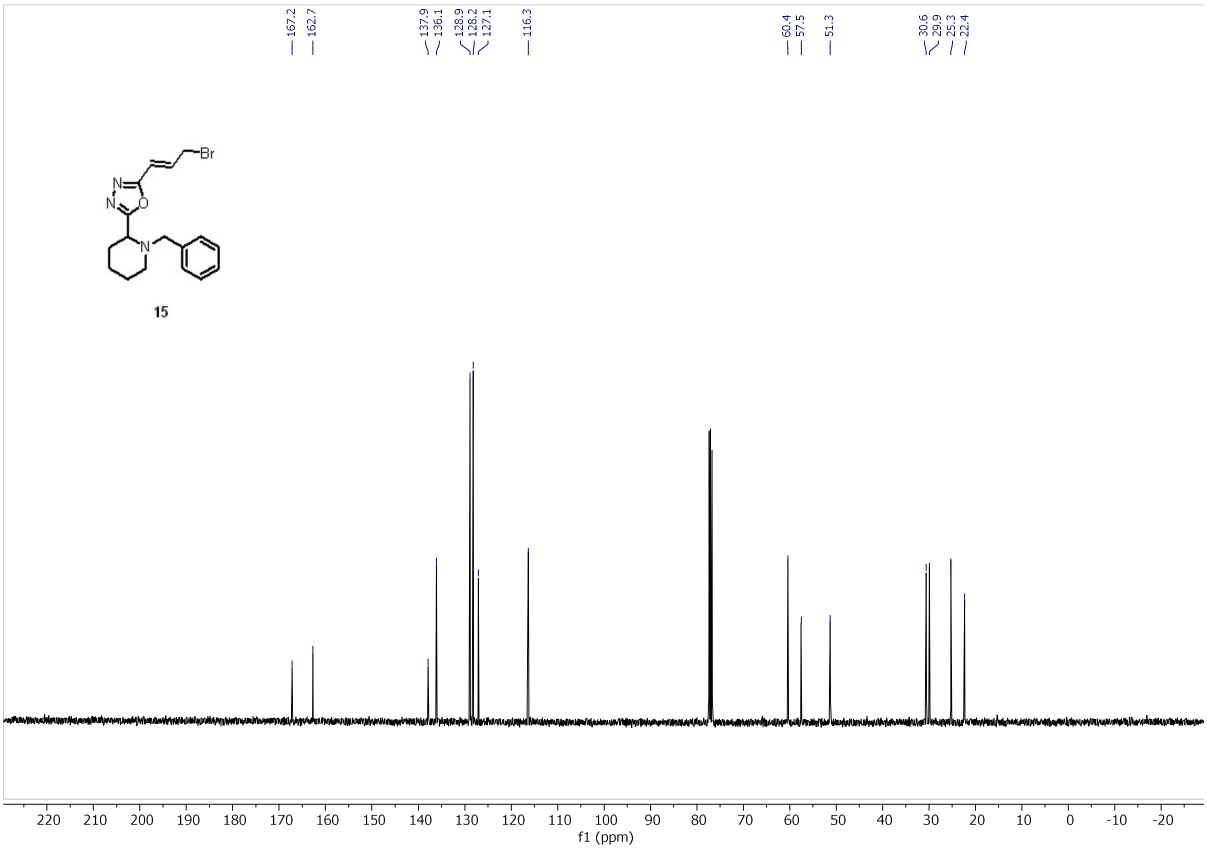

2-(1-benzylpiperidin-2-yl)-5-(bromomethyl)-1,3,4-oxadiazole - 16

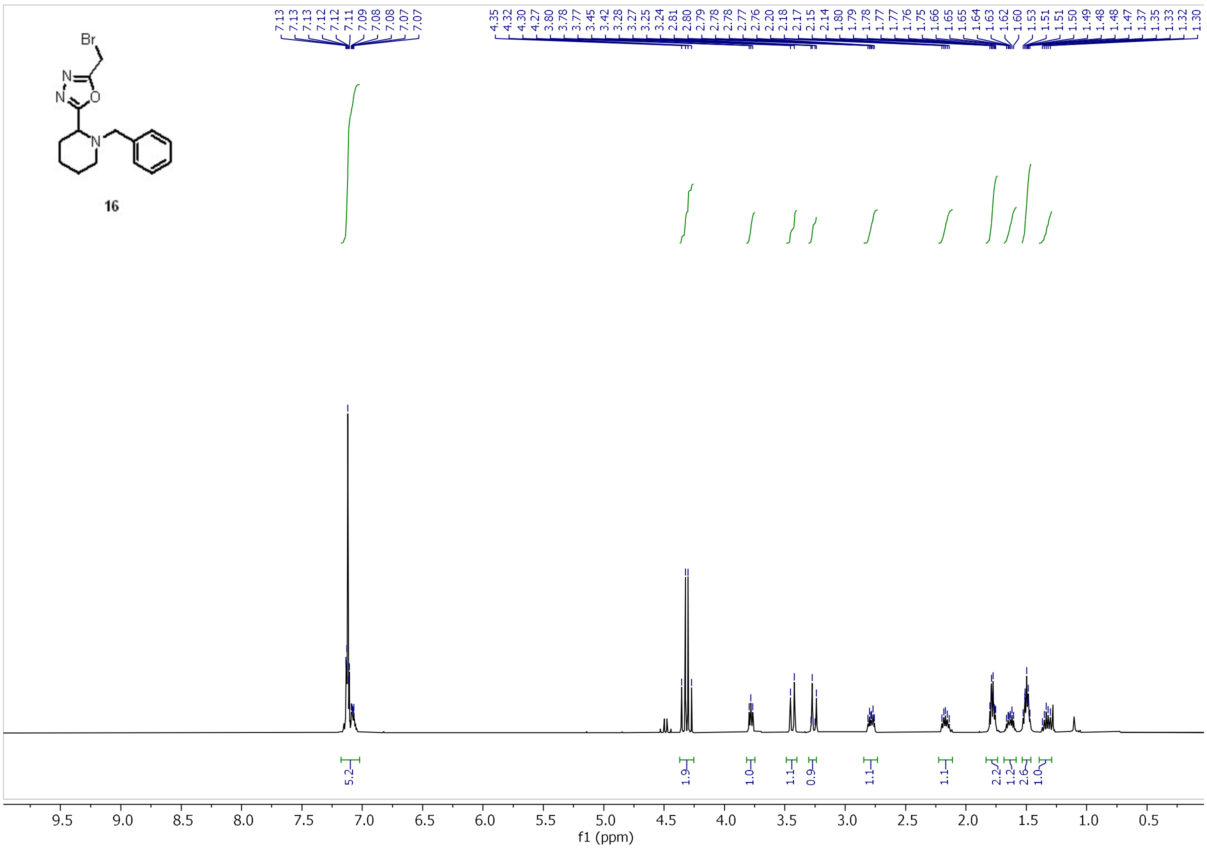

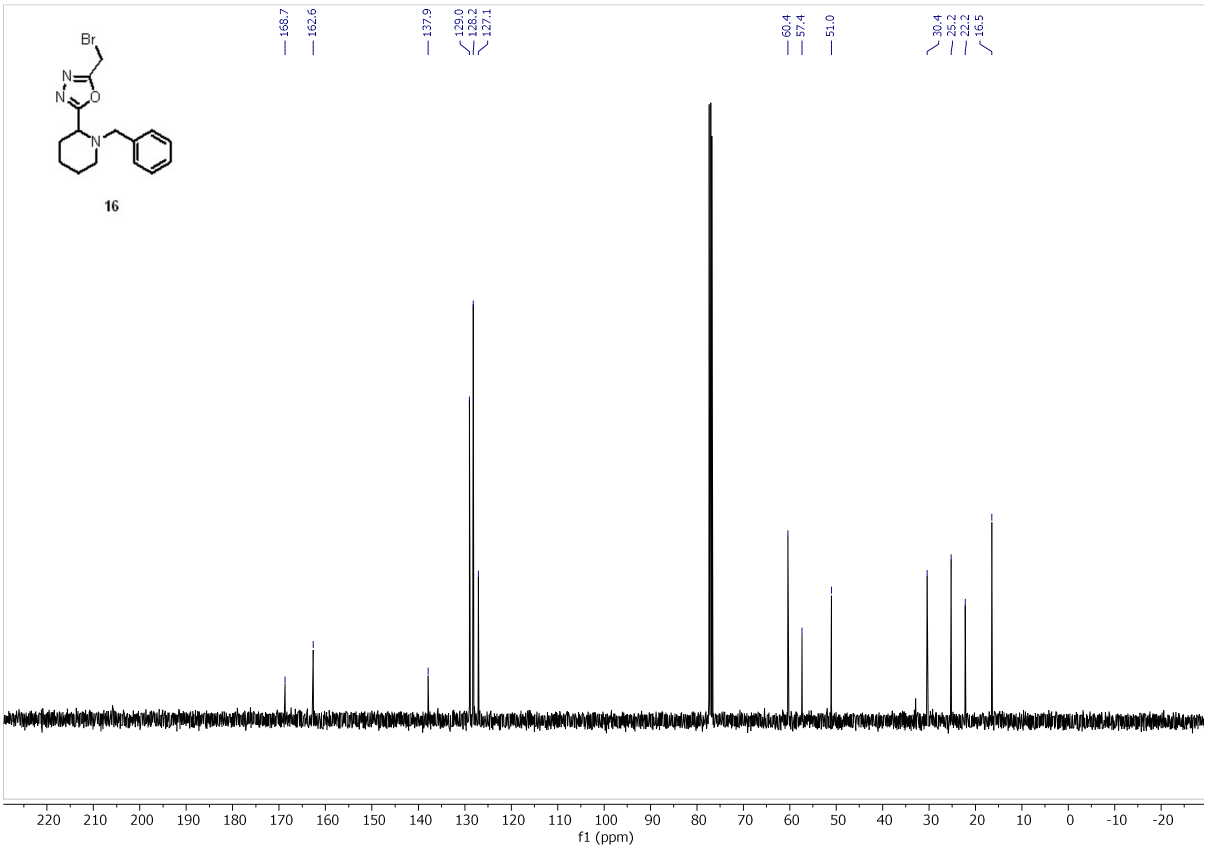

1-(5-(1-benzylpiperidin-2-yl)-1,3,4-oxadiazol-2-yl)ethan-1-one - 17

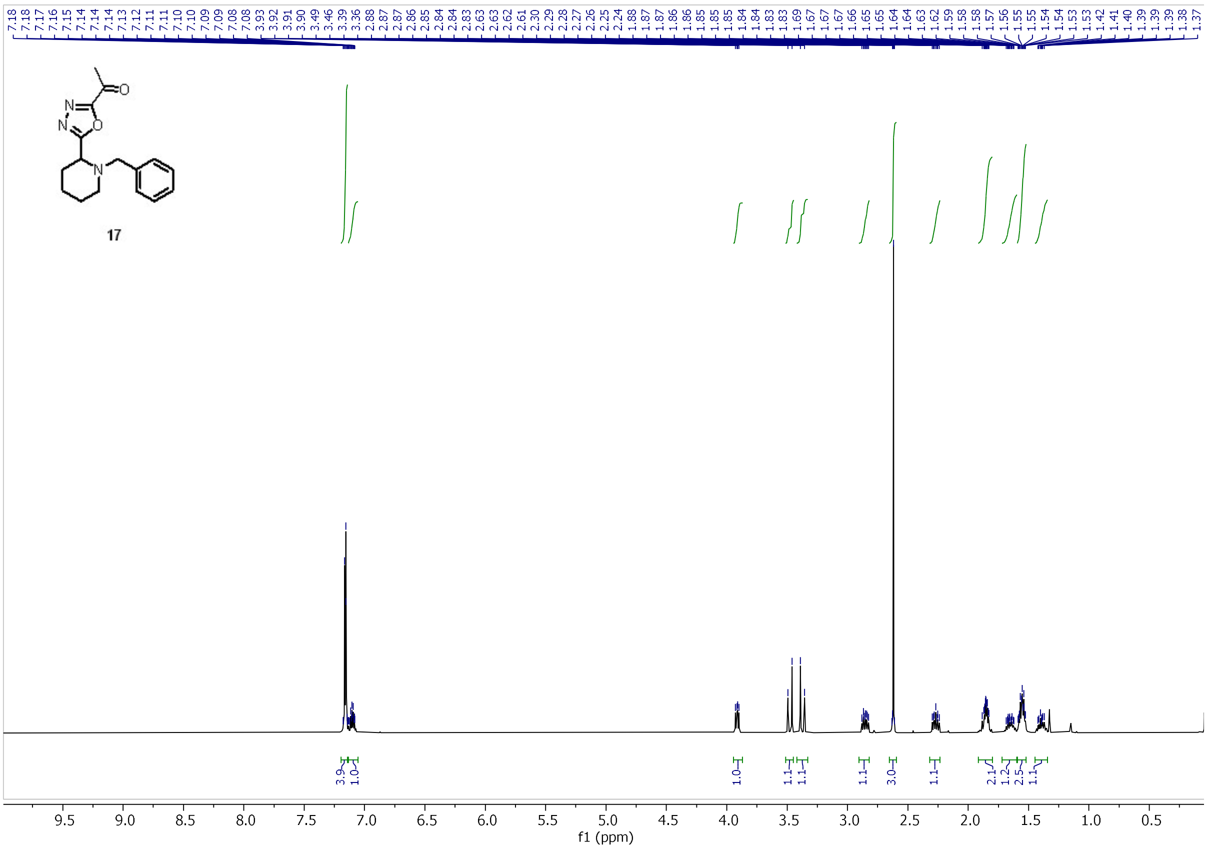

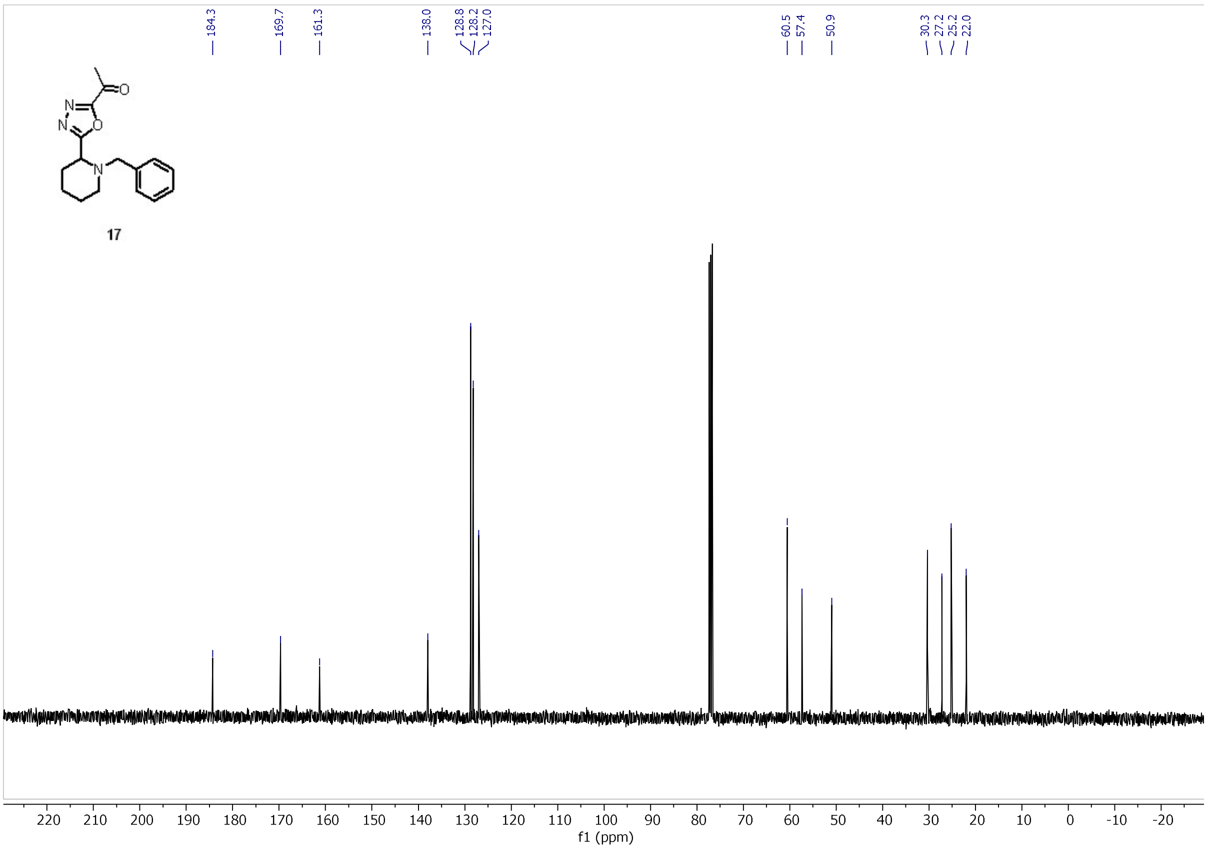

1-(tert-butoxycarbonyl)piperidin-4-yl 5-(1-benzylpiperidin-2-yl)-1,3,4-oxadiazole-2-carboxylate - 18

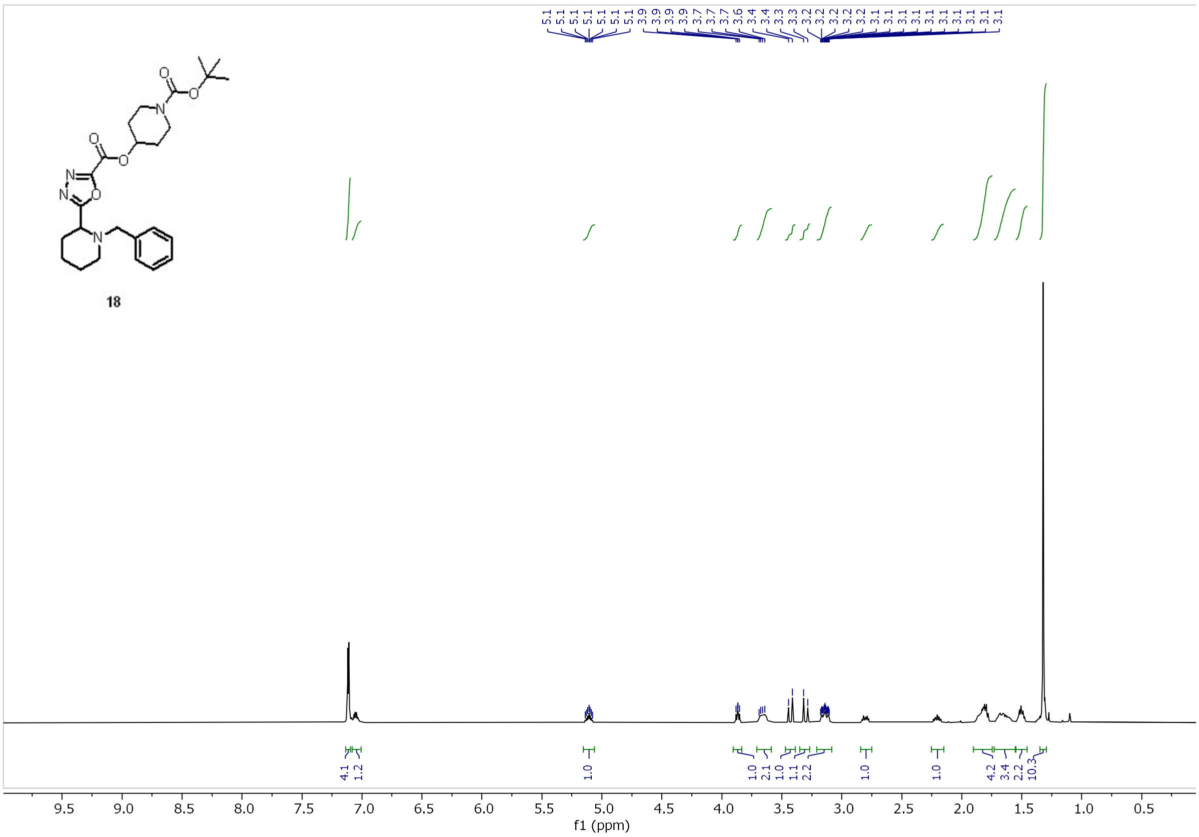

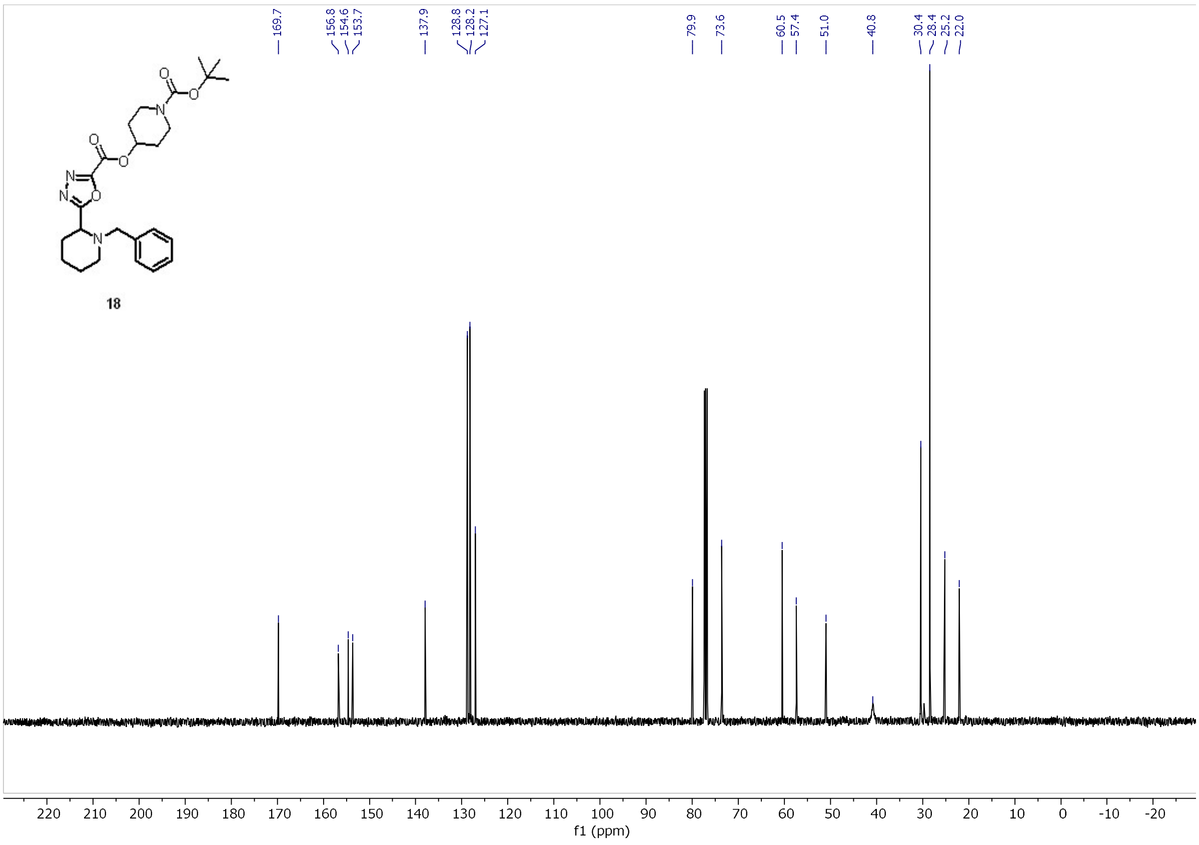

2-(1-benzylpiperidin-2-yl)-5-((2-methyl-4-(((4-methyl-2-(4-(trifluoromethyl)phenyl)thiazol-5-yl)methyl)thio)phenoxy)methyl)-1,3,4-oxadiazole - 19

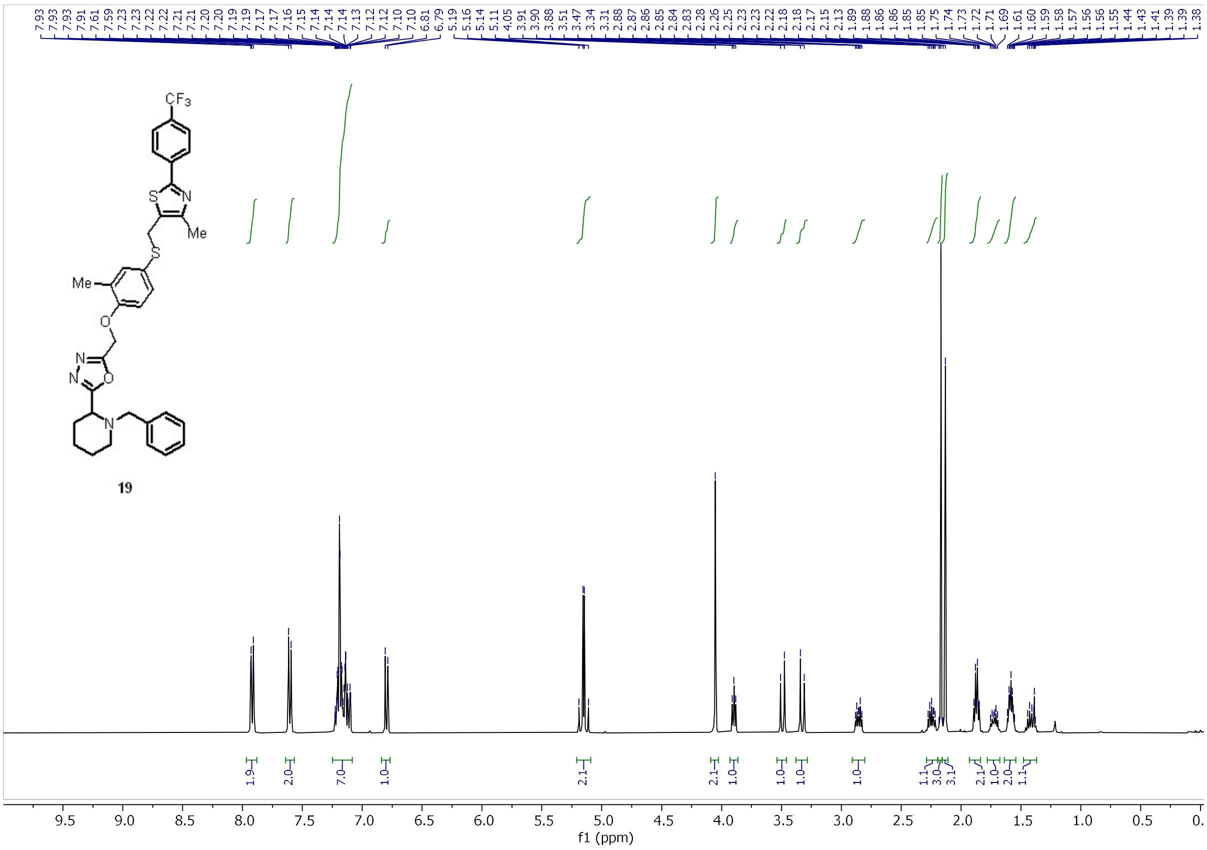

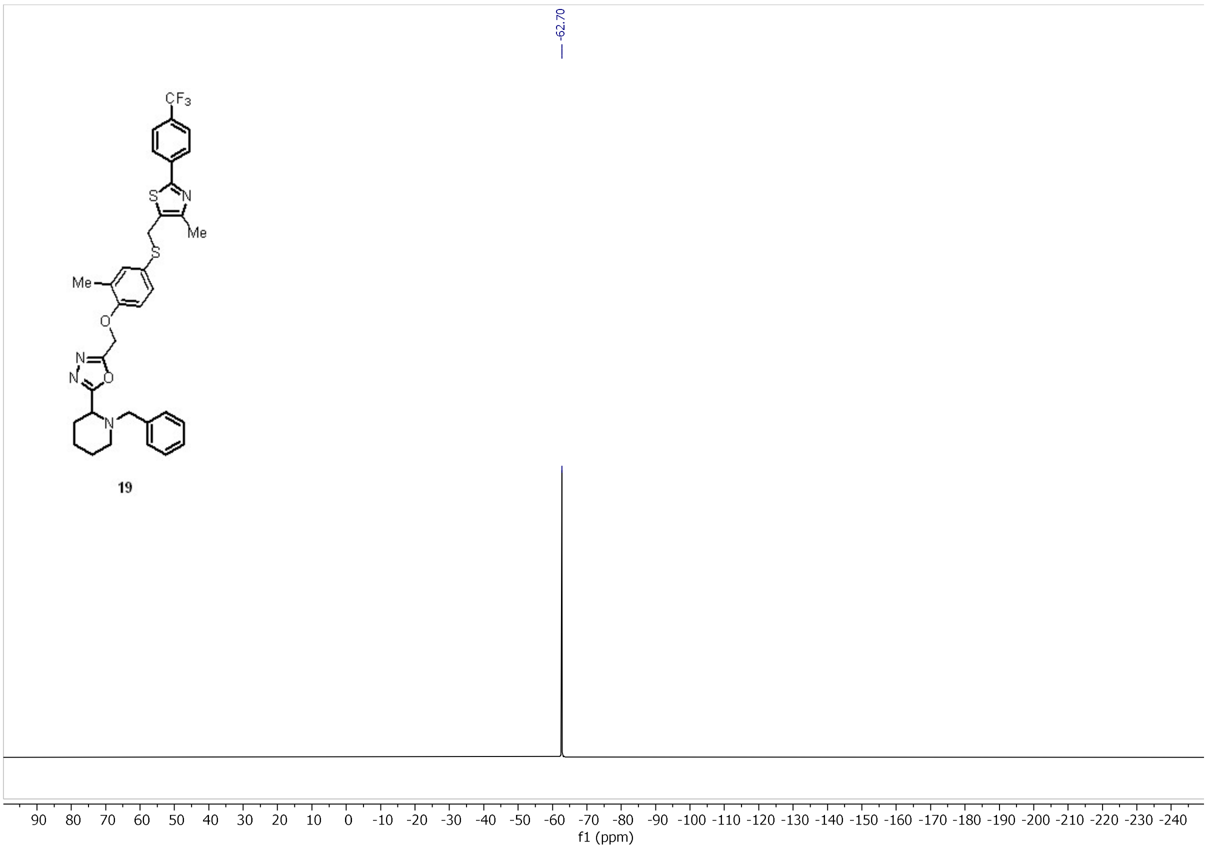

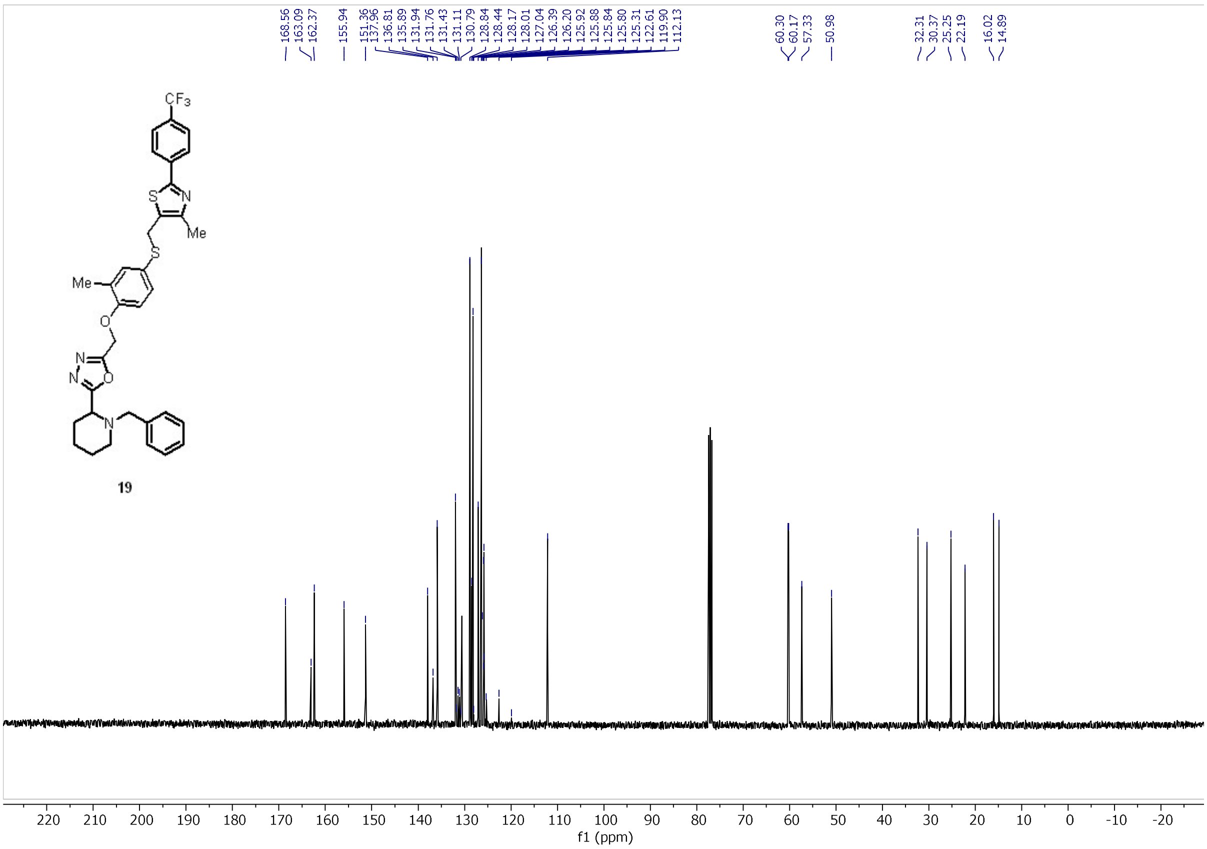

N-(4-((2-(5-(1-benzylpiperidin-2-yl)-1,3,4-oxadiazol-2-yl)propan-2-yl)oxy)phenethyl)-4-chlorobenzamide - 20

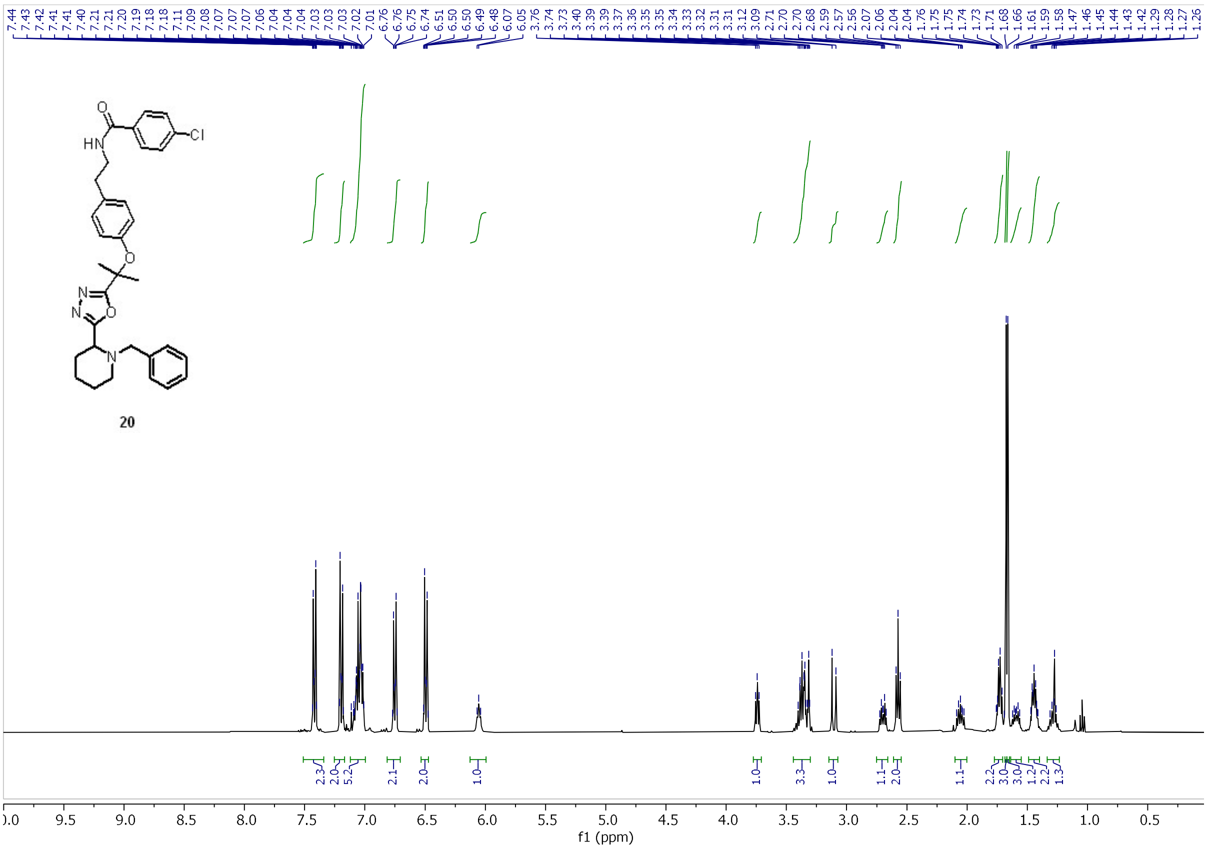

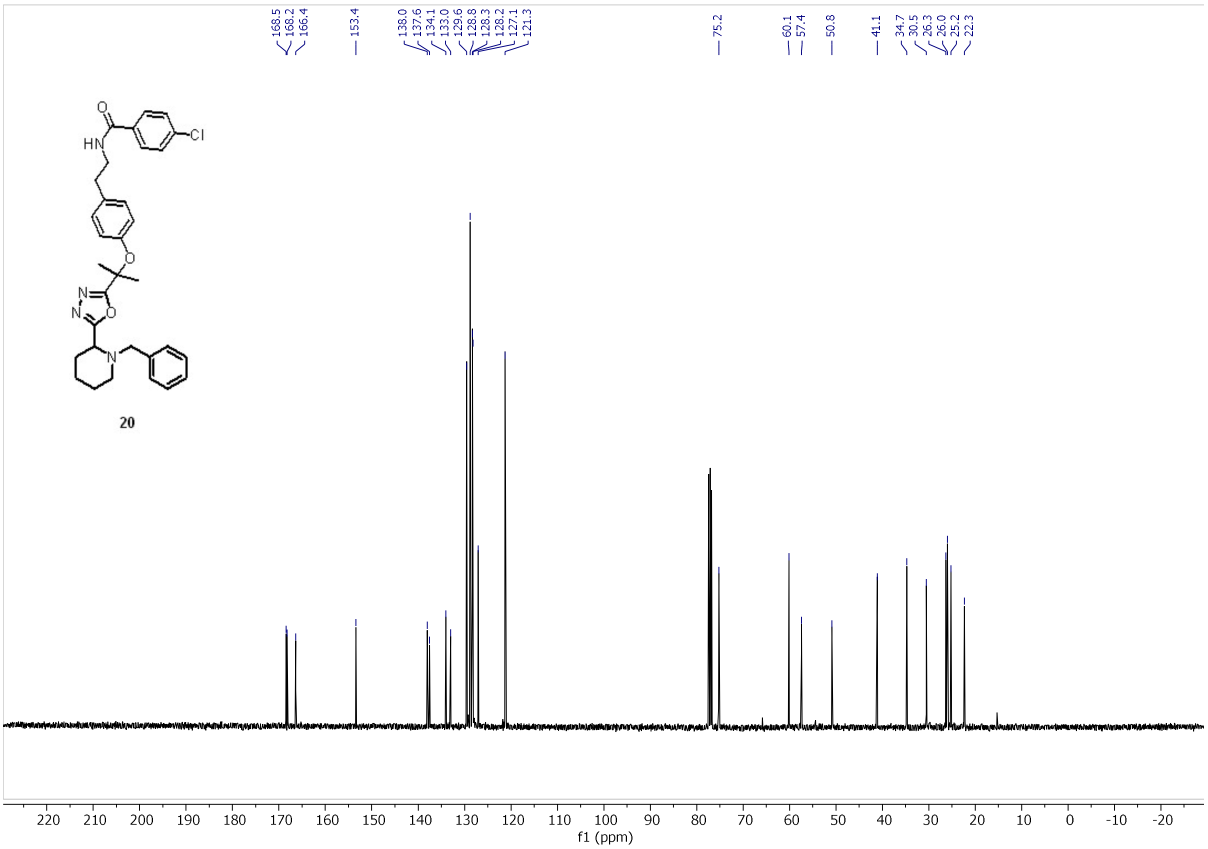

2-(1-benzylpiperidin-2-yl)-5-methyl-1,3,4-thiadiazole - 21

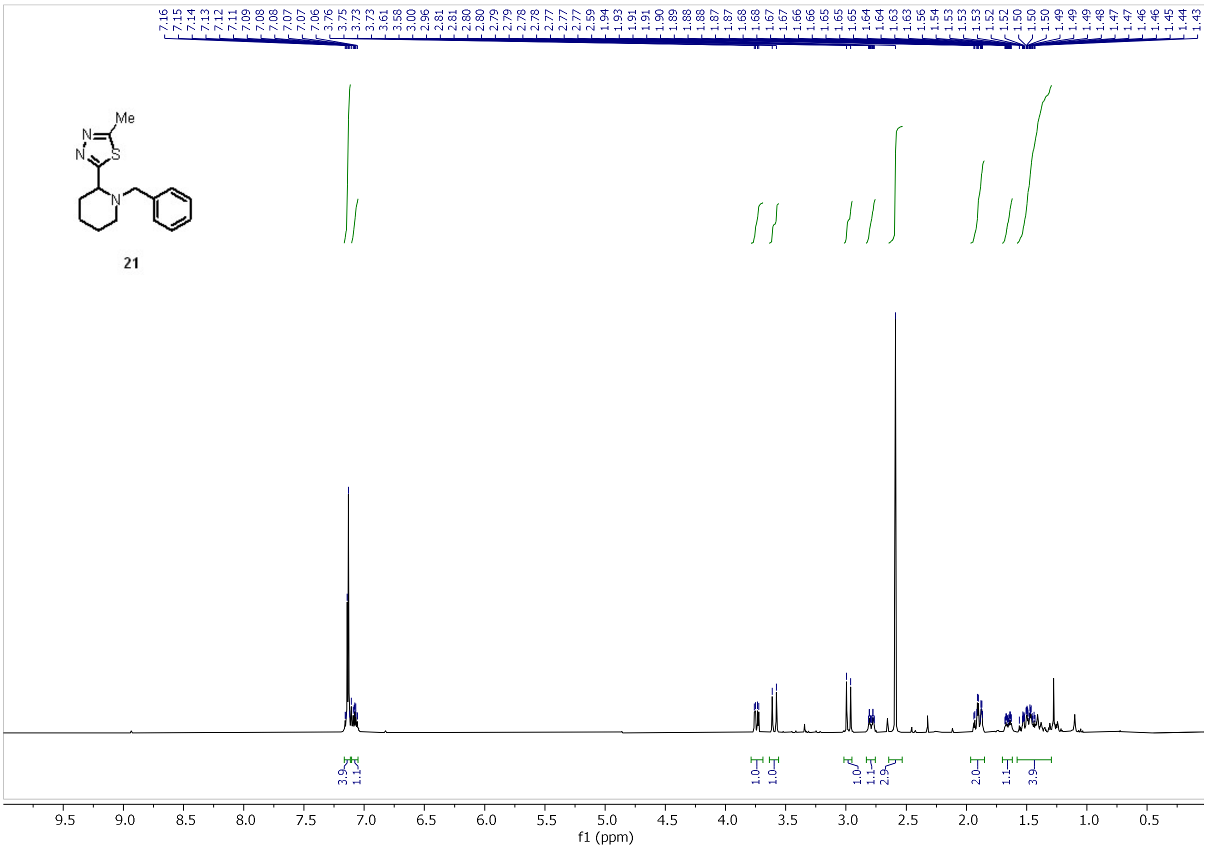

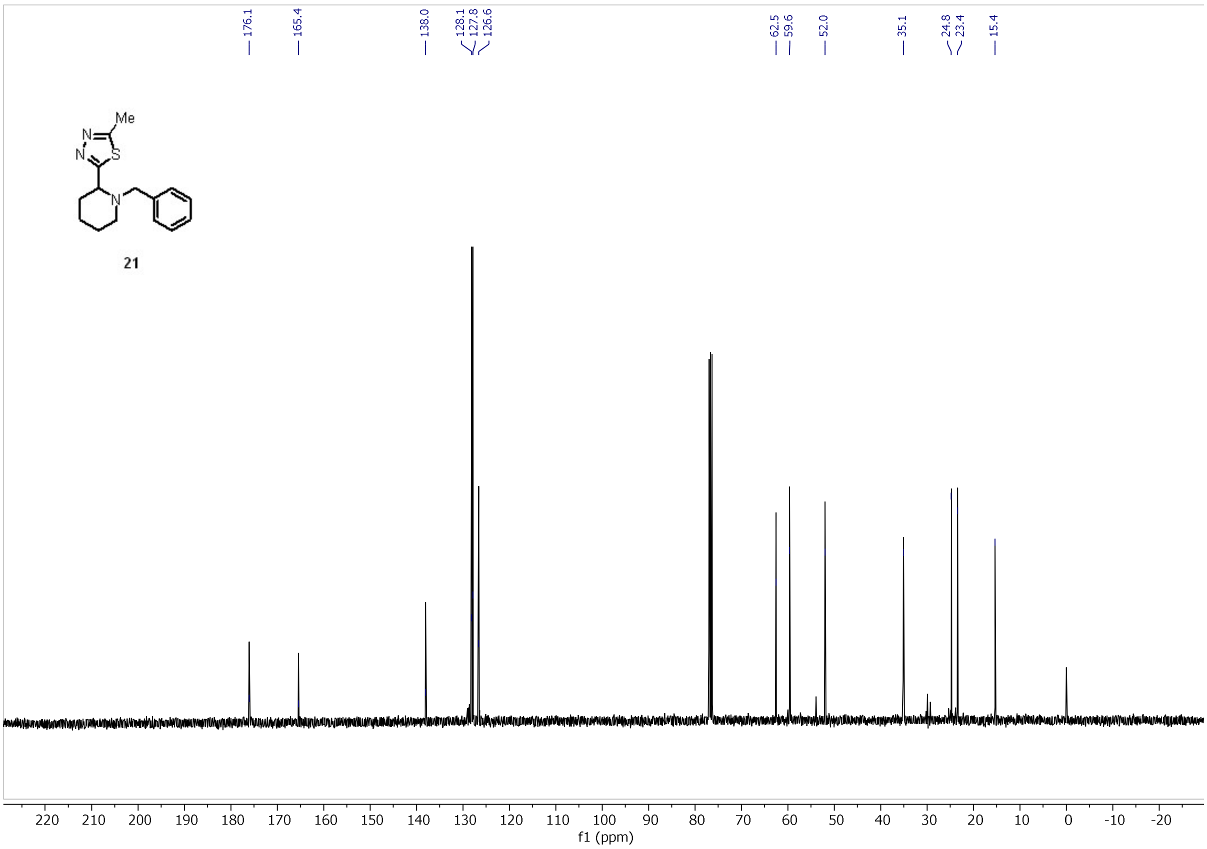

2-(1-benzylpiperidin-2-yl)-5,7-diphenyl-1,3,4-oxadiazepine - 22

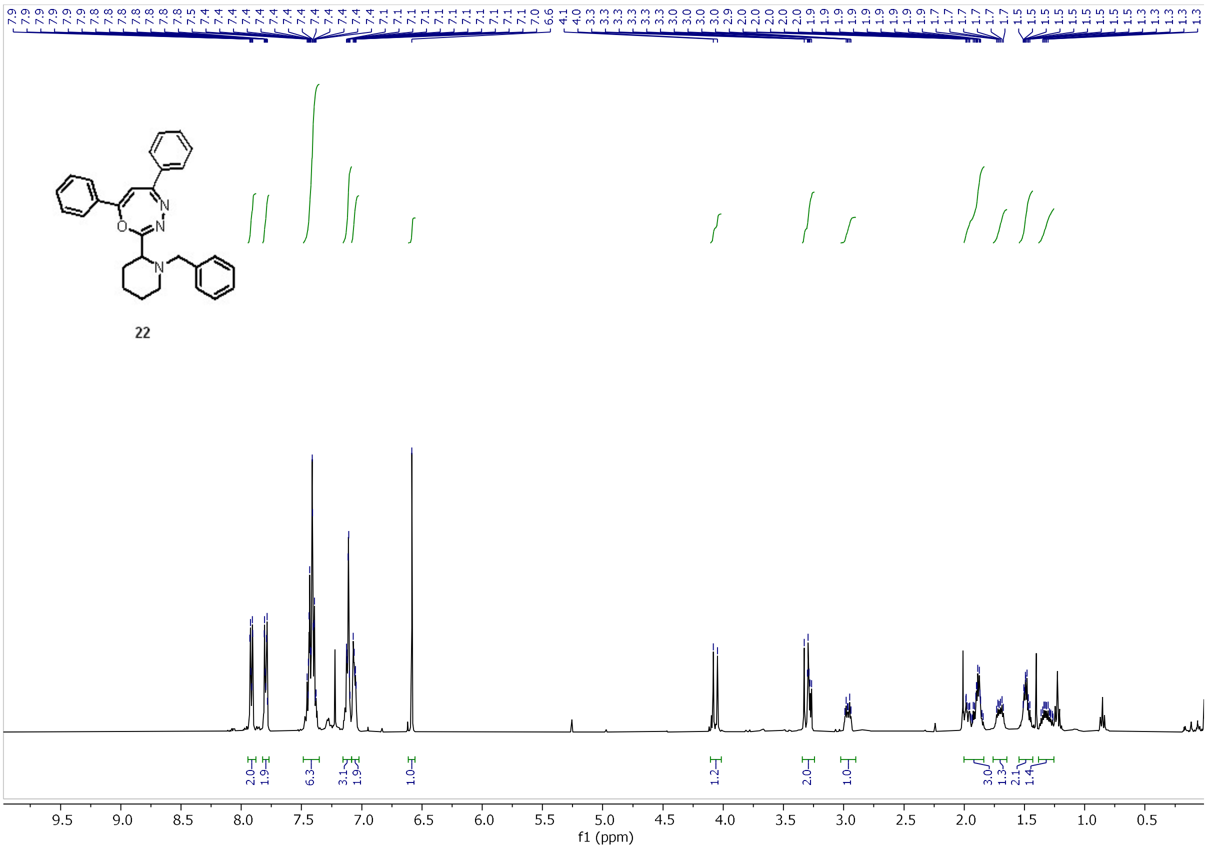

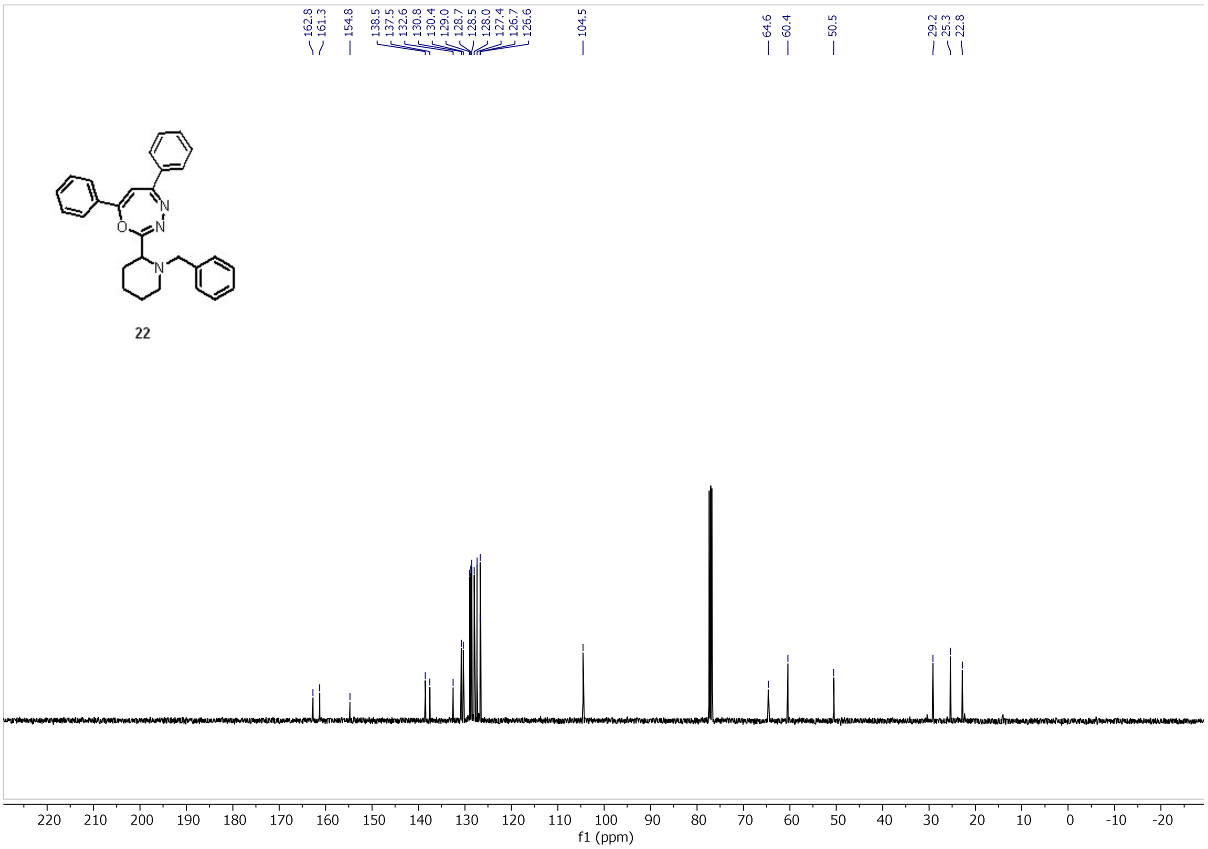

3-(1-benzylpiperidin-2-yl)-[1,2,4]triazolo[4,3-c]quinazoline - 23

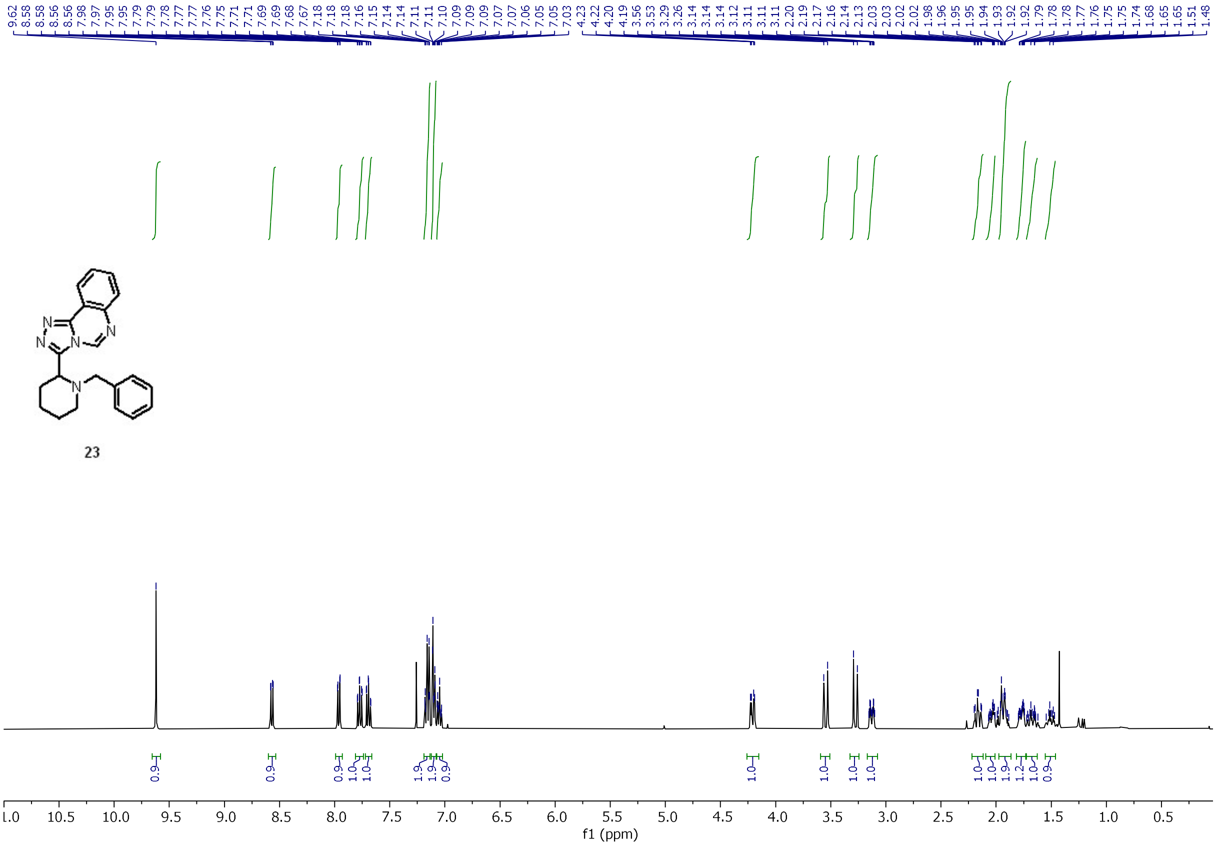

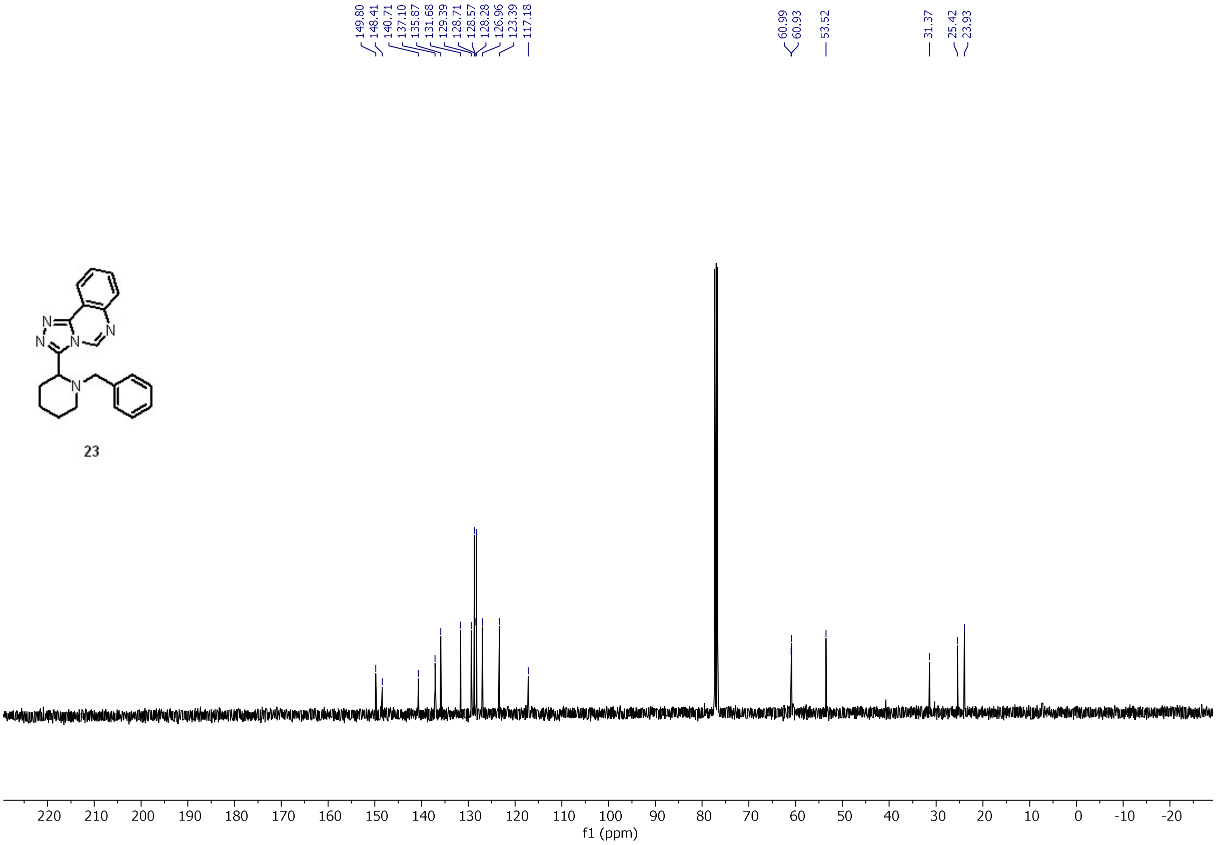

23

1-benzyl-2-(5-phenyl-4-tosyl-4H-1,2,4-triazol-3-yl)piperidine - 24

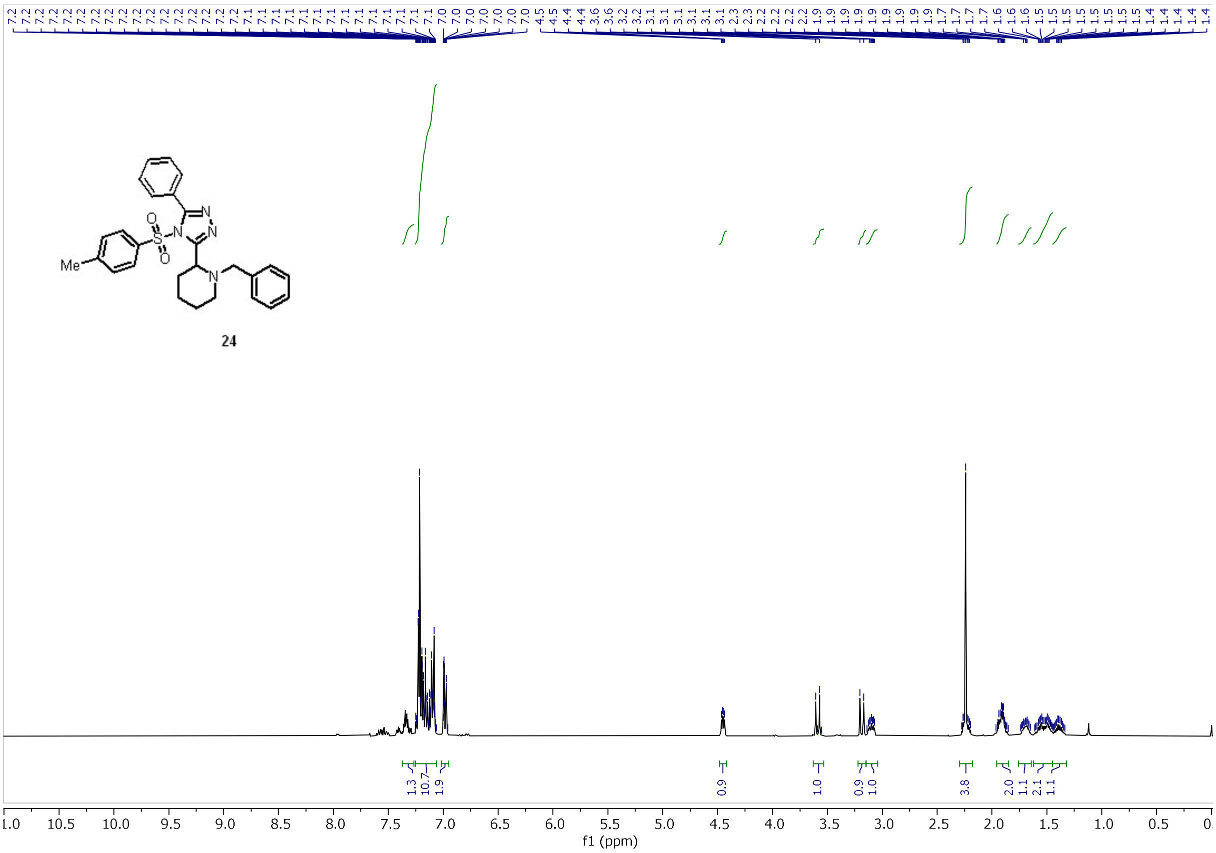

24

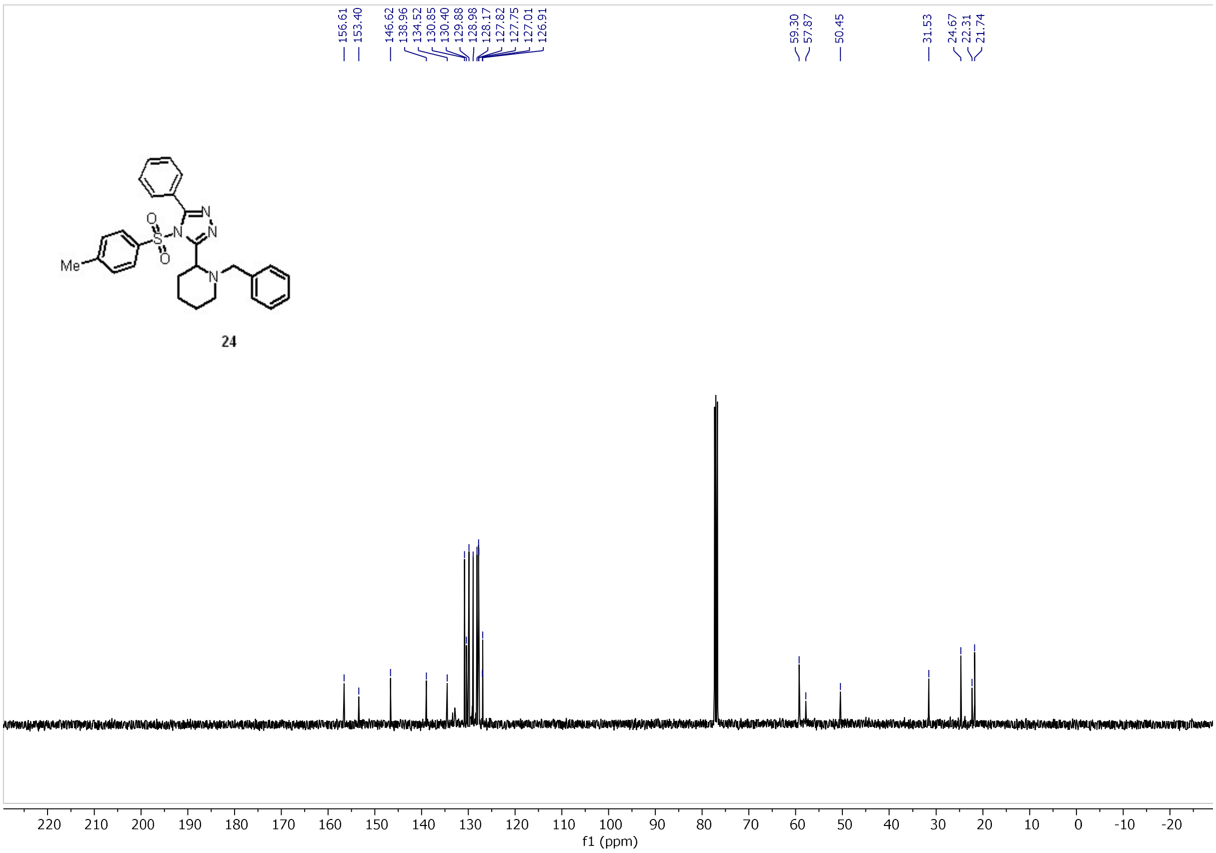

tert-butyl 3-(1-benzylpiperidin-2-yl)-5-phenyl-4H-1,2,4-triazole-4-carboxylate - 25

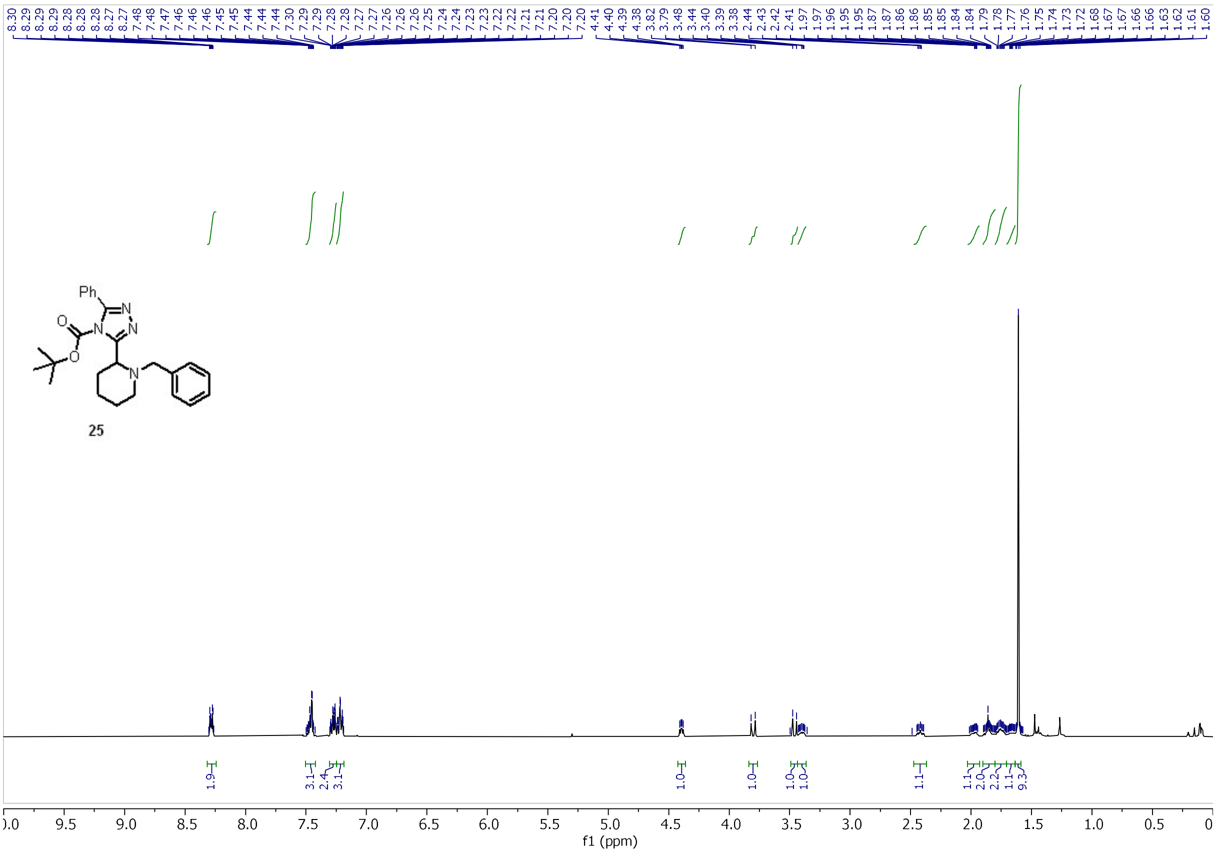

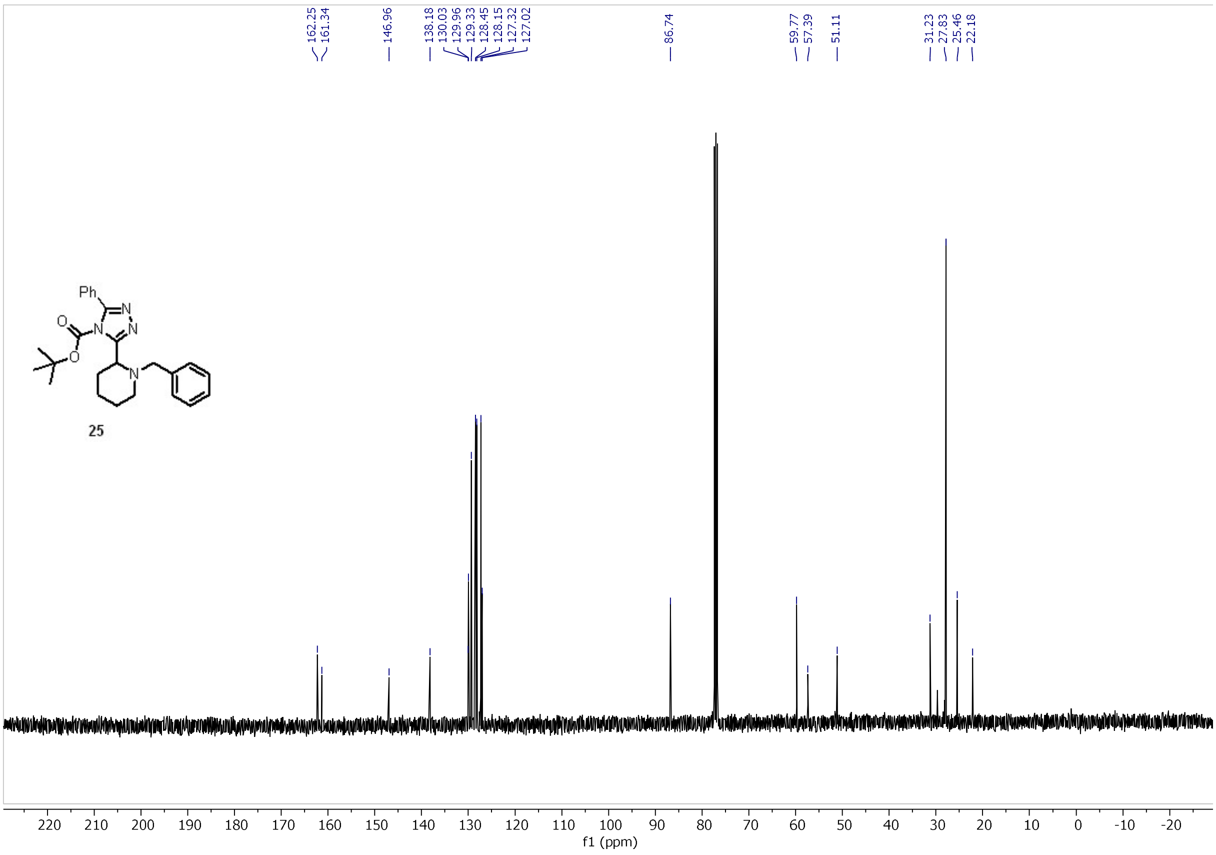

1-benzyl-2-(5-phenyl-4H-1,2,4-triazol-3-yl)piperidine - 27

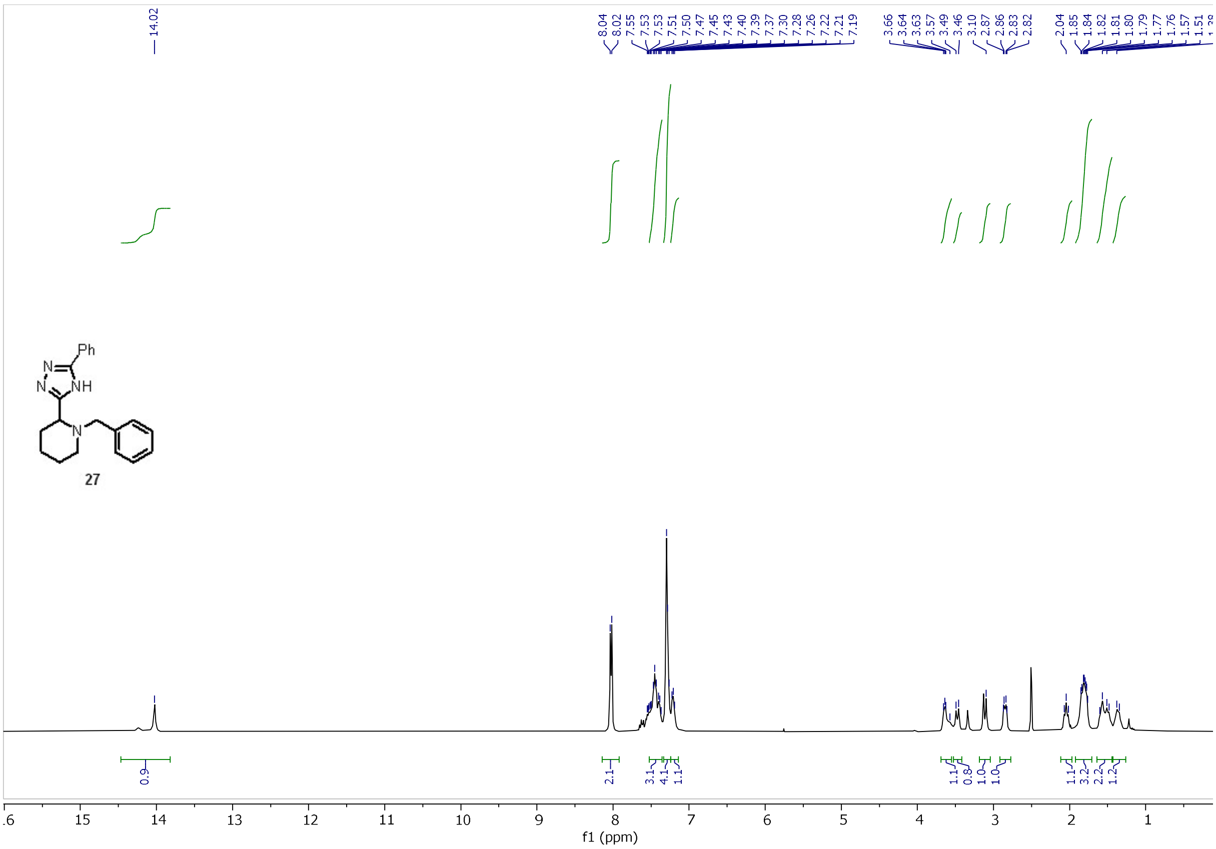

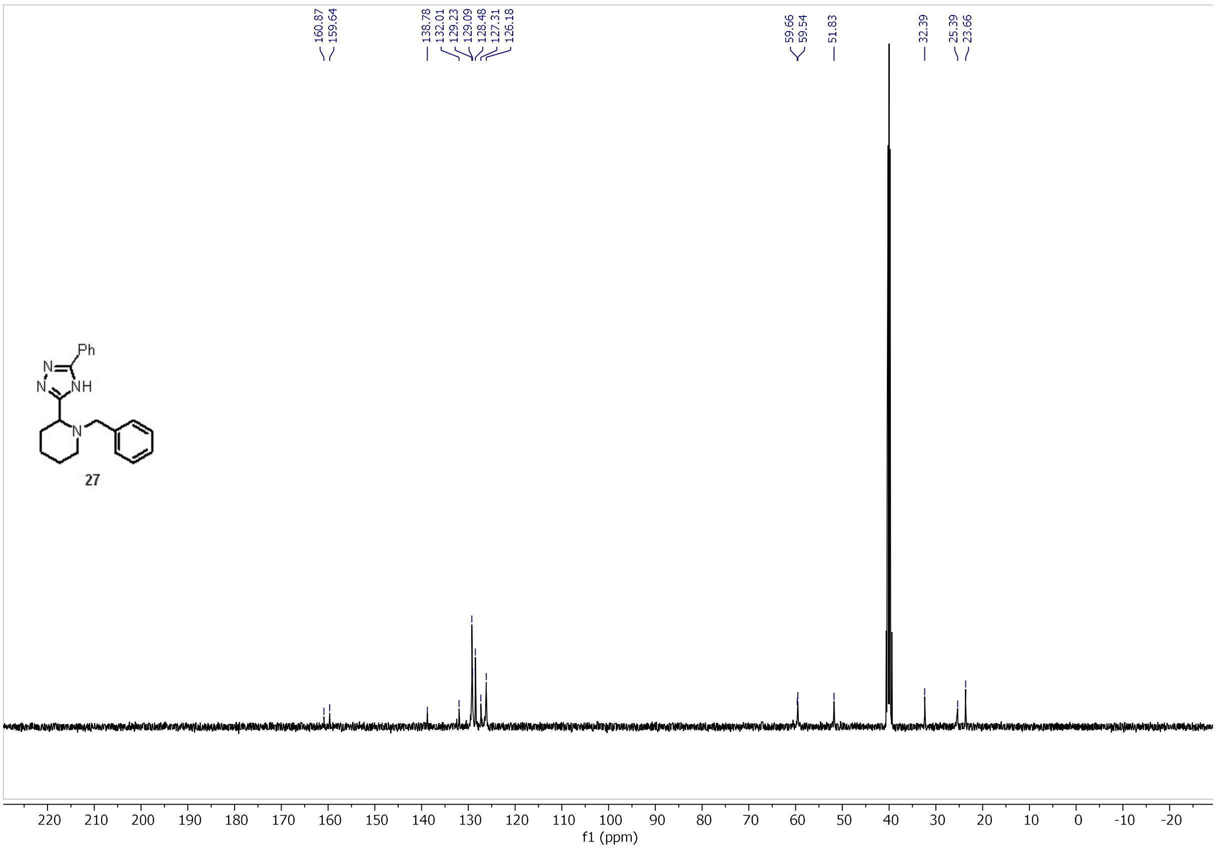

3-(1-benzylpiperidin-2-yl)benzo[4,5]isothiazolo[3,2-c][1,2,4]triazole 5,5-dioxide - 28

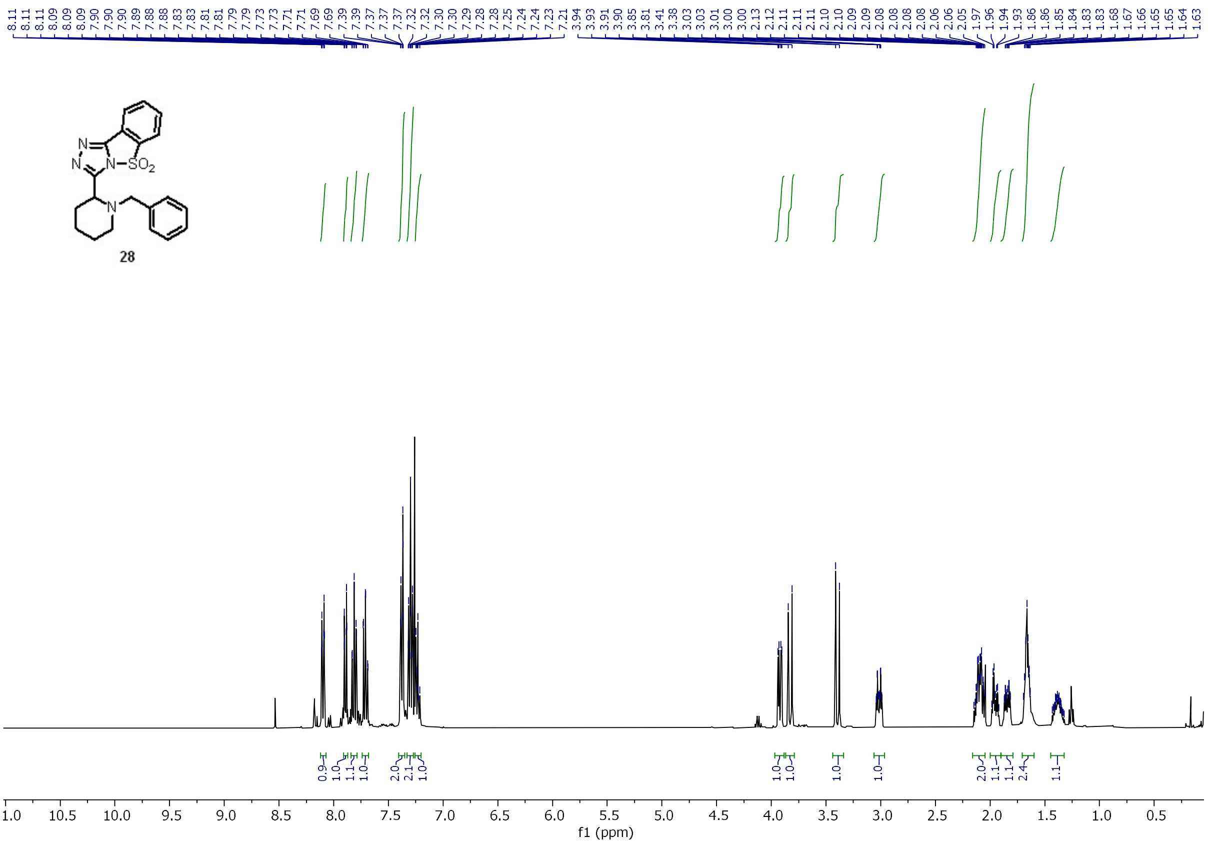

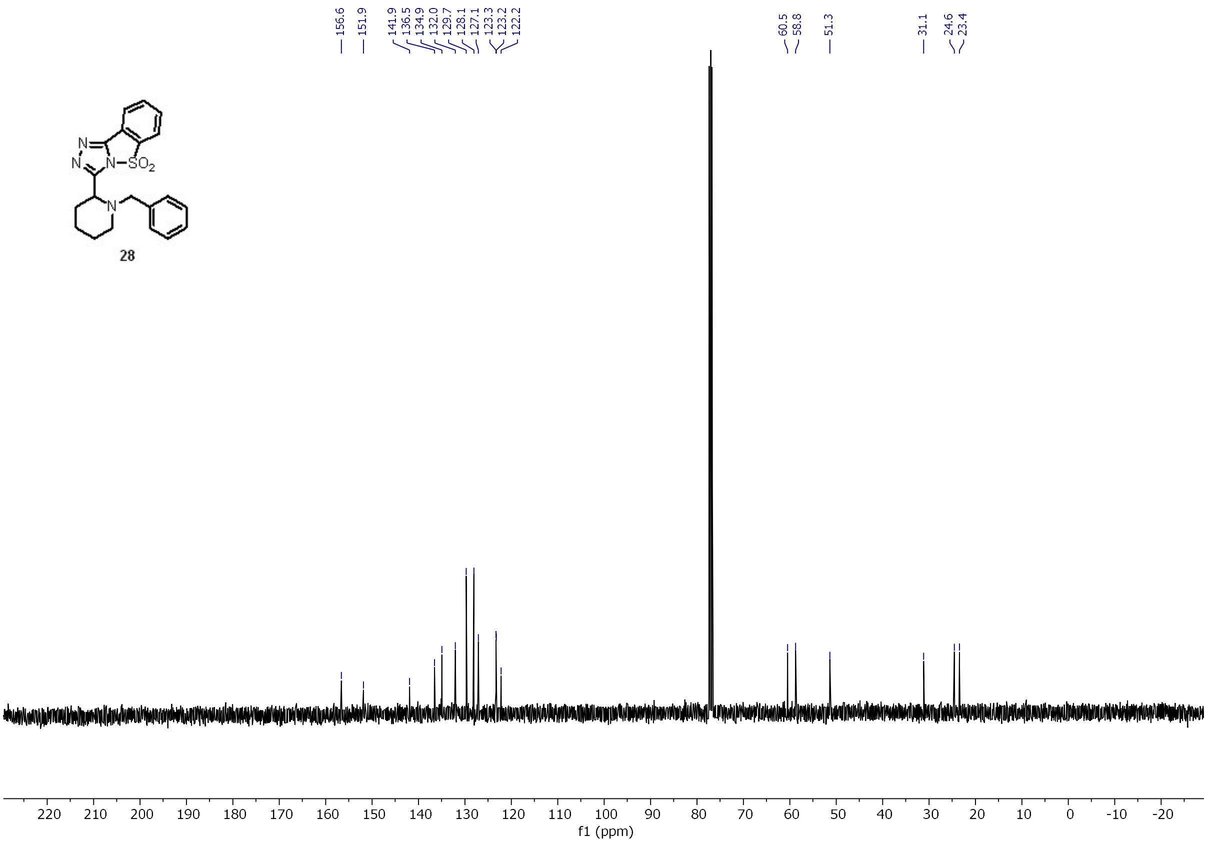

4-((3-(1-benzylpiperidin-2-yl)-5-methyl-4H-1,2,4-triazol-4-yl)sulfonyl)aniline - 29

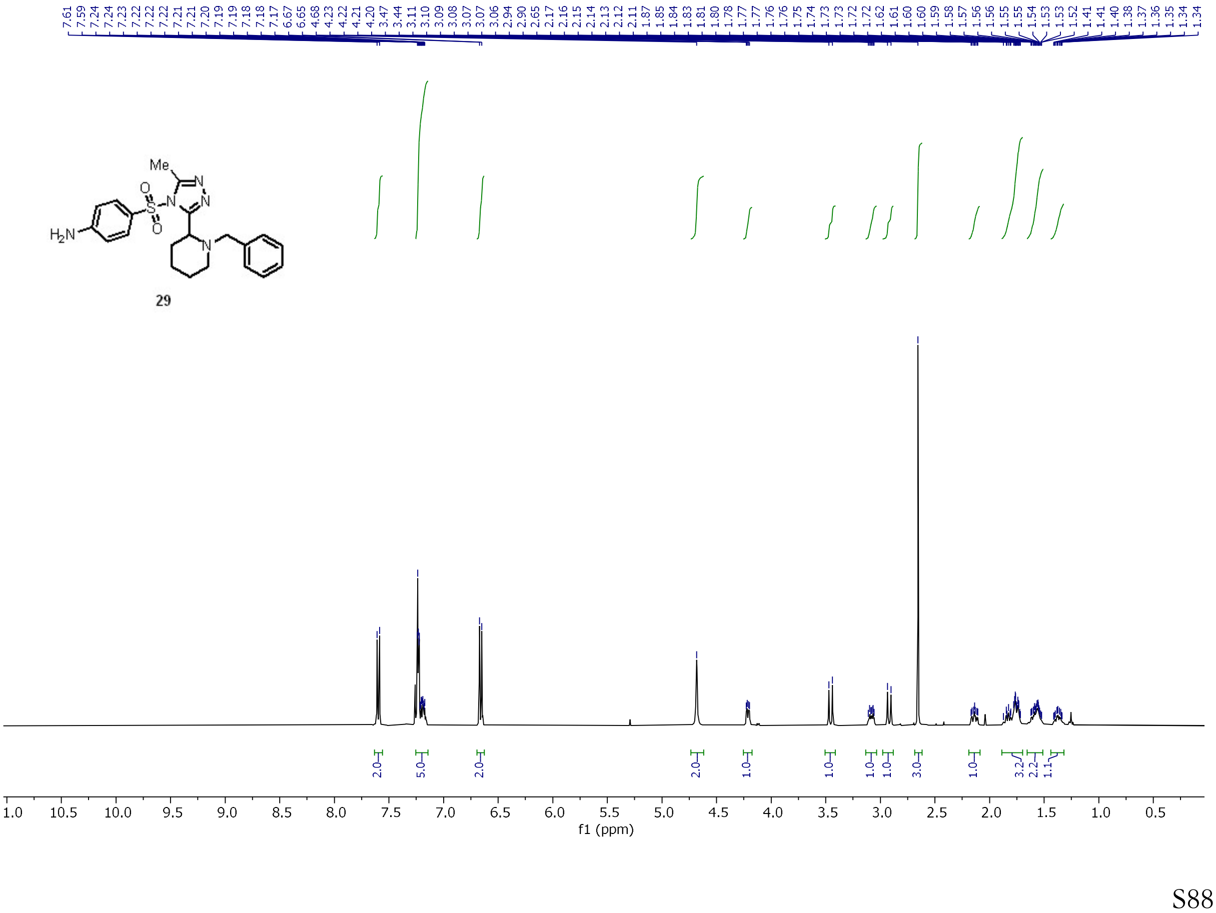

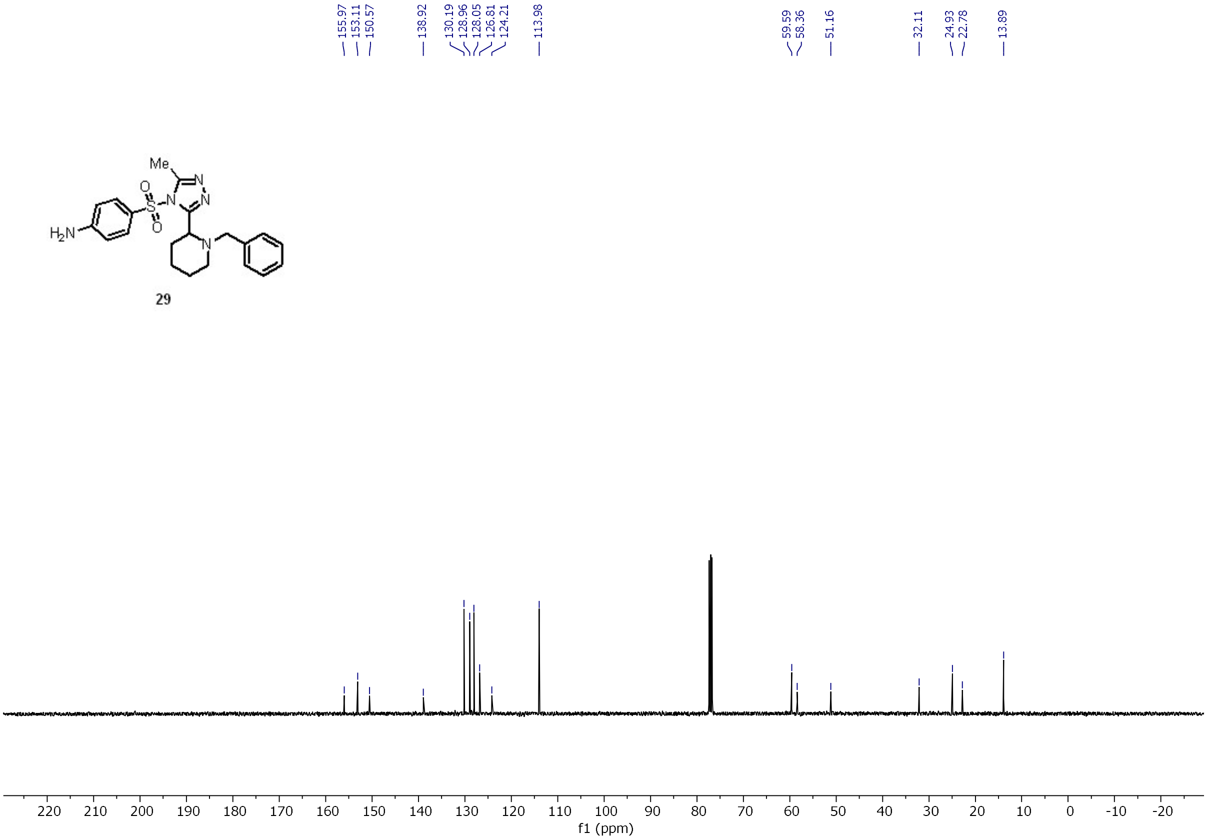

2-(1-benzylpyrrolidin-2-yl)-5-phenyl-1,3,4-oxadiazole - 30

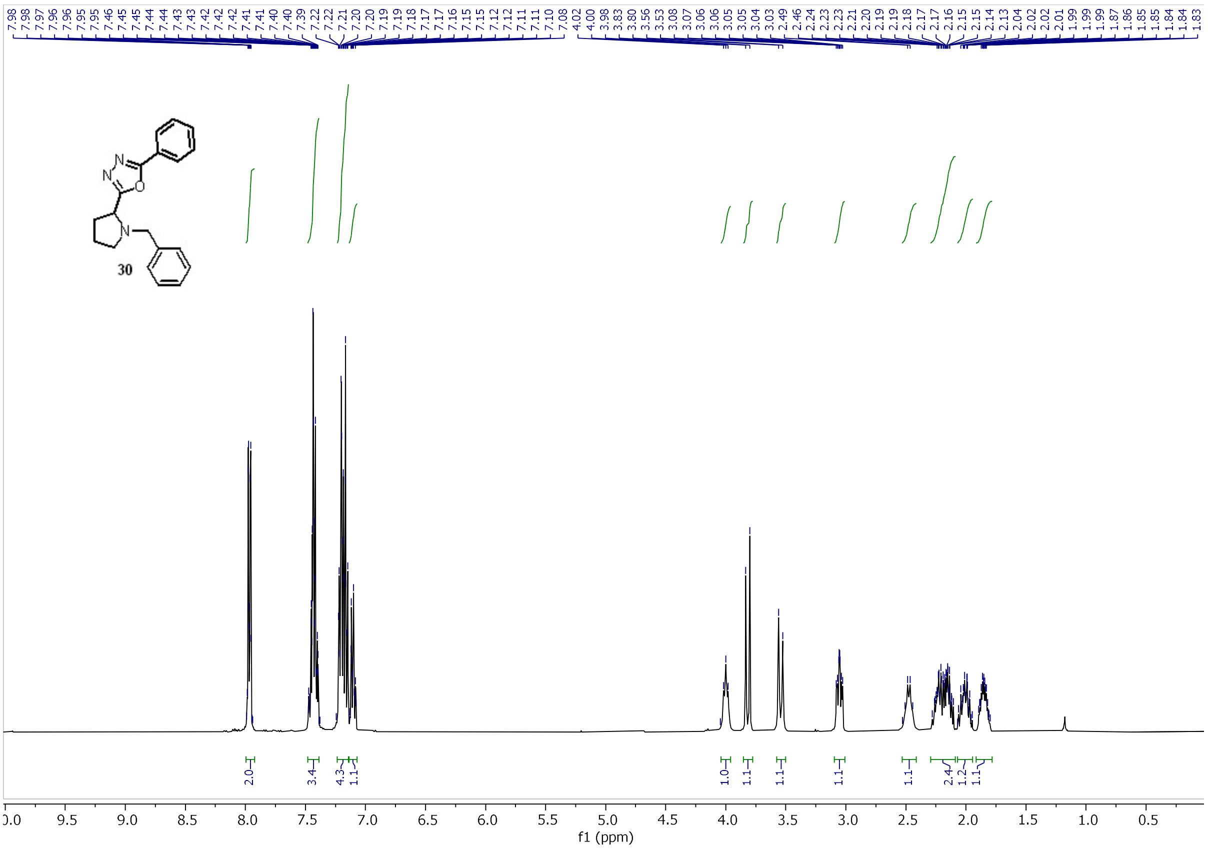

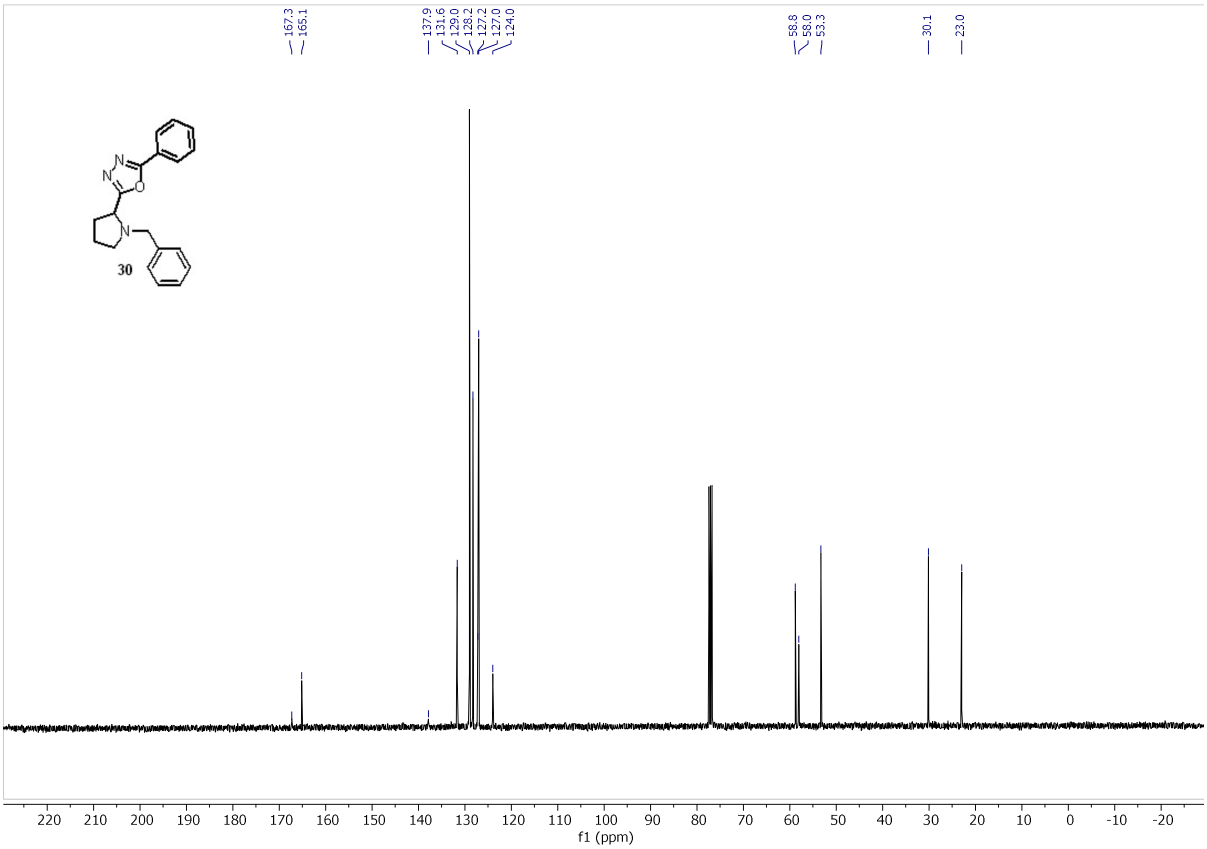

2-(1-methylazepan-2-yl)-5-phenyl-1,3,4-oxadiazole - 31

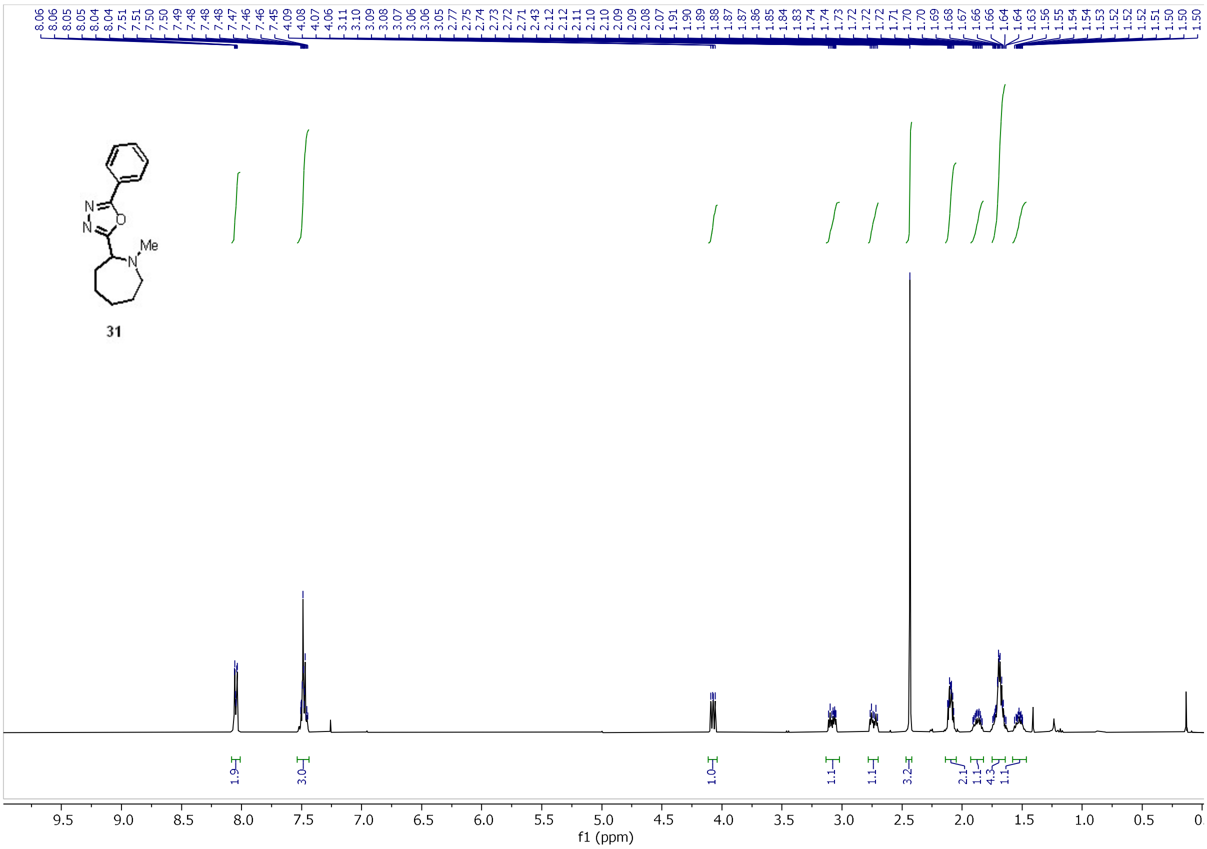

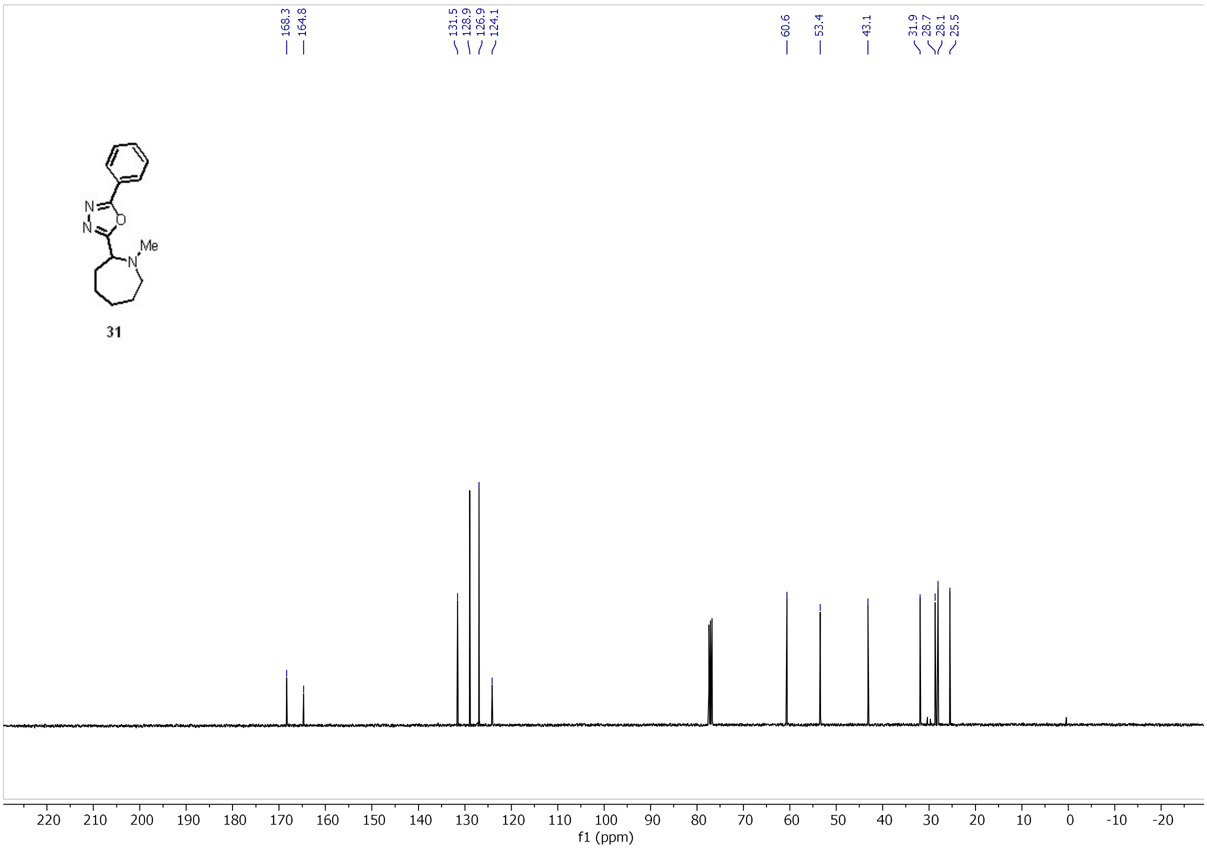

2-(1-(prop-2-yn-1-yl)azepan-2-yl)-5-(4-(trifluoromethoxy)phenyl)-1,3,4-oxadiazole - 32

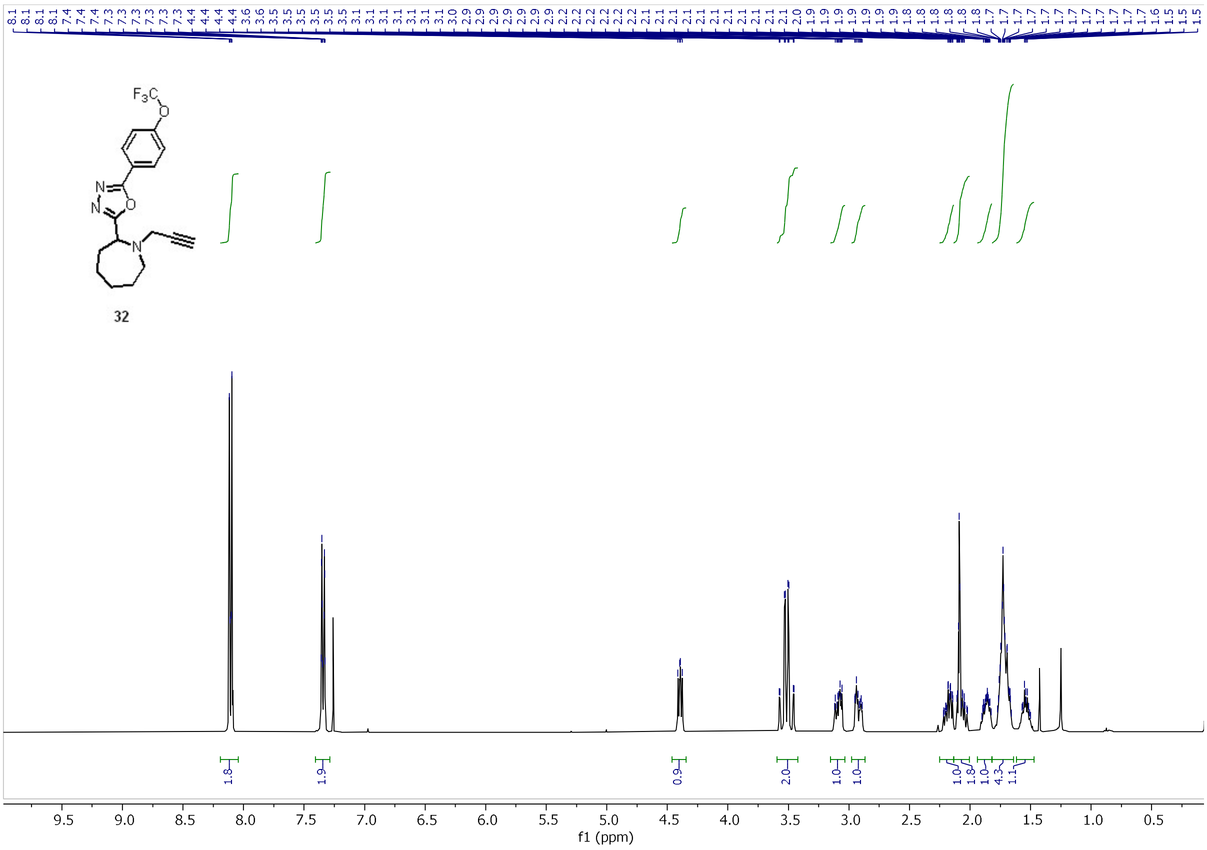

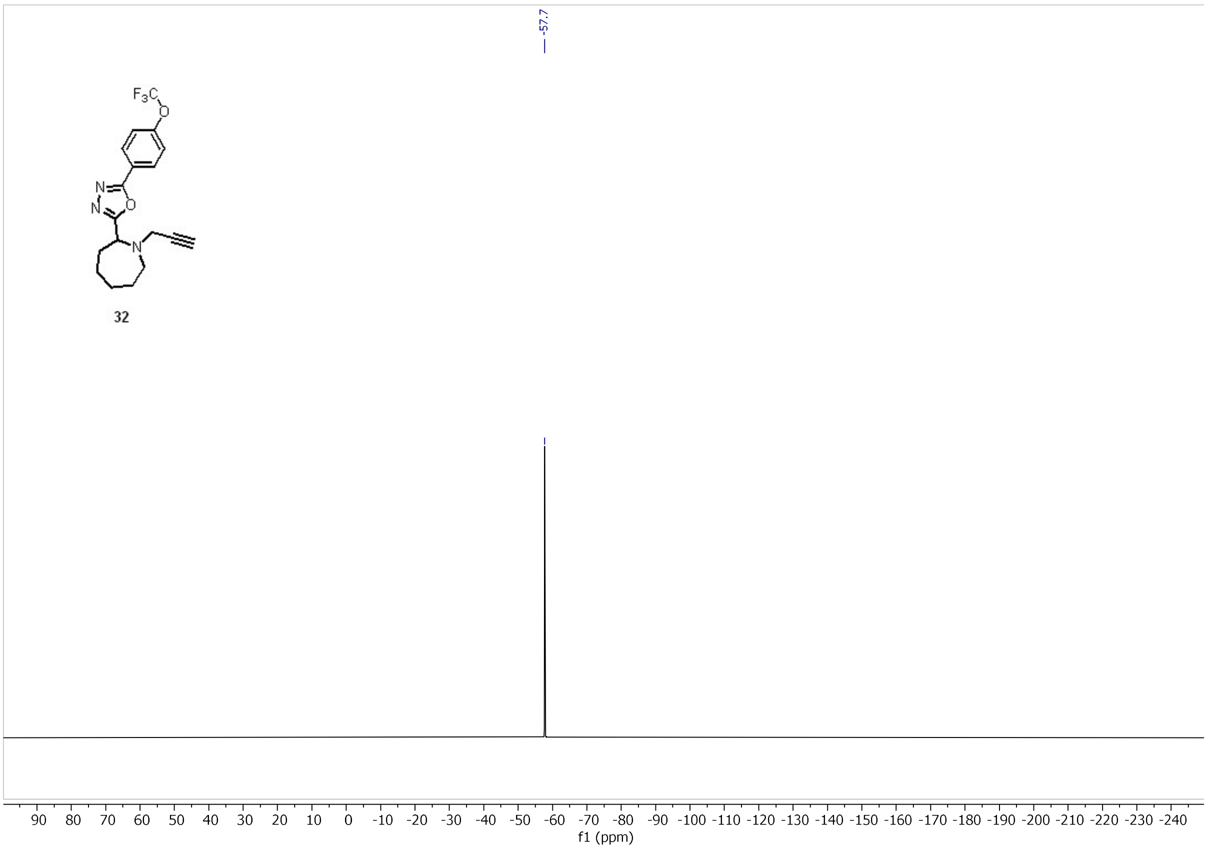

-57.7
F3C
O
N
N
O
N
32
90 80 70 60 50 40 30 20 10 0 -10 -20 -30 -40 -50 -60 -70 -80 -90 -100 -110 -120 -130 -140 -150 -160 -170 -180 -190 -200 -210 -220 -230 -240
f1 (ppm)

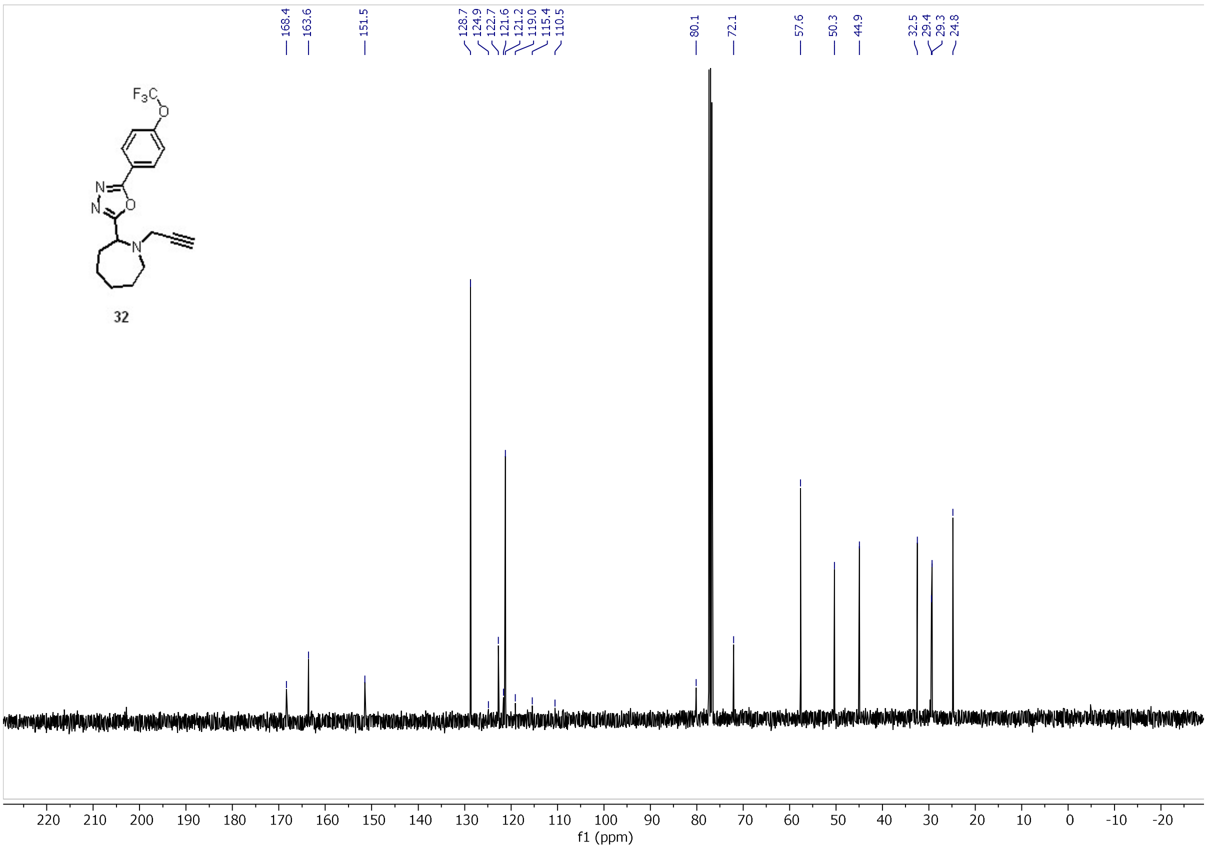

168.4
163.6
151.5
128.7
124.9
122.7
121.6
121.2
119.0
115.4
110.5
80.1
72.1
57.6
50.3
44.9
32.5
29.4
29.3
24.8
F3C
O
N
N
O
N
32
220 210 200 190 180 170 160 150 140 130 120 110 100 90 80 70 60 50 40 30 20 10 0 -10 -20
f1 (ppm)

2-(1-allylazocan-2-yl)-5-(2,3-dihydrobenzo[b][1,4]dioxin-6-yl)-1,3,4-oxadiazole - 33

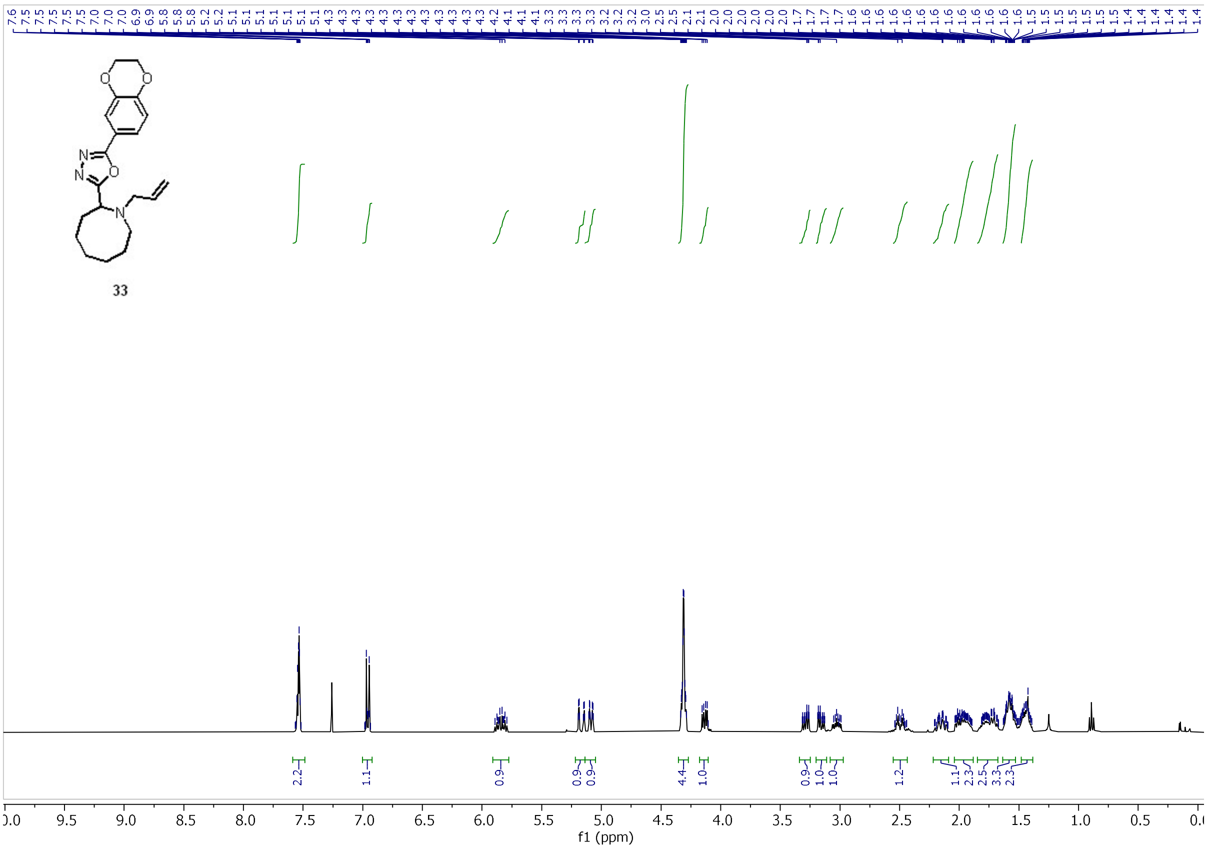

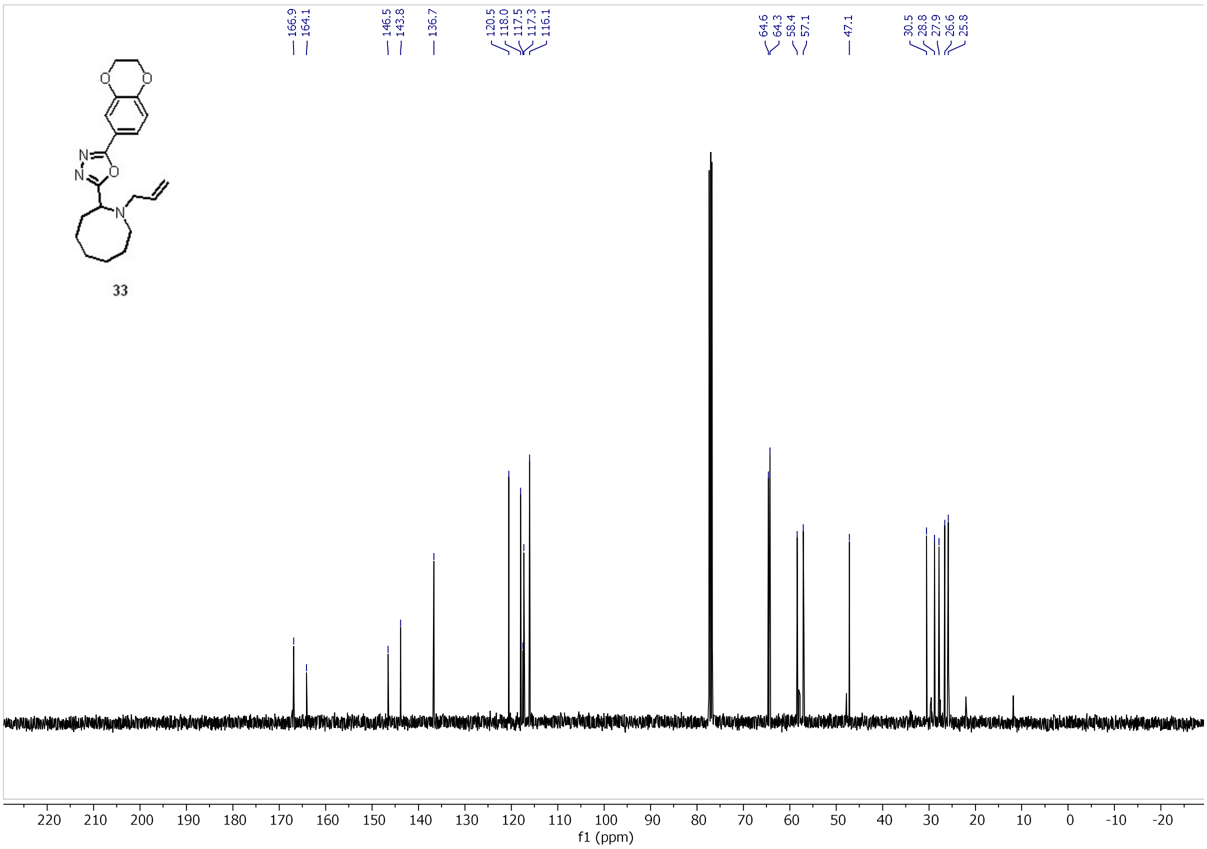

N-((4-fluorophenyl)(5-(thiophen-2-yl)-1,3,4-oxadiazol-2-yl)methyl)-N-methylaniline - 34

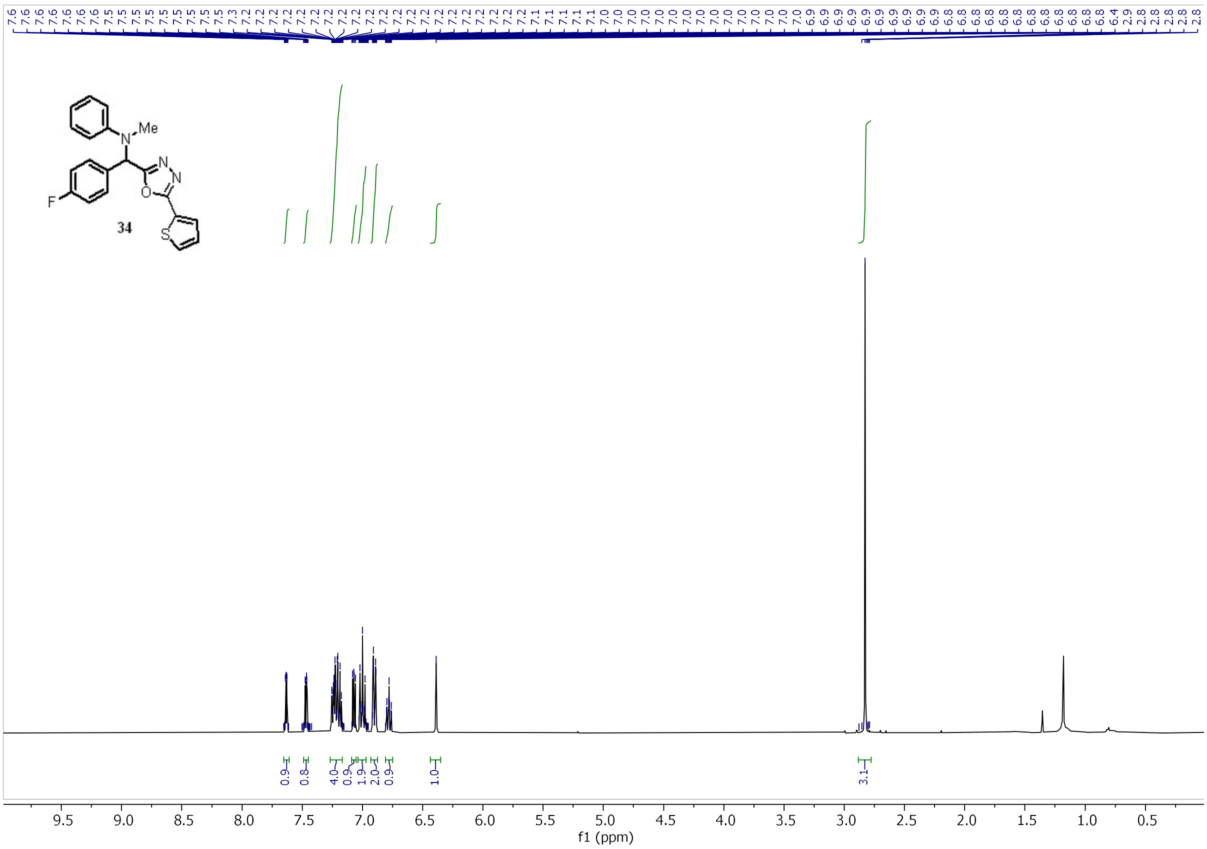

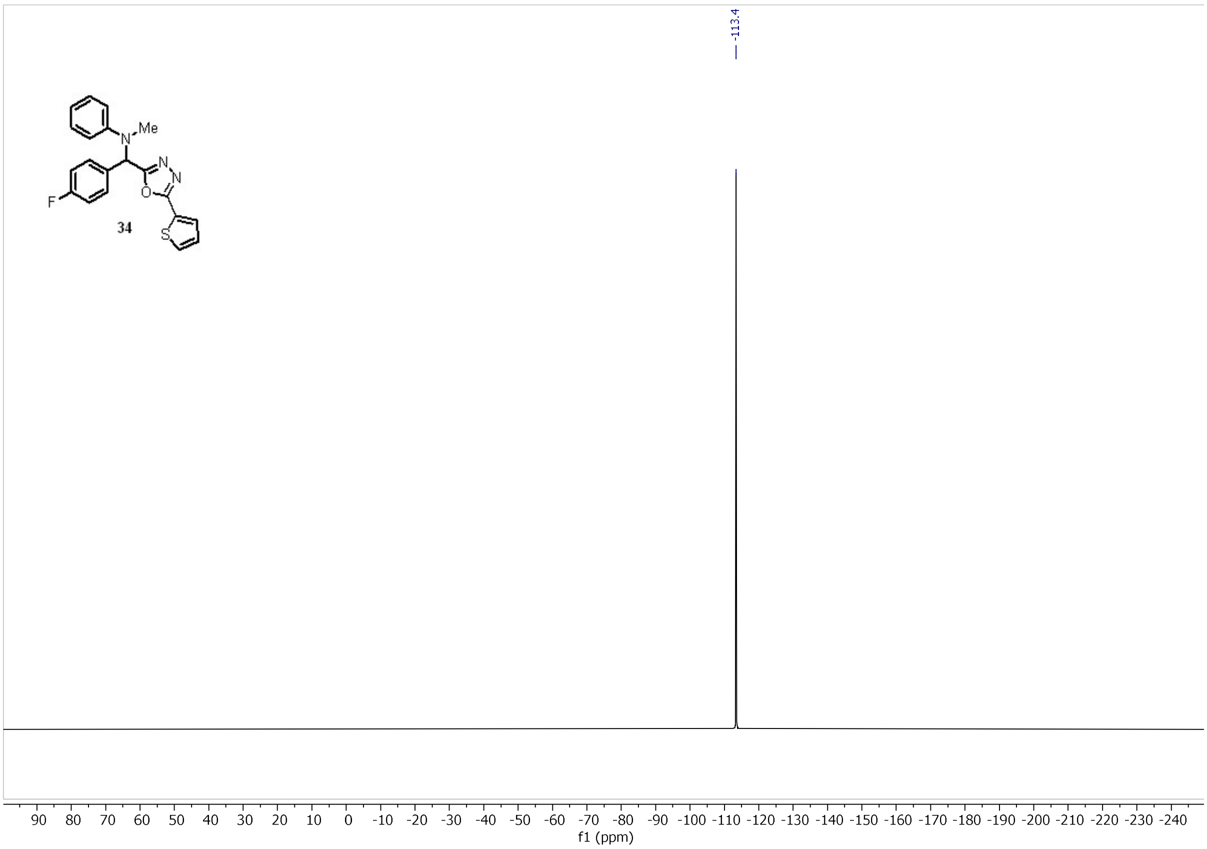

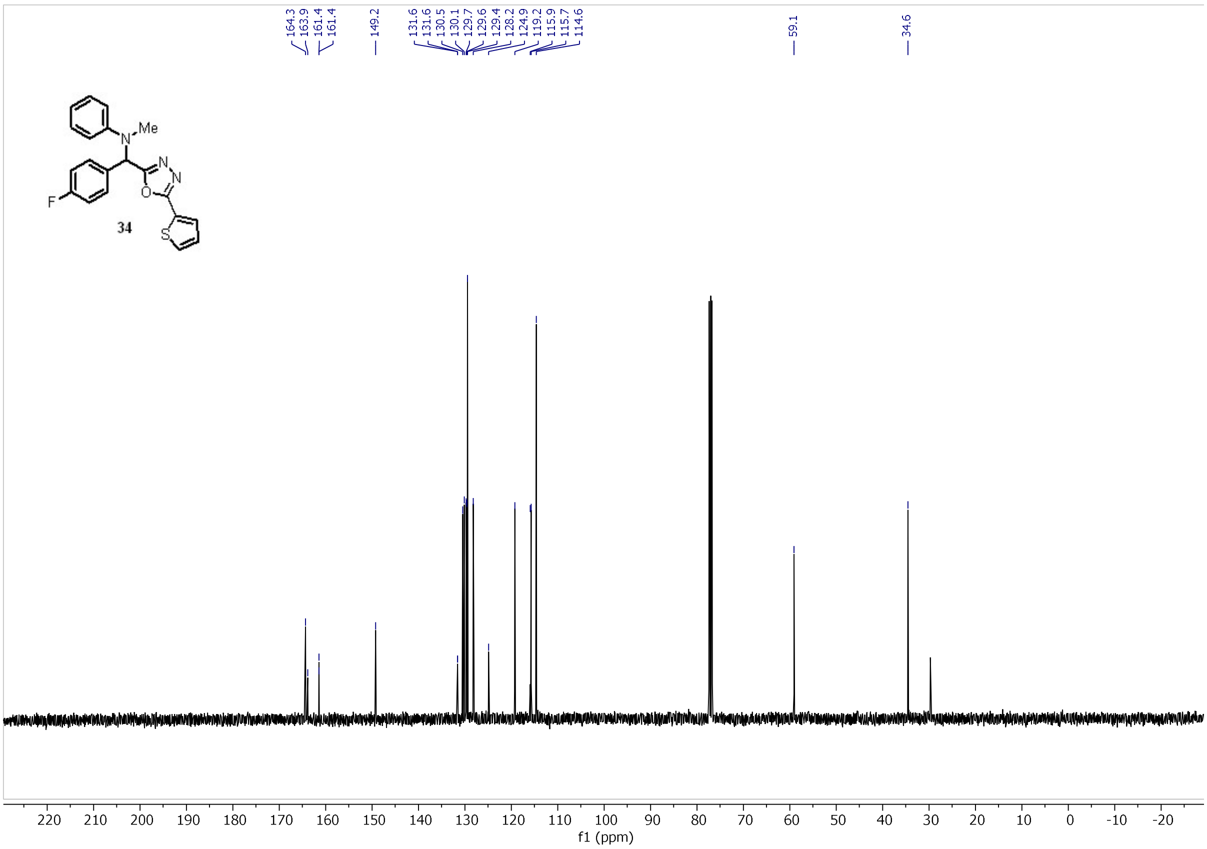

N-((5-(furan-2-yl)-1,3,4-oxadiazol-2-yl)(4-methoxyphenyl)methyl)-N-methylaniline - 35

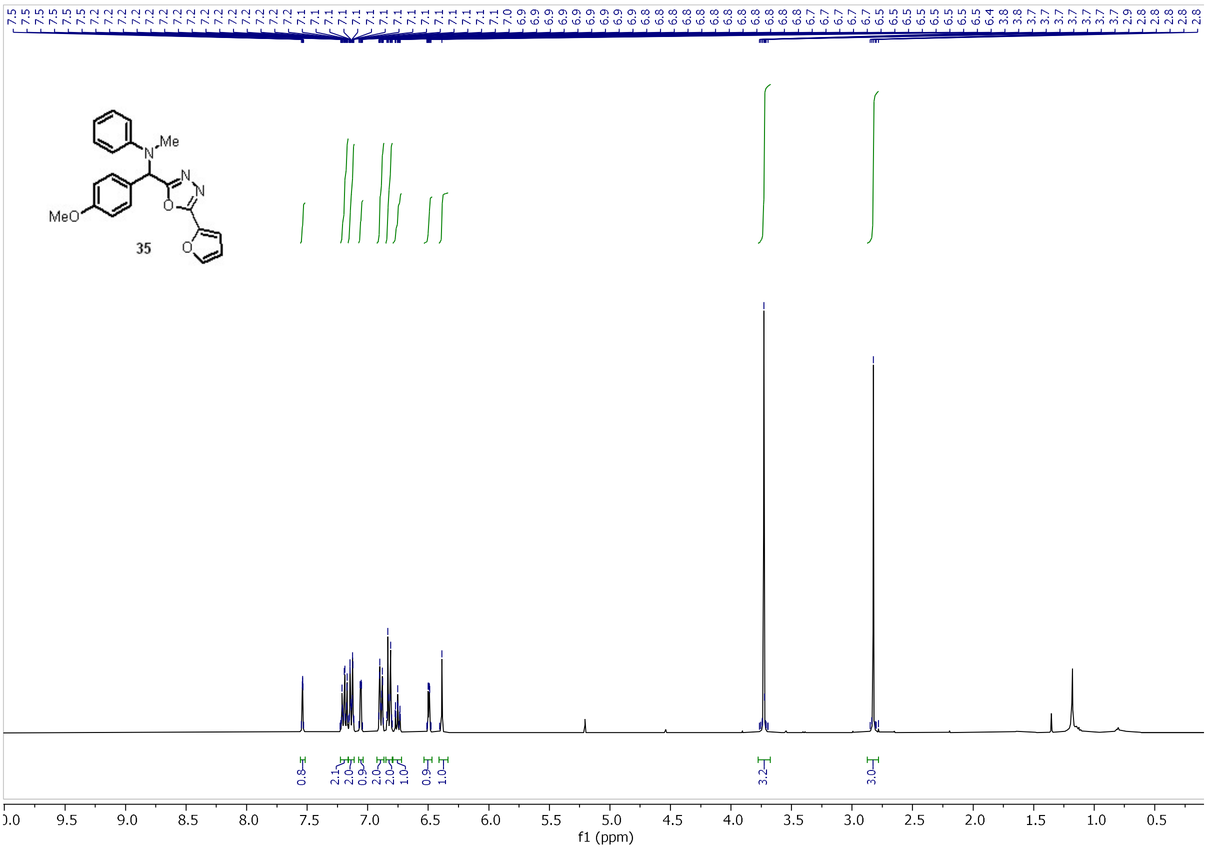

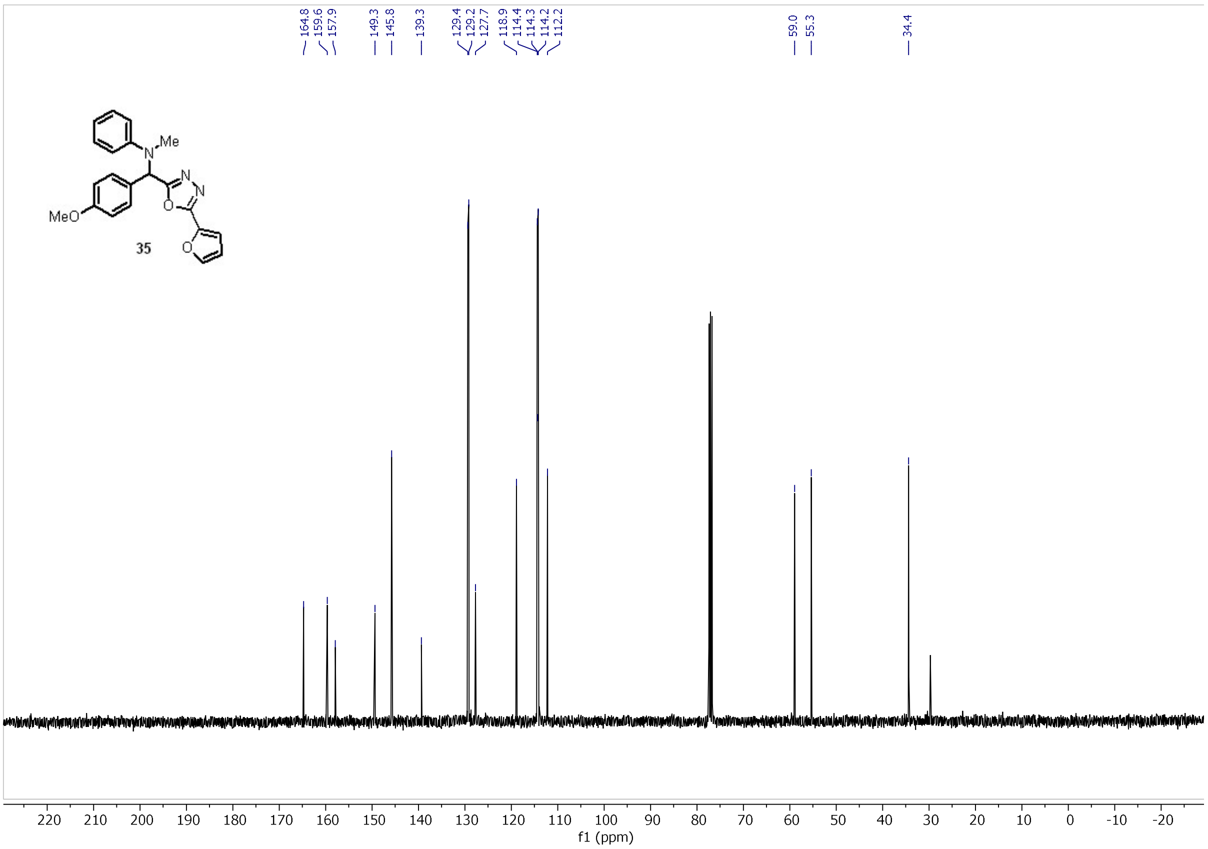

4-(5-((4-fluorophenyl)(indolin-1-yl)methyl)-1,3,4-oxadiazol-2-yl)benzonitrile - 36

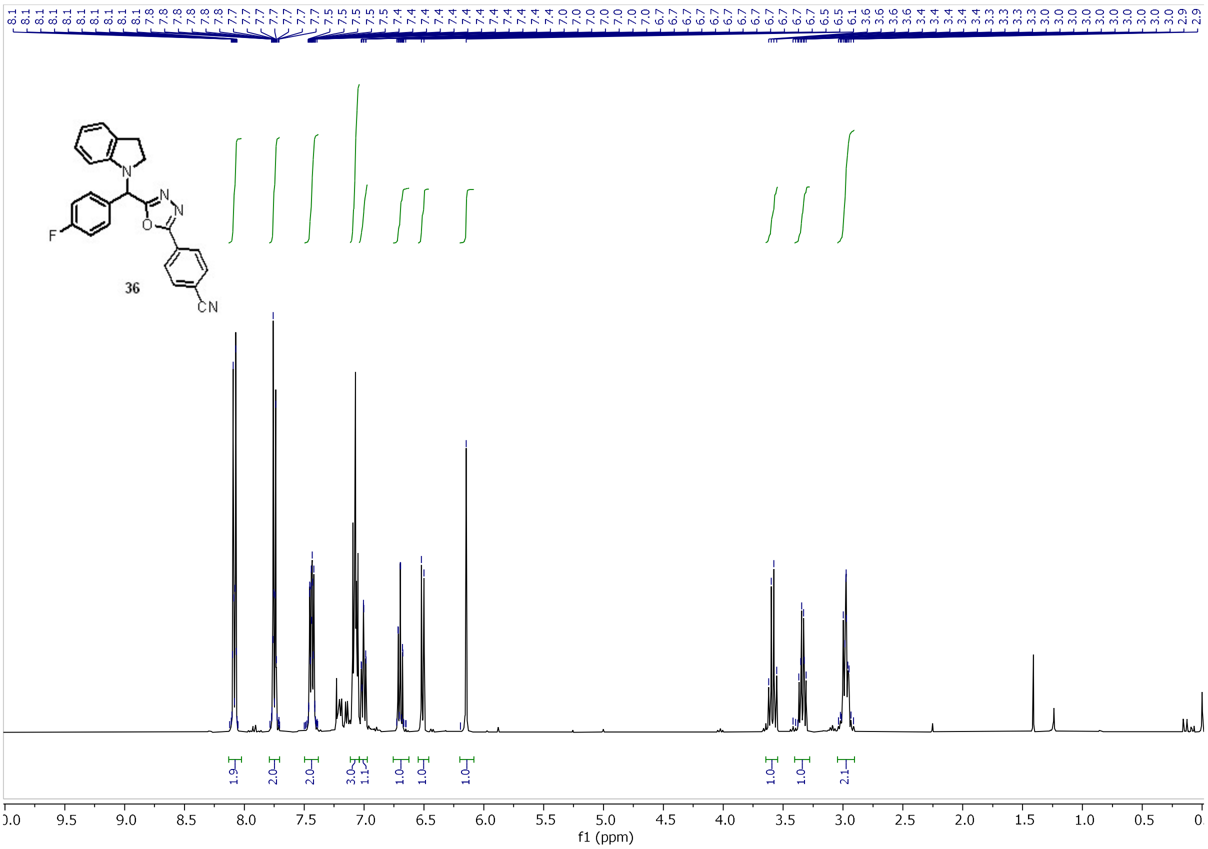

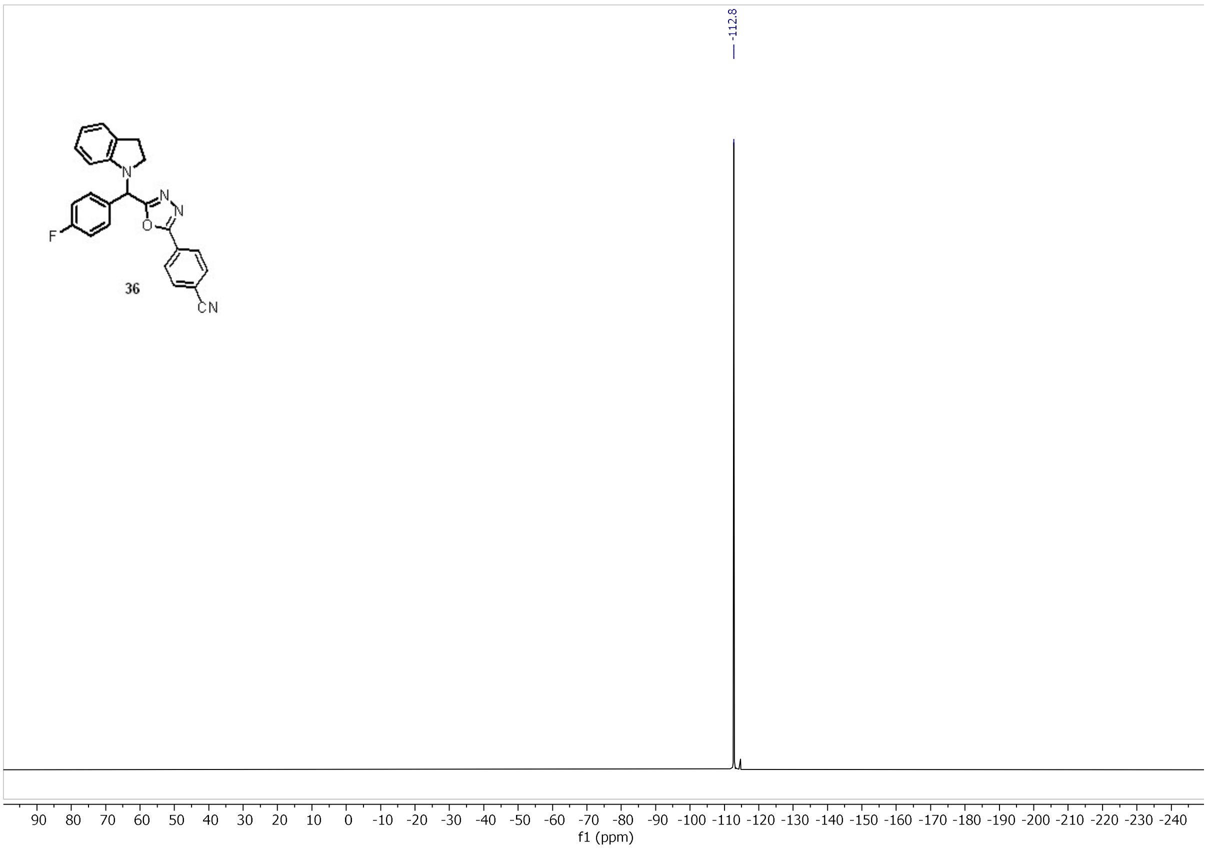

-112.8
F
N
N
N
O
36
CN
f1 (ppm)

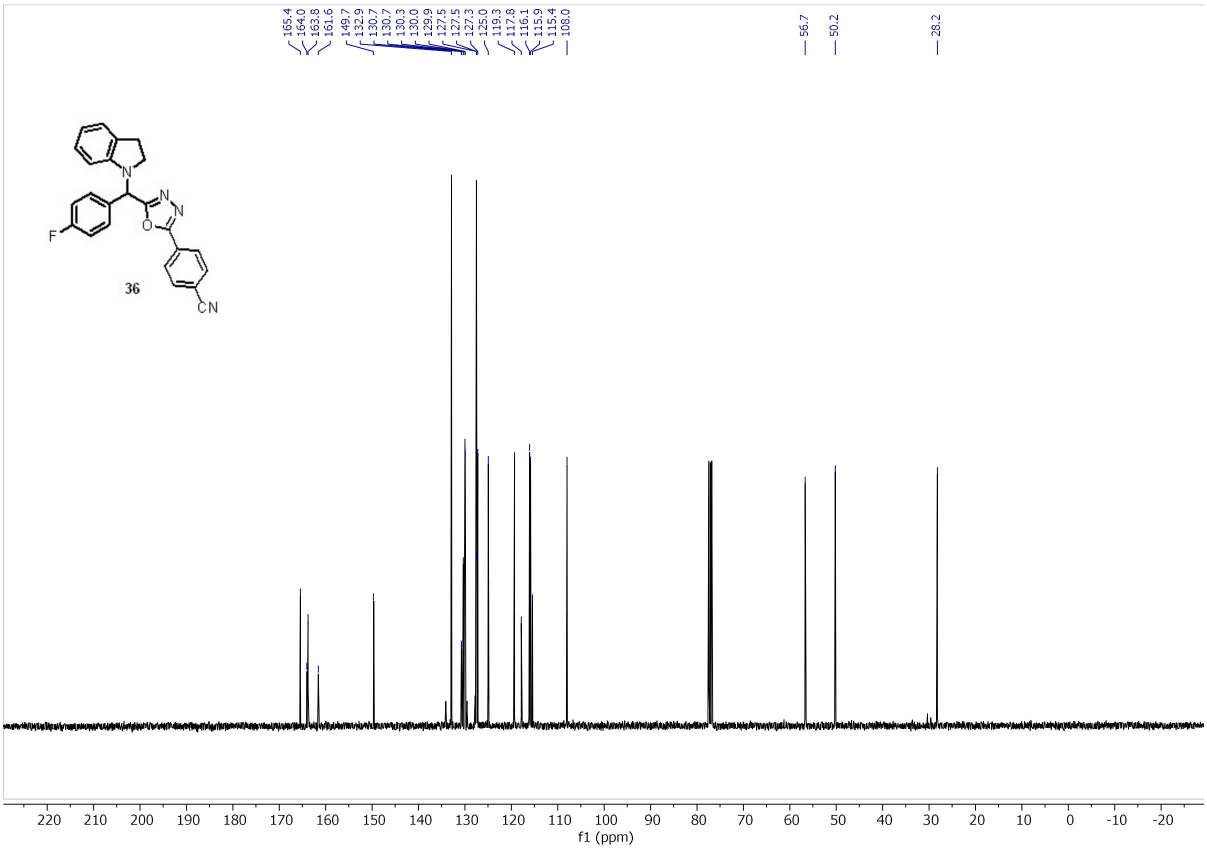

165.4
164.0
163.8
161.6
149.7
132.9
130.7
130.7
130.3
130.0
129.9
127.5
127.5
127.3
125.0
119.3
117.8
116.1
115.9
115.4
108.0
56.7
50.2
28.2
F
N
N
N
O
36
CN
f1 (ppm)

(4-chlorophenyl)(5-methoxy-2-methyl-3-((5-((methyl(phenyl)amino)methyl)-1,3,4-oxadiazol-2-yl)methyl)-1H-indol-1-yl)methanone - 37

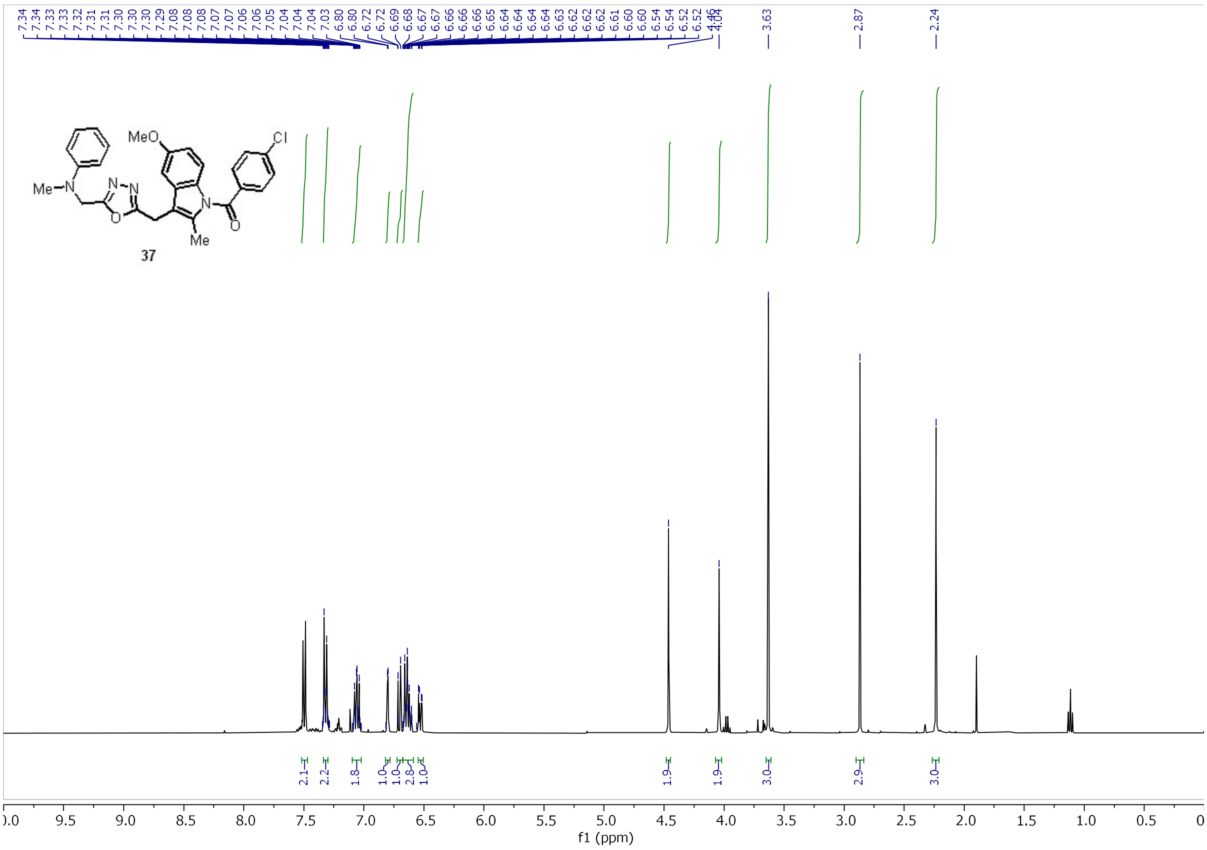

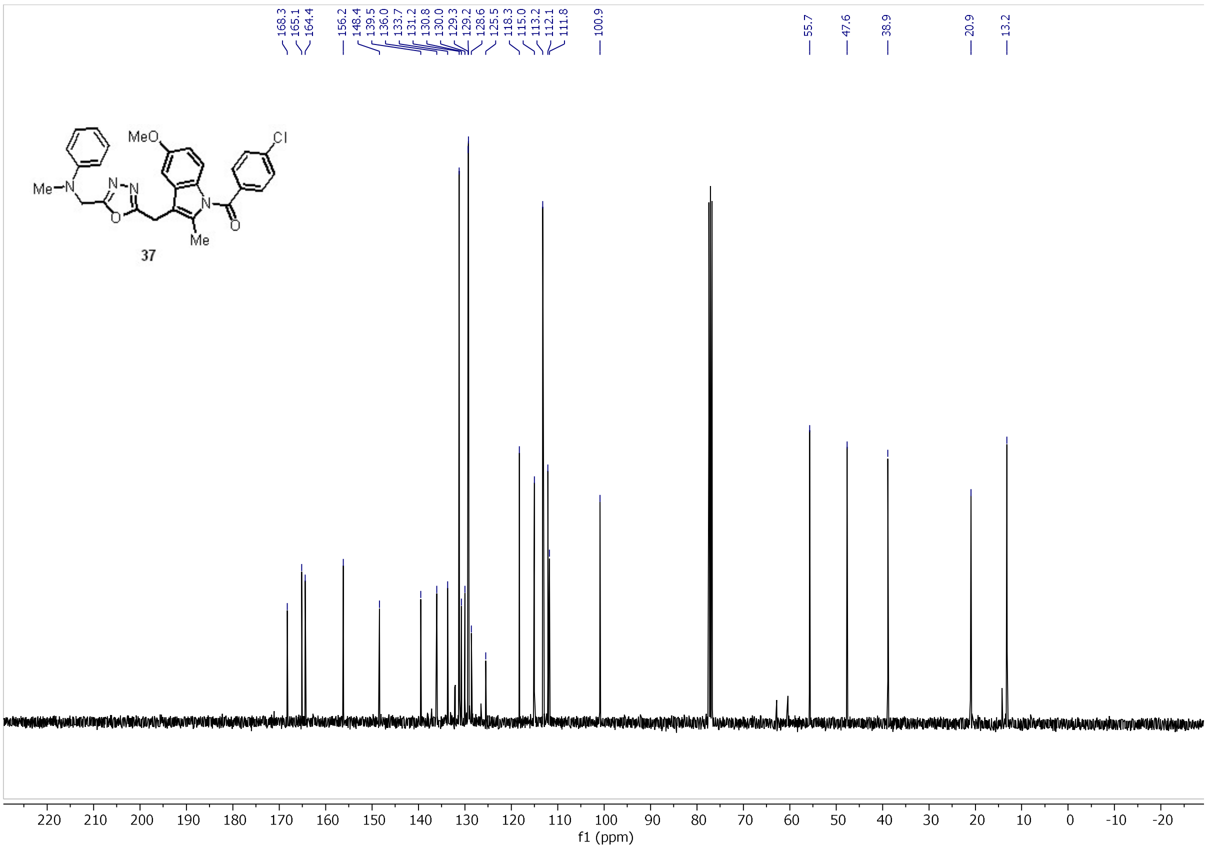

ethyl (E)-5-(((5-(4-bromophenyl)-1,3,4-oxadiazol-2-yl)(4-methoxyphenyl)methyl)(phenyl)amino)pent-2-enoate - 38

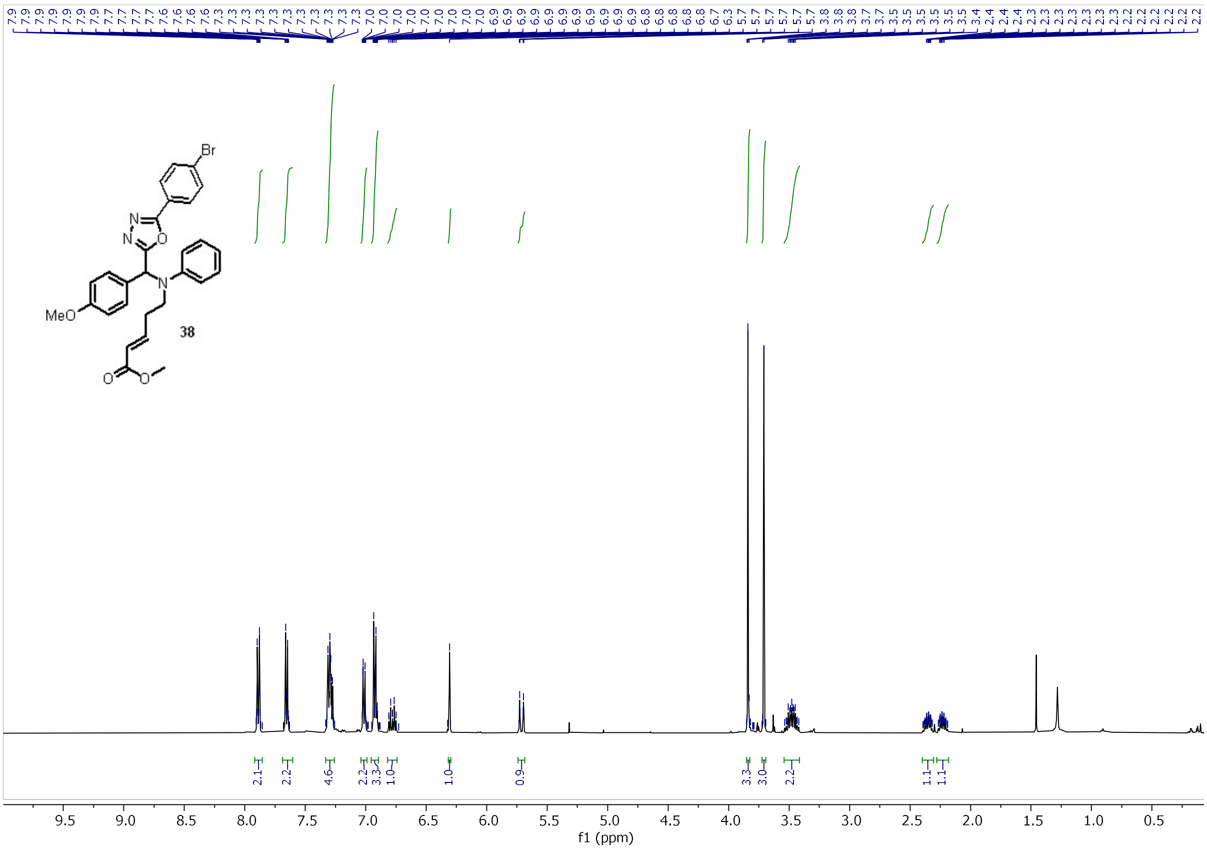

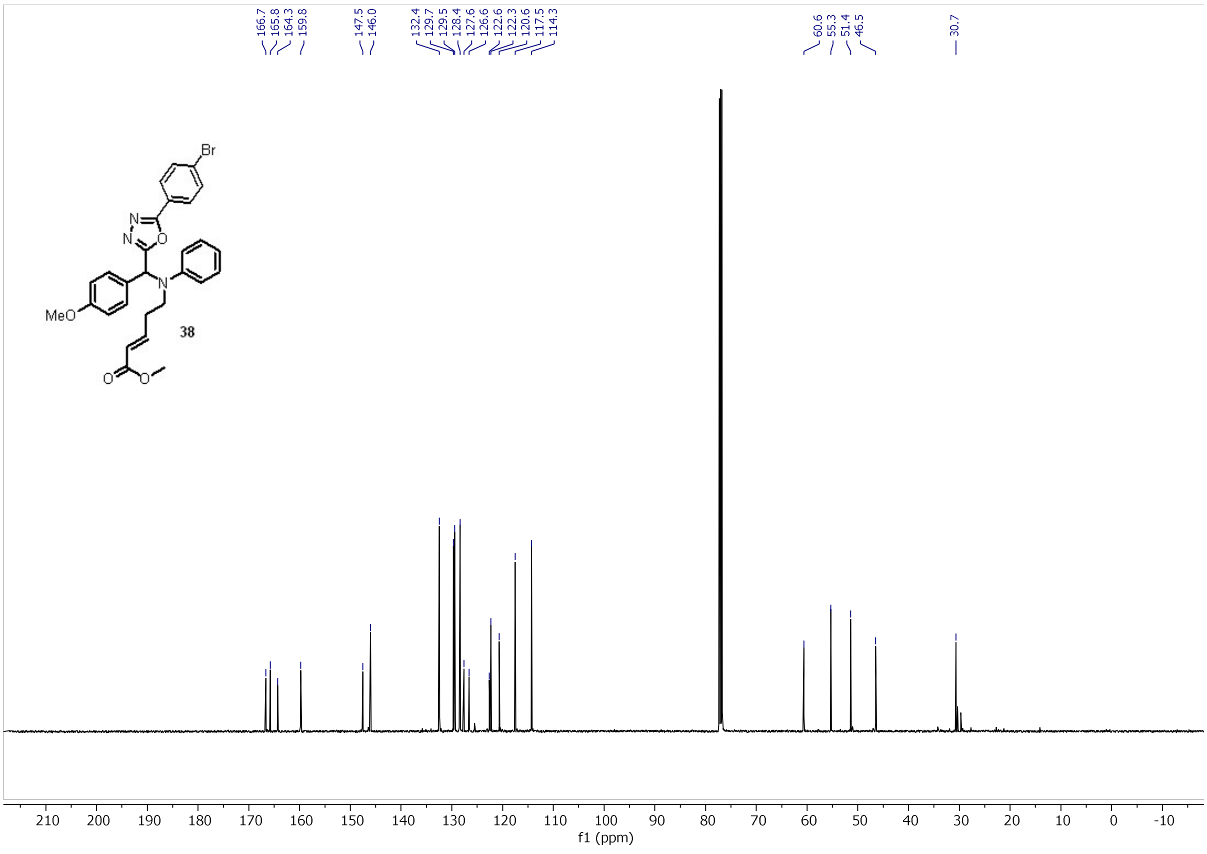

tert-butyl 4-((5-((((benzyloxy)carbonyl)amino)methyl)-1,3,4-oxadiazol-2-yl)(2-iodophenyl)methyl)piperazine-1-carboxylate - 39

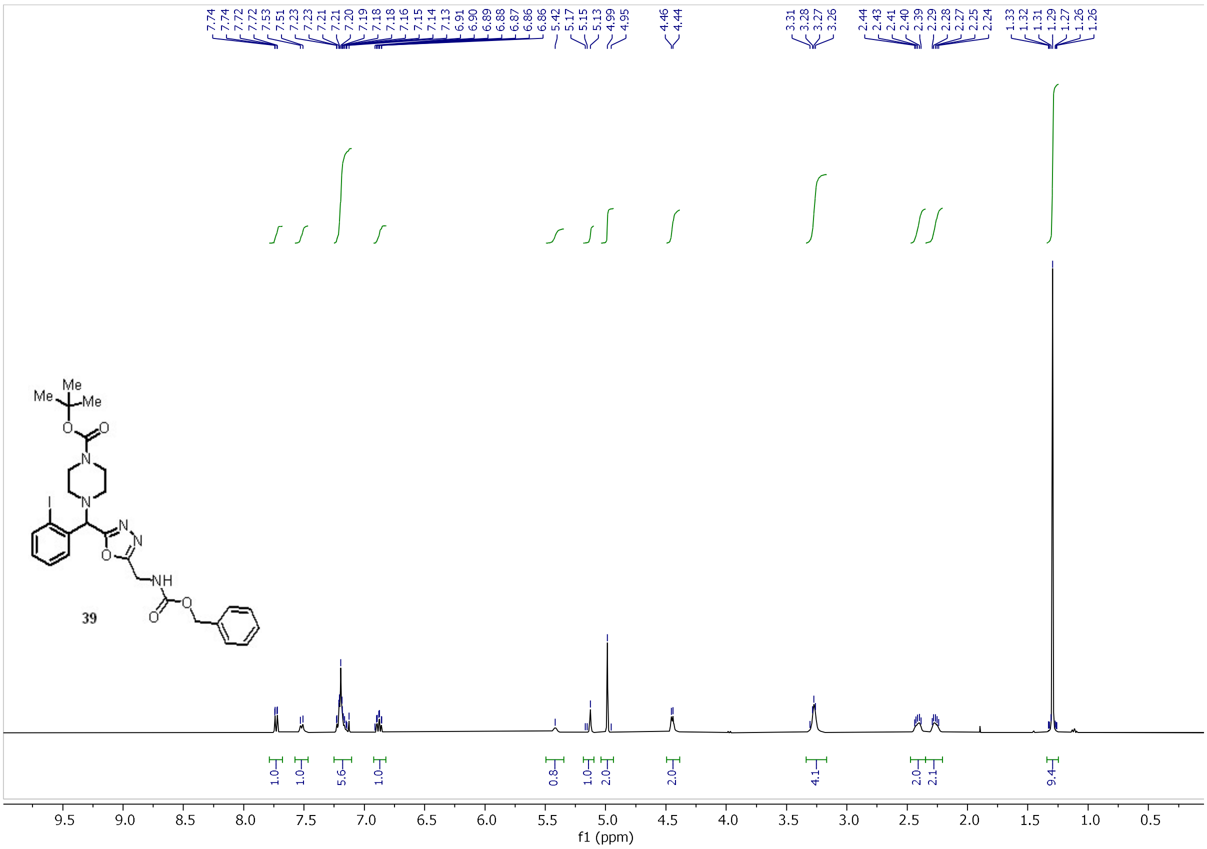

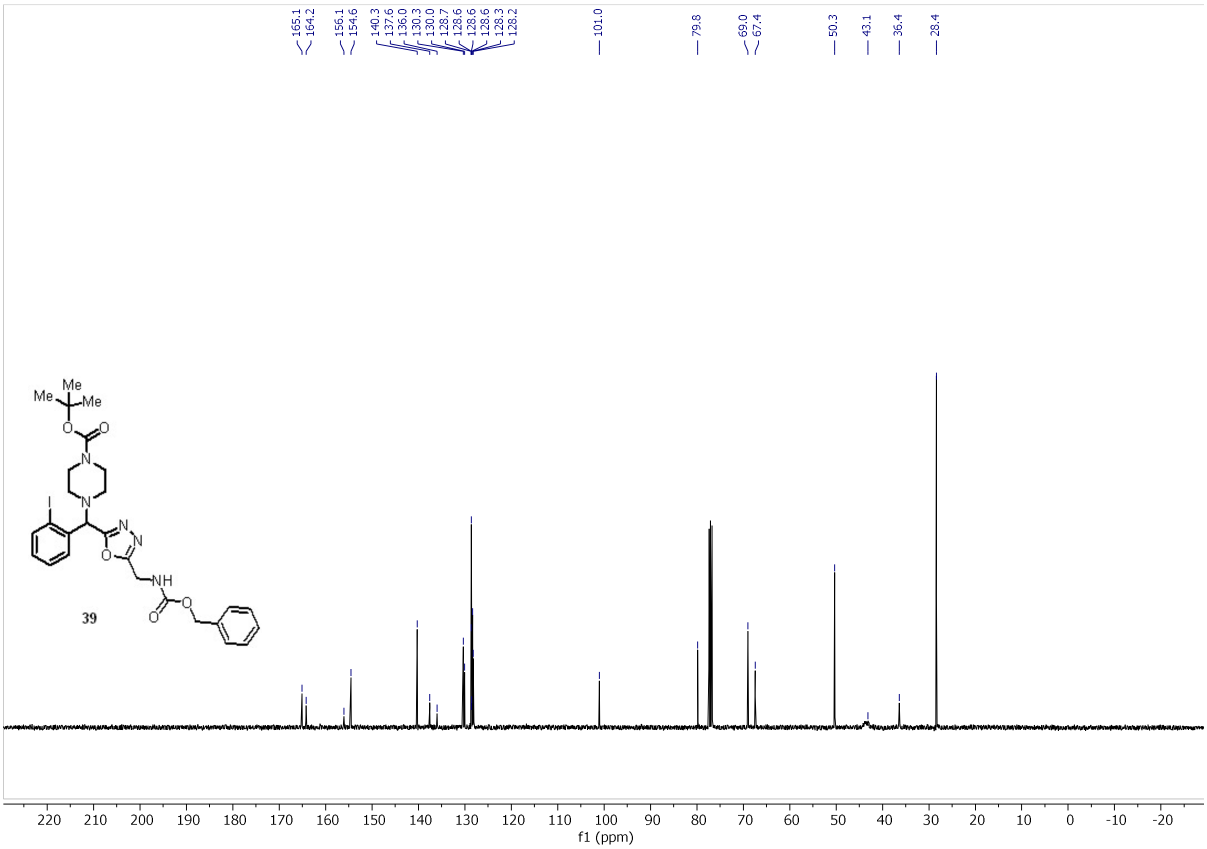

4-((5-((1H-indol-3-yl)methyl)-1,3,4-oxadiazol-2-yl)(4-nitrophenyl)methyl)morpholine - 40

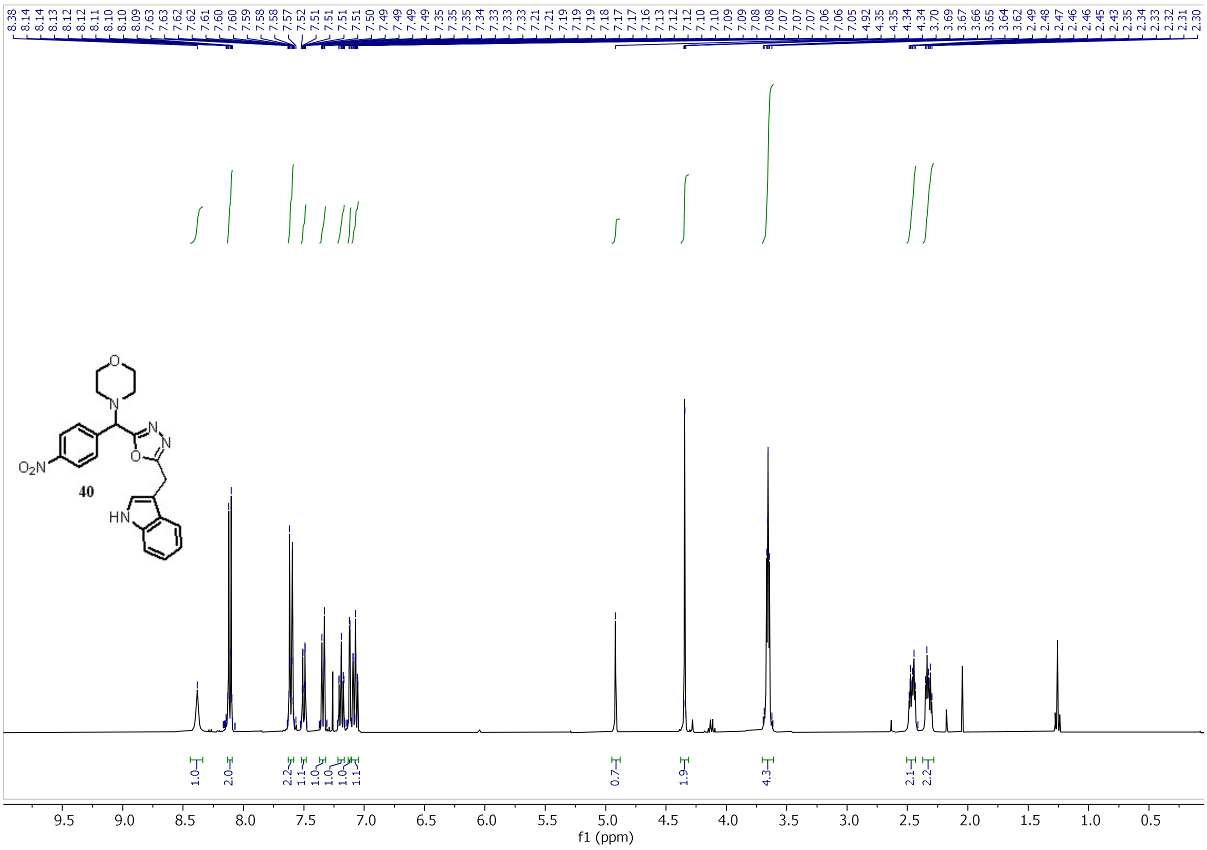

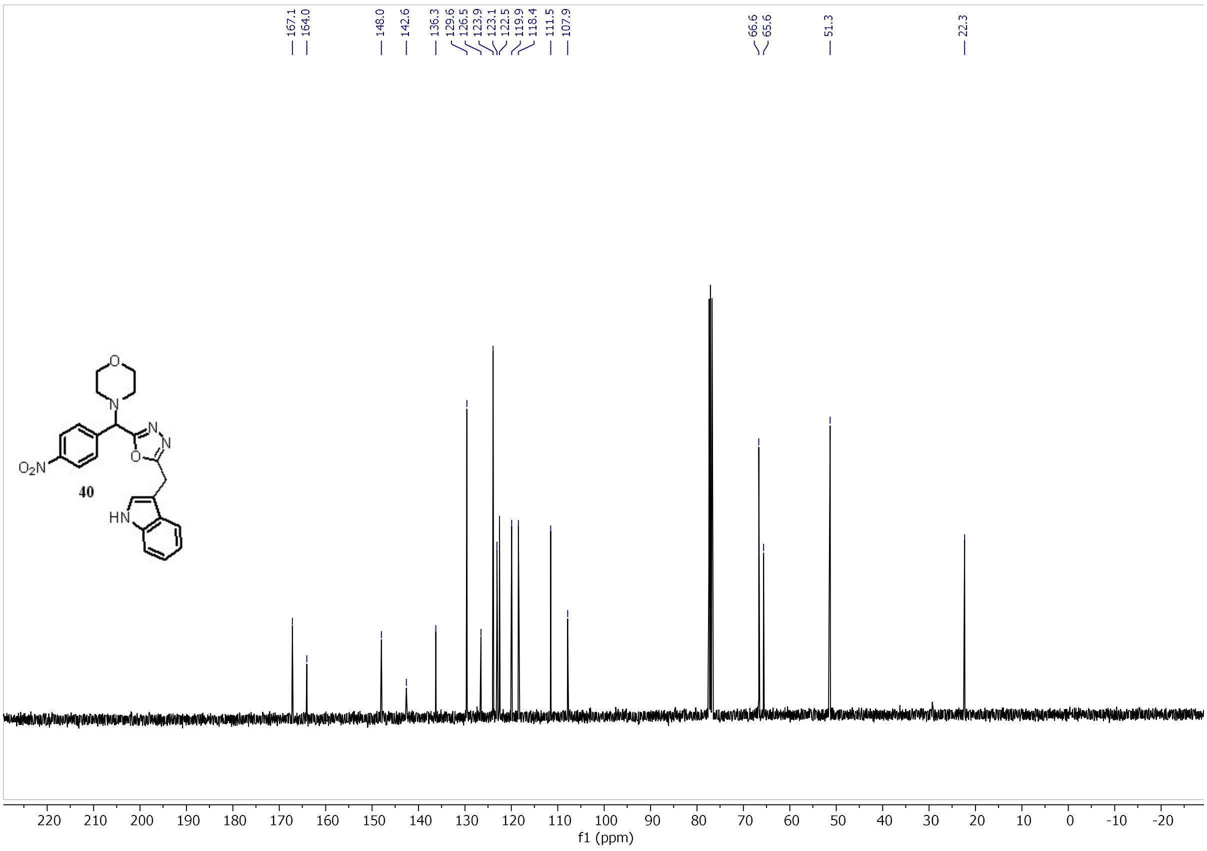

2-((4-methylpiperazin-1-yl)(4-(4,4,5,5-tetramethyl-1,3,2-dioxaborolan-2-yl)phenyl)methyl)-5-(phenanthren-9-yl)-1,3,4-oxadiazole - 41

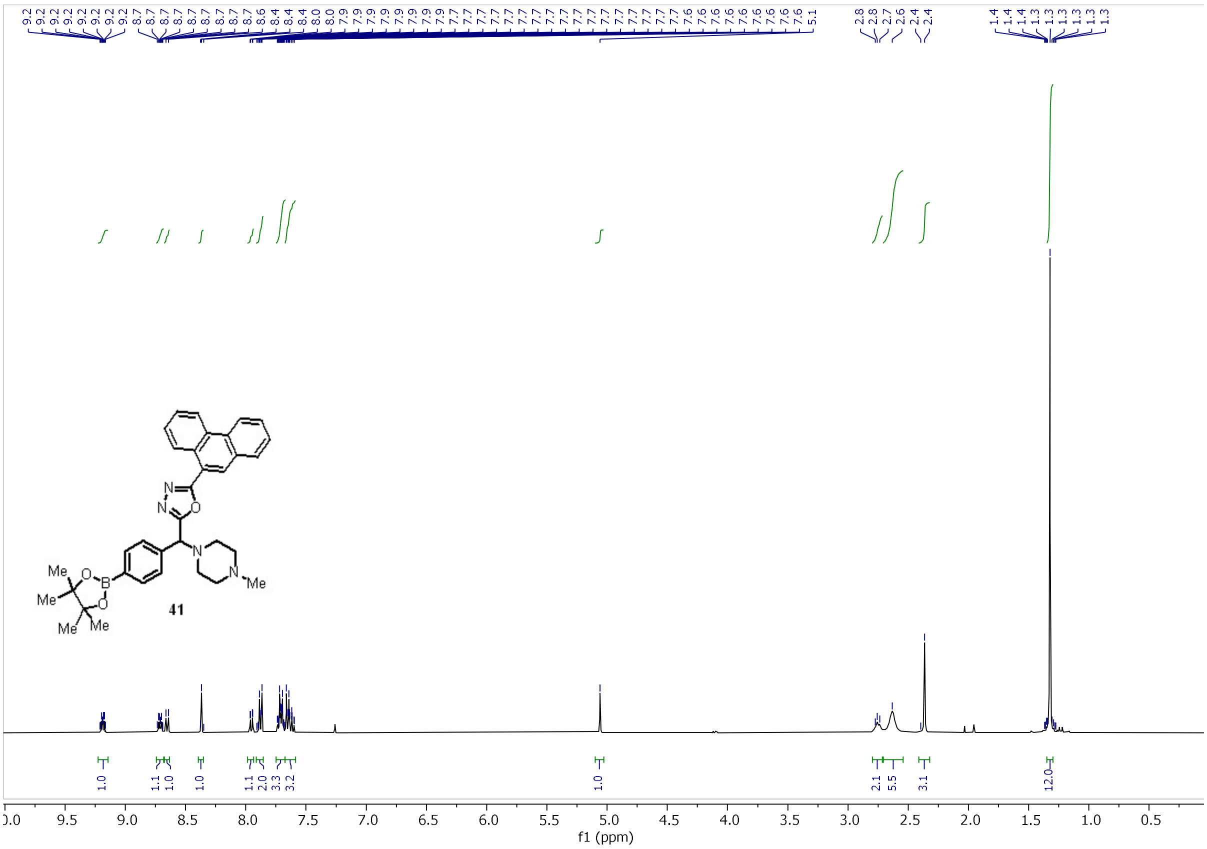

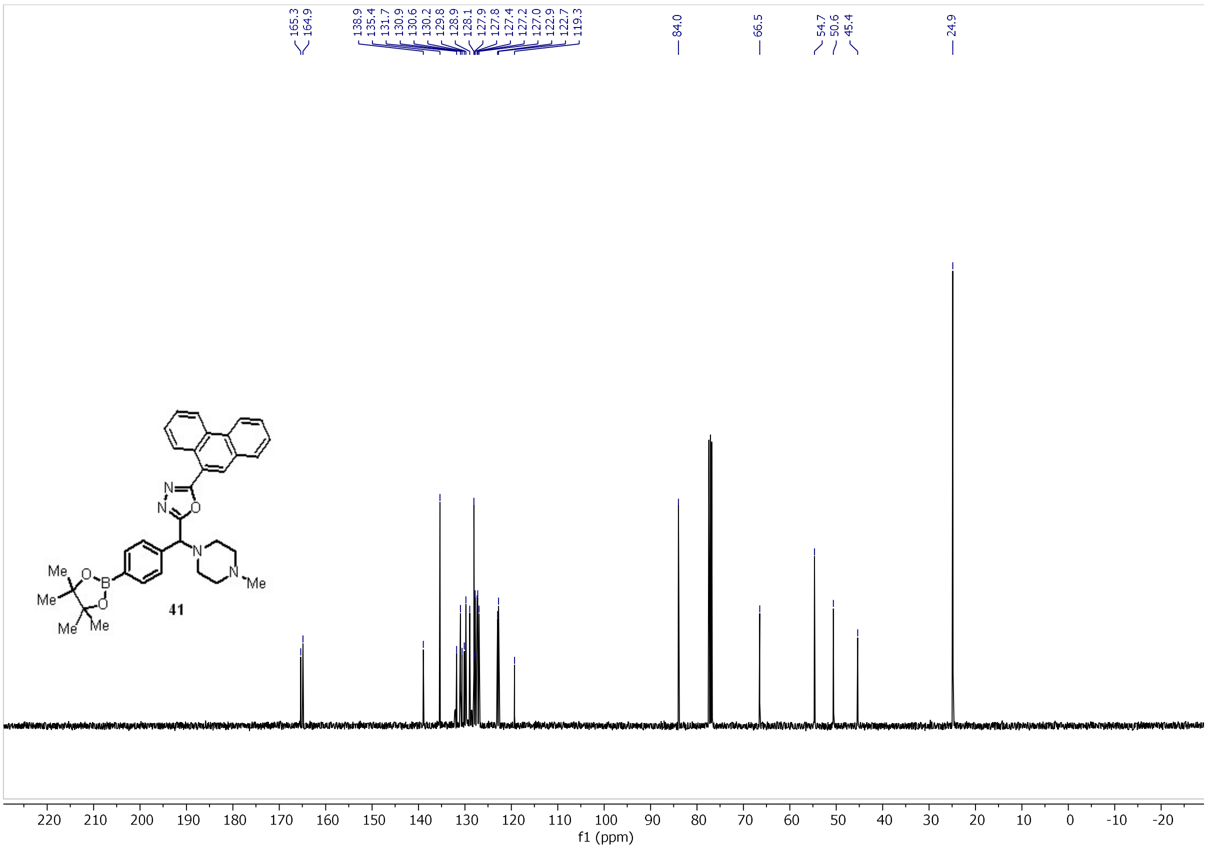

N,O-dimethyl-N-((5-(5-methyl-2-phenyloxazol-4-yl)-1,3,4-oxadiazol-2-yl)(phenyl)methyl)hydroxylamine - 42

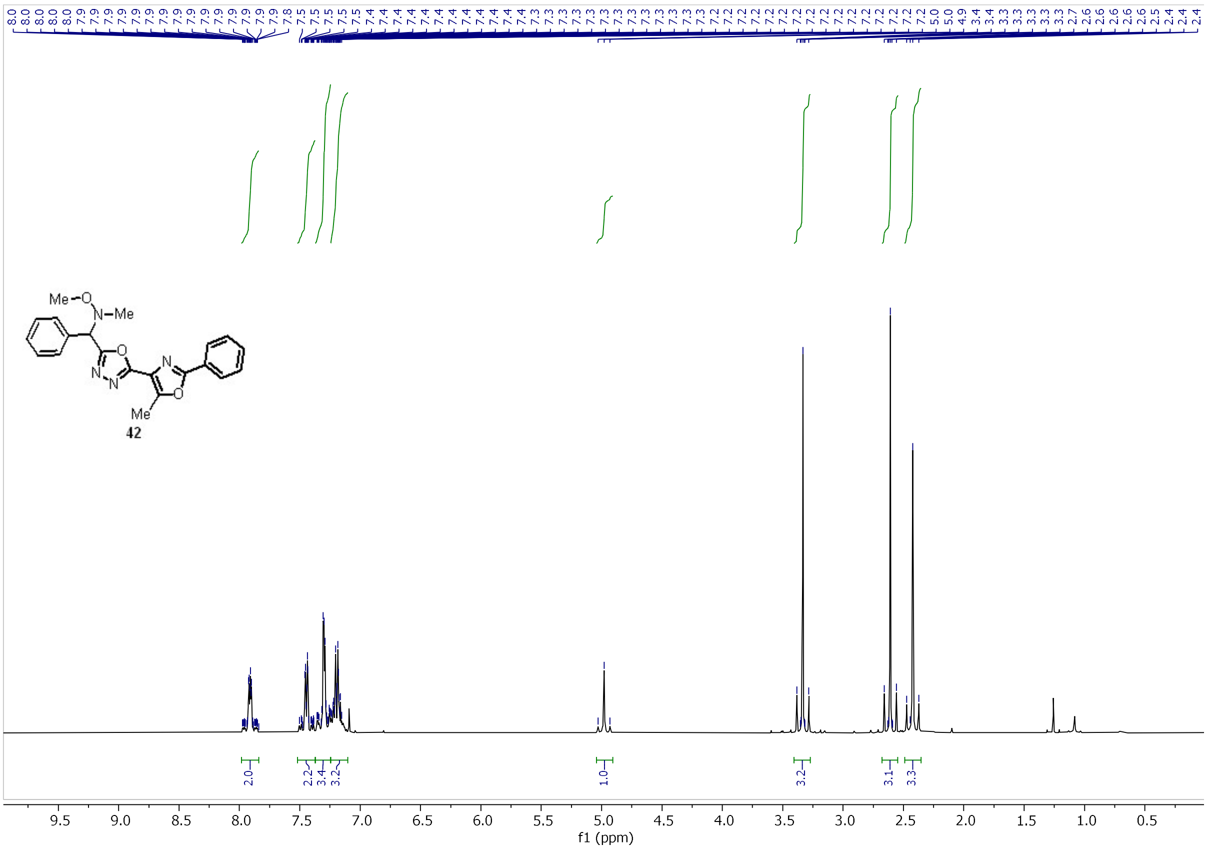

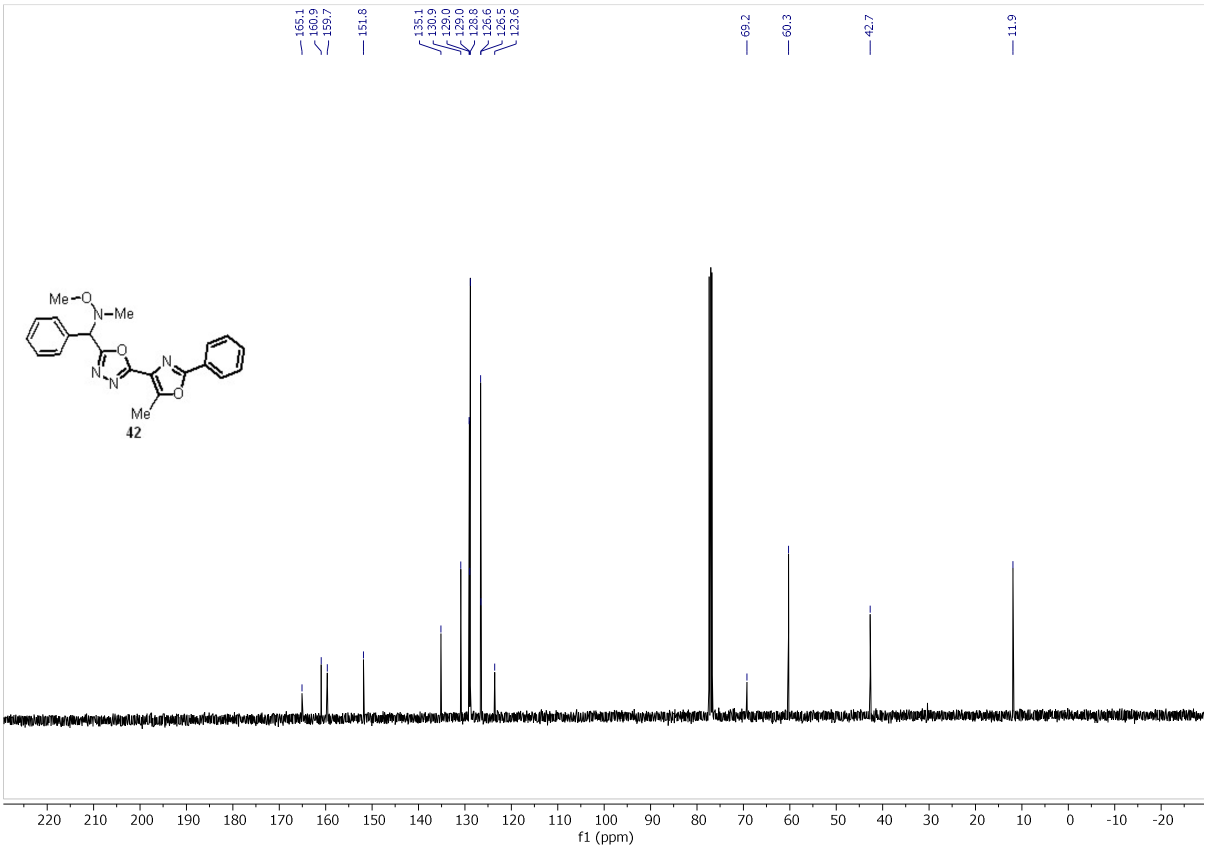

4-((dimethylamino)(5-(3-iodophenyl)-1,3,4-oxadiazol-2-yl)methyl)-9H-fluoren-9-one - 43

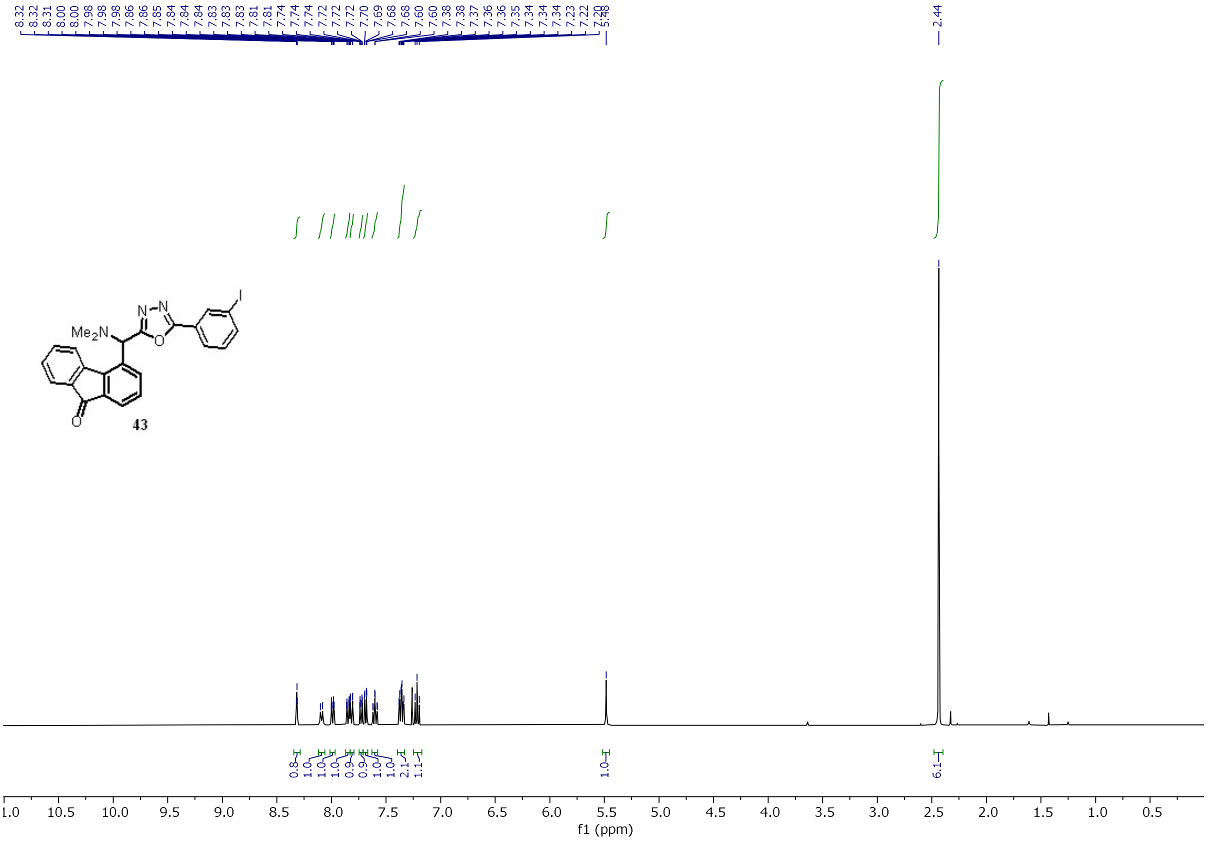

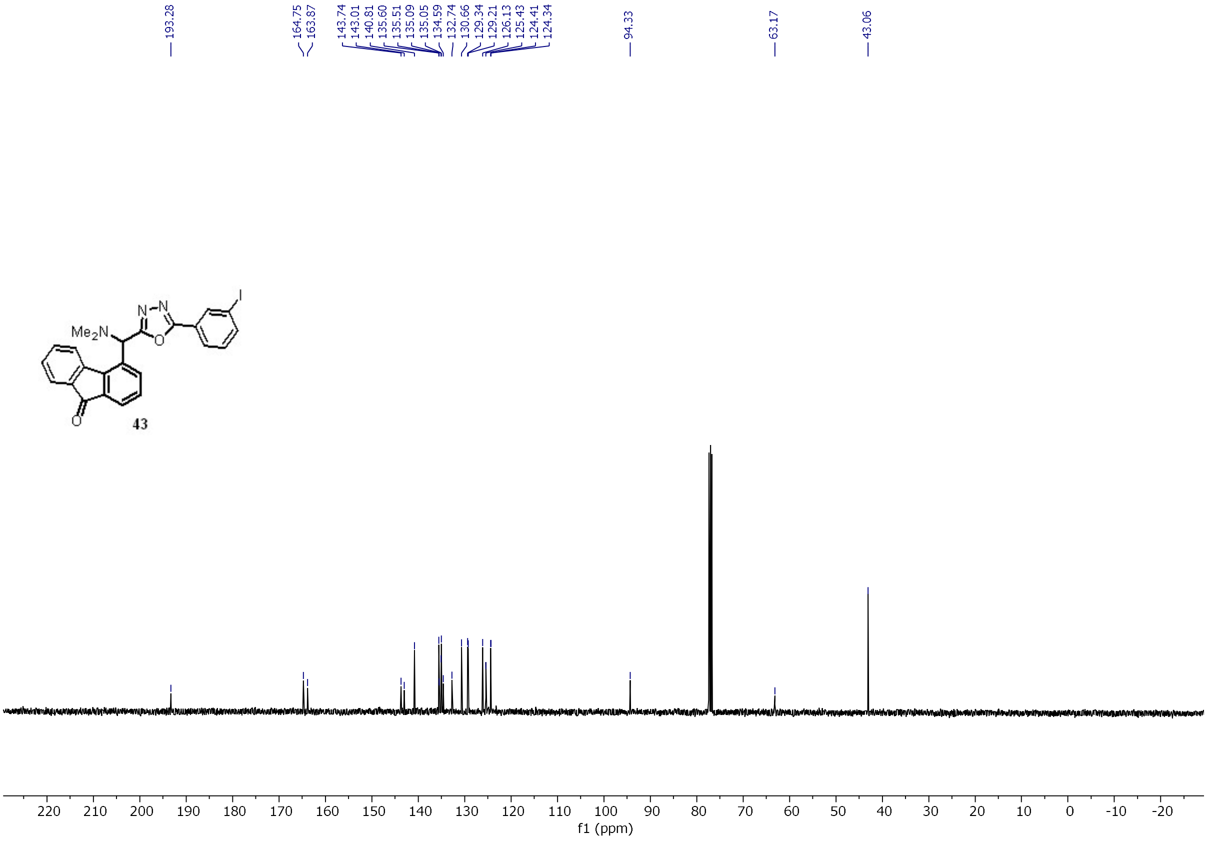

2-(5-(1-(dimethylamino)-3-phenylpropyl)-1,3,4-oxadiazol-2-yl)benzonitrile - 44

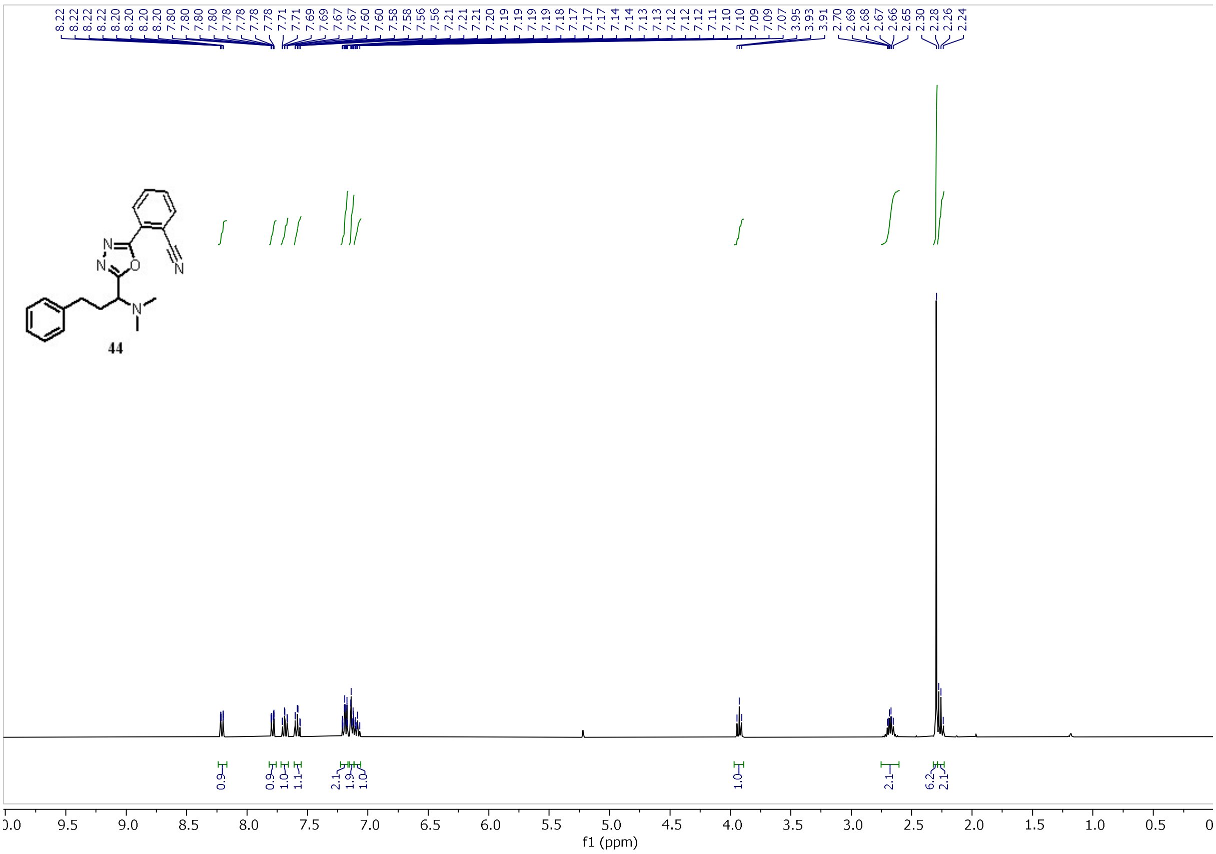

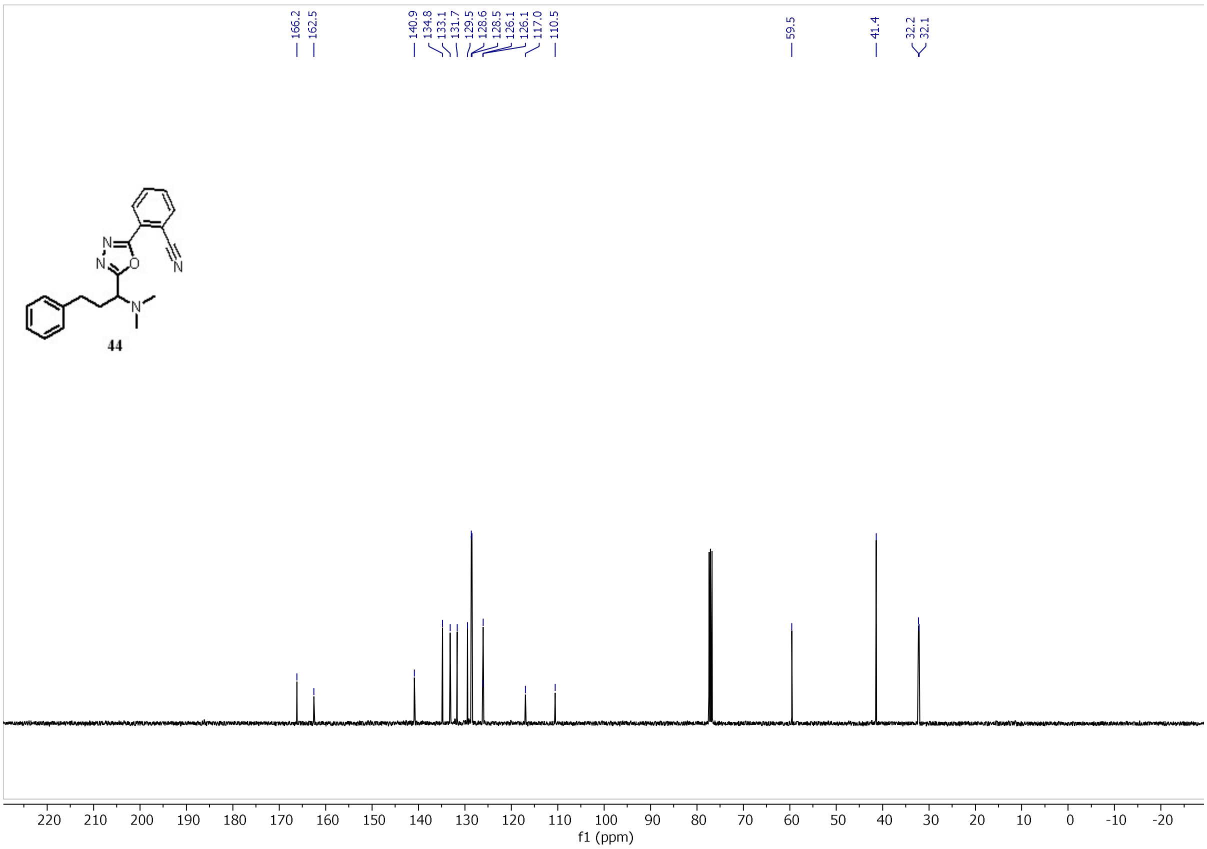

tert-butyl (R)-(1-(5-((dimethylamino)methyl)-1,3,4-oxadiazol-2-yl)-2-(naphthalen-2-yl)ethyl)carbamate - 45

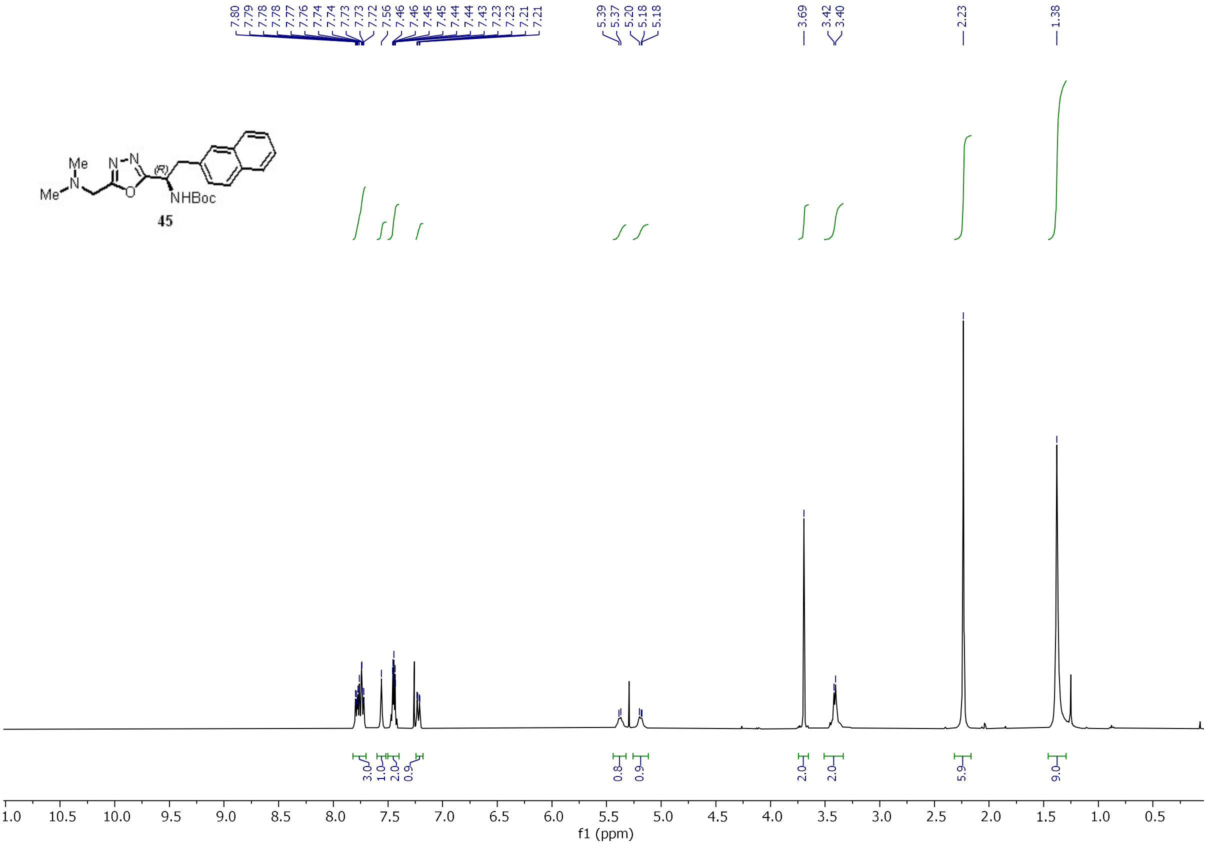

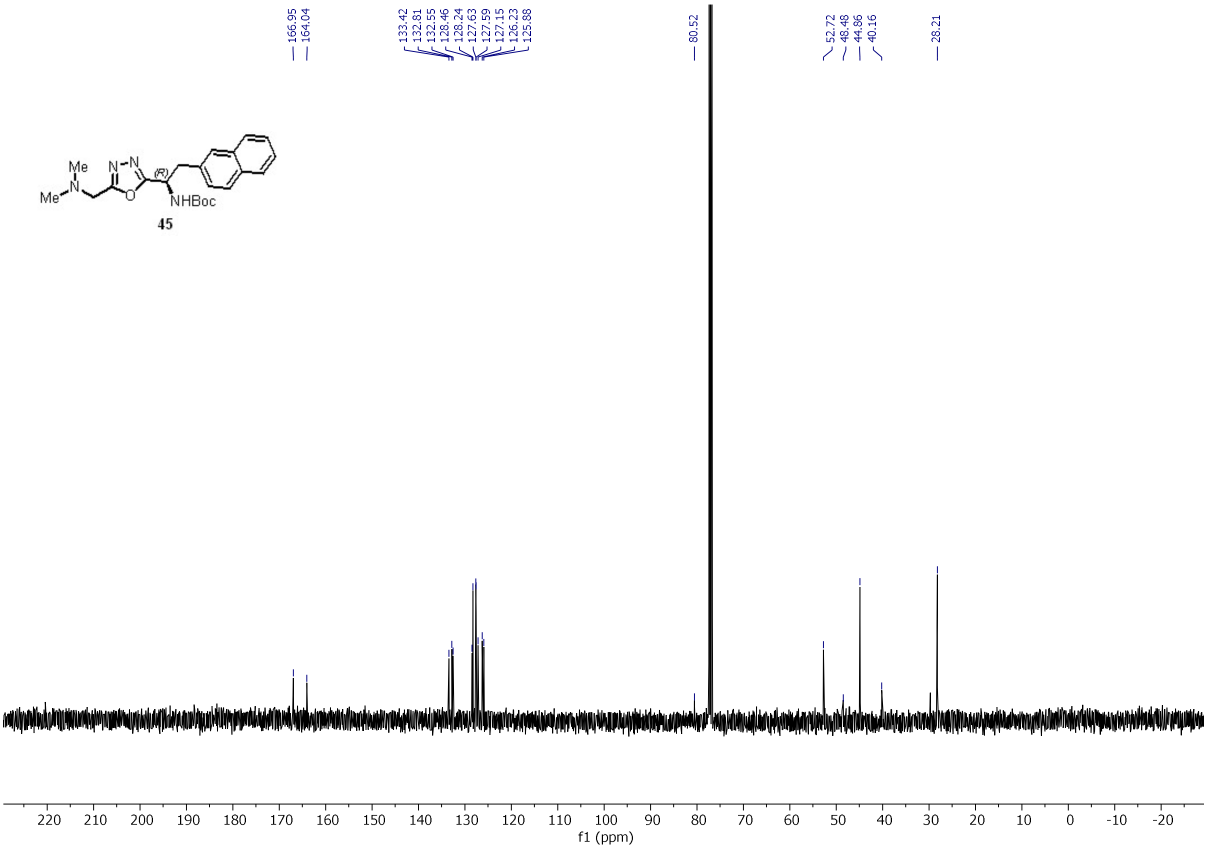

1-(4-(5-(pyrrolidin-1-ylmethyl)-1,3,4-oxadiazol-2-yl)piperidin-1-yl)ethan-1-one - 46

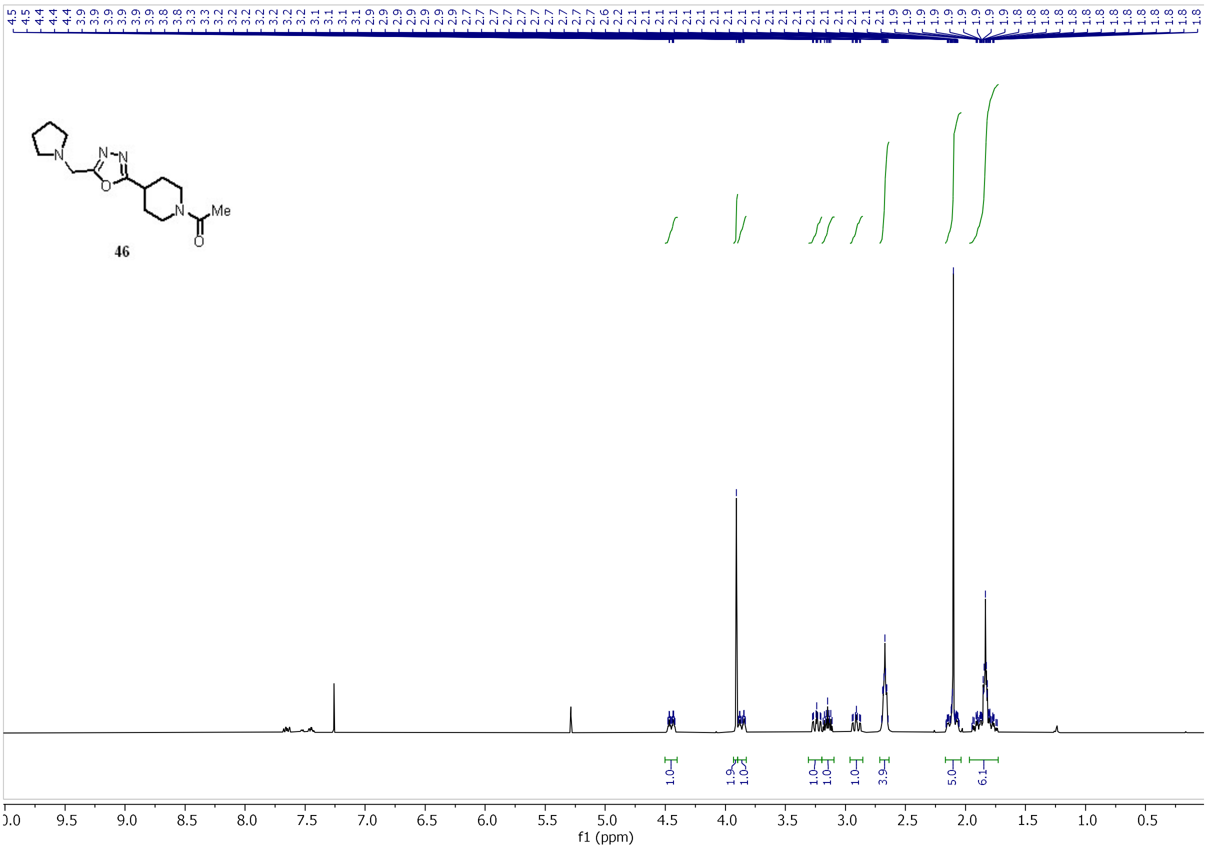

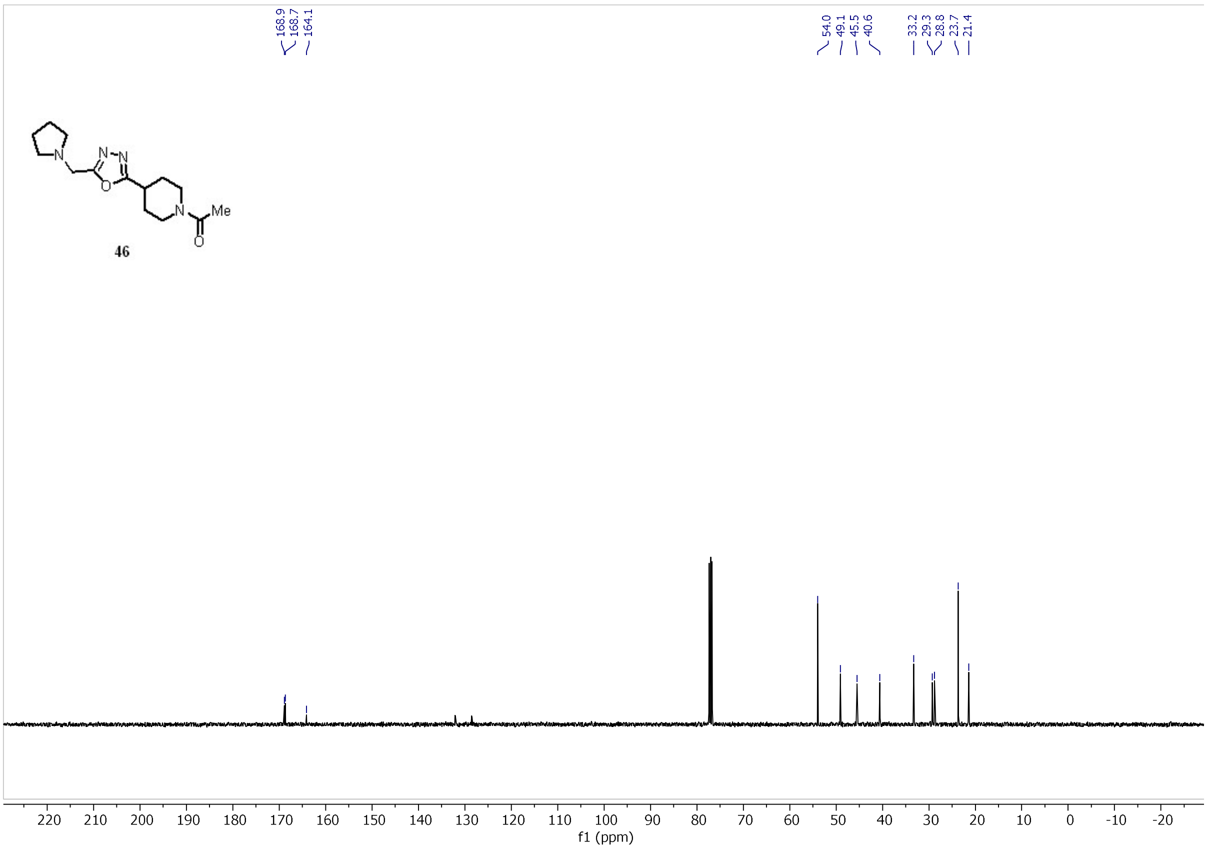

1,3,5-tris(5-(piperidin-1-ylmethyl)-1,3,4-oxadiazol-2-yl)benzene - 47

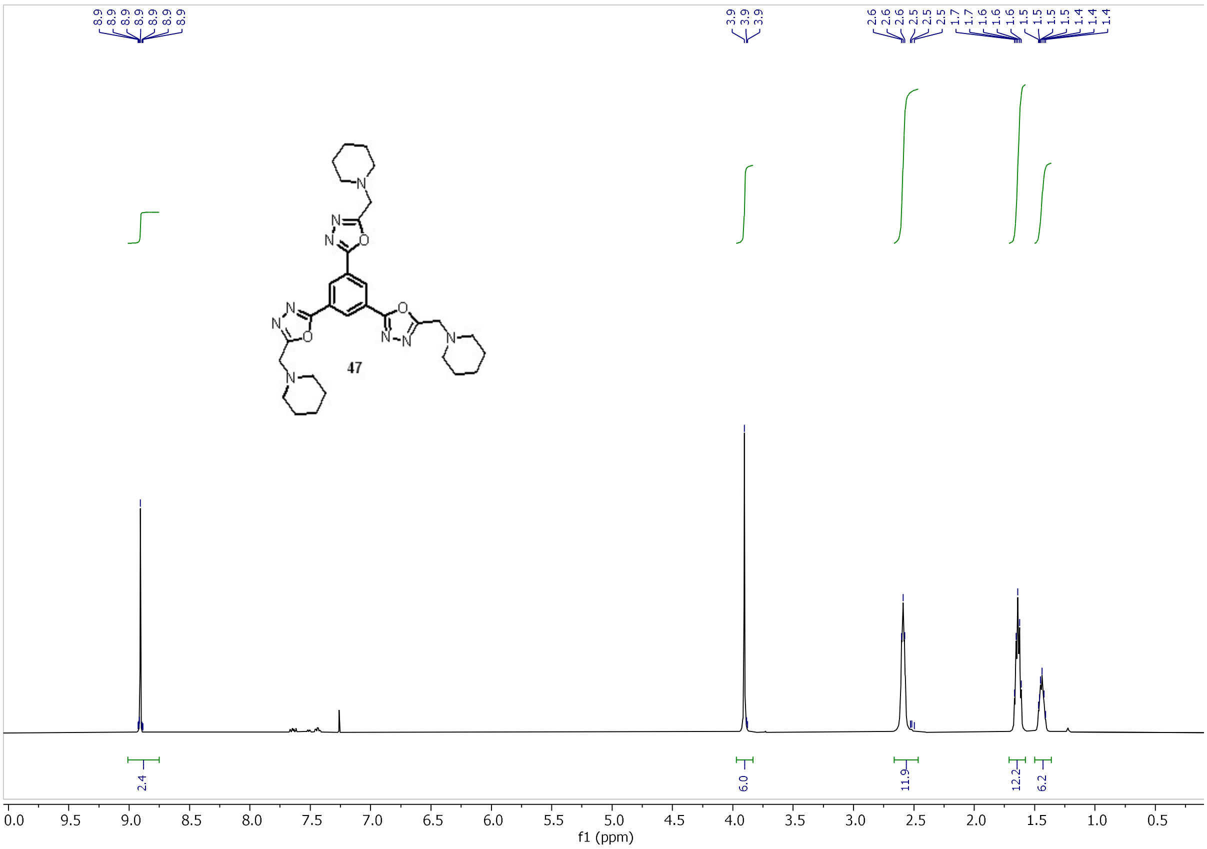

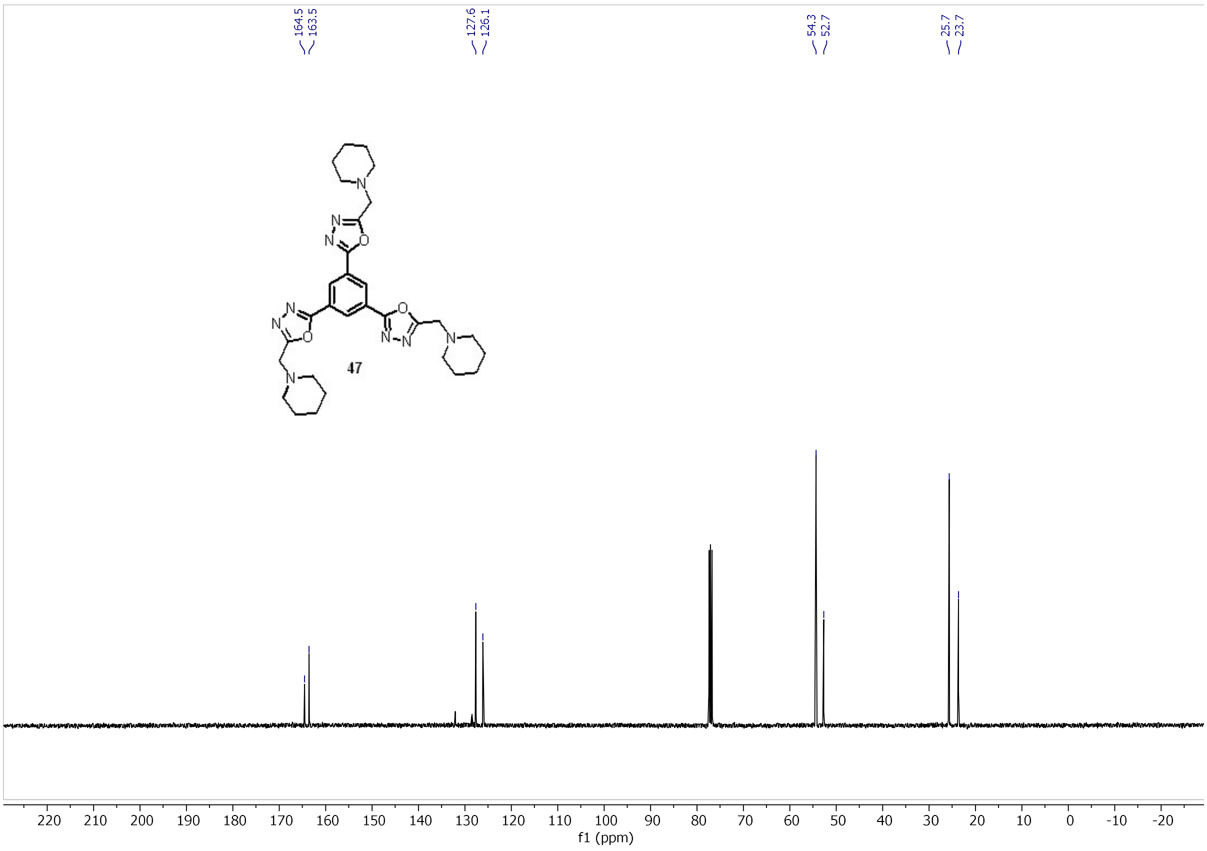

2-(2-chloro-5,11-dimethyl-6,11-dihydro-5H-benzo[e]pyrimido[5,4-b][1,4]diazepin-6-yl)-5-((3,5-dichloropyridin-4-yl)methyl)-1,3,4-oxadiazole - 48

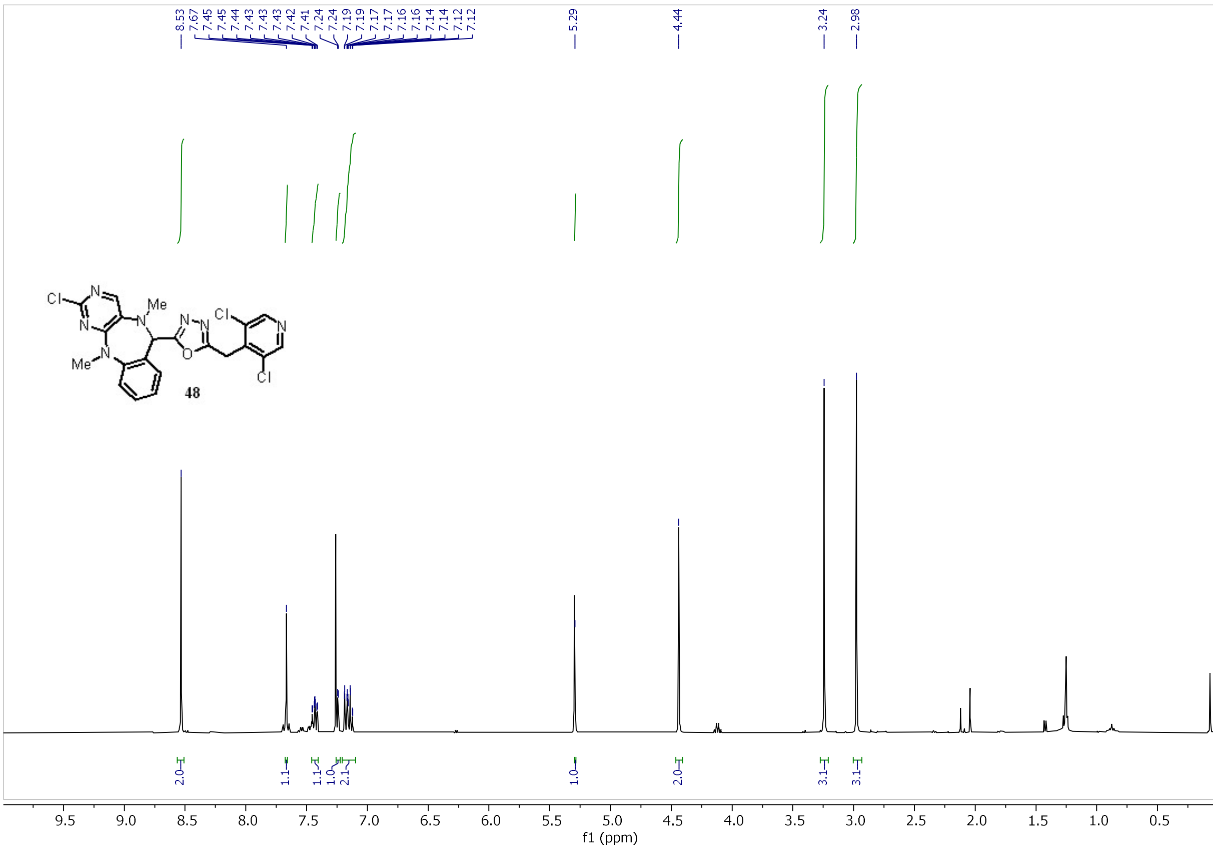

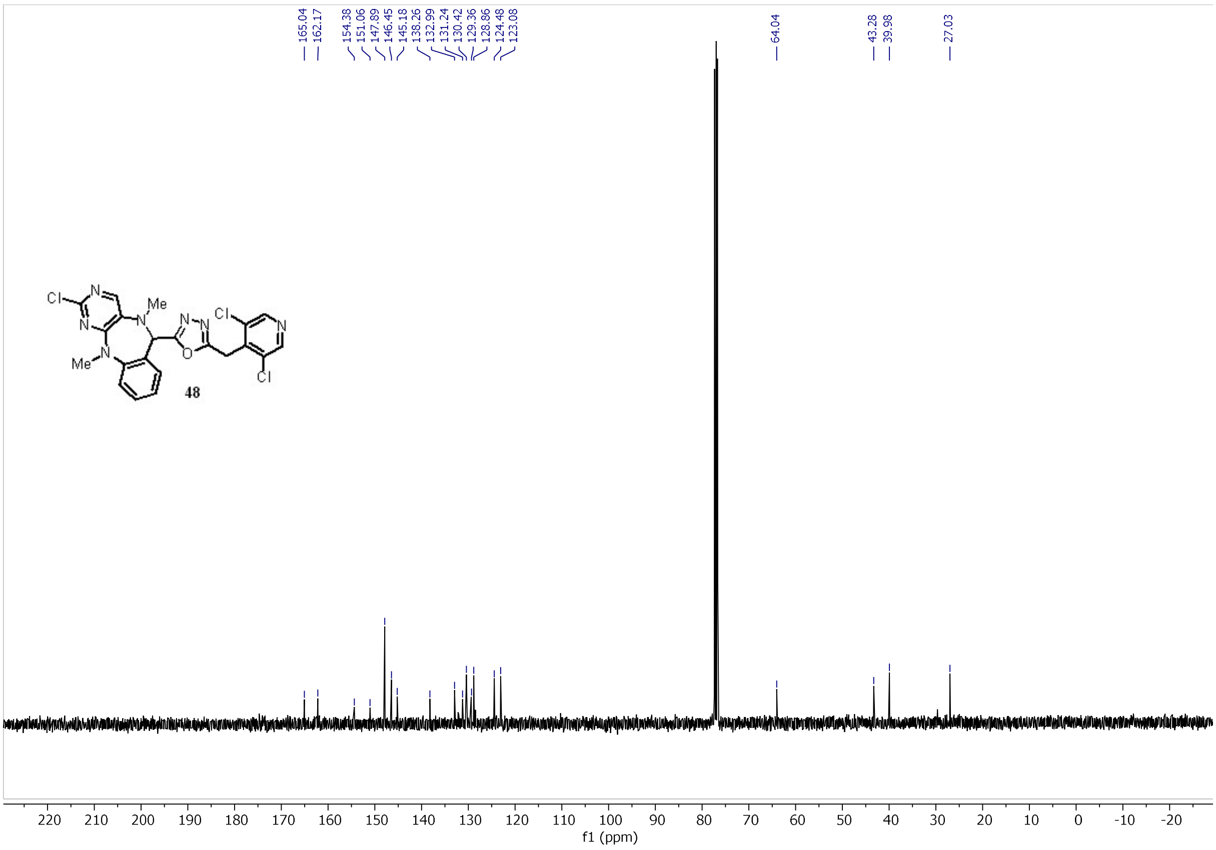

(Z)-3-((5-(1-(diethylamino)-2-(naphthalen-1-yloxy)propyl)-1,3,4-oxadiazol-2-yl)methylene)indolin-2-one - 49

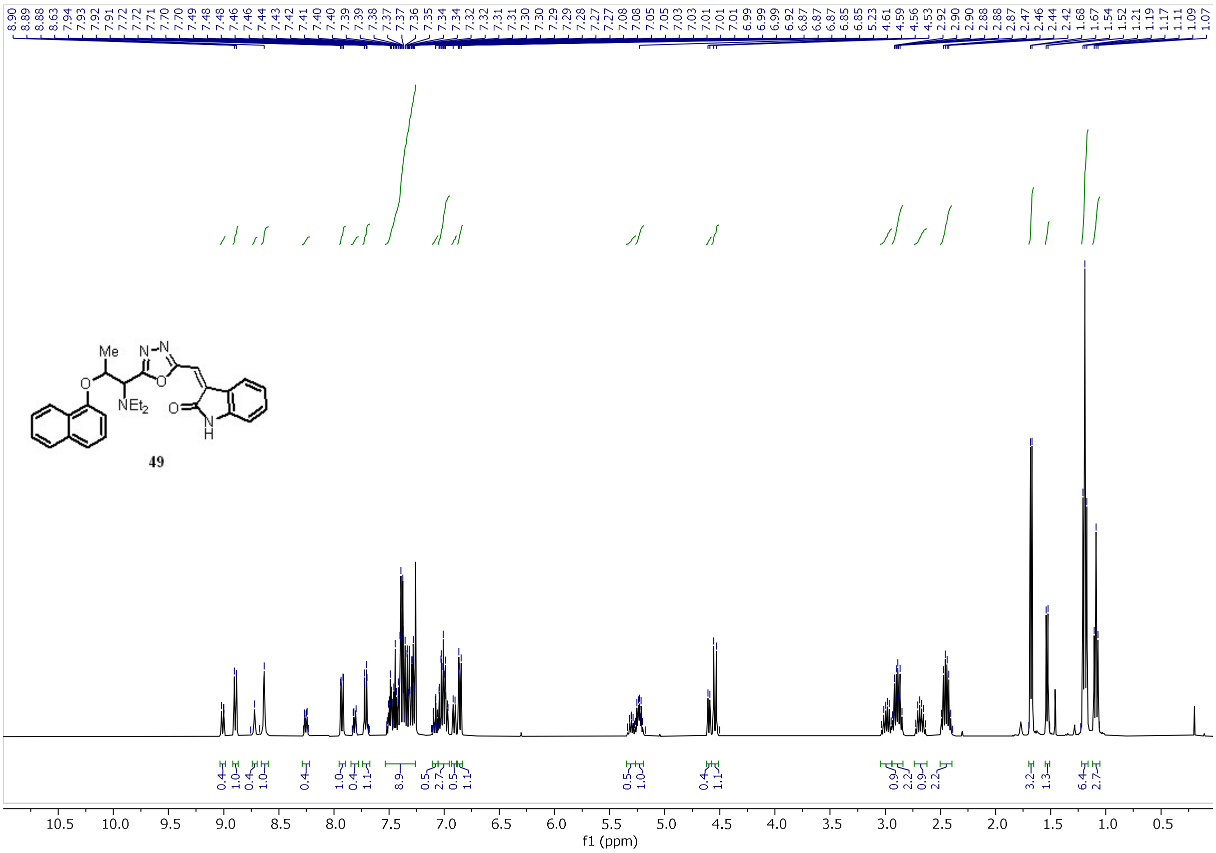

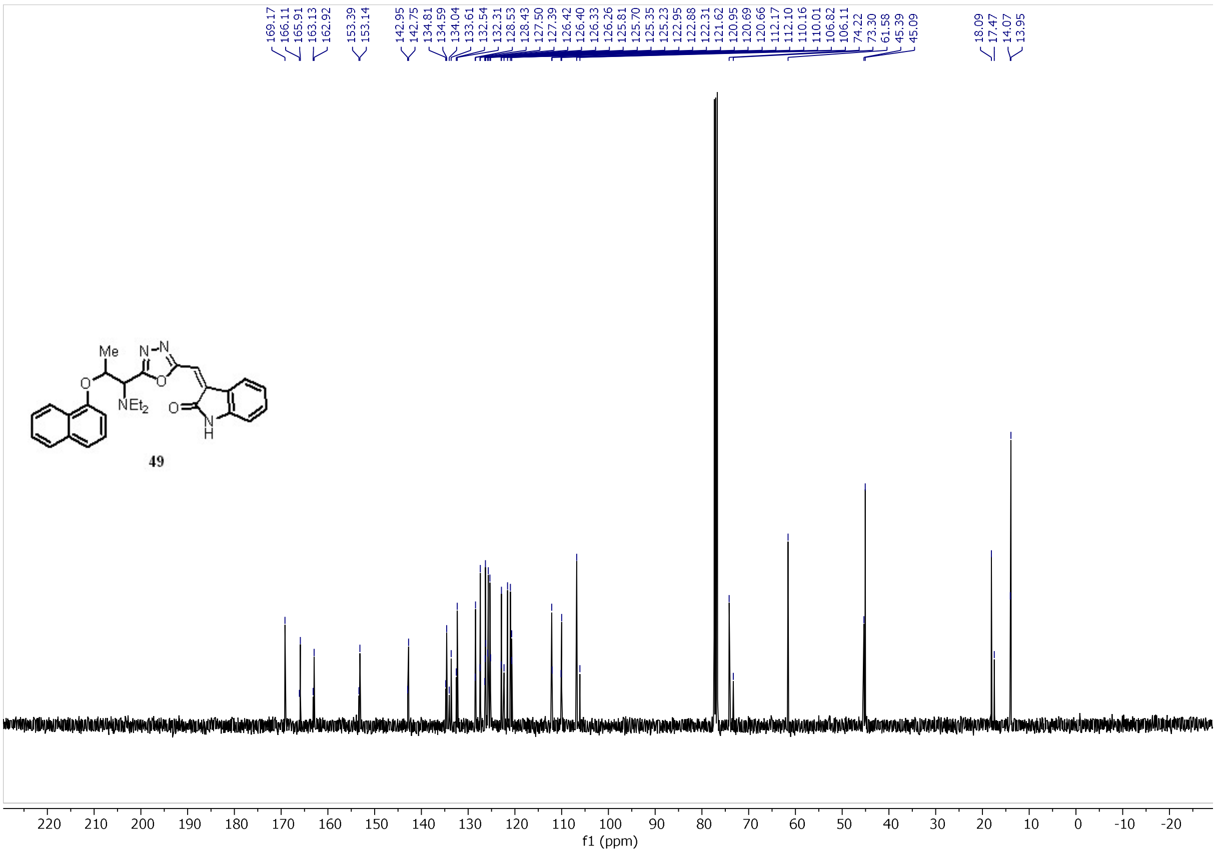

2-((2,3-dihydrobenzo[b][1,4]dioxin-6-yl)(piperidin-1-yl)methyl)-5-(3-fluoro-4-(trifluoromethyl)phenyl)-1,3,4-oxadiazole – 50

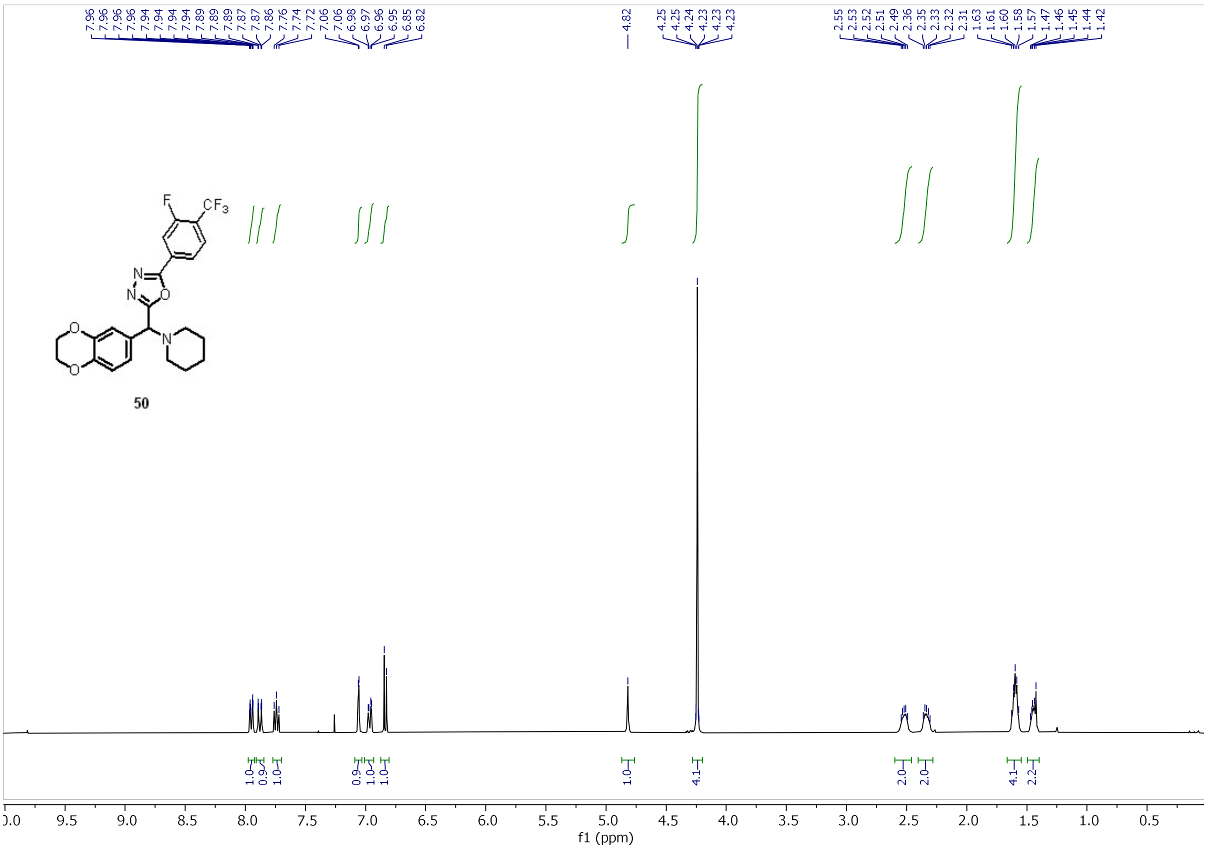

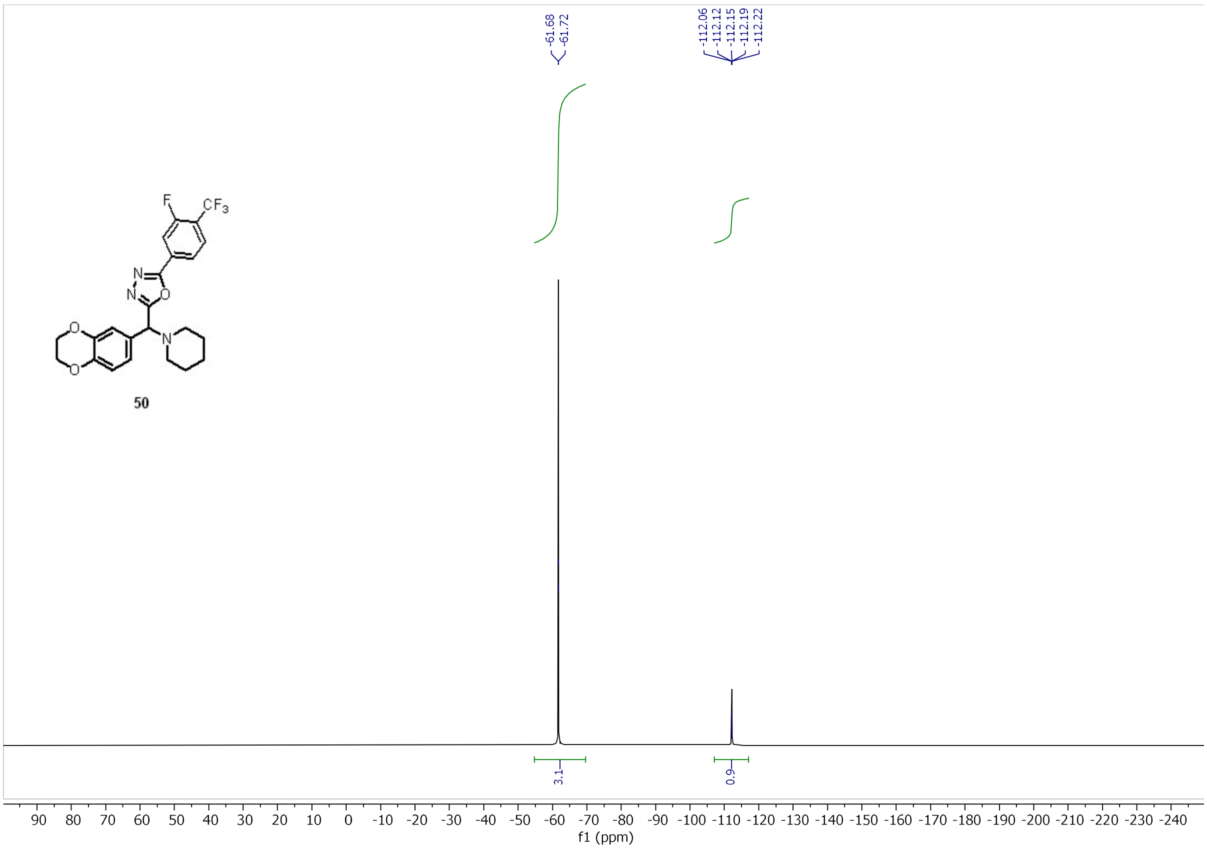

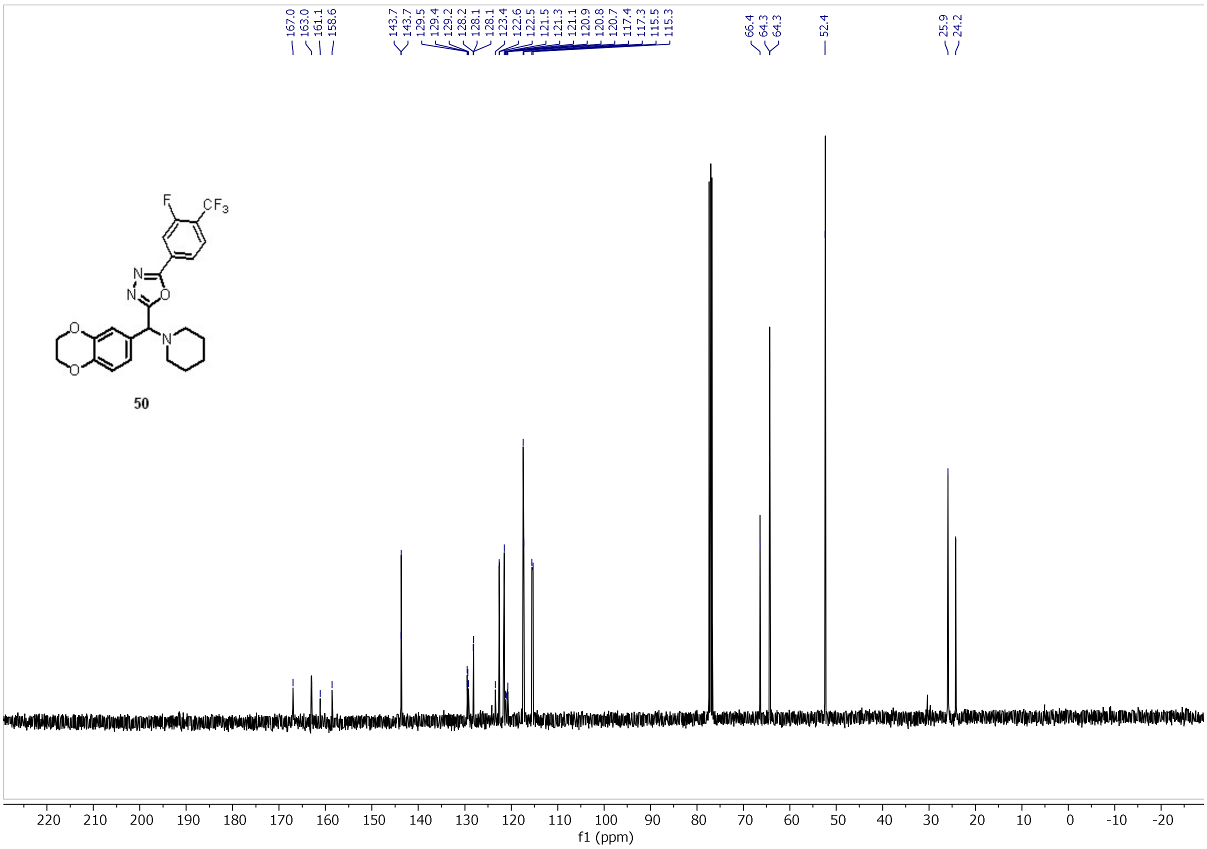

N-(3-(10,11-dihydro-5H-dibenzo[a,d][7]annulen-5-ylidene)propyl)-1-(5-(2-fluorophenyl)-1,3,4-oxadiazol-2-yl)-N-methyl-3-phenylpropan-1-amine - **51**

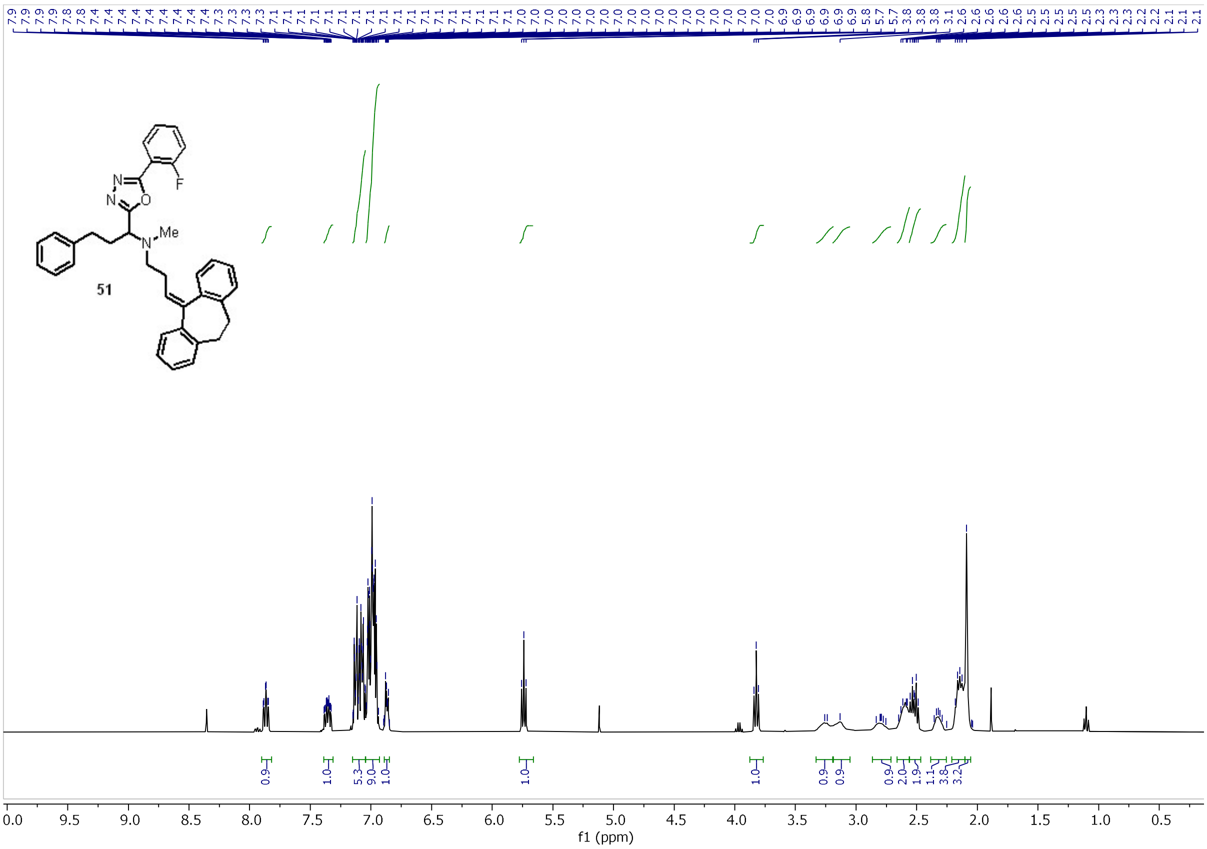

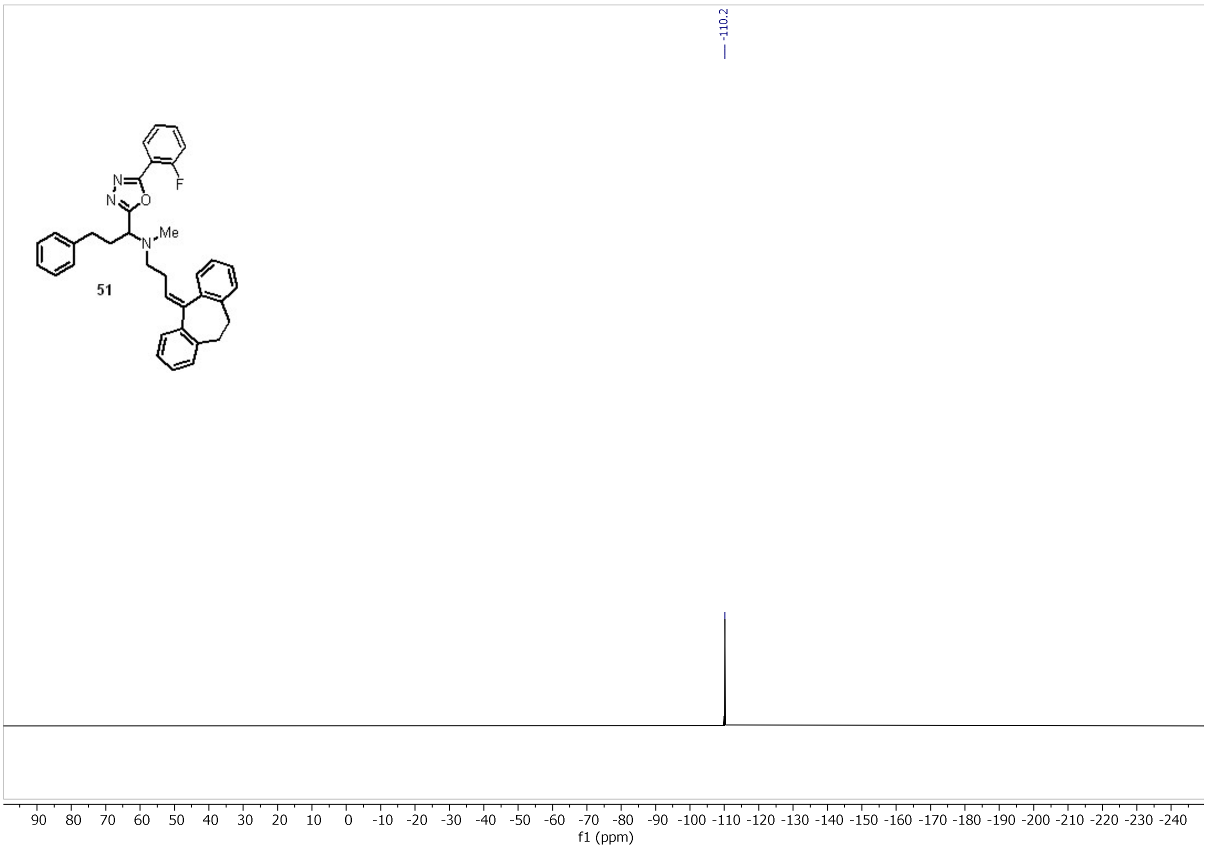

-110.2
N
N
O
F
Me
51
90 80 70 60 50 40 30 20 10 0 -10 -20 -30 -40 -50 -60 -70 -80 -90 -100 -110 -120 -130 -140 -150 -160 -170 -180 -190 -200 -210 -220 -230 -240
f1 (ppm)

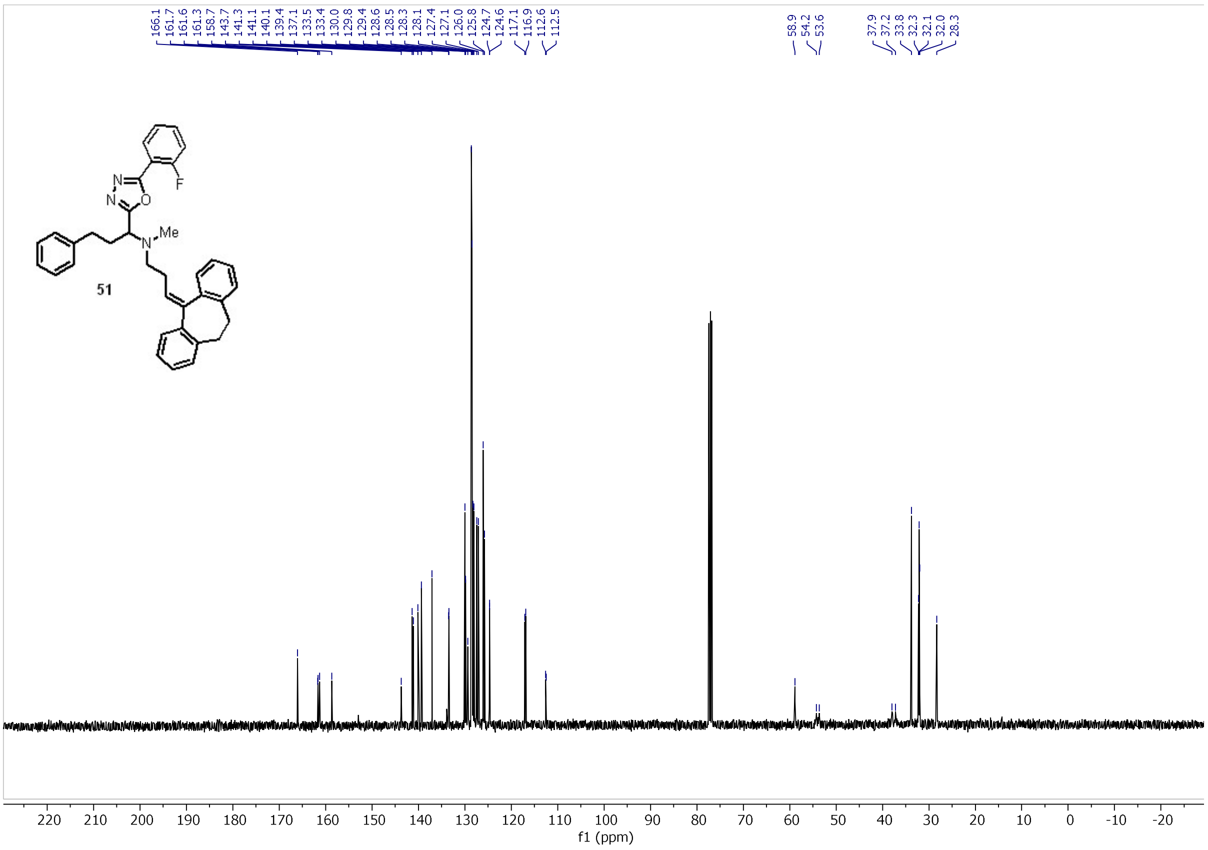

166.1 161.7 161.6 161.3 158.7 143.7 141.3 141.1 140.1 139.4 137.1 133.5 133.4 130.0 129.8 129.4 128.6 128.5 128.3 128.1 127.4 127.1 126.0 125.8 124.7 124.6 117.1 116.9 112.6 112.5
58.9 54.2 53.6
37.9 37.2 33.8 32.3 32.1 32.0 28.3
N
N
O
F
Me
51
220 210 200 190 180 170 160 150 140 130 120 110 100 90 80 70 60 50 40 30 20 10 0 -10 -20
f1 (ppm)

2-(1-(4-(benzo[d][1,3]dioxol-5-ylmethyl)piperazin-1-yl)-2-(4-chlorophenoxy)ethyl)-5-(5-chloro-2-(methylthio)pyrimidin-4-yl)-1,3,4-oxadiazole - 52

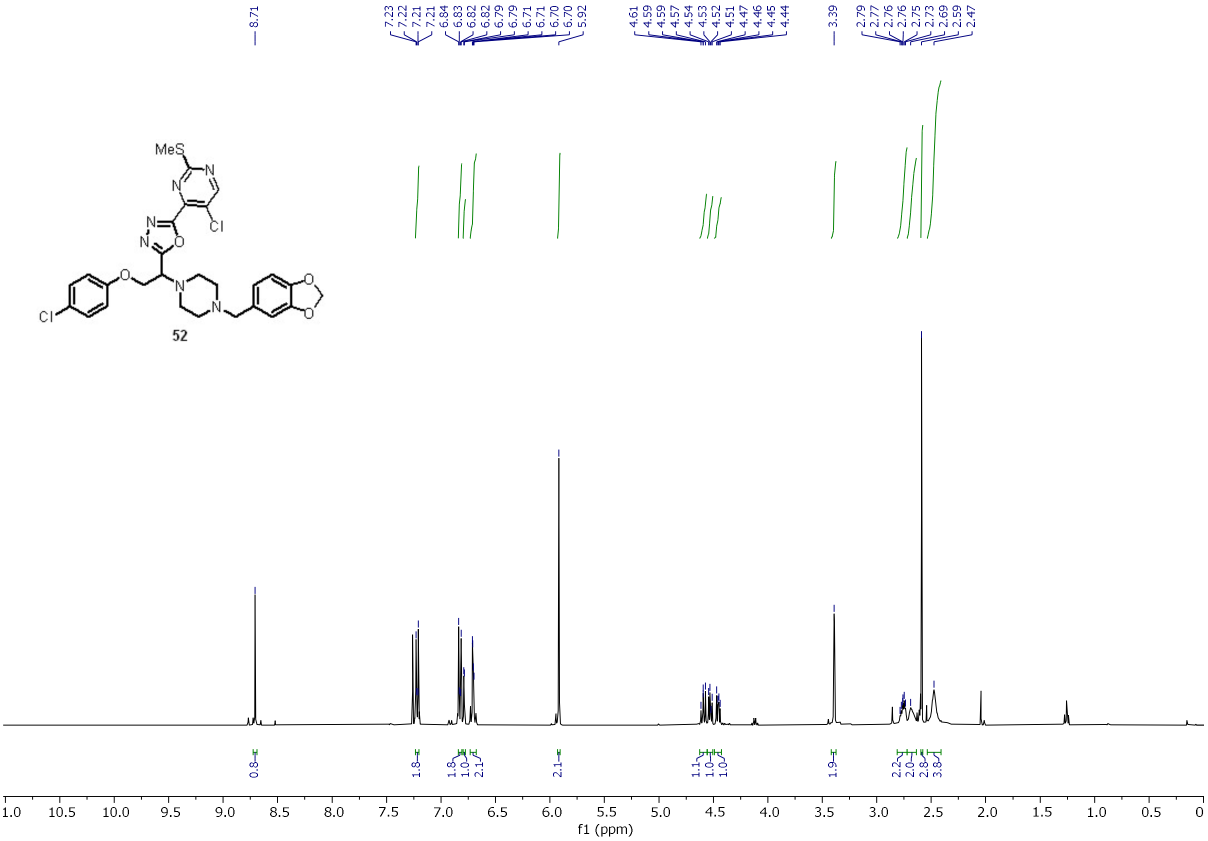

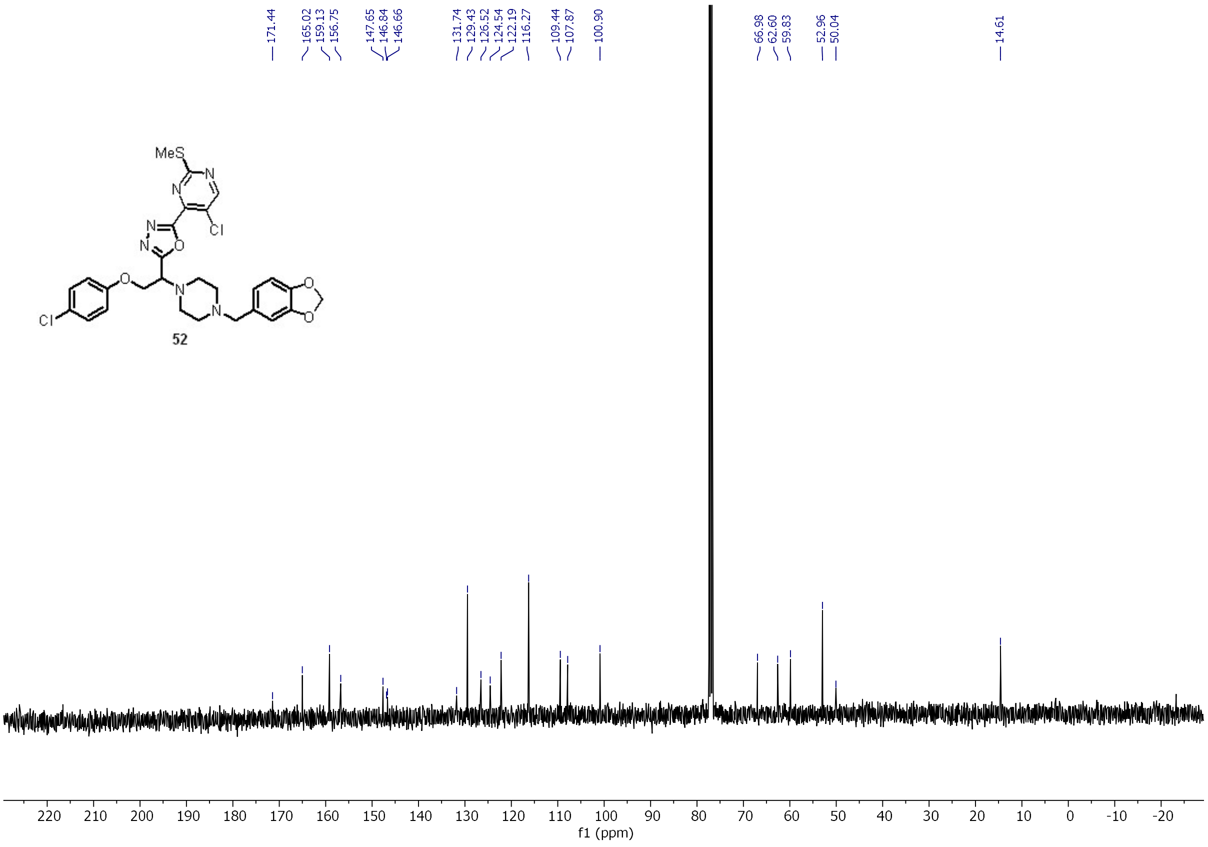

4-(5-((2,3-dihydrobenzo[b][1,4]dioxin-6-yl)(piperidin-1-yl)methyl)-1,3,4-oxadiazol-2-yl)-N,N-dipropylbenzenesulfonamide - 53

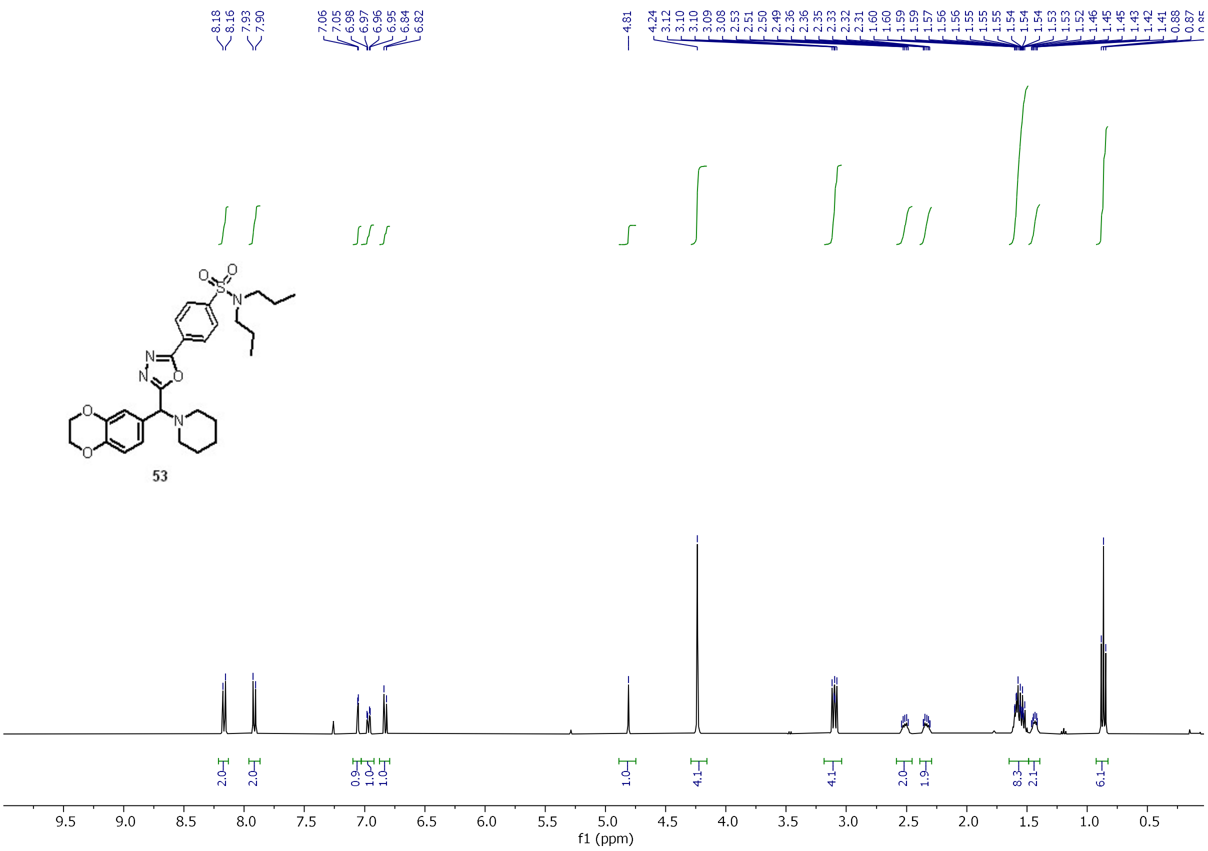

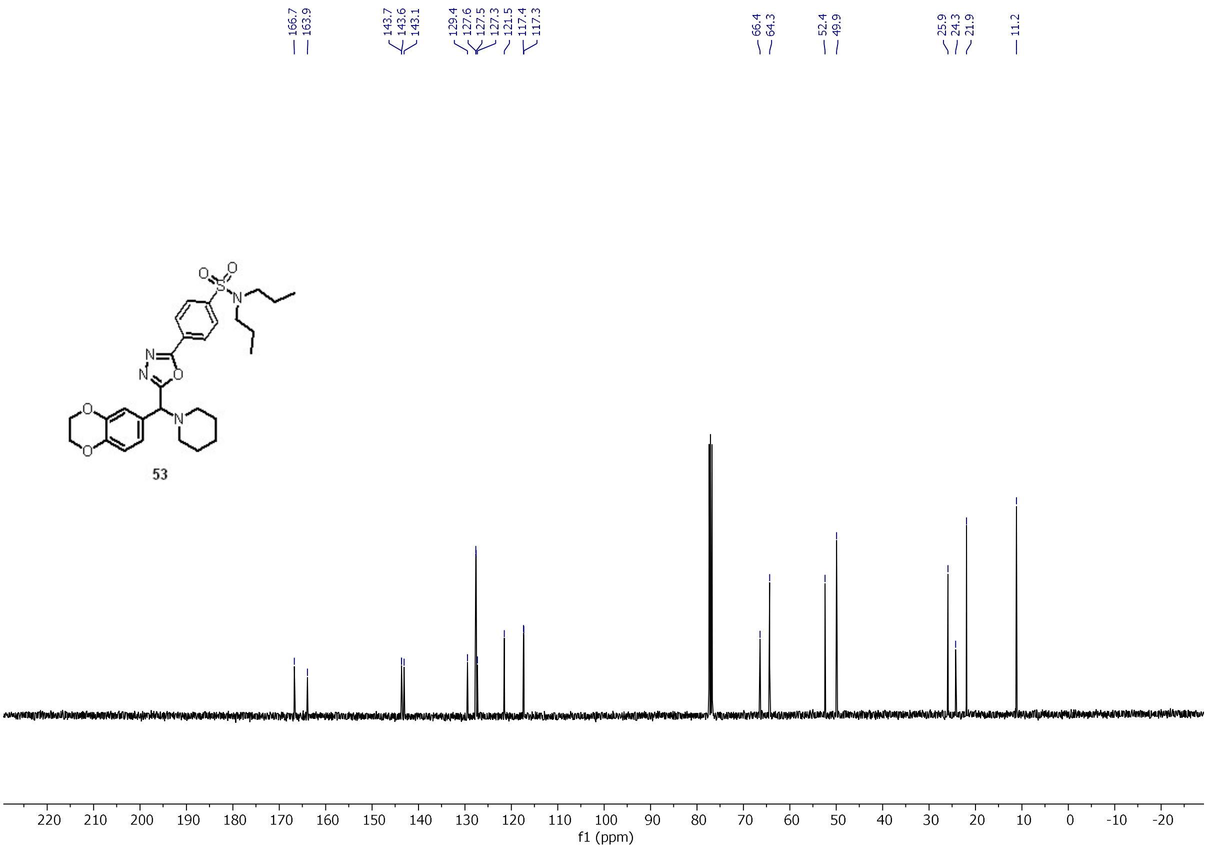

4-(5-((2,3-dihydrobenzo[b][1,4]dioxin-6-yl)(piperidin-1-yl)methyl)-4-tosyl-4H-1,2,4-triazol-3-yl)-N,N-dipropylbenzenesulfonamide - 54

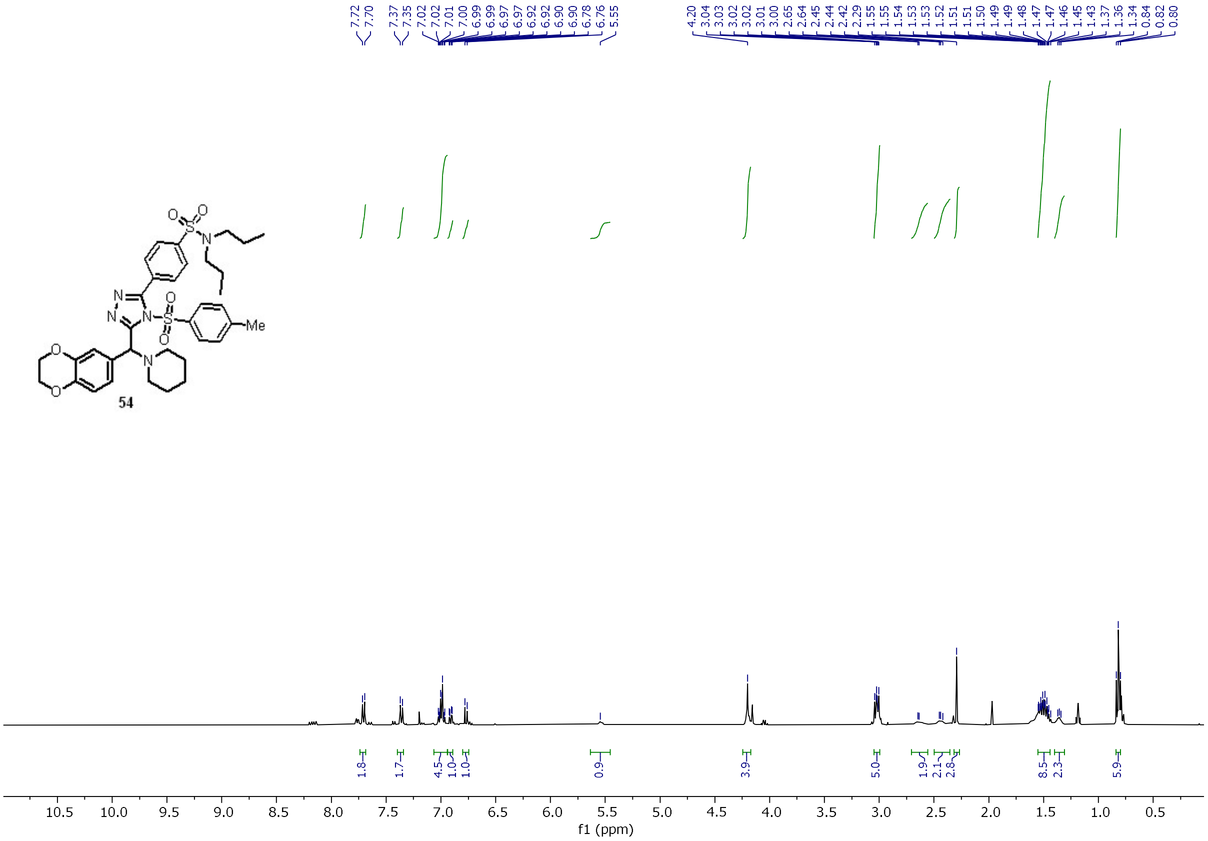

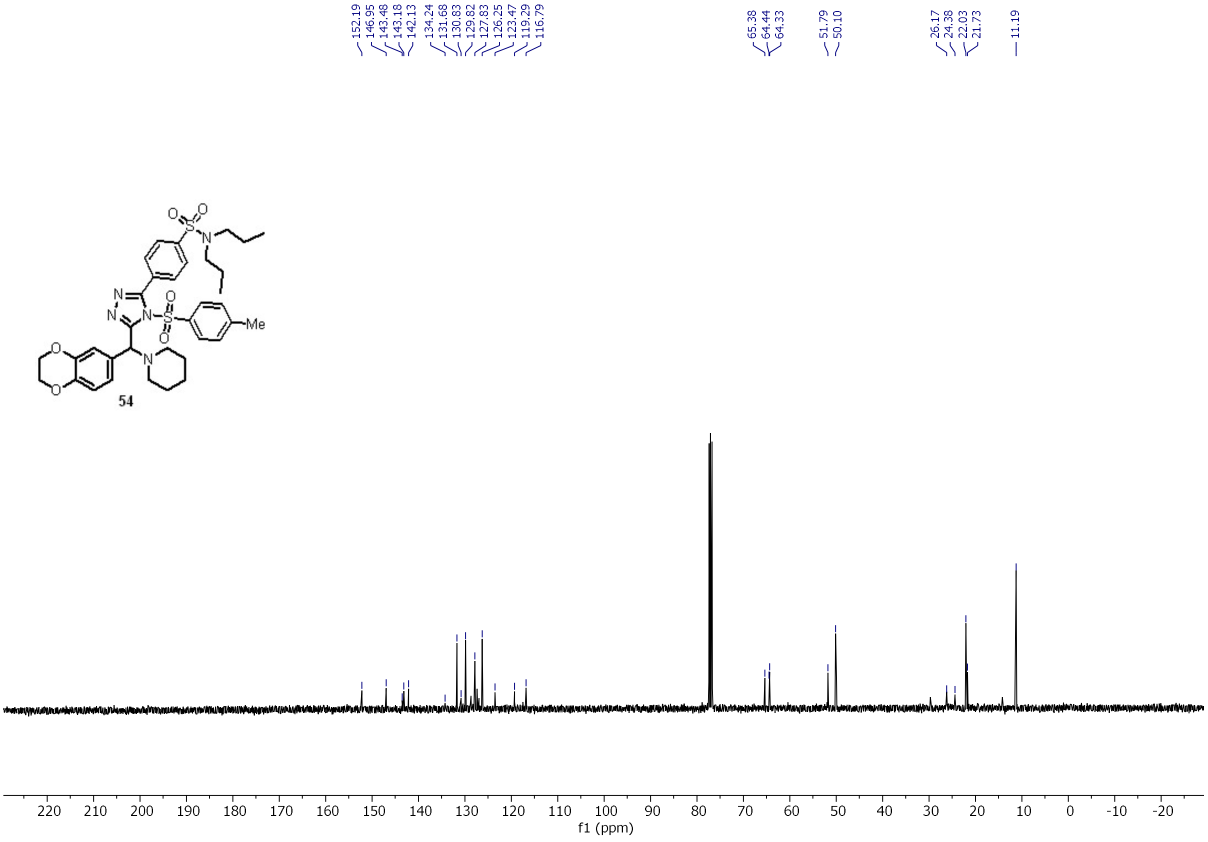

4-(5-((2,3-dihydrobenzo[b][1,4]dioxin-6-yl)(piperidin-1-yl)methyl)-1,3,4-thiadiazol-2-yl)-N,N-dipropylbenzenesulfonamide - 55

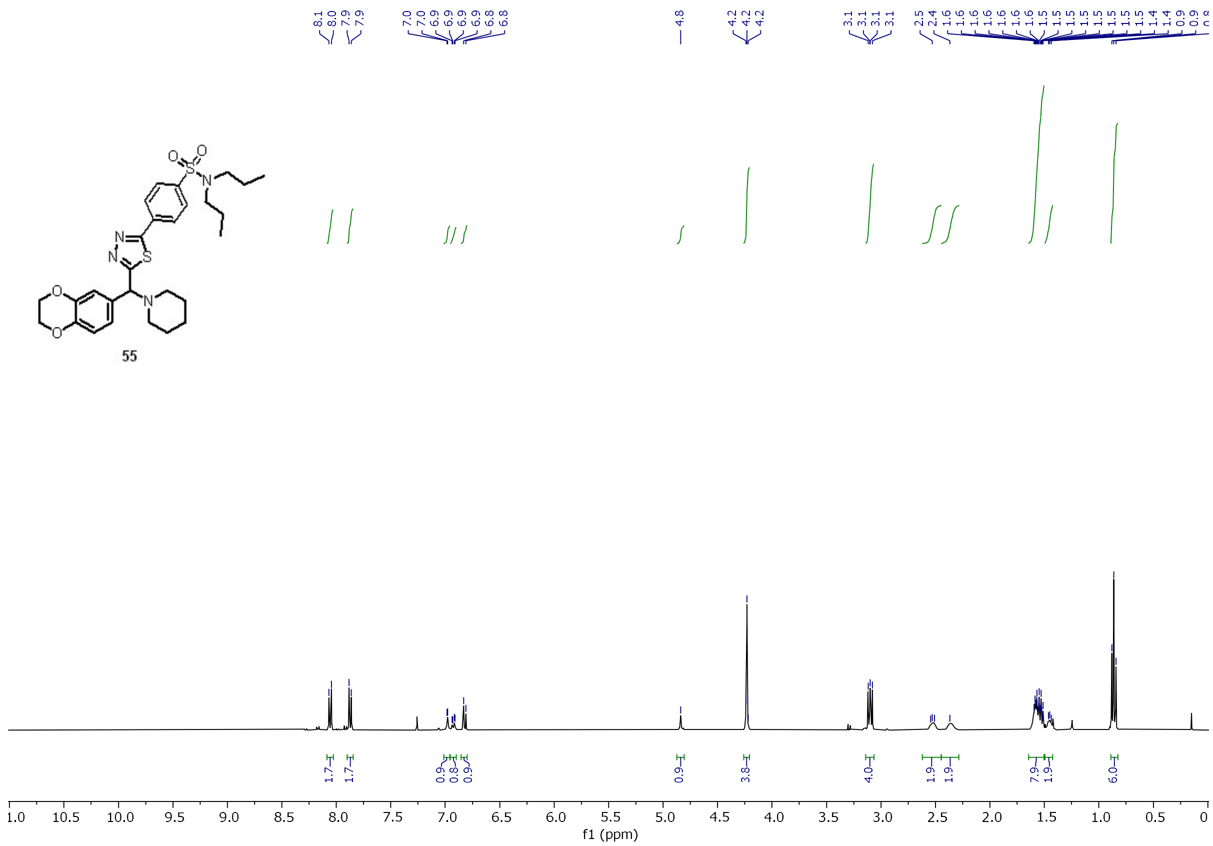

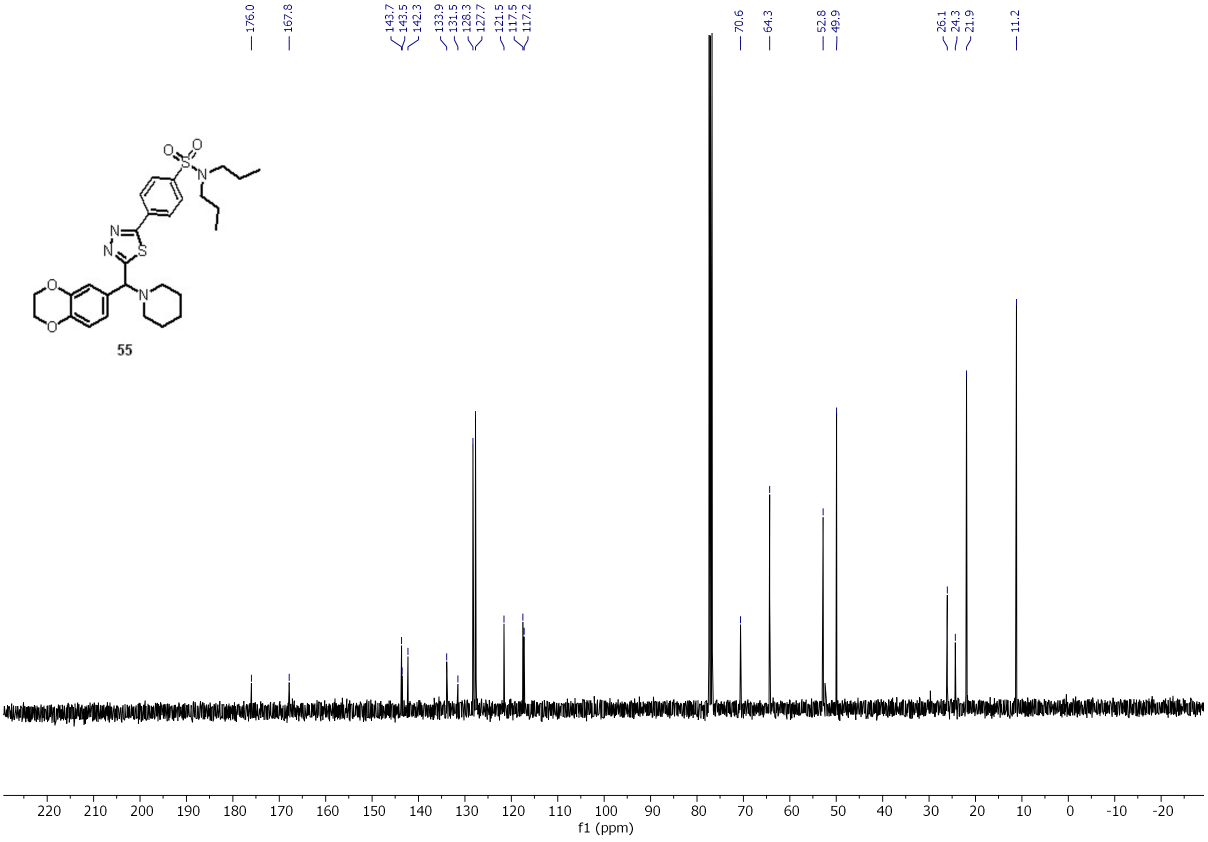

6. References

(1) Á. L. Fuentes de Arriba, E. Lenci, M. Sonawane, O. Formery, D. J. Dixon, *Angew. Chem., Int. Ed.* **2017**, *56*, 3655-3659.

(2) L.-G. Xie, D. J. Dixon, *Chem. Sci.* **2017**, *8*, 7492-7497.

(3) L.-G. Xie, D. J. Dixon, *Nat. Commun.* **2018**, *9*, 2841.

(4) T. Rogova, P. Gabriel, S. Zavitsanou, J. A. Leitch, F. Duarte, D. J. Dixon, *ACS Catal.* **2020**, *10*, 11438-11447.

(5) M. M. Bio, G. Javadi, Z. J. Song, *Synthesis* **2005**, 19-21.

(6) X. Zhang, D. W. C. MacMillan, *J. Am. Chem. Soc.* **2016**, *138*, 13862-13865.

(7) L. Yang, S. Li, L. Cai, Y. Ding, L. Fu, Z. Cai, H. Ji, G. Li, *Org. Lett.* **2017**, *19*, 2746-2749.

(8) K.-I. Tanaka, S. Yoshifuji, Y. Nitta, *Chem. Pharm. Bull.* **1988**, *36*, 3125-3129.

(9) K. Osowska-Pacewicka, S. Zawadzki, A. Zwierzak, *Phosphorus Sulfur Silicon Relat. Elem.* **1993**, *82*, 49-54.

(10) N. Ohmura, A. Nakamura, A. Hamasaki, M. Tokunaga, *Eur. J. Org. Chem.* **2008**, *2008*, 5042-5045.
